# Localized Phylogenetic Discordance Among Nuclear Loci Due to Incomplete Lineage Sorting and Introgression in the Family of Cotton and Cacao (Malvaceae)

**DOI:** 10.3389/fpls.2022.850521

**Published:** 2022-04-13

**Authors:** Rebeca Hernández-Gutiérrez, Cássio van den Berg, Carolina Granados Mendoza, Marcia Peñafiel Cevallos, Efraín Freire M., Emily Moriarty Lemmon, Alan R. Lemmon, Susana Magallón

**Affiliations:** ^1^Posgrado en Ciencias Biológicas, Universidad Nacional Autónoma de México, Mexico City, Mexico; ^2^Departamento de Botánica, Instituto de Biología, Universidad Nacional Autónoma de México, Mexico City, Mexico; ^3^Departamento de Ciencias Biológicas, Universidade Estadual de Feira de Santana, Feira de Santana, Brazil; ^4^Herbario Nacional del Ecuador (QCNE), Instituto Nacional de Biodiversidad, Quito, Ecuador; ^5^Department of Biological Science, Florida State University, Tallahassee, FL, United States; ^6^Department of Scientific Computing, Florida State University, Tallahassee, FL, United States

**Keywords:** gene tree congruence, Malvaceae, molecular heterogeneity, phylogenomic dating, phylogenetic discordance, species tree

## Abstract

The economically important cotton and cacao family (Malvaceae *sensu* lato) have long been recognized as a monophyletic group. However, the relationships among some subfamilies are still unclear as discordant phylogenetic hypotheses keep arising when different sources of molecular data are analyzed. Phylogenetic discordance has previously been hypothesized to be the result of both introgression and incomplete lineage sorting (ILS), but the extent and source of discordance have not yet been evaluated in the context of loci derived from massive sequencing strategies and for a wide representation of the family. Furthermore, no formal methods have been applied to evaluate if the detected phylogenetic discordance among phylogenomic datasets influences phylogenetic dating estimates of the concordant relationships. The objective of this research was to generate a phylogenetic hypothesis of Malvaceae from nuclear genes, specifically we aimed to (1) investigate the presence of major discordance among hundreds of nuclear gene histories of Malvaceae; (2) evaluate the potential source of discordance; and (3) examine whether discordance and loci heterogeneity influence on time estimates of the origin and diversification of subfamilies. Our study is based on a comprehensive dataset representing 96 genera of the nine subfamilies and 268 nuclear loci. Both concatenated and coalescence-based approaches were followed for phylogenetic inference. Using branch lengths and topology, we located the placement of introgression events to directly evaluate whether discordance is due to introgression rather than ILS. To estimate divergence times, concordance and molecular rate were considered. We filtered loci based on congruence with the species tree and then obtained the molecular rate of each locus to distribute them into three different sets corresponding to shared molecular rate ranges. Bayesian dating was performed for each of the different sets of loci with the same parameters and calibrations. Phylogenomic discordance was detected between methods, as well as gene histories. At deep coalescent times, we found discordance in the position of five subclades probably due to ILS and a relatively small proportion of introgression. Divergence time estimation with each set of loci generated overlapping clade ages, indicating that, even with different molecular rate and gene histories, calibrations generally provide a strong prior.

## Introduction

Deep, conflicting phylogenetic relationships are often found in angiosperm clades, and the advancement of molecular sequencing of large amounts of loci from different compartments, as well as the thorough application of phylogenetic and coalescence methods, have greatly contributed to solve some of them (e.g., Wang et al., 2019b; Koenen et al., 2020; Cai et al., 2021; Jost et al., 2021). Nevertheless, in some cases, even with numerous genes, phylogenetic relationships remain unsolved or poorly supported due to the high incongruence among gene histories and the obscuring signal of past evolutionary processes such as incomplete lineage sorting (ILS) and reticulation, e.g., in Amaranthaceae s.l. (Morales-Briones et al., 2021).

An important aspect to consider when using hundreds or thousands of loci is that molecular rate heterogeneity increases; thus, phylogenetic tree inference should consider incongruence and molecular rate heterogeneity (Dornburg et al., 2019). Phylogenies represent the basis for downstream evolutionary analyses, such as divergence time estimation, which uses molecular clock models that are sensitive to rate heterogeneity, biasing age estimates if rate heterogeneity is not considered appropriately (Angelis et al., 2018; Smith et al., 2018; Carruthers et al., 2020). The test of evolutionary hypotheses is hindered by the intricate phylogenetic relationships, which would show equivocal or unconclusive results on, for example, the origin and diversification of lineages, or ancestral state and biogeographic area reconstructions.

The family Malvaceae is the largest in the order Malvales, with 4,465 species and 245 genera (The Plant List, 2021) distributed in nine subfamilies, which comprise the traditional families Sterculiaceae, Tiliaceae, Bombacaceae, and Malvaceae *sensu stricto* (Alverson et al., 1999; Bayer et al., 1999). Many members of the family are important components of tropical ecosystems, and some others are of high economic importance (e.g., cotton, chocolate, cola nut, and durian). Malvaceae is highly diverse in growth forms, fruit types, floral morphology, and geographic and biome distribution. Understanding how this family evolved to reach such a high variation is a challenging task, starting from the phylogeny, since recalcitrant discordance in the relationships among some subfamilies, i.e., Helicterioideae, Sterculioideae, Tilioideae, Dombeyoideae, and Brownlowioideae (Alverson et al., 1999; Bayer et al., 1999; Nyffeler et al., 2005; Richardson et al., 2015; Hernández-Gutiérrez and Magallón, 2019; Cvetković et al., 2021), weakens the possible hypotheses about its evolution. The evolution of Malvaceae seems to be highly complex because nuclear genes show a different history from the plastome, but differences in the same genomic compartment are also present (Conover et al., 2019; Cvetković et al., 2021; Hernández-Gutiérrez et al., 2021; Wang et al., 2021). Importantly, there is no consensus on the relationships among some subfamilies due to conflicting, but highly supported resolutions, as observed in past but mostly in recent studies (Conover et al., 2019; Hernández-Gutiérrez and Magallón, 2019; Cvetković et al., 2021; Hernández-Gutiérrez et al., 2021; Wang et al., 2021).

Within Malvaceae, multiple whole-genome multiplications (WGM) have occurred, as observed by analyzing genomic data (Paterson et al., 2012; Wang et al., 2019a) but also inferred through chromosome counting (e.g., Costa et al., 2017). It has been hypothesized that deep reticulations gave rise to some major lineages of Malvaceae and that some of the resulting conflicting relationships are caused by ILS (Conover et al., 2019). The extent at which these two sources of phylogenetic discordance are causing of the contradictory hypotheses of Malvaceae phylogeny remains unknown. To analyze this question, numerous nuclear genes, and a larger taxon sampling, have the potential to inform about past processes underlying the intricate relationships among subfamilies of Malvaceae.

The timing of evolution of Malvaceae was previously estimated in a comprehensive study of the order Malvales, mostly based on plastid molecular markers (Hernández-Gutiérrez and Magallón, 2019). Although nuclear genes can potentially modify estimates of phylogenetic relationships, and phylogenomic data commonly violate molecular clock model assumptions, both factors consequently affect age estimates (Angelis et al., 2018). Because accurate divergence time estimations represent a framework to further analyze lineage evolution, here we examine to what extent gene conflict and molecular rate heterogeneity impact the divergence time estimation of Malvaceae. Using a comprehensive taxon sampling, our objective was to reconstruct the phylogenetic relationships of Malvaceae from nuclear genes. The specific aims of this study were to (1) investigate the presence of major phylogenetic discordance among hundreds of nuclear gene histories of Malvaceae; (2) evaluate the extent to which reticulation and ILS are causing discordance; and (3) to estimate divergence times considering discordance and heterogeneity in gene histories.

## Materials and Methods

### Plant Material, Taxon Sampling, and DNA Extraction

DNA extraction was performed from silica dried tissue, as well as herbarium material (Supplementary Table S1), with a modified CTAB protocol (Doyle and Doyle, 1987) that includes an additional treatment with RNAse A (Qiagen, Mexico City, Mexico) and proteinase K (recombinant, 1 mg/ml; Thermo Scientific, Mexico City, Mexico). The extraction and molecular procedures of Brazilian samples (Supplementary Table S1) were done at Laboratório de Sistemática Molecular de Plantas (LAMOL), Universidade Estadual de Feira de Santana. We included 96 species, each from a different genus, representing the nine subfamilies of Malvaceae s.l. (Supplementary Table S1). Nine species belonging to other families in the order Malvales were included as outgroups (Supplementary Table S1). To build a phylogenetic tree with a concatenated matrix, *Neurada procumbens* was selected for rooting the tree, following results obtained in a previous study (Hernández-Gutiérrez and Magallón, 2019). However, for rooting phylogenetic gene trees, different outgroups were selected because individual loci alignments have different taxon sampling due to sequencing capture variations (see details for each analysis below).

### Plant Anchored Enrichment Strategies

Molecular data were generated through two target enrichment strategies in the Center for Anchored Phylogenomics at Florida State University.^1^ Both strategies used the Angiosperm v.1 probe kit (Buddenhagen et al., 2016) which targets 499 nuclear exons that were found to be present in low or single copy in several species well distributed across the angiosperm phylogeny and 18 additional exons corresponding to selected selenium-tolerance genes. The rationale behind the design of this probe set is explained in detail by its authors (Buddenhagen et al., 2016), as well as in studies applying this kit to other angiosperm lineages (Lamiaceae: Fragoso-Martínez et al., 2017; Aristolochiaceae: Wanke et al., 2017). In general, the two strategies followed the same wet-lab procedures for library preparation, enrichment, and sequencing, which in summary were as follows. A Covaris E220 Focused-ultrasonicator was used to shear the DNA to a fragment size of 300–800 bp. A modification of the protocol of Meyer and Kircher (2010) was used to bind the adapters and indexes to the fragmented DNA with a Beckman-Coulter Biomek FXp liquid-handling robot. Indexed samples were pooled to carry out solution-based enrichment reactions with the Angiosperm v. 1 probe kit (Agilent Technologies Custom SureSelect XT kit), following manufacturer’s protocol. Streptavidin coated magnetic beads were used to separate the enriched DNA fragments from the remaining genomic DNA. The enrichment strategies differ from each other in how indexes were assigned during the library preparation step. In the first strategy, each species was first linked to a unique index and then pooled with other species for enrichment, as it is conventionally done. In the second strategy, six distantly related angiosperm species (among them one species of Malvaceae) were first pooled and then assigned a single index prior to enrichment, a method called Anchored MetaPrep (Lemmon, 2015). In the present study, five control samples were processed with both enrichment strategies and incorporated in the phylogenetic analyses to cross-validate the use of both data sources. Enrichment reactions from both strategies were sequenced in one PE150 Illumina HiSeq 2500 lane at the Translational Science Laboratory in the College of Medicine at Florida State University, Tallahassee, Florida, United States.

### Read Processing, Assembly, Orthology Assessment, and Alignment

All methods described in this section were performed in the Center for Anchored Phylogenomics. A detailed explanation of the bioinformatic methods employed can be found in Granados Mendoza et al. (2020), but in short, low-quality raw reads were filtered out with the CASAVA v. 1.8 pipeline using a high-chastity setting. Read demultiplexing was performed by ensuring perfect matches to one of 13 indexes developed in-house and reads with ambiguous matches were excluded. We used the method proposed by Rokyta et al. (2012) for read merging, because this method prevents merging at highly repetitive regions. Assembly followed the *quasi-de novo* strategy and used the Assembler.java program of Prum et al. (2015), with both merged and unmerged reads. The assembler first performs a divergent reference assembly, where reads are mapped to conserved regions of the target loci using three distantly related species to our target group (i.e., *Arabidopsis thaliana*, *Billbergia nutans*, and *Carex lurida*) that were included in the probe set design by Buddenhagen et al., 2016. Then, a second *de novo* assembly is carried out, where reads assembled in the first step serve as references to extend the assembly into the more variable flanking regions. Unambiguous base calls were assumed if no polymorphism was observed or if polymorphisms could be attributed to sequencing errors, assuming a binomial probability model with a probability of error = 0.1 and alpha = 0.05 (Buddenhagen et al., 2016). Heterozygous sites were coded following the IUPAC ambiguity codes, and if coverage was below 10, bases were called as N. To avoid cross contamination and inclusion of potential sequencing errors, assembled contigs with <30× mean coverage were excluded. Orthology assessment followed Prum et al. (2015) and was performed by grouping sequences by locus and calculating a distance matrix, where pairwise distances between two sequences corresponded to the percent of 20-mers found in both sequences. These distance matrices were then used to cluster sequences using the neighbor-joining algorithm (Saitou and Nei, 1987). If a single cluster was produced, we assumed no gene duplication for that specific locus. If more than one cluster was obtained, each cluster was considered as a different locus. Only clusters with more than 50% of the target species were used in further steps. MAFFT v.7.023b (Katoh and Standley, 2013) was used to generate preliminary alignments that were subsequently trimmed following Prum et al. (2015) and Hamilton et al. (2016). For trimming, an alignment site was considered as “good” when the most prevalent character state was shared across >50% of the sequences, then regions of 20 bp of each sequence were masked if they contained less than 15 “good” sites, and finally, sites having less than 56 unmasked bases were trimmed. The bioinformatic process of the data derived from the Anchored MetaPrep method follows Lemmon (2015). A total of 268 nuclear loci alignments were obtained after merging the information retrieved from both enrichment strategies (Supplementary File 1).

### Concatenated Phylogeny

We aimed at constructing a phylogeny with a concatenated matrix. For this, we concatenated all loci in R (R Core Team, 2020) with the chopper package^2^ and transformed this alignment to NEXUS format with the ips package (Heibl et al., 2019). To estimate the substitution model for each locus, we used PartitionFinder2 (Lanfear et al., 2016) implemented in the CIPRES Gateway (Miller et al., 2010), all models were evaluated with the “greedy” algorithm (Lanfear et al., 2012) and using RAxML (Stamatakis, 2014) for phylogenetic inference. The model GTR + I + G was identified as best-fitting for most of the loci. We conducted maximum likelihood (ML) inference with the concatenated matrix with RAxML v. 8.2.12 in the BEAGLE server from the Instituto de Biología of the National Autonomous University of Mexico (UNAM), using the partition sets that resulted from PartitionFinder2, tree search was set to 10. Bootstrap support for nodes was evaluated with 1,000 replicates. The nine species of the other Malvalean families were assigned as the outgroup.

To assess the support of individual loci in relation to each node, we took the ML topology and generated reverse constraints for the 110 nodes of the tree. Then, heuristic searches for each constraint and an unconstrained topology were performed with maximum parsimony (MP), followed by the inclusion of each locus individually, in order to assess their relative contribution to each node, in an analogous fashion to Lee et al. (2011). The resulting logs were processed with TreeRot v. 3 (Sorenson and Franzosa, 2007) to generate trees with individual values for each locus, and with a custom python script we extracted data from the trees with all the values (loci/nodes). With this data, we calculated for each locus: (1) number of nodes with positive values (supporting locus), (2) number of nodes with negative values (conflicting locus), (3) positive–negative, and (4) sum of all individual scores. For the nodes, we calculated (1) number of loci with positive values, (2) number of loci with negative values, (3) positive–negative, and (4) sum of individual scores, which corresponds to the overall Bremer support for that node.

### Species Tree Estimation

We performed a site-based analysis (i.e., without *a priori* specification of gene trees) with the concatenated matrix to estimate the species tree under the multispecies coalescent model (MSC) conducted in SVDquartets (Chifman and Kubatko, 2014) implemented in PAUP* v.4.0a166 (Swofford, 2002). The evaluation was performed for a maximum of 100,000 random quartets and statistical support for nodes was assessed by the calculation of 1,000 bootstrap replicates.

A summary coalescence method was also implemented. For this, we first estimated phylogenetic trees for each locus with maximum likelihood in RAxML v.8.2.12 (Stamatakis, 2014), setting GTR+G as the substitution model, 100 tree searches, and 1,000 bootstrap replicates. Bifurcations with bootstrap support ≤20 were collapsed with the program nw_ed of Newick Utilities v.1.6 (Junier and Zdobnov, 2010). A file containing the gene trees with low supported branches collapsed was the input for ASTRAL-III v.5.7.3 (Zhang et al., 2018). The support for branches was evaluated with local posterior probability (LPP).

### Phylogenetic Discordance Source

To explicitly evaluate the extent to which reticulation and ILS are causing phylogenetic discordance we used QuIBL (Quantifying Introgression *via* Branch Lengths; Edelman et al., 2019). For each triplet of species, QuIBL extracts the frequency of topologies formed by that triplet in all gene trees. Each triplet topology has one internal branch (considering one and the same outgroup for all the triplets) and QuIBL calculates the likelihood of two distribution models of the length of this branch. One model considers that the branch length derives from a proportion of ILS only, and the second model considers ILS plus the proportion of introgressed loci. Both models are examined for each triplet and are evaluated with Bayesian Information Criterion (BIC). In this study, a reduced taxon sampling was used because (1) this analysis requires that all species are present in every gene tree, and (2) we wanted to evaluate the discordance at a deep phylogenetic level, i.e., at the divergence of subfamilies. To reduce our taxon sampling, we used the R package treeplyr v.0.1.10 (Uyeda and Harmon, 2020) to prune the trees corresponding to the selected sampling of species. Thus, for this analysis, we used 123 gene trees from RAxML, each tree with 18 species representing the nine subfamilies and one species as the outgroup for all the triplets (*Muntingia calabura*). The ASTRAL tree was used for interpreting QuIBL results by distinguishing topologies that were discordant from those that resembled this species tree.

### Divergence Time Estimation

Molecular dating based on genomic data (i.e., hundreds or thousands of genes) may be challenging, as gene histories and molecular rate could be highly heterogeneous (Carruthers et al., 2020). This heterogeneity produces two general issues in molecular dating. One of them is the usual violation of the molecular clock model, exacerbated as more data are included, making it difficult to obtain accurate estimates (Smith et al., 2018; Carruthers et al., 2020). One solution to this issue is the “gene shopping” approach (Smith et al., 2018), where genes or loci are selected if they behave in a more clock-like fashion, with respect to other loci. The other issue is that applying one clock model to a large dataset may yield wrong estimates due to high substitution rate heterogeneity (Angelis et al., 2018; Nie et al., 2020), which may be solved by partitioning the data set in different clock regimes (Nie et al., 2020).

Here, we aimed to identify the extent of rate heterogeneity in our molecular dataset and whether this impacts age estimates. For this, we applied a combination of approaches to overcome gene history conflict and both heterogeneity issues, first by dividing the complete loci dataset in sets of loci that differ in substitution rate variation (attending the molecular clock issue) and by applying different clock models to each of these sets of loci (addressing the issue of one model fitting high heterogeneity). We compared the results among three sets of loci that differ in rate variance, additionally comparing a fourth analysis with the concatenated dataset but partitioned by the three sets of loci, and a fifth analysis of few loci with low rate variance. The next sections describe the filtering of loci and analyses.

#### “Gene Shopping”: Data Filtering

First, we used SortaDate (Smith et al., 2018) scripts to sort gene molecular behavior, following a “gene shopping” framework (Smith et al., 2018). SortaDate scripts were implemented in python 2.7 and it was used along with the software phyx (Brown et al., 2017) to select those loci that shared similar rate variation. The input files were the individual, rooted gene trees, which we obtained from the RAxML analyses described above (268 trees), and the rooted species tree, which was the ASTRAL species tree because it is fully resolved. Species and gene trees were rooted with the pxrr function from the phyx software (Brown et al., 2017). We sorted the trees based on the proportion of bipartitions shared with the species tree, then by the root-to-tip variance, and lastly by tree length.

From the results of SortaDate, we set the arbitrary criterium to select those trees that had at least 0.3 proportion of bipartitions corresponding to the species tree, which resulted in 123 gene trees. From this set, we calculated terciles from the root-to-tip variance and obtained three sets of 41 trees each. Thus, the first tercile has a low variance and the third tercile the highest variance. Note that the molecular rate variance was not necessarily related to the proportion of bipartitions (i.e., gene tree discordance). The sequence alignments of individual loci corresponding to the selected sets of trees were then concatenated to perform dating analyses (three molecular matrices each with 41 loci). Additionally, we wanted to analyze if applying a molecular clock model to different partitions affects the estimates, so we concatenated the three sets of loci, obtaining a molecular matrix with 123 loci with three partitions. Moreover, to examine if the homogeneity and number of loci affect the estimates, we selected five loci corresponding to those that had the lowest rate variance (i.e., closer to a strict clock fashion) and built a fifth molecular matrix.

#### Dating Analyses

We estimated divergence times with BEAST2 v.2.6.3 (Bouckaert et al., 2019). We performed five dating analyses: one for each set of 41 concatenated loci, one for loci of all three sets partitioned by set, and another with five loci with the lowest molecular rate variance to evaluate whether estimates are affected when using the least heterogeneous molecular dataset, that is, fitting to a single clock regime (“clock-likeness” approach). We applied the following settings to the five analyses. In BEAUti v.2.6.3, we implemented a GTR + G molecular substitution model, using empirical base frequencies, molecular clock set as uncorrelated with rates obtained from a log-normal prior distribution (UCLN; Drummond et al., 2006) and a birth-death tree prior. We constrained the topology to resemble the analysis with ASTRAL only for the highly supported subfamilies and major clades (i.e., all subfamilies belonging to a major group), but left unconstrained the relationships among and inside these clades.

We constrained subfamilies to be monophyletic, this excluded Helicterioideae and Byttnerioideae; relationships within subfamilies were not constrained. We applied a secondary calibration to the root of the tree, i.e., the crown node of Malvales, as a uniform prior distribution with minimum value of 110.48 Ma and maximum value of 138.33 Ma, as obtained from the BEAST analysis performed by Ramírez-Barahona et al. (2020). Eight calibrations informed by the fossil record (Supplementary Table S2) were applied to the crown group of Malvaceae and to most of the subfamilies. To set the calibrations, we used uniform distributions with the minimum value being the upper bound of the stratigraphic epoch of each fossil, and the maximum value 138.33 Ma (the maximum value assigned to the root). We ran two independent analyses with 500–600 million generations each, sampling parameters every 5,000 steps. The analyses in BEAST2 were performed in the server BEAGLE of Instituto de Biología (UNAM). For each analysis, the resulting estimates were summarized in LogCombiner v.2.6.3, removing 20% of the samples as burn-in of the posterior parameter values, and 70% of the posterior sampling of trees. The Maximum Clade Credibility (MCC) tree and node mean heights were obtained in TreeAnnotator v.2.6.3. The MCC tree of each analysis was visualized in FigTree v.1.4.4^4^ and their annotated data were extracted with the R package treeio (Wang et al., 2020) for comparison among the four different analyses. Finally, we tested whether the prior settings were constraining the estimates by running an analysis without considering a molecular dataset and only including the prior specifications.

## Results

### Taxon and Genetic Sampling

In this study, 96 species of Malvaceae and nine outgroup species representing other families of Malvales were considered (Supplementary Table S1). By integrating the results from the two enrichment strategies, we obtained 268 potentially single-copy nuclear loci. From the complete 268 gene sampling, 28 were captured only through the conventional AHE method, whereas the rest were captured with both techniques (Supplementary Table S1).

### Concatenated Phylogeny

**Figure 1:**
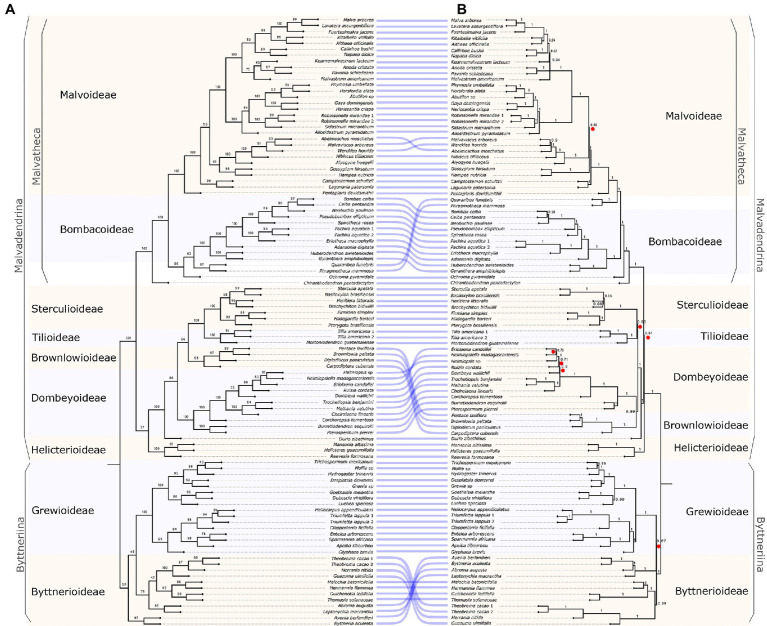
Species trees of Malvaceae derived from two coalescence methods. **(A)** Species tree from SVDquartets. Numbers associated to nodes represent Bootstrap values. **(B)** Species tree from ASTRAL. Numbers associated to nodes represent local posterior probabilities (LPP). Red circles indicate relationships with low quartet score (<40%). To visualize similarities between the two analysis, purple lines connect species between trees.

In the concatenated dataset of all 268 nuclear loci, the representatives of the nine non-Malvaceae families were designated as outgroup, but the relationships among them were mostly weakly supported (Supplementary Figure S1). The only highly supported (100 Bootstrap support, BS) relationship was between *M. calabura* (Muntingiaceae) and *Bdallophytum americanum* (Cytinaceae). Within Malvaceae, two clades are recovered, Byttneriina and Malvadendrina. Byttneriina comprises two monophyletic subfamilies, Grewioideae and Byttnerioideae. Grewioideae is strongly supported, as well as the relationships within it, whereas Byttnerioideae is moderately supported (72 BS) as a monophyletic group. The rest of the subfamilies are included in Malvadendrina (Figure 1). Most members of Helicterioideae (except *Durio zibethinus*) form a clade that is the sister group of the rest of the subfamilies, which form two groups. One group comprises *D. zibethinus* as the sister taxon of a group formed by the monophyletic, highly supported subfamilies Sterculioideae, Tilioideae, and Brownlowioideae + Dombeyoideae. The other group is Malvatheca (Figure 1), where *Chiranthodendron pentadactylon* is the sister of the remaining members of the group, and *Ochroma pyramidale* is the sister taxon of Bombacoideae + Malvoideae (Supplementary Figure S1).

We retrieved relatively high Bremer support for all the loci, indicating low conflict (Supplementary Table S3). Of all the loci, only one presented more conflicting than supported nodes (L256), but its overall sum of supports is positive. On the other hand, two loci presented more supported than conflicting nodes (L149 and L129) but with an overall negative sum of supports. All other loci have support for most of the nodes, and the fact that the overall sums of support values for either loci or nodes are always positive, indicates that even when there are some negative values, these are of smaller magnitude in relation to the positive support for all loci.

### Species Tree Estimation

The results obtained with SVDquartets (Figure 1A) yielded many weakly supported bipartitions due to discordance in the bootstrap replicates. Highly supported (85–100 BS) clades were (1) Malvaceae as a whole; (2) Grewioideae; and (3) a clade containing *Durio zibethinus* as sister to Dombeyoideae, Brownlowioideae, Tilioideae, and Sterculioideae. These subfamilies are strongly supported as monophyletic, but the relationships among them are poorly supported (Figure 1A). Another group of highly supported relationships include (1) the Malvatheca clade; (2) *Chiranthodendron* as the sister lineage of the rest of Malvatheca species; (3) *Ochroma* as sister to *Quararibea* + *Phragmotheca* and Bombacoideae; and (4) Malvoideae.

The resulting species tree from the ASTRAL analysis (Figure 1B) shows strongly supported clades (1 LPP), such as Byttneriina and Malvadendrina. Within Byttneriina, highly supported clades are Grewioideae and part of Byttnerioideae (i.e., excluding tribe Byttnerieae, here comprising *Leptonychia*, *Byttneria,* and associated genera). The relationship of Helicterioideae (excluding *Durio*) and the rest of Malvadendrina are poorly supported (0.61 LPP). Within Malvadendrina, *Durio* is strongly supported as the sister of a clade comprising the subfamilies Sterculioideae, Tilioideae, Brownlowioideae, and Dombeyoideae. Brownlowioideae and Dombeyoideae are highly supported (1 LPP) as sister clades, and Sterculioideae and Tilioideae are moderately supported (0.83 LPP). Strongly supported relationships within Malvatheca are the placement of *Chiranthodendron* as the sister to the remaining species of Malvatheca, and successively *Ochroma* as the sister to the remaining species. *Quararibea* + *Phragmotheca* forms a clade that is sister to the Malvoideae.

The normalized quartet score (QT), which is the proportion of quartets in gene trees concordant with the species tree, is 0.9, meaning that concordance among gene trees is of 90% for the entire phylogeny. However, there are branches with low QT (<40%), indicating high gene tree discordance, coinciding with short branches (Figure 1B) and places of incongruent relationships with the SVDquartets tree (Figure 1A). Some of these branches are Byttnerioideae and some members of this subfamily that form a clade sister to Grewioideae; Malvatheca and its relationship with the rest of Malvadendrina; and branches within the subfamilies Dombeyoideae and Malvoideae.

We detected phylogenetic discordance by examining the low SVDquartets bootstrap values (Figure 1A) and the quartet score (QT) from the ASTRAL analysis (Figure 1B). Places with high discordance are the relationship between Helicterioideae and the four subfamilies Dombeyoideae, Tilioideae, Brownlowioideae, and Sterculioideae (77 BS; Figure 1); and Helicterioideae and the rest of Malvadendrina (37.51 QT; Figure 1); the relationship between Brownlowioideae and Sterculioideae + Tilioideae (66 BS; Figure 1); and Brownlowioideae and Dombeyoideae (42.45 QT; Figure 1). Byttnerioideae appeared as monophyletic in the analysis with a concatenated matrix (Supplementary Figure S1), but paraphyletic in the rest of the analyses (SVDquartets and ASTRAL; Figure 1), as well as in the temporally calibrated trees.

### Phylogenetic Discordance Source

**Figure 2:**
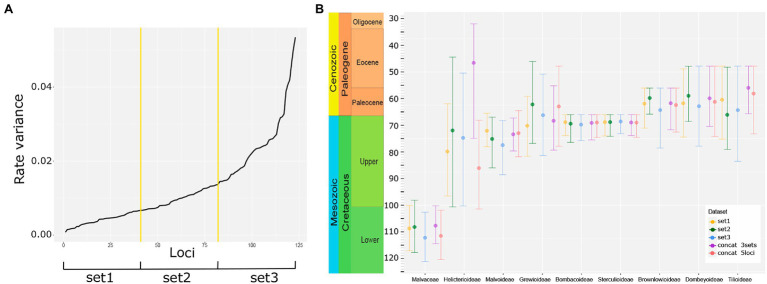
Summary of the sources of phylogenetic discordance obtained from QuIBL. **(A)** Species tree with the relationships among Malvaceae subfamilies derived from ASTRAL. Arrows indicate the direction of introgression or ILS events: black arrows represent relative strong introgression (>1% total non-ILS proportion), gray arrows represent relative weak introgression (<1% total non-ILS proportion), and orange arrow represents ILS. **(B)** Total proportion (%) of loci that show introgression between pairs of subfamilies or groups of subfamilies; relative strong introgression (>1%) shown in bold. See Supplementary Table S4 for detailed results.

We evaluated the proportion of ILS and introgression in the discordant gene trees with QuIBL (Edelman et al., 2019), a method that analyzes triplet topologies present in the gene trees. QuIBL extracts branch lengths in each triplet topology to test two models of branch length distribution: one model includes a distribution generated only by ILS, and the other includes two distributions, one for ILS only and another for introgression. Model selection was obtained with BIC values, selecting those values that were significantly different with dBIC <−10 or >10, as recommended by Edelman et al. (2019). We examined the discordance of the relationships among subfamilies by including two representatives of each subfamily and a sample of 108 gene trees (Supplementary Table S4), resulting in 816 triplets and 2,248 topologies. Table 1 summarizes QuIBL results that showed significant values (see Supplementary Table S4 for detailed, significant results). Significant results suggest that 62 discordant topologies are caused by introgression, and three are caused by ILS (Supplementary Table S4). We summarized QuIBL results considering that some topologies represent a single introgression event, for example, triplets that have different species but of the same subfamily have equal values, thus corresponding to a single introgression event that is ancestral to the divergence of the species included. This contrast with the results found between Byttnerioideae and Malvoideae, where two different genera yielded different proportions of introgression (Figure 2). We obtained high proportions of ILS across the phylogeny of Malvaceae (Figure 2; Table 1), but according with the preferred model, the discordance can only be explained jointly with introgression given that 0.9–3.7% of the loci are introgressed (Table 1). We identified 12 main events of introgression that involve all nine subfamilies, and one event of ILS alone (i.e., without introgression) in Helicterioideae-Malvatheca (Table 1; Figure 2).

**Table 1:** Source of phylogenetic discordance due to introgression and ILS between pairs of taxa.

Taxa pairs	Subfamily groups	ILS proportion	Non-ILS proportion	BIC2	BIC1	dBIC	Total non-ILS prop. (%)
*Guichenotia-Carpodiptera, Guichenotia-Brownlowia, Theobroma-Carpodiptera, Theobroma-Brownlowia*	Byttnerioideae-Brownlowioideae	0.00	1.00	−30.91	−18.65	−12.26	3.70
*Brownlowia-Pachira, Carpodiptera-Pachira, Corchoropsis-Pachira, Cheirolaena-Pachira, Mortoniodendron-Pachira*	Bombacoideae-Brownlowioideae+Dombeyoideae+Tilioideae	0.00	1.00	−33.99	−21.12	−12.87	2.78
*Guichenotia-Pentaplaris*	Byttnerioideae-Malvoideae	0.75	0.25	−45.91	−35.53	−10.37	1.85
*Heritiera-Pentaplaris, Brachychiton-Pentaplaris*	Sterculioideae-Malvoideae	0.50	0.50	−34.70	−22.46	−12.23	1.85
*Mortoniodendron-Pentaplaris*	Tilioideae-Malvoideae	0.50	0.50	−34.70	−22.46	−12.23	1.85
*Guichenotia-Corchoropsis, Guichenotia-Cheirolaena*	Byttnerioideae-Dombeyoideae	0.93	0.06	−79.60	−67.69	−11.91	0.93
*Guichenotia-Durio*	Byttnerioideae-*Durio*	0.93	0.06	−79.60	−67.69	−11.91	0.93
*Guichenotia-Heritiera, Guichenotia-Brachychiton*	Byttnerioideae-Sterculioideae	0.93	0.06	−79.60	−67.69	−11.91	0.93
*Mortoniodendron-Guichenotia, Guichenotia-Tilia*	Byttnerioideae-Tilioideae	0.93	0.06	−79.60	−67.69	−11.91	0.93
*Reevesia-Durio*	Helicterioideae-*Durio*	0.97	0.03	−211.37	−196.92	−14.45	0.93
*Cheirolaena-Tilia, Corchoropsis-Tilia*	Dombeyoideae-Tilioideae	0.97	0.03	−207.21	−194.06	−13.15	0.93
*Durio-Huberodendron Durio-Pachira*	Bombacoideae-*Durio*	0.97	0.03	−187.35	−174.36	−12.99	0.93
*Reevesia-Corchoropsis, Reevesia-Cheirolaena*	Helicterioideae-Dombeyoideae	0.96	0.04	−160.88	−149.11	−11.77	0.93
*Corchoropsis-Mortoniodendron, Mortoniodendron-Cheirolaena*	Dombeyoideae-Tilioideae	0.97	0.03	−228.43	−216.54	−11.89	0.93
*Mortoniodendron-Reevesia, Reevesia-Tilia*	Helicterioideae-Tilioideae	0.97	0.03	−176.27	−163.27	−13.00	0.93
*Reevesia-Heritiera, Reevesia-Brachychiton*	Helicterioideae-Sterculioideae	0.96	0.04	−166.77	−152.58	−14.19	0.93
*Carpodiptera-Reevesia, Reevesia-Brownlowia*	Helicterioideae-Brownlowioideae	0.97	0.03	−172.49	−160.95	−11.54	0.93
*Huberodendron-Glyphaea*	Grewioideae-Bombacoideae	0.87	0.13	−43.04	−30.94	−12.10	0.93
*Pentaplaris-Glyphaea, Duboscia-Pentaplaris*	Grewioideae-Malvoideae	0.50	0.50	−24.16	−3.71	−20.45	0.93
*Huberodendron-Abutilon*	Malvoideae-Bombacoideae	0.97	0.03	−187.35	−174.36	−12.99	0.93
*Theobroma-Pentaplaris*	Byttnerioideae-Malvoideae	0.50	0.50	−24.16	−5.16	−19.00	0.93
*Reevesia-Pachira*	Helicterioideae-Bombacoideae	0.72	0.28	−246.36	**−256.52**	10.16	0.10
*Reevesia-Huberodendron*	Helicterioideae-Bombacoideae	0.79	0.21	−204.46	**−214.47**	10.01	0.06
*Reevesia-Abutilon*	Helicterioideae-Malvoideae	0.84	0.16	−204.18	**−214.43**	10.25	0.05

Summary of the QuIBL results considering only significant (>10 dBIC) values. For simplicity, we refer to the included species by their genus name. ILS proportion reports the proportion of loci with ILS signal, whereas non-ILS proportion indicates the proportion of loci that show additionally an introgression pattern. Bayesian information criterion values are reported for BIC1 and BIC2, where BIC1 is the ILS-only model, while BIC2 model considers ILS and introgression. dBIC is the difference between BIC2 and BIC1 values and it was considered a measure of significance. Total non-ILS proportion is the number of introgressed loci between the two species in the taxa pairs. Bold numbers correspond to models where ILS was preferred over introgression. For detailed results, see Supplementary Table S4.

The proportion of introgressed loci is relatively high between the following pairs: Byttnerioideae-Brownlowioideae; Bombacoideae-(Dombeyoideae + Brownlowioideae + Tilioideae)-; Byttnerioideae-Malvoideae; and Malvoideae-(Sterculioideae + Tilioideae; Figure 2). Relatively low introgression is observed between Dombeyoideae-Tilioideae; Byttnerioideae-(Dombeyoideae + Sterculioideae + Tilioideae + *Durio*); Malvoideae-Bombacoideae; Bombacoideae-*Durio*; and Helicterioideae-*Durio* (Figure 2; Table 1). Given that the species tree (ASTRAL and SVDquartets) shows that *Durio* is separated from the rest of Helicterioideae, we describe the QuIBL results distinguishing Helicterioideae, with *Reevesia* as representative, from *Durio*. Introgression between Helicterioideae and the subfamilies Brownlowioideae, Dombeyoideae, Sterculioideae, and Tilioideae has the same magnitude, it is accompanied by a high proportion of ILS (96–97% of loci show ILS; Supplementary Table S4), and the tree counts are relatively similar among the three possible topologies, all of which indicate that ILS is highly frequent among these groups, but the signal is obscured due to a low but significant proportion of introgression. In turn, the trees with Helicterioideae as sister to Malvatheca are probably due to ILS only, and not introgression.

### Divergence Time Estimation

**Figure 3:**
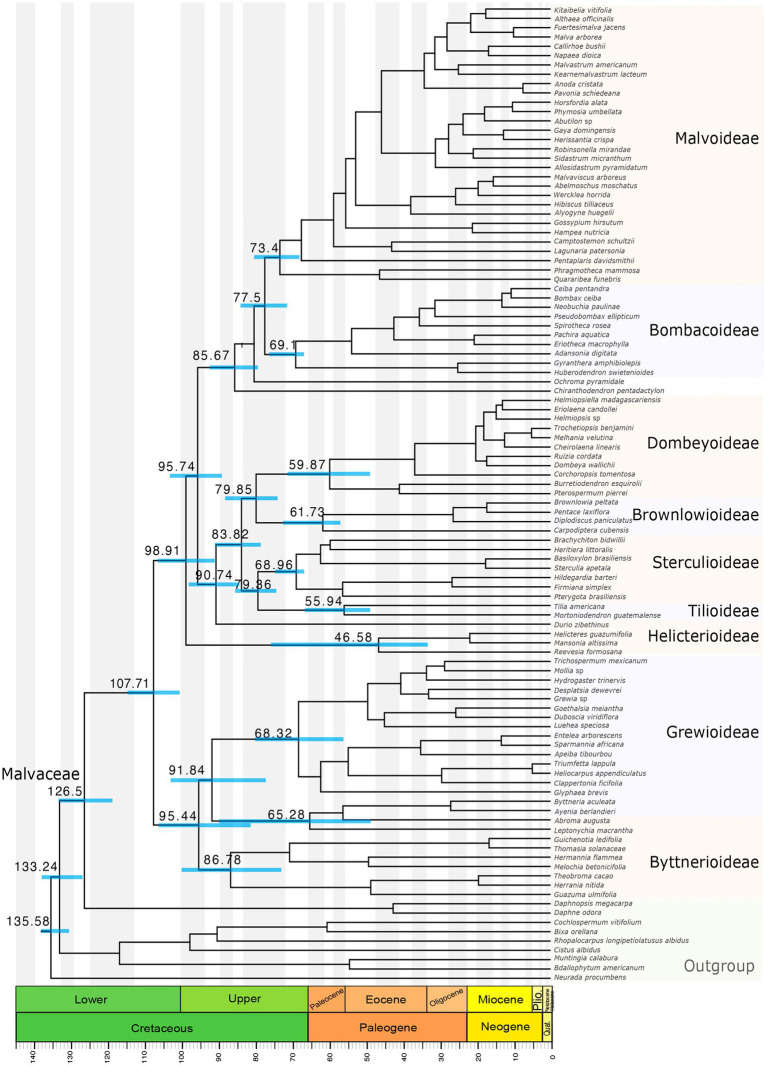
Molecular rate heterogeneity and age estimates. **(A)** Molecular rate variance for 123 loci, where the first tercile has lower variance (set1) and the third tercile has a higher variance (set3) as calculated with SortaDate. **(B)** Crown age estimates derived from five different datasets for Malvaceae and subfamilies, obtained with BEAST2. Circles show mean values, while the bars show 95% highest posterior density (HPD) intervals.

To know whether the heterogeneity of molecular substitution rate, characteristic of genomic data, affects the estimation of divergence times, we conducted five dating analysis. First, we followed a “gene shopping” approach to filter loci from the complete 268 loci sampling. Loci were selected first by the proportion of splits (bipartitions) according to the species tree from ASTRAL, and then by molecular rate variance, resulting in 123 loci. We found heterogeneity in the molecular rate variance (Figure 3A; Supplementary Table S5); thus, thresholds were applied to obtain three sets, each one including 41 different loci sharing relatively similar molecular rate variance (Figure 3A). We performed two additional analyses, one with the three concatenated sets (123 loci) and another analysis with five concatenated loci that had the lowest rate variances to test whether number of loci and lower rate heterogeneity are influencing age estimates.

We tested if the priors were constraining the estimates instead of being informed by the molecular datasets, found that the molecular datasets are informing the posterior density (Supplementary Figure S5). In general, age estimates for clades are similar among the five different sets and their 95% Highest Posterior Density (HPD) intervals overlap (Figure 3B; Supplementary Table S6; Supplementary File 2). This result is not maintained, however, when some phylogenetic relationships are different, for example, Helicterioideae is sister to Malvatheca or to the rest of Malvadendrina in the different analyses, so its age varies the most (Figure 3B; Supplementary Table S6). In general, we note that the set with the highest molecular rate variances (set3) yielded older ages (Figure 3B), but the difference between the sets with low and medium rate variance (set1 and set2, respectively) was not pronounced (Figure 3A). The length of the HPD intervals is similar among the five analyses when considering the major clades in Malvaceae, *ca.* 19.8–23.8 million years for crown age and *ca.* 16.7–21.1 million years for stem age.

**Figure 4:**
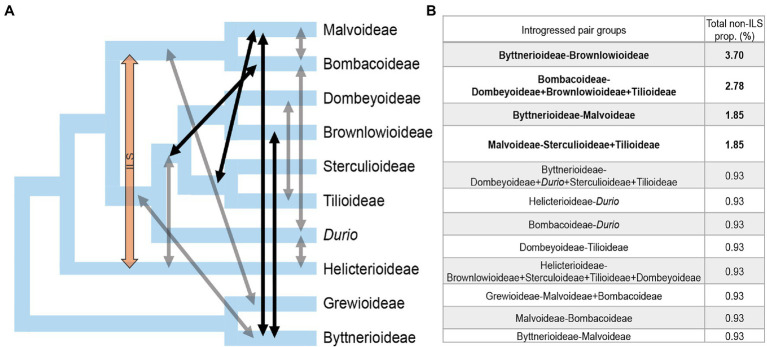
Maximum clade credibility tree derived from the concatenated dataset (123 loci) partitioned by set (set1, set2, and set3). Bars associated to age values are the 95% highest posterior density (HPD) intervals.

Considering that the five analyses yielded overlapping estimates and that the concatenated dataset of the three sets (concat_3sets) overall generated narrower 95% HPD intervals (Supplementary Table S6), that is, more precise estimates, we present the results of this dataset. Our results indicate an origin (stem age) of Malvaceae with a mean age of 126.5 Ma (Million years ago; 134–118 Ma 95% HPD; Figure 4), and a diversification age (crown age) with a mean of 107.71 Ma (114–100 Ma 95% HPD; Figure 4), both in the Lower Cretaceous. The nine subfamilies originated in the Upper Cretaceous, between 98.9 and 77.5 Ma (Supplementary Table S6), and diversified between the Upper Cretaceous and early Paleogene (74–59 Ma; Figure 4), except Helicterioideae and Tilioideae, which diversified in the early Eocene (56–47 Ma; Figure 4).

## Discussion

### Phylogenetic Relationships in Light of ILS and Introgression

Since the circumscription of Malvaceae s.l., the motivation for resolving its phylogenetic relationships has been to investigate intriguing aspects of the family’s evolution, such as its biogeographic distribution, paleontological evidence, or life history traits (Alverson et al., 1998, and references therein). More than 20 years later, the same motivation remains, and some key questions regarding Malvaceae evolution are still difficult to trace mostly due to conflicting phylogenetic results (e.g., Conover et al., 2019; Hernández-Gutiérrez et al., 2021). Consequently, in each independent study, where phylogenetic relationships are inferred *de novo,* new evolutionary hypotheses are formulated, instead of having a hypothesis that includes discordance sources in the evolution of Malvaceae and based on a consensus on the relationships within the family. Here, the aim of the research was to generate a phylogenetic hypothesis of Malvaceae that accounts for discordance and heterogeneity among nuclear loci, for which we evaluated the extent and potential sources of discordance, and examined its effect on estimating divergence times.

We sampled all nine subfamilies (40% of all genera) and analyzed the phylogenetic relationships with nuclear data; therefore, this is the first study showing inter- and intra-subfamilial relationships with nuclear sequences. We found high proportions of ILS (Table 1), which reduce accuracy in the “single-site” coalescence methods, such as SVDquartets (Chou et al., 2015). Thus, we will base our discussion on further discuss the results from ASTRAL (Figure 1B). Either by concatenating all loci or with coalescence, the concordant, highly supported (1 LPP) deep relationships include the two major clades of Malvaceae, Byttneriina, and Malvadendrina. Although there is low degree of introgression between Byttneriina and Malvadendrina members, as our analysis of discordance shows (Figure 2) and as was previously found in a reduced nuclear loci sample (Hernández-Gutiérrez et al., 2021), there is strong support of them being two, relatively old, and independent lineages. Other strongly supported relationships pertain to a clade conformed by four subfamilies, Sterculioideae, Tilioideae, and Brownlowioideae + Dombeyoideae (1 LPP); the Malvatheca clade; and within Malvatheca, *Chiranthodendron* as the sister to the remaining species of the clade, and *Ochroma* is subsequently sister to Bombacoideae + Malvoideae (Figure 1B).

Phylogenetic discordance among nuclear loci is evidence of the possible processes that took place in the history of Malvaceae. Hence, rather than a highly supported and completely resolved topology, here we aimed to obtain an estimate of the extent of phylogenetic discordance in the intricate history of reticulation and rapid diversification characteristic of the family (Conover et al., 2019). Through the coalescence methods, it was possible to locate specific points deep in the phylogeny where discordance is higher: among the four subfamilies Sterculioideae, Tilioideae, Dombeyoideae, and Brownlowioideae; the placement of Helicterioideae; and the Byttnerioideae groups (Figure 1). We discuss each of these three cases:

Three previous studies using plastomes have yielded conflicting, highly supported results. For example, in Conover et al. (2019), Dombeyoideae is sister to a clade formed by Sterculioideae + Tilioideae and Malvatheca; in Wang et al. (2020), Sterculioideae is sister only to Tilioideae + Dombeyoideae; and in Cvetković et al. (2021), Sterculioideae is sister to Tilioideae + Dombeyoideae, Brownlowioideae, and Malvatheca. With nuclear data, the study by Hernández-Gutiérrez et al. (2021) shows Sterculioideae as sister to Brownlowioideae, and, similarly to Conover et al. (2019), Dombeyoideae as sister to the remaining Malvadendrina subfamilies, albeit with low support. In the present study, we found these relationships: Sterculioideae + Tilioideae (0.83 LPP), Dombeyoideae + Brownlowioideae (0.99 LPP), these four subfamilies forming a highly supported clade (1 LPP). The low support of Sterculioideae as sister to Tilioideae, and the general conflict of these four subfamilies observed in the previous and the present study is explained by a strong signal of ILS present between each of these four subfamilies and other subfamilies, for example, Malvoideae and Byttnerioideae (Table 1) and combined with a relatively little proportion of reticulation with a member of Malvatheca experienced early in their diversification (Figure 2; Table 1).

In most of the phylogenetic analyses, we retrieved Helicterioideae (excluding *Durio*) as the sister group of the remaining Malvadendrina (0.61 LPP), which is congruent with plastome phylogenetic analyses (Conover et al., 2019; Cvetković et al., 2021; Wang et al., 2021). Our results yield a significantly preferred model of ILS when Helicterioideae is associated to Malvatheca (Table 1); thus, the discordant placement of Helicterioideae is probably caused by ILS only (Figure 2). In all our analyses, *Durio zibethinus* appears outside Helicterioideae and is sister to the clade comprising Sterculioideae, Tilioideae, Dombeyoideae, and Brownlowioideae. This placement is possibly derived from reticulation with members of other subfamilies given that phylogenetic discordance analyses show a high degree of introgression between *Durio* and Bombacoideae and *Durio* and Byttnerioideae (Table 1). The former introgression event had been previously inferred (Conover et al., 2019), all of which might be causing that nuclear information leads to such phylogenetic results. This needs to be explicitly examined with a denser sampling of species from the genus *Durio* and tribe Durioneae.

In this study, Byttnerioideae appeared as paraphyletic (Figure 1), except in the concatenated analysis where the subfamily was monophyletic (72 BS; Supplementary Figure S1). However, previous analyses with few plastid molecular markers, but a well-represented taxon sampling, showed that Byttnerioideae was strongly to moderately supported as a monophyletic group (Whitlock et al., 2001; Richardson et al., 2015; Hernández-Gutiérrez and Magallón, 2019). A group including *Byttneria* and *Leptonichia* (and other species of tribe Byttneriae) are separated from the rest of Byttnerioideae and are more closely related to Grewioideae (Figure 1), although with low support (0.27 LPP) and deriving from less than 30 QT (Figure 1B). Additional to ILS, the source of discordance in this case seems to derive from a low proportion of introgression between *Guichenotia* and the common ancestor of Sterculioideae, Tilioideae, Dombeyoideae, and Brownlowioideae (Figure 2; Table 1) and between *Theobroma*/*Guichenotia* and Malvoideae. This is an area for further research, as plastome analyses have included maximum two genera of this group (Conover et al., 2019; Cvetković et al., 2021; Wang et al., 2021).

Overall, our results indicate that ILS is the main source of phylogenetic discordance in the relationships among subfamilies, but only if combined with different degrees of introgression (Table 1). Thus, together these two processes explain the contentious relationships of the major lineages of Malvaceae (Figure 2). Analyzing whole-genome multiplications, Conover et al. (2019) formulated two alternative hypotheses. One considers an allopolyploidization event between dombeyoid and Malvatheca ancestors that gave rise to *Durio*. This hypothesis is somewhat consistent with our findings, but we detected a significant signal of introgression between *Durio* and Bombacoideae (not Malvoideae) and *Reevesia* (Helicterioideae). A potential allopolyploidization between the ancestors of Helicterioideae and Malvatheca/Bombacoideae may have caused the position of *Durio* apart from the rest of Helicterioideae found with our nuclear loci. The second hypothesis considers that Malvatheca originated *via* allopolyploidization between Sterculioideae + Tilioideae and Helicterioideae. This scenario is supported by a consistent introgression signal between Malvoideae and Sterculioideae + Tilioideae, but introgression was also detected between the four subfamilies Sterculioideae + Tilioideae + Dombeyoideae + Brownlowioideae and Bombacoideae. Therefore, it is possible that the observed signal in the genomes comes from a reticulation event involving the ancestors of the four subfamilies and the ancestor of Malvatheca. Moreover, considering Byttnerioideae and Grewioideae adds to the formulated hypotheses by Conover et al. (2019) a more complicated component, which is introgression between Malvadendrina and Byttneriina members obscured by a generalized ILS (Figure 2). How these past events shaped the morphological evolution of Malvaceae is now an interesting question to address, since it has been proved that floral traits acquired by introgression in baobabs might have led to adaptive evolution (Karimi et al., 2020).

A potential limitation of our analyses on the source of phylogenetic discordance relies on the assumption that gene trees are correctly estimated, because they were used to estimate the ASTRAL species tree that subsequently was used to compare the discordant topologies when interpreting QuIBL results. One particular aspect of gene tree inference concerns the collapsing of low supported bipartitions, where it has been identified that different collapsing methods have severe impacts on tree reconstruction (Simmons and Gatesy, 2021). Furthermore, as we discuss further in the next section, molecular rate heterogeneity has a strong impact on phylogenetic inferences in general, with new evidence on its impact on species tree estimation (Vankan et al., 2021). In the present study, we considered the heterogeneity in rates for divergence times, but not for the species tree estimation.

### Molecular Rate Heterogeneity and Discordance: Implications for Molecular Dating

Rate heterogeneity in Malvaceae was previously quantified within Malvatheca (Baum et al., 2004) and among the genomes of cotton, durian, and cacao (Wang et al., 2019a), where shifts in molecular evolutionary rate were many unit fold between Malvoideae and Bombacoideae (Baum et al., 2004) and between cotton and either durian or cacao (Wang et al., 2019a). It was thus expected to find high heterogeneity in our nuclear loci sampling (Figure 3A). Molecular rate heterogeneity is a long-recognized factor influencing both phylogenetic inference and divergence time estimation (Yang, 1995; Sanderson, 1997; Thorne et al., 1998), an influence that is exacerbated using hundreds of loci (Smith et al., 2018; Dornburg et al., 2019). Particularly important is the selection of loci and the assumptions on molecular clock models (Carruthers et al., 2020). Here, we used a “gene-shopping” approach to categorize loci by their rate, and then form three sets, of low, moderate, and high heterogeneity, each with 41 concatenated loci (Figure 3A). We also concatenated all loci into a single alignment partitioned by set. A final alignment was considered using the five loci with the lowest rate variance, which represents a conservative analysis given its homogeneity in molecular rate. Divergence time estimates show similar results among the five analyses, but the few observed substantial differences are probably due to phylogenetic discordances. Moreover, older ages were obtained from the third tercile of rate variance (set3 in Figures 3A,B), demonstrating that, although close and overlapping results, rate variance influences the general pattern of divergence times.

We found congruent age estimates possibly due to the number of calibrations we used, as it is known that when the heterogeneity in rate estimates is large multiple calibrations may constrain the estimates (Ho and Phillips, 2009). Divergence times here obtained are older than in Hernández-Gutiérrez and Magallón (2019), except for Tilioideae, which is younger. The difference might be related to numerous factors, such as molecular rate, taxon sampling, and phylogenetic relationships, but possibly mostly because the secondary calibration here applied to the Malvales, which was derived from the Ramírez-Barahona et al. (2020) study, is older than the one applied in the previous analysis. Wang et al. (2021) performed a divergence time estimation of Malvaceae and its subfamilies showing younger ages, probably due to the young secondary calibration, which was based on an analysis with a secondary calibration and Pure birth (Yule, 1924) tree diversification model (Richardson et al., 2015). Surprisingly, the here estimated crown age of Malvaceae roughly coincides with that estimated in Cvetković et al. (2021), but subfamilial ages in the present study are older possibly due to the larger taxon sampling.

In this study, we found that nuclear loci are highly variable in molecular rate and in phylogenetic histories, translated in high heterogeneity, and phylogenetic discordance, in particular, during the early diversification of the subfamilies. However, we were able to detect that ILS and different extents of introgression underlie this discordance and that rate heterogeneity slightly affects divergence time estimation due possibly to the combined information from the calibration priors. We also found that Helicterioideae and Byttnerioideae need to be further sampled and analyzed in the context to the remaining Malvadendrina groups and the relationships within them.

## Data Availability Statement

The data presented in the study are deposited in the NCBI Sequence Read Archive repository in the BioProject accession PRJNA815625.

## Author Contributions

RH-G, CB, CGM, MPC, EFM, and SM: field collection. RH-G, CB, CGM, and EL: laboratory procedure. AL: development and application of bioinformatic pipelines for raw data processing. RH-G: data analyses and visualization of results and writing of the first draft. All authors reviewed and edited the draft and agreed to the submitted version of the manuscript.

## Funding

We gratefully acknowledge funding provided by Programa de Apoyos a Proyectos de Investigación e Innovación Tecnológica of the Universidad Nacional Autónoma de México (UNAM) PAPIIT IG200316 and Fronteras de la Ciencia, Consejo Nacional de Ciencia y Tecnólogía (CONACyT) project number 2016-01-1867, both granted to SM. RH-G received a doctoral scholarship from CONACyT (407103/288658) and received the Elizabeth E. Bascom Fellowship for Latin American Women from the Missouri Botanical Garden, the American Society of Plant Taxonomists (ASPT) Graduate Research Grant, and the International Association for Plant Taxonomy (IAPT) Research Grant.

## Conflict of Interest

The authors declare that the research was conducted in the absence of any commercial or financial relationships that could be construed as a potential conflict of interest.

## Publisher’s Note

All claims expressed in this article are solely those of the authors and do not necessarily represent those of their affiliated organizations, or those of the publisher, the editors and the reviewers. Any product that may be evaluated in this article, or claim that may be made by its manufacturer, is not guaranteed or endorsed by the publisher.

## Footnotes

^1^ www.anchoredphylogeny.com

^2^ https://github.com/fmichonneau/chopper

^4^ github.com/rambaut/figtree
