# Supplementary material for: Localized Phylogenetic Discordance Among Nuclear Loci Due to Incomplete Lineage Sorting and Introgression in the Family of Cotton and Cacao (Malvaceae)
Source: Front Plant Sci. 2022 Apr 13;13:850521. doi: 10.3389/fpls.2022.850521 (PMC9043901; doi:10.3389/fpls.2022.850521)
Supplement: Supplementary file 2 [file Data_Sheet_2.DOCX]

#NEXUS

[Hernández-Gutiérrez et al. Localized Phylogenetic Discordance among Nuclear Loci due to ILS and Introgression in The Family of Cotton and Cacao (Malvaceae)]

[This file contains five chronograms corresponding to different data sets: TREE1=set1, TREE2=set2, TREE3=set3, TREE4=concat_3sets, TREE5=concat_5loci]

Begin taxa;

Dimensions ntax=105;

Taxlabels

Bombax ceiba

Herissantia_crispa

Malva_arborea

Rhopalocarpus_longipetiolatus

Cistus_albidus

Althaea_officinalis

Lagunaria_patersonia

Alyogyne_huegelii

Daphne_odora

Napaea_dioica

Grewia_sp

Callirhoe_bushii

Firmiana_simplex

Kitaibelia_vitifolia

Sterculia_apetala

Abelmoschus_moschatus

Dombeya_wallichii

Chiranthodendron_pentadactylon

Quararibea_funebris

Hampea_nutricia

Daphnopsis_megacarpa

Heliocarpus_appendiculatus

Ceiba_pentandra

Hibiscus_tilliaceus

Bixa_orellana

Cochlospermum_vitifolium

Pseudobombax_ellipticum

Ochroma_pyramidale

Malvaviscus_arboreus

Pavonia_schiedeana

Anoda_cristata

Phymosia_umbellata

Ayenia_berlandieri

Kearnemalvastrum_lacteum

Malvastrum_americanum

Fuertesimalva_jacens

Allosidastrum_pyramidatum

Melochia_betonicifolia

Sidastrum_micranthum

Gaya_domingensis

Neobuchia_paulinae

Bdallophytum_americanum

Carpodiptera_cubensis

Herrania_nitida

Guazuma_ulmifolia

Ruizia_cordata

Melhania_velutina

Glyphaea_brevis

Desplatsia_dewevrei

Mortoniodendron_guatemalense

Sparmannia_africana

Mollia_sp

Gossypium_hirsutum

Entelea_arborescens

Abutilon_sp

Reevesia_formosana

Mansonia_altissima

Horsfordia_alata

Pentaplaris_davidsmithii

Heritiera_littoralis

Durio_zibethinus

Brachychiton_bidwillii

Eriotheca_macrophylla

Hydrogaster_trinervis

Pterygota_brasiliensis

Leptonychia_macrantha

Neurada_procumbens

Pentace_laxiflora

Phragmotheca_mammosa

Camptostemon_schultzii

Trichospermum_mexicanum

Abroma_augusta

Corchoropsis_tomentosa

Apeiba_tibourbou

Helicteres_guazumifolia

Wercklea_horrida

Spirotheca_rosea

Huberodendron_swietenioides

Trochetiopsis_benjamini

Eriolaena_candollei

Adansonia_digitata

Byttneria_aculeata

Pterospermum_pierrei

Duboscia_viridiflora

Basiloxylon_brasiliensis

Gyranthera_amphibiolepis

Diplodiscus_paniculatus

Brownlowia_peltata

Burretiodendron_esquirolii

Hermannia_flammea

Thomasia_solanaceae

Guichenotia_ledifolia

Helmiopsis_sp

Cheirolaena_linearis

Helmiopsiella_madagascariensis

Goethalsia_meiantha

Clappertonia_ficifolia

Hildegardia_barteri

Theobroma_cacao

Pachira_aquatica

Robinsonella_mirandae

Tilia_americana

Triumfetta_lappula

Muntingia_calabura

Luehea_speciosa

;

End;

Begin trees;

Translate

1 Bombax ceiba,

2 Herissantia_crispa,

3 Malva_arborea,

4 Rhopalocarpus_longipetiolatus,

5 Cistus_albidus,

6 Althaea_officinalis,

7 Lagunaria_patersonia,

8 Alyogyne_huegelii,

9 Daphne_odora,

10 Napaea_dioica,

11 Grewia_sp,

12 Callirhoe_bushii,

13 Firmiana_simplex,

14 Kitaibelia_vitifolia,

15 Sterculia_apetala,

16 Abelmoschus_moschatus,

17 Dombeya_wallichii,

18 Chiranthodendron_pentadactylon,

19 Quararibea_funebris,

20 Hampea_nutricia,

21 Daphnopsis_megacarpa,

22 Heliocarpus_appendiculatus,

23 Ceiba_pentandra,

24 Hibiscus_tilliaceus,

25 Bixa_orellana,

26 Cochlospermum_vitifolium,

27 Pseudobombax_ellipticum,

28 Ochroma_pyramidale,

29 Malvaviscus_arboreus,

30 Pavonia_schiedeana,

31 Anoda_cristata,

32 Phymosia_umbellata,

33 Ayenia_berlandieri,

34 Kearnemalvastrum_lacteum,

35 Malvastrum_americanum,

36 Fuertesimalva_jacens,

37 Allosidastrum_pyramidatum,

38 Melochia_betonicifolia,

39 Sidastrum_micranthum,

40 Gaya_domingensis,

41 Neobuchia_paulinae,

42 Bdallophytum_americanum,

43 Carpodiptera_cubensis,

44 Herrania_nitida,

45 Guazuma_ulmifolia,

46 Ruizia_cordata,

47 Melhania_velutina,

48 Glyphaea_brevis,

49 Desplatsia_dewevrei,

50 Mortoniodendron_guatemalense,

51 Sparmannia_africana,

52 Mollia_sp,

53 Gossypium_hirsutum,

54 Entelea_arborescens,

55 Abutilon_sp,

56 Reevesia_formosana,

57 Mansonia_altissima,

58 Horsfordia_alata,

59 Pentaplaris_davidsmithii,

60 Heritiera_littoralis,

61 Durio_zibethinus,

62 Brachychiton_bidwillii,

63 Eriotheca_macrophylla,

64 Hydrogaster_trinervis,

65 Pterygota_brasiliensis,

66 Leptonychia_macrantha,

67 Neurada_procumbens,

68 Pentace_laxiflora,

69 Phragmotheca_mammosa,

70 Camptostemon_schultzii,

71 Trichospermum_mexicanum,

72 Abroma_augusta,

73 Corchoropsis_tomentosa,

74 Apeiba_tibourbou,

75 Helicteres_guazumifolia,

76 Wercklea_horrida,

77 Spirotheca_rosea,

78 Huberodendron_swietenioides,

79 Trochetiopsis_benjamini,

80 Eriolaena_candollei,

81 Adansonia_digitata,

82 Byttneria_aculeata,

83 Pterospermum_pierrei,

84 Duboscia_viridiflora,

85 Basiloxylon_brasiliensis,

86 Gyranthera_amphibiolepis,

87 Diplodiscus_paniculatus,

88 Brownlowia_peltata,

89 Burretiodendron_esquirolii,

90 Hermannia_flammea,

91 Thomasia_solanaceae,

92 Guichenotia_ledifolia,

93 Helmiopsis_sp,

94 Cheirolaena_linearis,

95 Helmiopsiella_madagascariensis,

96 Goethalsia_meiantha,

97 Clappertonia_ficifolia,

98 Hildegardia_barteri,

99 Theobroma_cacao,

100 Pachira_aquatica,

101 Robinsonella_mirandae,

102 Tilia_americana,

103 Triumfetta_lappula,

104 Muntingia_calabura,

105 Luehea_speciosa

;

tree TREE1 = (((((((((((((((((1[&height=2.210040760287013E-14,height_95%_HPD={0.0,5.6843418860808015E-14},height_median=2.8421709430404007E-14,height_range={0.0,8.526512829121202E-14},length=11.870410378854281,length_95%_HPD={5.099065677601146,19.033219067386014},length_median=11.591822184384178,length_range={3.9725590511718565,25.35311854777251},rate=0.001316633046300474,rate_95%_HPD={5.93446523669192E-4,0.0023128763091894767},rate_median=0.001204391700951821,rate_range={5.075475808093844E-4,0.0036570648746158552}]:11.870410378854283,23[&height=2.210040760287013E-14,height_95%_HPD={0.0,5.6843418860808015E-14},height_median=2.8421709430404007E-14,height_range={0.0,8.526512829121202E-14},length=11.870410378854281,length_95%_HPD={5.099065677601146,19.033219067386014},length_median=11.591822184384178,length_range={3.9725590511718565,25.35311854777251},rate=0.0016306876607606218,rate_95%_HPD={7.388023906739699E-4,0.0028734080853226296},rate_median=0.0014945957333914982,rate_range={6.662934971820484E-4,0.004688357195689938}]:11.870410378854283)[&height=11.870410378854304,height_95%_HPD={5.099065677601146,19.033219067386014},height_median=11.591822184384192,height_range={3.972559051171885,25.35311854777254},length=2.1620508656353983,length_95%_HPD={0.27300980161078314,5.090969049379041},length_median=1.7645669866714329,length_range={0.1606967536962287,11.332043394594606},posterior=1.0,rate=0.0011342734811303694,rate_95%_HPD={1.8524856966306623E-4,0.002513236060946858},rate_median=9.604823320366369E-4,rate_range={1.7524052119933666E-4,0.007648816813670984}]:2.162050865635365,41[&height=2.220836691498082E-14,height_95%_HPD={0.0,5.6843418860808015E-14},height_median=2.8421709430404007E-14,height_range={0.0,8.526512829121202E-14},length=14.032461244489642,length_95%_HPD={7.04747646478117,21.46350302215224},length_median=13.81867153300827,length_range={5.993023519148267,26.318525141414852},rate=0.0013795127609789448,rate_95%_HPD={7.334265568853397E-4,0.002253091211206755},rate_median=0.001296864746410829,rate_range={6.181106194390212E-4,0.0034245858460242146}]:14.032461244489646)[&height=14.032461244489669,height_95%_HPD={7.0474764647812265,21.463503022152267},height_median=13.818671533008285,height_range={5.993023519148267,26.31852514141488},length=12.79628824254693,length_95%_HPD={3.2503939903823635,22.15321310840352},length_median=12.328233704645974,length_range={2.1665586778394044,35.689803076730385},posterior=1.0,rate=0.0017381146440286449,rate_95%_HPD={5.720421875933131E-4,0.0036764920575144146},rate_median=0.0015002729415154949,rate_range={4.864659576398279E-4,0.00802414449571696}]:12.796288242546906,27[&height=2.2148705189867017E-14,height_95%_HPD={0.0,5.6843418860808015E-14},height_median=2.8421709430404007E-14,height_range={0.0,8.526512829121202E-14},length=26.828749487036546,length_95%_HPD={17.72494986669635,35.48019940516524},length_median=26.477394649267893,length_range={15.543730243175958,44.36309376605466},rate=0.0012858718908702208,rate_95%_HPD={8.72633780172084E-4,0.0017687585773753082},rate_median=0.001261995243920596,rate_range={6.90867463867282E-4,0.002164668989826638}]:26.828749487036553)[&height=26.828749487036575,height_95%_HPD={17.72494986669635,35.480199405165266},height_median=26.47739464926792,height_range={15.543730243175986,44.36309376605469},length=4.067148308131178,length_95%_HPD={0.7711771704750987,8.923939326460186},length_median=3.5128044374437764,length_range={0.4707492635759678,16.697765044996245},posterior=1.0,rate=0.0011400757677896742,rate_95%_HPD={2.382348230182035E-4,0.0024334069750980886},rate_median=9.747671783800776E-4,rate_range={2.2900888637269025E-4,0.005288553126097593}]:4.0671483081312125,77[&height=2.2285074847269996E-14,height_95%_HPD={0.0,2.8421709430404007E-14},height_median=2.8421709430404007E-14,height_range={0.0,8.526512829121202E-14},length=30.89589779516777,length_95%_HPD={22.110940891443306,39.476670343341254},length_median=30.789177613236014,length_range={17.980563373060008,47.99079670862184},rate=0.0014473277849534568,rate_95%_HPD={0.0010484773102872075,0.0018994627613399084},rate_median=0.0014266825763110127,rate_range={8.908140504825468E-4,0.002550272238498}]:30.895897795167766)[&height=30.895897795167787,height_95%_HPD={22.110940891443335,39.47667034334128},height_median=30.789177613236028,height_range={17.980563373060065,47.99079670862187},length=5.364355385772829,length_95%_HPD={1.025315089602799,11.838369562881326},length_median=4.534061298162236,length_range={0.6974335954644459,24.18823944050547},posterior=1.0,rate=0.001120292859500714,rate_95%_HPD={1.9959165352263002E-4,0.0024237817875061777},rate_median=9.494111560492842E-4,rate_range={1.9105652040374667E-4,0.005317222406590522}]:5.364355385772772,(63[&height=2.2046427946814784E-14,height_95%_HPD={0.0,2.8421709430404007E-14},height_median=2.8421709430404007E-14,height_range={0.0,8.526512829121202E-14},length=14.45562659010985,length_95%_HPD={4.579041668683118,24.84844318570974},length_median=13.638455834001796,length_range={4.076771219363252,35.32629129640645},rate=9.439438252687069E-4,rate_95%_HPD={3.7317509995367354E-4,0.0016911043975947058},rate_median=8.640297575594265E-4,rate_range={3.328167737994597E-4,0.0032000042049315455}]:14.455626590109858,100[&height=2.2046427946814784E-14,height_95%_HPD={0.0,2.8421709430404007E-14},height_median=2.8421709430404007E-14,height_range={0.0,8.526512829121202E-14},length=14.45562659010985,length_95%_HPD={4.579041668683118,24.84844318570974},length_median=13.638455834001796,length_range={4.076771219363252,35.32629129640645},rate=0.0012728402190944454,rate_95%_HPD={4.967367511876337E-4,0.002255903398521872},rate_median=0.0011637829122169822,rate_range={4.489993512040012E-4,0.004094832545239208}]:14.455626590109858)[&height=14.45562659010988,height_95%_HPD={4.579041668683118,24.84844318570977},height_median=13.638455834001824,height_range={4.076771219363309,35.32629129640645},length=21.804626590830686,length_95%_HPD={9.548169357823042,35.84044236232366},length_median=21.298138489527133,length_range={6.471846414187027,56.968795076198475},posterior=1.0,rate=9.217538420015272E-4,rate_95%_HPD={4.000828633366104E-4,0.0016188581229120195},rate_median=8.464286442259918E-4,rate_range={2.902230144932132E-4,0.002599763789179339}]:21.80462659083068)[&height=36.26025318094056,height_95%_HPD={26.658747587965408,46.5464715916728},height_median=36.0629228034897,height_range={23.648485021855947,62.4533463799027},length=11.591711781438159,length_95%_HPD={2.121246243312328,22.9456307758702},length_median=10.739055798501603,length_range={1.8575675273263954,34.77715486170267},posterior=1.0,rate=9.740427101330993E-4,rate_95%_HPD={2.588589156305307E-4,0.0020955827253125127},rate_median=8.108815827097444E-4,rate_range={2.3588619467083033E-4,0.0048200626945092435}]:11.591711781438235,81[&height=2.236746484861763E-14,height_95%_HPD={0.0,2.8421709430404007E-14},height_median=2.8421709430404007E-14,height_range={0.0,5.6843418860808015E-14},length=47.85196496237876,length_95%_HPD={36.45806126979522,59.2999153380205},length_median=47.80977082402896,length_range={29.92362128590213,67.62237852115719},rate=0.001086947098312849,rate_95%_HPD={8.328091104863492E-4,0.001368329121337175},rate_median=0.0010689912568485958,rate_range={7.371103541284413E-4,0.0017062211569051436}]:47.85196496237877)[&height=47.851964962378794,height_95%_HPD={36.45806126979525,59.299915338020526},height_median=47.80977082402897,height_range={29.923621285902144,67.62237852115722},length=21.000499964000447,length_95%_HPD={10.297134803943763,34.614539335566505},length_median=20.504774079316086,length_range={3.6000410964721254,42.63929243943964},posterior=1.0,rate=7.653951077009237E-4,rate_95%_HPD={3.782308607102361E-4,0.0012285211292494308},rate_median=7.072631634796685E-4,rate_range={3.300733583394155E-4,0.0038706116087858147}]:21.000499964000518,(78[&height=2.2390193124851456E-14,height_95%_HPD={0.0,2.8421709430404007E-14},height_median=2.8421709430404007E-14,height_range={0.0,5.6843418860808015E-14},length=34.65142181768941,length_95%_HPD={12.483385337449079,58.37391844481111},length_median=35.15787096431247,length_range={4.973948655317116,65.23085236560534},rate=0.0010265641736121438,rate_95%_HPD={4.405189706894597E-4,0.002021373313767823},rate_median=8.211359801029937E-4,rate_range={4.405189706894597E-4,0.005663645200443432}]:34.65142181768942,86[&height=2.2390193124851456E-14,height_95%_HPD={0.0,2.8421709430404007E-14},height_median=2.8421709430404007E-14,height_range={0.0,5.6843418860808015E-14},length=34.65142181768941,length_95%_HPD={12.483385337449079,58.37391844481111},length_median=35.15787096431247,length_range={4.973948655317116,65.23085236560534},rate=7.920592398165363E-4,rate_95%_HPD={3.3213201341797535E-4,0.0015779959475983691},rate_median=6.355913837443291E-4,rate_range={3.3213201341797535E-4,0.004417541882923683}]:34.65142181768942)[&height=34.65142181768944,height_95%_HPD={12.483385337449107,58.37391844481114},height_median=35.1578709643125,height_range={4.973948655317116,65.23085236560537},length=34.2010431086897,length_95%_HPD={10.16428097958945,55.02299137673968},length_median=33.4249140980891,length_range={7.737605526126259,70.70108974786866},posterior=1.0,rate=5.854098308840424E-4,rate_95%_HPD={2.354766294724483E-4,0.0012185286046555522},rate_median=5.040637645430954E-4,rate_range={2.2192164321027842E-4,0.0021420839515228006}]:34.20104310868987)[&height=68.85246492637931,height_95%_HPD={66.00035418397002,73.8340439409743},height_median=68.17689162728175,height_range={66.00035418397002,80.74892821032957},length=7.354038000901635,length_95%_HPD={2.3258954968189016,12.92580161623492},length_median=7.048871447024531,length_range={1.5188415068746508,20.352060487884273},posterior=1.0,rate=0.001972441240448393,rate_95%_HPD={7.589835715191226E-4,0.0039920061752244905},rate_median=0.0017122601517576396,rate_range={6.348026677682634E-4,0.008292061522069911}]:7.3540380009016815,((((((((((2[&height=2.2614634852660526E-14,height_95%_HPD={0.0,5.6843418860808015E-14},height_median=2.8421709430404007E-14,height_range={0.0,8.526512829121202E-14},length=13.871250312783,length_95%_HPD={7.079520353508741,21.121624998529143},length_median=13.63260448822819,length_range={4.916831806205181,29.98846253317778},rate=0.0016081376112965032,rate_95%_HPD={8.694285119237135E-4,0.002634510434058991},rate_median=0.0015200352057430835,rate_range={7.328096515934601E-4,0.0042788880612310305}]:13.871250312782996,40[&height=2.2614634852660526E-14,height_95%_HPD={0.0,5.6843418860808015E-14},height_median=2.8421709430404007E-14,height_range={0.0,8.526512829121202E-14},length=13.871250312783,length_95%_HPD={7.079520353508741,21.121624998529143},length_median=13.63260448822819,length_range={4.916831806205181,29.98846253317778},rate=0.0017629328005264275,rate_95%_HPD={9.198053453966855E-4,0.002826851474904204},rate_median=0.0016590504662036877,rate_range={7.139948900061874E-4,0.004664063197399677}]:13.871250312782996)[&height=13.871250312783019,height_95%_HPD={7.079520353508769,21.121624998529143},height_median=13.632604488228203,height_range={4.916831806205209,29.98846253317781},length=9.13579721318251,length_95%_HPD={2.9367210333683005,15.56957867576044},length_median=8.865304305401153,length_range={1.7451018149872226,21.656698179789146},posterior=1.0,rate=0.0016191062644841019,rate_95%_HPD={6.680760020813368E-4,0.003072277501042639},rate_median=0.001435480547402192,rate_range={5.908026773349324E-4,0.007075881169779965}]:9.135797213182546,((32[&height=2.26714555432451E-14,height_95%_HPD={0.0,5.6843418860808015E-14},height_median=2.8421709430404007E-14,height_range={0.0,8.526512829121202E-14},length=9.621403458958678,length_95%_HPD={4.7991565656760145,14.54969641702371},length_median=9.411696188102859,length_range={3.3675687187880783,21.519928210483982},rate=0.0020660927181609615,rate_95%_HPD={0.001073411630040528,0.0033196672621041315},rate_median=0.0019500571045741415,rate_range={8.145495653452318E-4,0.006014351853268525}]:9.621403458958678,58[&height=2.26714555432451E-14,height_95%_HPD={0.0,5.6843418860808015E-14},height_median=2.8421709430404007E-14,height_range={0.0,8.526512829121202E-14},length=9.621403458958678,length_95%_HPD={4.7991565656760145,14.54969641702371},length_median=9.411696188102859,length_range={3.3675687187880783,21.519928210483982},rate=0.0013358119083469095,rate_95%_HPD={6.969261253493165E-4,0.002139759816578066},rate_median=0.0012631046874409,rate_range={5.297731572330819E-4,0.003368624545506048}]:9.621403458958678)[&height=9.621403458958701,height_95%_HPD={4.799156565676043,14.549696417023767},height_median=9.411696188102887,height_range={3.3675687187881067,21.51992821048404},length=5.92181838879159,length_95%_HPD={1.3014235725767662,11.137101986658337},length_median=5.5162233771497355,length_range={0.5678893939701339,18.529966325232664},posterior=1.0,rate=0.0015328660301337379,rate_95%_HPD={4.3722001733337594E-4,0.0031539959110088943},rate_median=0.0013171027647594176,rate_range={3.644520825720518E-4,0.009092265184663453}]:5.92181838879158,55[&height=2.286748692576188E-14,height_95%_HPD={0.0,5.6843418860808015E-14},height_median=2.8421709430404007E-14,height_range={0.0,8.526512829121202E-14},length=15.543221847750258,length_95%_HPD={9.894495991884256,20.819769379708717},length_median=15.45419882310933,length_range={7.349913461143743,26.252336724242014},rate=0.0016403895708141604,rate_95%_HPD={0.001071090330128017,0.002303013580407237},rate_median=0.0015951117527954377,rate_range={9.10972612380101E-4,0.0032435582744462887}]:15.543221847750258)[&height=15.543221847750281,height_95%_HPD={9.894495991884313,20.819769379708745},height_median=15.454198823109358,height_range={7.349913461143771,26.25233672424207},length=7.463825678215249,length_95%_HPD={2.1366106942335534,13.30506615604024},length_median=7.198601409807544,length_range={1.0989519381309236,18.900052715226792},posterior=1.0,rate=0.0018743734692040367,rate_95%_HPD={6.485201431965461E-4,0.003795862701115231},rate_median=0.0016078605563205701,rate_range={5.966728031594184E-4,0.010307741003097913}]:7.463825678215283)[&height=23.007047525965564,height_95%_HPD={16.683832859622385,30.647180794014588},height_median=22.87653437348264,height_range={13.387564592887003,35.52201640341775},length=4.801867057317267,length_95%_HPD={1.3097759964578728,9.536794404979872},length_median=4.437486991115435,length_range={0.7058621822012441,14.65651475538067},posterior=1.0,rate=0.0015922521382596467,rate_95%_HPD={4.967820487784955E-4,0.003340118731992928},rate_median=0.0013874224544217248,rate_range={3.619556862501897E-4,0.007853529033234248}]:4.80186705731721,(39[&height=2.2393034159380687E-14,height_95%_HPD={0.0,5.6843418860808015E-14},height_median=2.8421709430404007E-14,height_range={0.0,5.6843418860808015E-14},length=22.63386943363888,length_95%_HPD={12.56968449967188,31.951556978424975},length_median=22.83806234660912,length_range={8.288464351743102,39.40733321967451},rate=0.0015353357059868537,rate_95%_HPD={9.535018977698736E-4,0.002432004426563156},rate_median=0.001441471548703395,rate_range={7.808106561000158E-4,0.004053602643261992}]:22.633869433638875,101[&height=2.2393034159380687E-14,height_95%_HPD={0.0,5.6843418860808015E-14},height_median=2.8421709430404007E-14,height_range={0.0,5.6843418860808015E-14},length=22.63386943363888,length_95%_HPD={12.56968449967188,31.951556978424975},length_median=22.83806234660912,length_range={8.288464351743102,39.40733321967451},rate=4.779277256303992E-4,rate_95%_HPD={2.863017337783111E-4,7.338150022096894E-4},rate_median=4.5099155625483926E-4,rate_range={2.3229254775673292E-4,0.001126651170380441}]:22.633869433638875)[&height=22.633869433638896,height_95%_HPD={12.569684499671908,31.951556978425003},height_median=22.83806234660915,height_range={8.288464351743102,39.40733321967454},length=5.1750451496438625,length_95%_HPD={0.36199558905764206,13.382727334000279},length_median=3.953378204455724,length_range={0.34169983470010834,25.095042238140763},posterior=1.0,rate=9.779199700148669E-4,rate_95%_HPD={1.5412654681566394E-4,0.002278352846160358},rate_median=7.779517267706935E-4,rate_range={1.5412654681566394E-4,0.008400896547416941}]:5.1750451496438785)[&height=27.808914583282775,height_95%_HPD={21.025867755841247,35.42525354437498},height_median=27.62158353073832,height_range={17.961083146123087,41.921661539893336},length=4.051643747879937,length_95%_HPD={0.7548529147483407,8.492825444219903},length_median=3.5348934450539957,length_range={0.3342743903850902,14.585879998658783},posterior=1.0,rate=0.0012905929259093998,rate_95%_HPD={2.6717826280571607E-4,0.0027955099695207326},rate_median=0.0011079088999523881,rate_range={2.2790852961704144E-4,0.0070485386703978935}]:4.051643747879979,37[&height=2.2643045197952814E-14,height_95%_HPD={0.0,5.6843418860808015E-14},height_median=2.8421709430404007E-14,height_range={0.0,5.6843418860808015E-14},length=31.860558331162725,length_95%_HPD={24.956526640659504,39.91763150290544},length_median=31.510806288608435,length_range={20.67722823834805,44.76483222494322},rate=0.0013339724183989653,rate_95%_HPD={0.001006174966739308,0.0016667181337578717},rate_median=0.0013254693510758094,rate_range={8.891033463739571E-4,0.00217463784036287}]:31.860558331162732)[&height=31.860558331162753,height_95%_HPD={24.956526640659504,39.917631502905465},height_median=31.510806288608478,height_range={20.67722823834808,44.76483222494322},length=13.312797622825219,length_95%_HPD={4.220695610354056,21.716671129880268},length_median=12.986478131017364,length_range={3.6137051465378107,31.432906372711273},posterior=1.0,rate=0.002959064326140967,rate_95%_HPD={0.0011485420764231046,0.006080057632363058},rate_median=0.002631992077828614,rate_range={0.0011485420764231046,0.009166355348655768}]:13.312797622825332,(((((3[&height=2.2469742091669863E-14,height_95%_HPD={0.0,5.6843418860808015E-14},height_median=2.8421709430404007E-14,height_range={0.0,8.526512829121202E-14},length=8.198433834820229,length_95%_HPD={3.403146872449696,13.400501463984867},length_median=7.922061045309043,length_range={2.3075000648584023,18.63228837452118},rate=0.001920781000158288,rate_95%_HPD={9.088616760196156E-4,0.0033531654251180852},rate_median=0.0017899813026575023,rate_range={7.264153107407314E-4,0.0059621867979544356}]:8.19843383482023,36[&height=2.2469742091669863E-14,height_95%_HPD={0.0,5.6843418860808015E-14},height_median=2.8421709430404007E-14,height_range={0.0,8.526512829121202E-14},length=8.198433834820229,length_95%_HPD={3.403146872449696,13.400501463984867},length_median=7.922061045309043,length_range={2.3075000648584023,18.63228837452118},rate=0.0018959127612935918,rate_95%_HPD={8.450661337534561E-4,0.003235872112982452},rate_median=0.0017607885479452364,rate_range={7.017876238896741E-4,0.0059621867979544356}]:8.19843383482023)[&height=8.198433834820253,height_95%_HPD={3.4031468724497245,13.400501463984895},height_median=7.922061045309043,height_range={2.3075000648584023,18.63228837452121},length=11.43146296388873,length_95%_HPD={4.547681680213103,18.249583846387793},length_median=10.956092398150794,length_range={3.885348013971253,27.185140697612184},posterior=1.0,rate=0.0031869627440401405,rate_95%_HPD={0.0014655771445823626,0.005229672405451603},rate_median=0.002997795380110439,rate_range={0.001177634154367706,0.008127780837851099}]:11.431462963888723,(6[&height=2.2356100710500714E-14,height_95%_HPD={0.0,5.6843418860808015E-14},height_median=2.8421709430404007E-14,height_range={0.0,8.526512829121202E-14},length=15.401280454348283,length_95%_HPD={7.61303046953671,22.773564633062975},length_median=15.111979415486417,length_range={6.080717162364408,29.910364333577334},rate=0.0017689545808049437,rate_95%_HPD={9.252676983888817E-4,0.002889155426003342},rate_median=0.00168647491762475,rate_range={7.769570843787924E-4,0.004159951523141143}]:15.401280454348285,14[&height=2.2356100710500714E-14,height_95%_HPD={0.0,5.6843418860808015E-14},height_median=2.8421709430404007E-14,height_range={0.0,8.526512829121202E-14},length=15.401280454348283,length_95%_HPD={7.61303046953671,22.773564633062975},length_median=15.111979415486417,length_range={6.080717162364408,29.910364333577334},rate=9.628550709832765E-4,rate_95%_HPD={5.023959622087151E-4,0.0015544947334366263},rate_median=9.175107074903823E-4,rate_range={3.9905290664899644E-4,0.002201277034436602}]:15.401280454348285)[&height=15.401280454348308,height_95%_HPD={7.61303046953671,22.77356463306303},height_median=15.111979415486417,height_range={6.0807171623644365,29.910364333577363},length=4.228616344360664,length_95%_HPD={0.5911237016478879,9.124990894195932},length_median=3.7359400450945586,length_range={0.2817774290663806,15.84962579661007},posterior=1.0,rate=0.0010805792372427067,rate_95%_HPD={2.2763839517914912E-4,0.0023613050239844778},rate_median=9.058342964418575E-4,rate_range={2.0754673209099063E-4,0.008593145611233644}]:4.228616344360669)[&height=19.629896798708977,height_95%_HPD={13.45100602837256,26.954679628808776},height_median=19.140860440844044,height_range={10.117788945071155,33.228147232713496},length=5.7856968229008805,length_95%_HPD={1.319736992019358,10.702355298938897},length_median=5.443516419859371,length_range={0.8781583568229223,22.015753773252882},posterior=1.0,rate=0.001845969298149512,rate_95%_HPD={5.196988490731733E-4,0.00393352102471332},rate_median=0.0015737806499513843,rate_range={3.254149080679311E-4,0.00893617568400765}]:5.7856968229008885,(10[&height=2.221120794951005E-14,height_95%_HPD={0.0,5.6843418860808015E-14},height_median=2.8421709430404007E-14,height_range={0.0,8.526512829121202E-14},length=12.41481485768041,length_95%_HPD={6.211081674206781,19.13616961951017},length_median=12.233449795429578,length_range={3.8143203581095975,24.500336495114965},rate=0.0021198359990660357,rate_95%_HPD={0.0010546450371531236,0.003474608694380801},rate_median=0.0019796554470333607,rate_range={8.421391578797746E-4,0.00637176031271029}]:12.414814857680415,12[&height=2.221120794951005E-14,height_95%_HPD={0.0,5.6843418860808015E-14},height_median=2.8421709430404007E-14,height_range={0.0,8.526512829121202E-14},length=12.41481485768041,length_95%_HPD={6.211081674206781,19.13616961951017},length_median=12.233449795429578,length_range={3.8143203581095975,24.500336495114965},rate=0.0018578228413195146,rate_95%_HPD={9.361122807269334E-4,0.003056144348677472},rate_median=0.0017396167811232644,rate_range={7.413880044645991E-4,0.00605514393518445}]:12.414814857680415)[&height=12.414814857680438,height_95%_HPD={6.21108167420681,19.1361696195102},height_median=12.233449795429593,height_range={3.814320358109626,24.500336495114965},length=13.000778763929349,length_95%_HPD={5.63268029537457,21.24672768360132},length_median=12.58035277809553,length_range={3.8377535215887377,32.287586753039264},posterior=1.0,rate=0.002606341754810311,rate_95%_HPD={0.001070227827923,0.004374426142832814},rate_median=0.0024279933188982947,rate_range={7.837896854253258E-4,0.007720845355597123}]:13.000778763929427)[&height=25.415593621609865,height_95%_HPD={18.42539305315958,32.236389681451286},height_median=25.24188154549953,height_range={15.806056357675189,38.40154557748343},length=3.3092896754142664,length_95%_HPD={0.6320008488015247,6.549206502256652},length_median=2.9659078462098165,length_range={0.31774205048452586,12.229684100934591},posterior=1.0,rate=0.0016336237921475735,rate_95%_HPD={3.953653640212277E-4,0.00333784900667853},rate_median=0.0014212146411626022,rate_range={3.5553832781523185E-4,0.008701535873133942}]:3.309615447606596,(34[&height=2.218847967327622E-14,height_95%_HPD={0.0,5.6843418860808015E-14},height_median=2.8421709430404007E-14,height_range={0.0,5.6843418860808015E-14},length=22.67937889414945,length_95%_HPD={14.15672887627079,32.85016998764631},length_median=22.60467731155886,length_range={7.208990450683217,40.228317217623186},rate=4.6877693862074565E-4,rate_95%_HPD={2.707701094492775E-4,6.937944418489974E-4},rate_median=4.48224264391693E-4,rate_range={2.2745757026384854E-4,0.0014626345644423947}]:22.679378894149455,35[&height=2.218847967327622E-14,height_95%_HPD={0.0,5.6843418860808015E-14},height_median=2.8421709430404007E-14,height_range={0.0,5.6843418860808015E-14},length=22.67937889414945,length_95%_HPD={14.15672887627079,32.85016998764631},length_median=22.60467731155886,length_range={7.208990450683217,40.228317217623186},rate=0.0016708874427501905,rate_95%_HPD={0.001032177653382449,0.0024441806195630534},rate_median=0.001591500057642876,rate_range={7.555318183133548E-4,0.004832152186854757}]:22.679378894149455)[&height=22.679378894149476,height_95%_HPD={14.156728876270847,32.85016998764631},height_median=22.604677311558873,height_range={7.208990450683245,40.228317217623186},length=6.046344064776007,length_95%_HPD={0.898943426298203,12.942721403689774},length_median=5.352133316286391,length_range={0.4906456473658949,25.089939545924125},posterior=1.0,rate=8.906553759585419E-4,rate_95%_HPD={1.911115027087996E-4,0.0021431625290655944},rate_median=7.001891109548865E-4,rate_range={1.6861115267445494E-4,0.005438676336382129}]:6.045830175066985)[&height=28.72520906921646,height_95%_HPD={22.11955418014324,35.84891643568287},height_median=28.62288219586705,height_range={18.60900261306972,42.37096685270154},length=2.465887519056986,length_95%_HPD={0.44628588044109563,5.173777118285244},length_median=2.1636727639881386,length_range={0.26676773162283496,12.886623025268364},posterior=0.9996001599360256,rate=0.0013218084587468858,rate_95%_HPD={2.8472687392093053E-4,0.0028133439890528104},rate_median=0.0011281427330383238,rate_range={2.3786571656903152E-4,0.007306657129068215}]:2.4654154481425543,(30[&height=2.212313587910396E-14,height_95%_HPD={0.0,5.6843418860808015E-14},height_median=2.8421709430404007E-14,height_range={0.0,8.526512829121202E-14},length=9.36160043619871,length_95%_HPD={2.3833148927688796,18.32199724400502},length_median=8.542739535535496,length_range={1.4445205721836203,29.287896557338257},rate=9.79763334710554E-4,rate_95%_HPD={2.6601382689955323E-4,0.0018918345240578947},rate_median=8.700250869543064E-4,rate_range={2.103668098299474E-4,0.004549179460421908}]:9.36160043619872,31[&height=2.212313587910396E-14,height_95%_HPD={0.0,5.6843418860808015E-14},height_median=2.8421709430404007E-14,height_range={0.0,8.526512829121202E-14},length=9.36160043619871,length_95%_HPD={2.3833148927688796,18.32199724400502},length_median=8.542739535535496,length_range={1.4445205721836203,29.287896557338257},rate=0.0011972112924666304,rate_95%_HPD={3.5623698233239735E-4,0.0023760920625927042},rate_median=0.0010523310218942578,rate_range={2.651982758628701E-4,0.005907531385607853}]:9.36160043619872)[&height=9.361600436198742,height_95%_HPD={2.383314892768908,18.321997244005047},height_median=8.542739535535524,height_range={1.4445205721836487,29.287896557338257},length=21.828184419258957,length_95%_HPD={10.211589790587254,32.20182059732025},length_median=21.918476998570966,length_range={4.301805529466904,39.05447231755673},posterior=1.0,rate=6.374381435948972E-4,rate_95%_HPD={3.378828770119985E-4,0.0010544830693988655},rate_median=5.915091506203654E-4,rate_range={2.8576110256426057E-4,0.002837722295317526}]:21.829024081160274)[&height=31.190624517359016,height_95%_HPD={23.704255699435933,37.699621371198646},height_median=31.072364285619685,height_range={20.972876951969,46.10702562925661},length=13.982731436628898,length_95%_HPD={5.750313572113754,23.209424603490163},length_median=13.516092928074322,length_range={3.2258196996432673,32.890190103558766},posterior=1.0,rate=0.0024402969357723613,rate_95%_HPD={0.0010545878739201481,0.004233815777064751},rate_median=0.002229284809539857,rate_range={8.902764654971925E-4,0.009166355348655768}]:13.98273143662907)[&height=45.173355953988086,height_95%_HPD={36.49150476400517,54.13575347365381},height_median=45.26297810982601,height_range={32.425183323526994,58.93007364849875},length=9.01489746746449,length_95%_HPD={2.751442102451932,15.321583811794397},length_median=8.66567093853719,length_range={2.1975527400875308,25.63843578603064},posterior=1.0,rate=0.0028265056881356986,rate_95%_HPD={9.788032604411395E-4,0.005216317926674747},rate_median=0.0025293932740167334,rate_range={8.565037847133605E-4,0.008936435422053874}]:8.836524818182362,(8[&height=2.2276551743682308E-14,height_95%_HPD={0.0,5.6843418860808015E-14},height_median=2.8421709430404007E-14,height_range={0.0,5.6843418860808015E-14},length=37.110614081442215,length_95%_HPD={25.445208466877617,48.92579713772216},length_median=37.19396931511143,length_range={17.127879322719522,54.805526650316196},rate=0.0015461917980004447,rate_95%_HPD={0.0010532192255244038,0.0021339054914524765},rate_median=0.0015020875123026457,rate_range={8.742349785115122E-4,0.0031979988736129173}]:37.11061408144223,(((16[&height=2.2509516575079062E-14,height_95%_HPD={0.0,5.6843418860808015E-14},height_median=2.8421709430404007E-14,height_range={0.0,5.6843418860808015E-14},length=16.2031025494302,length_95%_HPD={7.339832813252514,25.483563851274496},length_median=16.101497016882767,length_range={4.498045766047056,31.62060872799512},rate=0.002793222978834814,rate_95%_HPD={0.0014581950201975969,0.0047833795035728864},rate_median=0.002547734871512731,rate_range={0.0013178238972996388,0.00918271502173197}]:16.123971981345345,29[&height=2.2523721747725208E-14,height_95%_HPD={0.0,5.6843418860808015E-14},height_median=2.8421709430404007E-14,height_range={0.0,5.6843418860808015E-14},length=16.1429746358562,length_95%_HPD={7.238955764101547,25.3337381315001},length_median=16.015918930444776,length_range={4.498045766047056,31.62060872799512},rate=0.0019591828664512006,rate_95%_HPD={9.672847581824213E-4,0.0033292162882371207},rate_median=0.0017969366364622035,rate_range={8.84251830144292E-4,0.006336669264195219}]:16.123971981345345)[&height=16.123971981345367,height_95%_HPD={7.329683038782079,25.483563851274496},height_median=15.987285681819671,height_range={4.498045766047113,31.62060872799512},length=3.0596379755664747,length_95%_HPD={0.4049038220941412,6.991041707068135},length_median=2.603932359202723,length_range={0.27215815442689006,14.1489980525795},posterior=0.9676129548180727,rate=0.0012666327736174088,rate_95%_HPD={2.3620284123235843E-4,0.0027776119688950845},rate_median=0.001071781612612118,rate_range={2.0851605289642515E-4,0.008859489909446438}]:3.0489328641455273,76[&height=2.247258312619909E-14,height_95%_HPD={0.0,5.6843418860808015E-14},height_median=2.8421709430404007E-14,height_range={0.0,8.526512829121202E-14},length=19.094263024218495,length_95%_HPD={10.181310361747975,28.41403073961058},length_median=18.864457930662297,length_range={6.789688632175498,34.4787890090751},rate=0.0018879261380663882,rate_95%_HPD={0.0010639277153237005,0.0029942445671336413},rate_median=0.0017893771787194166,rate_range={9.463498058310273E-4,0.004927804701438154}]:19.172904845490873)[&height=19.172904845490894,height_95%_HPD={10.241164231195256,28.41403073961061},height_median=18.94791645528553,height_range={6.789688632175526,34.47878900907513},length=5.954644237384283,length_95%_HPD={1.3592596733377462,11.74317079617461},length_median=5.356009811138421,length_range={0.8409125368155799,22.554031831539703},posterior=1.0,rate=0.0015316533985551641,rate_95%_HPD={3.831748211926986E-4,0.0030133782270636827},rate_median=0.0013708853952048962,rate_range={3.0789867036017766E-4,0.007434264886714981}]:5.954644237384301,24[&height=2.2393034159380687E-14,height_95%_HPD={0.0,5.6843418860808015E-14},height_median=2.8421709430404007E-14,height_range={0.0,5.6843418860808015E-14},length=25.127549082875177,length_95%_HPD={15.198209864635587,35.66572149097861},length_median=24.987098399849906,length_range={9.608875198892775,42.44743726700037},rate=0.0016665804627858073,rate_95%_HPD={0.0010173331192981341,0.002444042927962671},rate_median=0.0015954812317459065,rate_range={9.248994496814123E-4,0.0041479859229978065}]:25.127549082875174)[&height=25.127549082875195,height_95%_HPD={15.198209864635587,35.66572149097861},height_median=24.98709839984992,height_range={9.608875198892775,42.44743726700037},length=11.983064998567073,length_95%_HPD={4.217828396902107,20.453102760408456},length_median=11.610352059470735,length_range={2.685977093447349,27.969775670355645},posterior=1.0,rate=0.0018990245577579314,rate_95%_HPD={7.66868988416086E-4,0.0035472577197821157},rate_median=0.0016882070181271076,rate_range={6.482094029729183E-4,0.0069862720519894665}]:11.983064998567055)[&height=37.11061408144225,height_95%_HPD={25.445208466877617,48.925797137722185},height_median=37.19396931511143,height_range={17.12787932271955,54.805526650316224},length=16.70604677968331,length_95%_HPD={6.419893545779047,27.758009074191634},length_median=16.171636341064534,length_range={4.367250238741519,39.222763319168834},posterior=1.0,rate=0.002766406353546778,rate_95%_HPD={0.001209992594787756,0.004974896593372653},rate_median=0.0025353828657793563,rate_range={0.0010096226759656517,0.008549497191527592}]:16.899266690728197)[&height=54.00988077217045,height_95%_HPD={46.09879222197458,60.735153417015084},height_median=54.14700240502819,height_range={43.02206536822021,66.63961777510377},length=2.2766168926154817,length_95%_HPD={0.3736920416444178,5.046834794505031},length_median=1.9496151247901565,length_range={0.1578310143557644,12.450410504859661},posterior=0.8990403838464615,rate=0.0014054168316511757,rate_95%_HPD={2.5943701078359646E-4,0.003001650051621368},rate_median=0.001192478370547453,rate_range={2.0088182480688363E-4,0.008866829487153604}]:2.2253903239509327,(20[&height=2.2466901057140632E-14,height_95%_HPD={0.0,2.8421709430404007E-14},height_median=2.8421709430404007E-14,height_range={0.0,5.6843418860808015E-14},length=21.684984749453804,length_95%_HPD={9.05050096315162,34.919989150914944},length_median=21.35898241124437,length_range={5.036829397486102,43.284569610692174},rate=0.0015971318166662677,rate_95%_HPD={6.98288800861418E-4,0.0029203656763434555},rate_median=0.0014289184079374722,rate_range={6.340804530252567E-4,0.005868427864483415}]:21.684984749453818,53[&height=2.2466901057140632E-14,height_95%_HPD={0.0,2.8421709430404007E-14},height_median=2.8421709430404007E-14,height_range={0.0,5.6843418860808015E-14},length=21.684984749453804,length_95%_HPD={9.05050096315162,34.919989150914944},length_median=21.35898241124437,length_range={5.036829397486102,43.284569610692174},rate=0.001165909271728539,rate_95%_HPD={5.45346735516793E-4,0.00216772861814678},rate_median=0.0010409949863365987,rate_range={4.4494150794927424E-4,0.004091711861296166}]:21.684984749453818)[&height=21.68498474945384,height_95%_HPD={9.05050096315162,34.919989150914944},height_median=21.35898241124439,height_range={5.036829397486159,43.2845696106922},length=34.181331012709244,length_95%_HPD={20.841131176507318,49.63284176287175},length_median=33.69576483805654,length_range={10.937952677665024,57.29546412513943},posterior=1.0,rate=0.0019493163989015422,rate_95%_HPD={0.0012086444572120868,0.002845093456768609},rate_median=0.001877477501766189,rate_range={0.0010415295954178628,0.005772437569552614}]:34.55028634666754)[&height=56.23527109612138,height_95%_HPD={49.099606959787764,63.59873599776823},height_median=56.41366446189557,height_range={45.500106113271016,68.90147552915614},length=2.373969348541611,length_95%_HPD={0.377832757693497,5.0809009975597235},length_median=2.063005069023035,length_range={0.1726248530971901,10.595698287145368},posterior=0.9992003198720512,rate=0.0014809113684977411,rate_95%_HPD={2.9830051068688724E-4,0.0032394037633179033},rate_median=0.001252837476590483,rate_range={2.121700084845736E-4,0.009852319459374807}]:2.3747081587978087,(7[&height=2.2489629333374462E-14,height_95%_HPD={0.0,2.8421709430404007E-14},height_median=2.8421709430404007E-14,height_range={0.0,5.6843418860808015E-14},length=45.84927601589183,length_95%_HPD={30.420629579645137,58.096655620779416},length_median=46.966880124151956,length_range={18.712514572728637,63.80050603405931},rate=0.0012722446068484854,rate_95%_HPD={9.126695499598437E-4,0.0017693345568226086},rate_median=0.0012051189220222047,rate_range={8.479950248796557E-4,0.0030286929911092267}]:45.84927601589183,70[&height=2.2489629333374462E-14,height_95%_HPD={0.0,2.8421709430404007E-14},height_median=2.8421709430404007E-14,height_range={0.0,5.6843418860808015E-14},length=45.84927601589183,length_95%_HPD={30.420629579645137,58.096655620779416},length_median=46.966880124151956,length_range={18.712514572728637,63.80050603405931},rate=9.691207424653558E-4,rate_95%_HPD={6.914949033865827E-4,0.0013592570452364262},rate_median=9.177335117560139E-4,rate_range={6.116860585744703E-4,0.002301957190044217}]:45.84927601589183)[&height=45.84927601589185,height_95%_HPD={30.420629579645166,58.096655620779416},height_median=46.966880124152,height_range={18.712514572728637,63.80050603405934},length=12.757818862998318,length_95%_HPD={1.7544237528196618,27.96991566125307},length_median=10.996968109019974,length_range={0.9788322311966908,43.08676820449807},posterior=1.0,rate=9.87337169197049E-4,rate_95%_HPD={1.8681972375399325E-4,0.0023172801167273167},rate_median=7.949901966235475E-4,rate_range={1.8681972375399325E-4,0.009203084673675047}]:12.760703239027336)[&height=58.60997925491919,height_95%_HPD={51.5795295038602,65.6135712017808},height_median=58.755145386636265,height_range={47.6689609539217,70.58494299481679},length=8.522533887827082,length_95%_HPD={3.490615066714014,14.83559553727585},length_median=8.152742995906877,length_range={2.887754352341318,20.254351423602657},posterior=1.0,rate=0.003442883129377127,rate_95%_HPD={0.0013239629581715431,0.006042714005678914},rate_median=0.0031763708028278125,rate_range={0.001190916912542316,0.008694380321736977}]:8.522533887827102,59[&height=2.2375987952205314E-14,height_95%_HPD={0.0,2.8421709430404007E-14},height_median=2.8421709430404007E-14,height_range={0.0,5.6843418860808015E-14},length=67.13251314274625,length_95%_HPD={60.339297332083106,73.86002663211715},length_median=67.16613242730223,length_range={56.332721694862045,79.45628714407563},rate=6.431751619929904E-4,rate_95%_HPD={5.650940118854894E-4,7.244833107575556E-4},rate_median=6.410108754556804E-4,rate_range={5.146280308419582E-4,8.103533181029233E-4}]:67.13251314274626)[&height=67.13251314274629,height_95%_HPD={60.339297332083106,73.86002663211718},height_median=67.16613242730226,height_range={56.33272169486207,79.45628714407566},length=4.93008241270191,length_95%_HPD={1.455950894288506,9.215385617742271},length_median=4.492108125382629,length_range={0.9516640892540806,16.24842504233125},posterior=1.0,rate=0.0019258455725558545,rate_95%_HPD={5.907078230423297E-4,0.0037935206570487573},rate_median=0.0017260993291890654,rate_range={4.269761404695607E-4,0.007246613875440123}]:4.930082412701736,(19[&height=2.2373146917676087E-14,height_95%_HPD={0.0,2.8421709430404007E-14},height_median=2.8421709430404007E-14,height_range={0.0,5.6843418860808015E-14},length=33.26071050453422,length_95%_HPD={11.389264083678967,56.66326511000753},length_median=31.923585139014314,length_range={8.942614707056592,68.07881798569545},rate=0.0010417542910886434,rate_95%_HPD={4.2718583971409574E-4,0.002021467552651265},rate_median=9.220365882006618E-4,rate_range={4.073111443668317E-4,0.003250882867432988}]:33.26071050453422,69[&height=2.2373146917676087E-14,height_95%_HPD={0.0,2.8421709430404007E-14},height_median=2.8421709430404007E-14,height_range={0.0,5.6843418860808015E-14},length=33.26071050453422,length_95%_HPD={11.389264083678967,56.66326511000753},length_median=31.923585139014314,length_range={8.942614707056592,68.07881798569545},rate=8.310355076479541E-4,rate_95%_HPD={3.461200700439821E-4,0.0016039811379229025},rate_median=7.325966888549851E-4,rate_range={3.069002377372952E-4,0.002630020515367784}]:33.26071050453422)[&height=33.26071050453424,height_95%_HPD={11.389264083678995,56.66326511000756},height_median=31.923585139014342,height_range={8.94261470705662,68.07881798569548},length=38.801885050914,length_95%_HPD={14.019644873129252,61.28697272228469},length_median=40.18193921708877,length_range={6.600213343666198,70.00304936392827},posterior=1.0,rate=5.111879162327269E-4,rate_95%_HPD={2.4338390892015386E-4,0.0010550954229044509},rate_median=4.1671001642887105E-4,rate_range={2.0874166391713695E-4,0.0024302718403248743}]:38.80188505091379)[&height=72.06259555544803,height_95%_HPD={65.49406867655169,78.06272824337773},height_median=72.12231773587337,height_range={61.34837434712536,86.17877439543702},length=4.1439073718325945,length_95%_HPD={0.9481075006859072,8.268260214126101},length_median=3.7127677034850564,length_range={0.5787400311280493,13.999196881669704},posterior=1.0,rate=0.001435431658294524,rate_95%_HPD={4.0059266801750185E-4,0.002829173789847892},rate_median=0.0012646943163785243,rate_range={3.1508579025907616E-4,0.007654613136206629}]:4.143907371832967)[&height=76.206502927281,height_95%_HPD={70.31843780825449,81.96241337320589},height_median=76.11986360292624,height_range={68.48886593573738,89.03797127635984},length=2.43565850245985,length_95%_HPD={0.4667324292209116,4.836918053516101},length_median=2.2067216555922613,length_range={0.2580710768757797,10.624939583415184},posterior=1.0,rate=0.00152146512785043,rate_95%_HPD={4.4607270645700205E-4,0.003358281177403124},rate_median=0.0012974531727874266,rate_range={2.5346128976820766E-4,0.007320075862358632}]:2.4356585024596598,28[&height=2.2381670021263772E-14,height_95%_HPD={0.0,2.8421709430404007E-14},height_median=2.8421709430404007E-14,height_range={0.0,5.6843418860808015E-14},length=78.64216142974063,length_95%_HPD={72.09523357328193,84.62208761808319},length_median=78.58427694509899,length_range={69.97474684994587,91.77886513274818},rate=3.165332981634084E-4,rate_95%_HPD={2.741431653796728E-4,3.628013250096519E-4},rate_median=3.1579612913108736E-4,rate_range={2.3897866638433802E-4,4.20322712414729E-4}]:78.64216142974063)[&height=78.64216142974065,height_95%_HPD={72.09523357328196,84.62208761808321},height_median=78.58427694509902,height_range={69.9747468499459,91.77886513274821},length=5.271231372815161,length_95%_HPD={1.4232634363550716,9.920460582390845},length_median=4.880209331246689,length_range={0.9836897499192219,15.76351397506292},posterior=1.0,rate=0.0018369113701616267,rate_95%_HPD={6.227546986510738E-4,0.0035209817206252525},rate_median=0.0016319453144934806,rate_range={4.6152356763917875E-4,0.007450156228923615}]:5.271231372815265,18[&height=2.2381670021263772E-14,height_95%_HPD={0.0,2.8421709430404007E-14},height_median=2.8421709430404007E-14,height_range={0.0,5.6843418860808015E-14},length=83.91339280255589,length_95%_HPD={76.2958413499999,91.73386046909721},length_median=83.84061941274463,length_range={72.03129142704796,98.40155225827966},rate=3.939458802392322E-4,rate_95%_HPD={3.3737417936541037E-4,4.4670543531436034E-4},rate_median=3.9359574679121914E-4,rate_range={2.6644543154060216E-4,5.039453446136743E-4}]:83.91339280255589)[&height=83.91339280255592,height_95%_HPD={76.29584134999993,91.73386046909724},height_median=83.84061941274464,height_range={72.03129142704799,98.40155225827966},length=8.507290472161396,length_95%_HPD={3.031367651596028,15.063922029792977},length_median=8.004197667125219,length_range={1.9861088226066812,21.07119786801961},posterior=1.0,rate=0.002426477836493048,rate_95%_HPD={8.854709249151031E-4,0.004202297788342323},rate_median=0.0022673469129655833,rate_range={8.09253367751804E-4,0.009092265184663453}]:8.507290472161273,61[&height=2.2381670021263772E-14,height_95%_HPD={0.0,2.8421709430404007E-14},height_median=2.8421709430404007E-14,height_range={0.0,5.6843418860808015E-14},length=92.42068327471719,length_95%_HPD={84.91712073818867,101.40069785741369},length_median=92.00497220907431,length_range={78.89327565092906,106.95344666490733},rate=6.951362752526188E-4,rate_95%_HPD={6.181252913780001E-4,7.68839606466651E-4},rate_median=6.948868388581769E-4,rate_range={5.533417489413035E-4,8.427455912576869E-4}]:92.42068327471716)[&height=92.42068327471719,height_95%_HPD={84.9171207381887,101.40069785741369},height_median=92.00497220907434,height_range={78.89327565092908,106.95344666490733},length=4.330195771111051,length_95%_HPD={0.9067351772427372,8.691572962932014},length_median=3.927295294112305,length_range={0.6008307332071894,15.709237779534703},posterior=1.0,rate=0.0014199591735843799,rate_95%_HPD={3.5037680795519126E-4,0.0029300158546100258},rate_median=0.0012326980882456603,rate_range={3.353526648836244E-4,0.00741849867111339}]:4.330195771111221,(((((13[&height=2.236746484861763E-14,height_95%_HPD={0.0,2.8421709430404007E-14},height_median=2.8421709430404007E-14,height_range={0.0,5.6843418860808015E-14},length=28.22732753507105,length_95%_HPD={9.247984354430258,45.434295000404845},length_median=27.435264935841325,length_range={7.932001411907834,54.416717580401496},rate=9.227718207440039E-4,rate_95%_HPD={4.0399470855712104E-4,0.0018273234257608956},rate_median=8.295631482877201E-4,rate_range={3.678587895058406E-4,0.002868103872780467}]:28.227327535071048,98[&height=2.236746484861763E-14,height_95%_HPD={0.0,2.8421709430404007E-14},height_median=2.8421709430404007E-14,height_range={0.0,5.6843418860808015E-14},length=28.22732753507105,length_95%_HPD={9.247984354430258,45.434295000404845},length_median=27.435264935841325,length_range={7.932001411907834,54.416717580401496},rate=0.0012543395149791185,rate_95%_HPD={5.658293449989613E-4,0.0024955693261568632},rate_median=0.0011275882171996833,rate_range={5.305660337152283E-4,0.0037687160128043514}]:28.227327535071048)[&height=28.22732753507107,height_95%_HPD={9.247984354430258,45.43429500040487},height_median=27.43526493584134,height_range={7.932001411907834,54.416717580401524},length=30.997410024698016,length_95%_HPD={13.828276577675325,48.111945497545435},length_median=31.46915393322427,length_range={7.772345859396026,54.69197959509748},posterior=1.0,rate=8.1598045720442E-4,rate_95%_HPD={4.1918486706716846E-4,0.0014897650089460762},rate_median=7.195760169618089E-4,rate_range={3.86817575467732E-4,0.0026194008192868597}]:30.997410024697928,65[&height=2.2373146917676087E-14,height_95%_HPD={0.0,2.8421709430404007E-14},height_median=2.8421709430404007E-14,height_range={0.0,5.6843418860808015E-14},length=59.224737559768954,length_95%_HPD={44.86608137238217,70.15322121286671},length_median=60.551659565771274,length_range={30.1475186371037,75.45188552676956},rate=8.863849598434071E-4,rate_95%_HPD={7.093533413261357E-4,0.0011433599980175341},rate_median=8.581176583257826E-4,rate_range={6.314203512147295E-4,0.0016433895956505786}]:59.224737559768975)[&height=59.224737559769,height_95%_HPD={44.8660813723822,70.15322121286674},height_median=60.55165956577129,height_range={30.14751863710373,75.45188552676959},length=9.603681035702886,length_95%_HPD={1.7495961302871876,22.2953407513578},length_median=8.038531591772283,length_range={1.51699339222111,39.54527035684548},posterior=1.0,rate=9.048823709640755E-4,rate_95%_HPD={2.0269643421743234E-4,0.001966300988903561},rate_median=7.727026597476678E-4,rate_range={1.9120514273713104E-4,0.0038507328254592395}]:9.603681035703232,((15[&height=2.23447365723838E-14,height_95%_HPD={0.0,2.8421709430404007E-14},height_median=2.8421709430404007E-14,height_range={0.0,5.6843418860808015E-14},length=22.221982531516765,length_95%_HPD={6.1980460785154605,40.09452824260063},length_median=21.234670842493358,length_range={5.288027584538071,58.468545197093846},rate=0.0012716646551846757,rate_95%_HPD={4.1183794471825964E-4,0.0025375661024562117},rate_median=0.0011073600367650753,rate_range={3.860192698375031E-4,0.0042510856021567974}]:22.221982531516762,85[&height=2.23447365723838E-14,height_95%_HPD={0.0,2.8421709430404007E-14},height_median=2.8421709430404007E-14,height_range={0.0,5.6843418860808015E-14},length=22.221982531516765,length_95%_HPD={6.1980460785154605,40.09452824260063},length_median=21.234670842493358,length_range={5.288027584538071,58.468545197093846},rate=0.0010344685430207028,rate_95%_HPD={3.4478331189989576E-4,0.002073511895876827},rate_median=9.007987630391396E-4,rate_range={3.166649222095281E-4,0.003780326358177881}]:22.221982531516762)[&height=22.221982531516783,height_95%_HPD={6.1980460785154605,40.09452824260063},height_median=21.234670842493387,height_range={5.288027584538071,58.468545197093846},length=43.16498847757145,length_95%_HPD={21.9231373783298,59.72754679311531},length_median=44.2877753171745,length_range={12.145997858833354,67.18606220969473},posterior=1.0,rate=6.136204866051237E-4,rate_95%_HPD={3.6992220684614694E-4,9.991278365336052E-4},rate_median=5.612667062661848E-4,rate_range={3.4272080580614514E-4,0.001963314684742679}]:43.30561520319753,(60[&height=2.2378828986734544E-14,height_95%_HPD={0.0,2.8421709430404007E-14},height_median=2.8421709430404007E-14,height_range={0.0,8.526512829121202E-14},length=63.57456288420578,length_95%_HPD={55.61820589261562,71.60664538433471},length_median=63.5969683101816,length_range={48.140449984894076,78.02598076596652},rate=8.463377330007484E-4,rate_95%_HPD={7.348421052468472E-4,9.632931100172583E-4},rate_median=8.433737523662391E-4,rate_range={6.690539281887392E-4,0.0011089452647340865}]:63.399326412476036,62[&height=2.2378828986734544E-14,height_95%_HPD={0.0,2.8421709430404007E-14},height_median=2.8421709430404007E-14,height_range={0.0,8.526512829121202E-14},length=63.45983395692493,length_95%_HPD={55.70276207514004,71.56927531417496},length_median=63.513120475823186,length_range={48.140449984894076,78.02598076596652},rate=6.57118933225848E-4,rate_95%_HPD={5.70089207981478E-4,7.530871010112426E-4},rate_median=6.540990445685562E-4,rate_range={5.127822406249811E-4,8.984448839432574E-4}]:63.399326412476036)[&height=63.39932641247606,height_95%_HPD={55.706777518009275,71.50693720497085},height_median=63.45638590042186,height_range={48.140449984894104,78.02598076596655},length=2.1041435231097014,length_95%_HPD={0.21827592286049935,5.180264051428665},length_median=1.680107312833826,length_range={0.0776835775458693,13.538362571239915},posterior=0.92203118752499,rate=0.0010298358361529446,rate_95%_HPD={1.9222654080135486E-4,0.002377790915530943},rate_median=8.46762709218583E-4,rate_range={1.7465750228996347E-4,0.006791965724015633}]:2.1282713222382625)[&height=65.52759773471432,height_95%_HPD={58.16812889711326,73.103824443774},height_median=65.38887249236198,height_range={52.10336218198542,79.39959879823847},length=3.300820860757981,length_95%_HPD={0.30758543118506054,8.508593356805079},length_median=2.571591987151834,length_range={0.20286807038367272,20.2739521771325},posterior=1.0,rate=9.598952816570924E-4,rate_95%_HPD={1.6377072459996162E-4,0.0022584568201333647},rate_median=7.80286383262319E-4,rate_range={1.5679371790643708E-4,0.006489461134756407}]:3.3008208607579093)[&height=68.82841859547223,height_95%_HPD={66.00055423049501,74.03909269568446},height_median=68.12896004442233,height_range={66.00055423049501,83.60277879689096},length=13.640999915365311,length_95%_HPD={4.381783220711455,21.65665210780351},length_median=13.311160676608587,length_range={3.9671414366507634,29.24670828548915},posterior=1.0,rate=0.0024558392637119815,rate_95%_HPD={0.0010435832968114516,0.00443121079832468},rate_median=0.0022393095052118594,rate_range={9.716689376129623E-4,0.007181742996503038}]:13.640999915365157,(50[&height=2.2381670021263772E-14,height_95%_HPD={0.0,2.8421709430404007E-14},height_median=2.8421709430404007E-14,height_range={0.0,5.6843418860808015E-14},length=60.411948560567616,length_95%_HPD={47.82015632317194,75.21607809065797},length_median=59.89881280365401,length_range={47.80130162540573,87.85100657363628},rate=8.420378385677802E-4,rate_95%_HPD={6.397016467188385E-4,0.0010469257506122772},rate_median=8.37468764290226E-4,rate_range={5.63165130684664E-4,0.0011231344674045973}]:60.41194856056761,102[&height=2.2381670021263772E-14,height_95%_HPD={0.0,2.8421709430404007E-14},height_median=2.8421709430404007E-14,height_range={0.0,5.6843418860808015E-14},length=60.411948560567616,length_95%_HPD={47.82015632317194,75.21607809065797},length_median=59.89881280365401,length_range={47.80130162540573,87.85100657363628},rate=2.481892286976994E-4,rate_95%_HPD={1.8534563483508888E-4,3.153014559441892E-4},rate_median=2.4600702474217496E-4,rate_range={1.5738768658784578E-4,3.8296603946504613E-4}]:60.41194856056761)[&height=60.41194856056763,height_95%_HPD={47.82015632317197,75.21607809065797},height_median=59.89881280365404,height_range={47.801301625405756,87.85100657363631},length=22.057469950269702,length_95%_HPD={6.581098432634121,34.177217586427616},length_median=22.173953886156248,length_range={4.01429192755775,47.381715975131954},posterior=1.0,rate=6.232307233103832E-4,rate_95%_HPD={2.704024789014158E-4,0.0012344211211442308},rate_median=5.402659505470632E-4,rate_range={2.3358082510428088E-4,0.003062245608553832}]:22.057469950269756)[&height=82.46941851083739,height_95%_HPD={74.15594692877153,91.31884443016344},height_median=82.19101097613105,height_range={71.3738420070595,98.60426377315788},length=6.129061351321225,length_95%_HPD={1.4059255819103242,12.327727453428878},length_median=5.547585205236906,length_range={0.9399233156596978,22.232968623911702},posterior=1.0,rate=0.0011386241874618561,rate_95%_HPD={2.637272352540056E-4,0.0024082065704536105},rate_median=9.748402440300744E-4,rate_range={2.2585235095346642E-4,0.0051564167733555676}]:6.129061351321013,((((17[&height=2.2225413122156193E-14,height_95%_HPD={0.0,2.8421709430404007E-14},height_median=2.8421709430404007E-14,height_range={0.0,5.6843418860808015E-14},length=22.349310225513353,length_95%_HPD={14.864369484855786,31.522661537832008},length_median=21.64204979856678,length_range={11.801266852070313,40.61151442836203},rate=6.475751976585809E-4,rate_95%_HPD={3.975959308525601E-4,8.946841759575884E-4},rate_median=6.422093051210608E-4,rate_range={2.7469295492793755E-4,0.0013566422982263294}]:22.460314508677246,((46[&height=2.2350418641442257E-14,height_95%_HPD={0.0,5.6843418860808015E-14},height_median=2.8421709430404007E-14,height_range={0.0,8.526512829121202E-14},length=16.981762880818994,length_95%_HPD={10.65461690921029,25.954313478896538},length_median=16.212351019400785,length_range={8.858928315497991,34.789985729999074},rate=8.061319444427413E-4,rate_95%_HPD={4.625952016117323E-4,0.0011652243061460678},rate_median=7.99060697734423E-4,rate_range={3.5674943490599783E-4,0.0015575665919525098}]:16.98807885007626,((80[&height=2.216291036251316E-14,height_95%_HPD={0.0,5.6843418860808015E-14},height_median=2.8421709430404007E-14,height_range={0.0,8.526512829121202E-14},length=11.888918927176492,length_95%_HPD={5.443835945795485,19.480399230052626},length_median=11.15615736354021,length_range={4.453608579410627,29.110536237417733},rate=0.0014461891448533074,rate_95%_HPD={6.719291288063556E-4,0.002243876293249817},rate_median=0.0014076815989194258,rate_range={5.371652606132813E-4,0.00376089333183592}]:11.888918927176492,95[&height=2.216291036251316E-14,height_95%_HPD={0.0,5.6843418860808015E-14},height_median=2.8421709430404007E-14,height_range={0.0,8.526512829121202E-14},length=11.888918927176492,length_95%_HPD={5.443835945795485,19.480399230052626},length_median=11.15615736354021,length_range={4.453608579410627,29.110536237417733},rate=0.0010418545333819402,rate_95%_HPD={4.662408899513565E-4,0.0016091443521842116},rate_median=0.0010098721770164503,rate_range={3.5361416894316206E-4,0.002742258493859198}]:11.888918927176492)[&height=11.888918927176514,height_95%_HPD={5.443835945795513,19.480399230052655},height_median=11.156157363540238,height_range={4.453608579410627,29.110536237417733},length=2.81642541652165,length_95%_HPD={0.3792434377266858,6.370046211906526},length_median=2.3597321640517137,length_range={0.26918995293776504,15.086588125754119},posterior=1.0,rate=0.0010764139510427758,rate_95%_HPD={1.9287197468909458E-4,0.0024040450092771837},rate_median=9.086337549289716E-4,rate_range={1.7690621580241433E-4,0.006152858026958248}]:2.8098646361593644,93[&height=2.2285074847269996E-14,height_95%_HPD={0.0,5.6843418860808015E-14},height_median=2.8421709430404007E-14,height_range={0.0,8.526512829121202E-14},length=14.699028374440816,length_95%_HPD={8.877532339443889,23.734415949276425},length_median=13.89693318633109,length_range={5.918434571069852,31.235825432590502},rate=0.0012409767314616098,rate_95%_HPD={6.567990038863872E-4,0.0018251973101118782},rate_median=0.001228602251961278,rate_range={5.284601387334928E-4,0.0028632777819399113}]:14.698783563335855)[&height=14.698783563335878,height_95%_HPD={8.797252529714584,23.669503285434075},height_median=13.904994182606771,height_range={5.918434571069909,31.23582543259053},length=2.2905191576536263,length_95%_HPD={0.29146019931332035,5.522343574022386},length_median=1.8729293390933748,length_range={0.12989667164053742,11.338305200664976},posterior=0.9966013594562175,rate=0.0010238963188379045,rate_95%_HPD={1.6542764552951773E-4,0.002317748755112689},rate_median=8.511779541009682E-4,rate_range={1.6542764552951773E-4,0.006052983159780963}]:2.289295286740403)[&height=16.98807885007628,height_95%_HPD={10.663527483907174,25.954313478896566},height_median=16.21309091474027,height_range={8.85892831549802,34.7899857299991},length=3.5535384405571357,length_95%_HPD={0.6045619867691272,7.651203685706761},length_median=3.0905828432823554,length_range={0.4645905024686243,14.135912680933274},posterior=1.0,rate=9.9634600658522E-4,rate_95%_HPD={1.9920159433752134E-4,0.002233689540732453},rate_median=8.236163555117974E-4,rate_range={1.6530578376653196E-4,0.005080470210679909}]:3.4473189876337393,((47[&height=2.2390193124851456E-14,height_95%_HPD={0.0,5.6843418860808015E-14},height_median=2.8421709430404007E-14,height_range={0.0,8.526512829121202E-14},length=5.699181983398784,length_95%_HPD={2.1401351744083286,9.480112833677694},length_median=5.415531089042602,length_range={1.5624452545447127,15.057364545589436},rate=0.002813926108861734,rate_95%_HPD={0.0012211653138638728,0.0051115567434663785},rate_median=0.002591939683855093,rate_range={8.78797402842623E-4,0.009064516816619943}]:5.699181983398787,79[&height=2.2390193124851456E-14,height_95%_HPD={0.0,5.6843418860808015E-14},height_median=2.8421709430404007E-14,height_range={0.0,8.526512829121202E-14},length=5.699181983398784,length_95%_HPD={2.1401351744083286,9.480112833677694},length_median=5.415531089042602,length_range={1.5624452545447127,15.057364545589436},rate=8.814930415274555E-4,rate_95%_HPD={3.3248438254808357E-4,0.0015659570899035414},rate_median=8.12976617211758E-4,rate_range={2.7571750494931015E-4,0.0030265784837773513}]:5.699181983398787)[&height=5.699181983398809,height_95%_HPD={2.1401351744083854,9.480112833677723},height_median=5.415531089042602,height_range={1.5624452545447411,15.057364545589465},length=8.893184800616877,length_95%_HPD={2.187756730278039,15.547480490987525},length_median=8.360695346341167,length_range={2.0185463703976296,29.421513089775402},posterior=1.0,rate=0.0026626421041930854,rate_95%_HPD={7.798504711880693E-4,0.005035983328436526},rate_median=0.0024133446068881625,rate_range={6.573284544522569E-4,0.009590445636294034}]:8.893184800616883,94[&height=2.2151546224396248E-14,height_95%_HPD={0.0,5.6843418860808015E-14},height_median=2.8421709430404007E-14,height_range={0.0,8.526512829121202E-14},length=14.592366784015663,length_95%_HPD={8.321063148253046,22.765784891385692},length_median=14.020808205432559,length_range={6.698632046225413,32.05435950495078},rate=0.002189051480321061,rate_95%_HPD={0.0011975132555307022,0.003259811408562078},rate_median=0.0021400563665221655,rate_range={8.58304745295382E-4,0.004527825238571151}]:14.59236678401567)[&height=14.592366784015692,height_95%_HPD={8.321063148253046,22.76578489138572},height_median=14.020808205432587,height_range={6.698632046225441,32.05435950495084},length=5.854617363176407,length_95%_HPD={2.052066259329166,11.122457484776191},length_median=5.463474506993002,length_range={1.0184795606898973,16.532841410395676},posterior=1.0,rate=0.0019504687374810063,rate_95%_HPD={6.443529136584E-4,0.003491268606301689},rate_median=0.0017804147376479123,rate_range={5.339747550847315E-4,0.009064516816619943}]:5.843031053694329)[&height=20.43539783771002,height_95%_HPD={13.234903985190797,28.99649588396848},height_median=19.755280590494408,height_range={12.219497790984292,37.55517709499961},length=2.049845402018277,length_95%_HPD={0.19584467928297045,4.935376738762159},length_median=1.6774798152783532,length_range={0.11191041181402284,11.681278931991443},posterior=0.9320271891243502,rate=0.0011943929912676598,rate_95%_HPD={1.6998570091039975E-4,0.002703279509423731},rate_median=9.949047374313473E-4,rate_range={1.6903175217333115E-4,0.008999202474602905}]:2.0249166709672473)[&height=22.460314508677268,height_95%_HPD={15.27194178592083,31.945156315091225},height_median=21.725156707677797,height_range={12.900232231843887,40.61151442836206},length=19.768486623504987,length_95%_HPD={6.419839708431951,31.122025032612825},length_median=19.46545108117793,length_range={5.629449029122995,40.950102867306924},posterior=1.0,rate=0.00249052109657004,rate_95%_HPD={0.00108289167190391,0.004595058725832063},rate_median=0.0022310394297422047,rate_range={0.001002707042244369,0.0075815861937408024}]:19.768486623504952,73[&height=2.2415762435614517E-14,height_95%_HPD={0.0,2.8421709430404007E-14},height_median=2.8421709430404007E-14,height_range={0.0,5.6843418860808015E-14},length=42.22880113218221,length_95%_HPD={27.43738147458231,55.879860188596126},length_median=41.846388832456036,length_range={25.144312666427695,67.26352301858249},rate=0.0013768955948590086,rate_95%_HPD={9.293662003397511E-4,0.001923784175935032},rate_median=0.0013463662453618312,rate_range={8.069233016573797E-4,0.0022690747424846964}]:42.2288011321822)[&height=42.22880113218222,height_95%_HPD={27.437381474582338,55.879860188596155},height_median=41.84638883245605,height_range={25.144312666427723,67.26352301858249},length=19.530943389199095,length_95%_HPD={6.837918346736657,30.58284198513124},length_median=19.375812539832175,length_range={5.568497537948858,38.81860260724882},posterior=1.0,rate=0.0022490882701733847,rate_95%_HPD={0.001027221820655199,0.004299438526758749},rate_median=0.0019970911497092054,rate_range={9.743093150935617E-4,0.007051618223685588}]:19.53094338919916,(83[&height=2.247542416072832E-14,height_95%_HPD={0.0,2.8421709430404007E-14},height_median=2.8421709430404007E-14,height_range={0.0,8.526512829121202E-14},length=49.801979777342225,length_95%_HPD={30.44392139319754,65.88198001817248},length_median=50.22548316676107,length_range={18.882254446854645,74.41425049403512},rate=0.001342397272157878,rate_95%_HPD={9.190461914706328E-4,0.001993195275145175},rate_median=0.0012830310826647082,rate_range={8.477627713943336E-4,0.003382151751861838}]:49.80197977734222,89[&height=2.247542416072832E-14,height_95%_HPD={0.0,2.8421709430404007E-14},height_median=2.8421709430404007E-14,height_range={0.0,8.526512829121202E-14},length=49.801979777342225,length_95%_HPD={30.44392139319754,65.88198001817248},length_median=50.22548316676107,length_range={18.882254446854645,74.41425049403512},rate=0.0014720137965303299,rate_95%_HPD={9.90955434788392E-4,0.0021628858056267123},rate_median=0.0014043822846692088,rate_range={8.63780263314432E-4,0.003719295026563995}]:49.80197977734222)[&height=49.80197977734224,height_95%_HPD={30.44392139319757,65.88198001817251},height_median=50.2254831667611,height_range={18.882254446854645,74.41425049403514},length=11.957764744039014,length_95%_HPD={1.3360915727423048,28.342794094659382},length_median=9.904557818524651,length_range={1.3039804433303601,44.514668498931556},posterior=1.0,rate=0.0010459812055862697,rate_95%_HPD={2.0723940122795574E-4,0.002472824065171946},rate_median=8.626591423170835E-4,rate_range={1.7646161878245715E-4,0.006194241370866017}]:11.957764744039139)[&height=61.75974452138138,height_95%_HPD={48.81886280160026,74.44451896998868},height_median=61.44853682989113,height_range={47.80966743696618,83.00268238361605},length=22.264638481730366,length_95%_HPD={9.105347952634432,35.390183587344296},length_median=22.11553013094487,length_range={5.933875037586361,43.329919462804355},posterior=1.0,rate=0.0021362056178290028,rate_95%_HPD={0.0010169450145883093,0.003918117771991843},rate_median=0.001914049701531894,rate_range={9.208114875961332E-4,0.007297937882229593}]:22.2646384817303,(43[&height=2.2384511055793E-14,height_95%_HPD={0.0,2.8421709430404007E-14},height_median=2.8421709430404007E-14,height_range={0.0,5.6843418860808015E-14},length=61.88766949969881,length_95%_HPD={56.00099781531068,71.08142581299548},length_median=60.824372535541315,length_range={56.00099781531068,83.56888815235222},rate=8.806988981263799E-4,rate_95%_HPD={7.430180859498615E-4,0.0010009675127466051},rate_median=8.88115383767143E-4,rate_range={6.447962656579497E-4,0.001054472715723875}]:61.887669499698795,((68[&height=2.2327690365208426E-14,height_95%_HPD={0.0,2.8421709430404007E-14},height_median=2.8421709430404007E-14,height_range={0.0,8.526512829121202E-14},length=18.7602092735294,length_95%_HPD={8.894164704813079,31.216149319395143},length_median=17.97938227385118,length_range={7.208462719222894,41.37181249859492},rate=0.0014628449429252605,rate_95%_HPD={6.402724507222487E-4,0.0023703945187998165},rate_median=0.0013829959159149252,rate_range={5.503717894784849E-4,0.0033664306429350586}]:18.760209273529412,88[&height=2.2327690365208426E-14,height_95%_HPD={0.0,2.8421709430404007E-14},height_median=2.8421709430404007E-14,height_range={0.0,8.526512829121202E-14},length=18.7602092735294,length_95%_HPD={8.894164704813079,31.216149319395143},length_median=17.97938227385118,length_range={7.208462719222894,41.37181249859492},rate=9.84340704512574E-4,rate_95%_HPD={4.6129254220514355E-4,0.0016298153367303313},rate_median=9.358636537069679E-4,rate_range={3.478566841135987E-4,0.0022481497152045285}]:18.760209273529412)[&height=18.760209273529433,height_95%_HPD={8.894164704813107,31.21614931939517},height_median=17.979382273851193,height_range={7.208462719222922,41.37181249859495},length=6.308039529548223,length_95%_HPD={0.9064936653717695,14.420438711469785},length_median=5.2925606391937166,length_range={0.624731097677298,30.107938172275468},posterior=1.0,rate=0.0011656476230338625,rate_95%_HPD={1.7005691515958762E-4,0.0027824341295977694},rate_median=9.566799889353108E-4,rate_range={1.7005691515958762E-4,0.007069522117686946}]:6.308039529548271,87[&height=2.2435649677319117E-14,height_95%_HPD={0.0,2.8421709430404007E-14},height_median=2.8421709430404007E-14,height_range={0.0,5.6843418860808015E-14},length=25.068248803077672,length_95%_HPD={13.369036063327542,37.59262386524978},length_median=24.336822641751162,length_range={10.609948268557346,47.549696540136566},rate=0.0013253566690202604,rate_95%_HPD={7.090353157554801E-4,0.002007172876933899},rate_median=0.0012792601505934344,rate_range={6.258877167200331E-4,0.0030990512618055722}]:25.068248803077683)[&height=25.068248803077704,height_95%_HPD={13.369036063327542,37.592623865249806},height_median=24.336822641751176,height_range={10.609948268557375,47.549696540136594},length=36.819420696621386,length_95%_HPD={20.779851756366185,52.60803934040321},length_median=37.01576056833207,length_range={13.56225974035361,60.15984879331988},posterior=1.0,rate=0.0011487182085402493,rate_95%_HPD={7.101821946024416E-4,0.0017770207817586778},rate_median=0.0010786889281731095,rate_range={6.195193271606979E-4,0.002985716450524767}]:36.81942069662111)[&height=61.887669499698816,height_95%_HPD={56.00099781531071,71.0814258129955},height_median=60.82437253554134,height_range={56.00099781531071,83.56888815235224},length=22.136713503412736,length_95%_HPD={12.177310685465045,33.84347534840879},length_median=21.790654077707195,length_range={7.494553680403243,41.15464006849602},posterior=1.0,rate=0.0020784443396729994,rate_95%_HPD={0.0011424882824766045,0.003165525471506469},rate_median=0.0019696529284759694,rate_range={0.001017644505366652,0.005699544404821813}]:22.136713503412864)[&height=84.02438300311168,height_95%_HPD={74.79437444451695,93.33966699696244},height_median=83.99773501206275,height_range={69.14969874833375,100.2571714238803},length=4.574096859046755,length_95%_HPD={0.5697400023603052,9.830498092614178},length_median=3.938477373838147,length_range={0.46822645836395793,19.268889373653835},posterior=1.0,rate=0.001334406594271223,rate_95%_HPD={2.5191322333237135E-4,0.0028853897169408187},rate_median=0.0011300989393079955,rate_range={1.9734516625187308E-4,0.008249224123908145}]:4.574096859046719)[&height=88.5984798621584,height_95%_HPD={80.23480750525295,97.25957151681834},height_median=88.37772834976862,height_range={75.35999725986636,103.35511666939163},length=8.152399183669786,length_95%_HPD={2.7379467277038145,15.02027286125734},length_median=7.73983227844338,length_range={1.5313516199753678,22.23355939895069},posterior=1.0,rate=0.0016174450671993456,rate_95%_HPD={5.505298120165914E-4,0.003042731280139028},rate_median=0.0014281111125753533,rate_range={4.562764878646585E-4,0.007352568568136939}]:8.152399183670013)[&height=96.75087904582841,height_95%_HPD={88.79671851801247,105.35063433920342},height_median=96.48106493347902,height_range={83.78891031849938,110.17725397141815},length=4.017616775992967,length_95%_HPD={0.9698559463096075,8.016587650886692},length_median=3.679890455192904,length_range={0.6330949593648825,14.643612046852084},posterior=1.0,rate=0.001580428888999706,rate_95%_HPD={4.76883831380012E-4,0.0031945290449983295},rate_median=0.0013717317683985212,rate_range={3.670500514607902E-4,0.006897031162201289}]:4.017616775992622,(56[&height=2.2378828986734544E-14,height_95%_HPD={0.0,2.8421709430404007E-14},height_median=2.8421709430404007E-14,height_range={0.0,5.6843418860808015E-14},length=79.85403685468988,length_95%_HPD={61.91256207912731,96.53580643775119},length_median=80.00307833235995,length_range={54.445640401874044,107.94507944673336},rate=6.704914020451252E-4,rate_95%_HPD={5.265318401789986E-4,8.37443790494519E-4},rate_median=6.61415431896094E-4,rate_range={4.5966891407704885E-4,0.0010004489480613297}]:79.8540368546899,(57[&height=2.2356100710500714E-14,height_95%_HPD={0.0,2.8421709430404007E-14},height_median=2.8421709430404007E-14,height_range={0.0,5.6843418860808015E-14},length=44.06685882072839,length_95%_HPD={24.25758335768751,66.70858065604355},length_median=44.79582410160754,length_range={10.397298826050658,72.41708175601717},rate=0.001497853695831491,rate_95%_HPD={8.718561689962281E-4,0.002390638472926219},rate_median=0.0013595799598193387,rate_range={8.069749642163679E-4,0.005818588114868398}]:44.0668588207284,75[&height=2.2356100710500714E-14,height_95%_HPD={0.0,2.8421709430404007E-14},height_median=2.8421709430404007E-14,height_range={0.0,5.6843418860808015E-14},length=44.06685882072839,length_95%_HPD={24.25758335768751,66.70858065604355},length_median=44.79582410160754,length_range={10.397298826050658,72.41708175601717},rate=0.0016765146480806346,rate_95%_HPD={9.724471803358401E-4,0.0026864802437138514},rate_median=0.0015254283573232549,rate_range={9.282417732545078E-4,0.0066427602628925655}]:44.0668588207284)[&height=44.06685882072842,height_95%_HPD={24.25758335768751,66.70858065604358},height_median=44.79582410160756,height_range={10.397298826050687,72.4170817560172},length=35.78717803396143,length_95%_HPD={10.646093220159315,59.04216620654583},length_median=35.140325581889726,length_range={8.23547951737369,72.88005944085694},posterior=1.0,rate=0.001577271057801141,rate_95%_HPD={6.922192922319603E-4,0.0032841122329325245},rate_median=0.0013769022806639948,rate_range={6.576719580218881E-4,0.0059192830724755}]:35.7871780339615)[&height=79.85403685468992,height_95%_HPD={61.91256207912734,96.53580643775122},height_median=80.00307833235995,height_range={54.44564040187407,107.94507944673339},length=20.914458967131434,length_95%_HPD={5.890417101414599,37.613129347070995},length_median=19.9728809936451,length_range={2.6207198577247652,49.52120424296692},posterior=1.0,rate=9.418232301286042E-4,rate_95%_HPD={3.350313830783981E-4,0.002035826408353378},rate_median=7.924465118060583E-4,rate_range={3.0410250535246603E-4,0.005332738281165591}]:20.91445896713111)[&height=100.76849582182103,height_95%_HPD={92.56551321404315,109.64050781437854},height_median=100.6369037067408,height_range={87.03010205923553,113.91433670663653},length=7.948166043489178,length_95%_HPD={3.0248833898017864,13.600102743552483},length_median=7.56010796769079,length_range={2.26705896517754,22.342872558613294},posterior=1.0,rate=0.002881519366851735,rate_95%_HPD={0.0012073123004344014,0.005000977272225884},rate_median=0.002676251109773313,rate_range={8.076802834102957E-4,0.008495619524871776}]:7.948166043489209,(((((((11[&height=2.245269588449449E-14,height_95%_HPD={0.0,5.6843418860808015E-14},height_median=2.8421709430404007E-14,height_range={0.0,8.526512829121202E-14},length=33.235554157345675,length_95%_HPD={21.818746873596126,43.748985287082576},length_median=32.976557203611335,length_range={18.776835366564512,53.39699994359019},rate=0.002462583616881023,rate_95%_HPD={0.0016823159657816061,0.0034106404859996268},rate_median=0.0024037209931373296,rate_range={0.0014543964607543955,0.004125918672667782}]:33.23555415734568,49[&height=2.245269588449449E-14,height_95%_HPD={0.0,5.6843418860808015E-14},height_median=2.8421709430404007E-14,height_range={0.0,8.526512829121202E-14},length=33.235554157345675,length_95%_HPD={21.818746873596126,43.748985287082576},length_median=32.976557203611335,length_range={18.776835366564512,53.39699994359019},rate=0.0016084754442260477,rate_95%_HPD={0.0010917363660577202,0.002216956665847974},rate_median=0.001573435560104169,rate_range={9.382970698513962E-4,0.002790117889139194}]:33.23555415734568)[&height=33.2355541573457,height_95%_HPD={21.818746873596155,43.748985287082604},height_median=32.976557203611335,height_range={18.776835366564512,53.39699994359022},length=8.528216952090258,length_95%_HPD={1.8545367555054213,17.174901851865158},length_median=7.663666128379845,length_range={1.2449348933904503,26.711060581252823},posterior=1.0,rate=0.0012495032926472338,rate_95%_HPD={3.100985446425915E-4,0.0027194183609090235},rate_median=0.0010612263354988249,rate_range={2.758688961495901E-4,0.006840263517264291}]:8.528216952090148,((52[&height=2.2307803123503826E-14,height_95%_HPD={0.0,5.6843418860808015E-14},height_median=2.8421709430404007E-14,height_range={0.0,8.526512829121202E-14},length=28.81598669249532,length_95%_HPD={17.588645881530454,39.91847001217795},length_median=28.870227470253084,length_range={12.181057843121337,47.45024882785307},rate=0.0017037476935369315,rate_95%_HPD={0.0010836511613754529,0.002488615808238566},rate_median=0.0016243825138819563,rate_range={9.683237093256061E-4,0.003664940116594913}]:28.815986692495315,71[&height=2.2307803123503826E-14,height_95%_HPD={0.0,5.6843418860808015E-14},height_median=2.8421709430404007E-14,height_range={0.0,8.526512829121202E-14},length=28.81598669249532,length_95%_HPD={17.588645881530454,39.91847001217795},length_median=28.870227470253084,length_range={12.181057843121337,47.45024882785307},rate=0.0016210901562508783,rate_95%_HPD={0.001039792221710986,0.0023483722912237023},rate_median=0.0015476187278358942,rate_range={9.736261899156812E-4,0.003557384661850221}]:28.815986692495315)[&height=28.815986692495336,height_95%_HPD={17.588645881530482,39.91847001217795},height_median=28.8702274702531,height_range={12.181057843121337,47.4502488278531},length=5.867772920775421,length_95%_HPD={0.9100210354865936,12.898757231426515},length_median=5.014849735165761,length_range={0.5680640952980411,26.46262467462519},posterior=1.0,rate=0.0011852494781168445,rate_95%_HPD={2.39719112231933E-4,0.0026250044543016876},rate_median=9.906746952178607E-4,rate_range={1.840763635025547E-4,0.00725270557662747}]:5.867772920775458,64[&height=2.2438490711848344E-14,height_95%_HPD={0.0,5.6843418860808015E-14},height_median=2.8421709430404007E-14,height_range={0.0,8.526512829121202E-14},length=34.68375961327078,length_95%_HPD={24.792852973060022,45.24471102595962},length_median=34.59965664435755,length_range={20.163747665511252,51.15410148498566},rate=0.00151639252068195,rate_95%_HPD={0.0010828060538236176,0.0020176606424695855},rate_median=0.0014845856477290155,rate_range={9.597835299316492E-4,0.002527704153272601}]:34.68375961327077)[&height=34.683759613270794,height_95%_HPD={24.79285297306005,45.244711025959646},height_median=34.59965664435758,height_range={20.16374766551128,51.15410148498566},length=7.080011496165225,length_95%_HPD={1.4230683206051822,14.95477194232005},length_median=6.219862141830987,length_range={1.0883245212949646,28.063101846910598},posterior=1.0,rate=0.0012873311244028687,rate_95%_HPD={2.4141340108574608E-4,0.002701791388084655},rate_median=0.0011141732360306611,rate_range={2.1891468915244722E-4,0.00627886020731648}]:7.080011496165056)[&height=41.76377110943585,height_95%_HPD={31.015043430749273,52.183854393269655},height_median=41.78327667755549,height_range={26.41644858722394,60.24486995023321},length=7.470402839092476,length_95%_HPD={1.5505120045226874,14.650862431185743},length_median=6.859029576763021,length_range={0.8752789916005952,27.312494625453937},posterior=1.0,rate=0.0014062120751081343,rate_95%_HPD={3.7158170696947776E-4,0.003116250075107781},rate_median=0.0011593322929351162,rate_range={2.9630005276867717E-4,0.00840751981375727}]:7.470402839092486,105[&height=2.2526562782254435E-14,height_95%_HPD={0.0,2.8421709430404007E-14},height_median=2.8421709430404007E-14,height_range={0.0,5.6843418860808015E-14},length=49.234173948528316,length_95%_HPD={38.47729005421786,59.80515984794937},length_median=49.36089065039344,length_range={32.23789887797767,66.59883300553933},rate=0.0010700416817030572,rate_95%_HPD={8.319862325666663E-4,0.0013201505596817124},rate_median=0.0010556994573695582,rate_range={7.688692594680542E-4,0.001680431284305418}]:49.234173948528316)[&height=49.23417394852834,height_95%_HPD={38.47729005421789,59.80515984794937},height_median=49.36089065039346,height_range={32.2378988779777,66.59883300553933},length=4.755112054783959,length_95%_HPD={1.0436496278516358,10.136854314201642},length_median=4.149167581903363,length_range={0.5913768949578895,22.28669720137836},posterior=1.0,rate=0.0011848299498183699,rate_95%_HPD={2.519094761653813E-4,0.0025260906943591075},rate_median=0.0010209997887547957,rate_range={1.9376475819012388E-4,0.00602392439251538}]:4.7551120547841705,(84[&height=2.2535085885842123E-14,height_95%_HPD={0.0,2.8421709430404007E-14},height_median=2.8421709430404007E-14,height_range={0.0,5.6843418860808015E-14},length=31.685546597732753,length_95%_HPD={14.19130842933751,46.812380758667786},length_median=31.727825947514823,length_range={12.369113834456854,59.74986160784053},rate=0.0013391951456481496,rate_95%_HPD={7.239634440054537E-4,0.002329614782886197},rate_median=0.0012254192792120935,rate_range={6.655610517355715E-4,0.003109151014502898}]:31.68554659773275,96[&height=2.2535085885842123E-14,height_95%_HPD={0.0,2.8421709430404007E-14},height_median=2.8421709430404007E-14,height_range={0.0,5.6843418860808015E-14},length=31.685546597732753,length_95%_HPD={14.19130842933751,46.812380758667786},length_median=31.727825947514823,length_range={12.369113834456854,59.74986160784053},rate=0.0016007996616791103,rate_95%_HPD={8.889640468779444E-4,0.0028231242133689442},rate_median=0.0014653744728643696,rate_range={7.61053259195885E-4,0.003866060422466371}]:31.68554659773275)[&height=31.68554659773277,height_95%_HPD={14.191308429337539,46.812380758667814},height_median=31.727825947514837,height_range={12.369113834456883,59.74986160784056},length=22.3037394055797,length_95%_HPD={6.850249227026708,36.99979024912909},length_median=22.231447416973396,length_range={4.375474915442254,48.28162271033362},posterior=1.0,rate=0.0012327498239085306,rate_95%_HPD={4.960127812856445E-4,0.0025404667303961753},rate_median=0.0010504889566034315,rate_range={4.7424642360129087E-4,0.0048946758363097986}]:22.303739405579737)[&height=53.98928600331251,height_95%_HPD={43.90460805494757,64.16903770430719},height_median=54.08999310705215,height_range={35.49040009570845,68.64536869003457},length=16.203261047159316,length_95%_HPD={5.868238262403537,27.883527805068127},length_median=15.406450946569535,length_range={4.989296567974407,42.17672462470854},posterior=1.0,rate=0.0018457674940650563,rate_95%_HPD={7.642183376447317E-4,0.003332662196728234},rate_median=0.001692303580942519,rate_range={6.416758262520852E-4,0.005464732270961557}]:16.203261047159238,((((22[&height=2.197256104905484E-14,height_95%_HPD={0.0,5.6843418860808015E-14},height_median=2.8421709430404007E-14,height_range={0.0,8.526512829121202E-14},length=6.284265165826189,length_95%_HPD={2.033809402812608,11.436904758579303},length_median=5.842434176257839,length_range={1.594441158536,21.163078428622427},rate=0.0020452202318580947,rate_95%_HPD={6.897393018454737E-4,0.0038718630154242917},rate_median=0.001858772224195042,rate_range={5.15601129961266E-4,0.007350974071547558}]:6.28426516582619,103[&height=2.197256104905484E-14,height_95%_HPD={0.0,5.6843418860808015E-14},height_median=2.8421709430404007E-14,height_range={0.0,8.526512829121202E-14},length=6.284265165826189,length_95%_HPD={2.033809402812608,11.436904758579303},length_median=5.842434176257839,length_range={1.594441158536,21.163078428622427},rate=9.174575824536062E-4,rate_95%_HPD={3.163232000451561E-4,0.0017258775541463794},rate_median=8.38416852597678E-4,rate_range={2.337597688302502E-4,0.0038337326226621313}]:6.28426516582619)[&height=6.284265165826212,height_95%_HPD={2.0338094028126363,11.436904758579331},height_median=5.842434176257882,height_range={1.5944411585360285,21.163078428622455},length=21.372543956230142,length_95%_HPD={9.74456360213074,34.31248336888174},length_median=20.774155678986872,length_range={7.959164702288831,44.38965817878605},posterior=1.0,rate=0.0029475754757862615,rate_95%_HPD={0.001404984648396866,0.004962679963870994},rate_median=0.0027358161576949026,rate_range={0.001256865687774406,0.00730717830605232}]:21.37254395623009,97[&height=2.2276551743682308E-14,height_95%_HPD={0.0,2.8421709430404007E-14},height_median=2.8421709430404007E-14,height_range={0.0,5.6843418860808015E-14},length=27.65680912205628,length_95%_HPD={14.223312917483355,40.23304049369102},length_median=27.278627897600074,length_range={11.932624744332557,53.318390411417184},rate=0.0012653224709138957,rate_95%_HPD={6.766961864365101E-4,0.0019406264043679896},rate_median=0.0011990252217526404,rate_range={5.930238210846235E-4,0.0027996032223715295}]:27.65680912205628)[&height=27.656809122056302,height_95%_HPD={14.223312917483355,40.23304049369105},height_median=27.278627897600103,height_range={11.932624744332585,53.318390411417184},length=28.15247502301127,length_95%_HPD={13.023879097593465,42.452918226010496},length_median=28.211059529098435,length_range={8.380172556854575,47.61049966795173},posterior=1.0,rate=0.0018995093356626587,rate_95%_HPD={0.0010497578641723048,0.0032649835053134724},rate_median=0.0017282278639338124,rate_range={0.001009285505742904,0.005689622777917518}]:28.15247502301117,((51[&height=2.221120794951005E-14,height_95%_HPD={0.0,5.6843418860808015E-14},height_median=2.8421709430404007E-14,height_range={0.0,8.526512829121202E-14},length=12.62092111811979,length_95%_HPD={4.103714123866098,23.25248438190613},length_median=11.522513018691903,length_range={3.529596824210273,37.36908655148241},rate=0.0019098900558621324,rate_95%_HPD={5.611589215424471E-4,0.003617640454837915},rate_median=0.0017602059830396357,rate_range={5.35517321134023E-4,0.005792798518857186}]:12.620921118119789,54[&height=2.221120794951005E-14,height_95%_HPD={0.0,5.6843418860808015E-14},height_median=2.8421709430404007E-14,height_range={0.0,8.526512829121202E-14},length=12.62092111811979,length_95%_HPD={4.103714123866098,23.25248438190613},length_median=11.522513018691903,length_range={3.529596824210273,37.36908655148241},rate=9.05262054994811E-4,rate_95%_HPD={2.710435006597661E-4,0.001695388399105412},rate_median=8.361499790714695E-4,rate_range={2.474595526944248E-4,0.002836329246438315}]:12.620921118119789)[&height=12.620921118119812,height_95%_HPD={4.103714123866126,23.252484381906157},height_median=11.522513018691917,height_range={3.5295968242103015,37.36908655148244},length=24.588970241194257,length_95%_HPD={8.497490853818306,40.380500303024874},length_median=23.879039214722567,length_range={5.500718777807634,51.05592297781067},posterior=1.0,rate=0.001281412825315019,rate_95%_HPD={5.647746400365662E-4,0.002396188936832447},rate_median=0.0011515598558884752,rate_range={5.271232017879993E-4,0.004736360283781989}]:24.58897024119424,74[&height=2.229643898538691E-14,height_95%_HPD={0.0,2.8421709430404007E-14},height_median=2.8421709430404007E-14,height_range={0.0,5.6843418860808015E-14},length=37.20989135931401,length_95%_HPD={22.380785781709335,53.98392083443841},length_median=37.215653093483,length_range={13.627141975522619,59.277353469850596},rate=0.0010213405262593867,rate_95%_HPD={6.15646493216575E-4,0.0015539474167064595},rate_median=9.651881489580517E-4,rate_range={5.482912315666193E-4,0.00257187612982112}]:37.20989135931403)[&height=37.20989135931405,height_95%_HPD={22.380785781709392,53.983920834438436},height_median=37.21565309348303,height_range={13.627141975522647,59.277353469850624},length=18.59939278575352,length_95%_HPD={5.392193834781807,32.93828719982797},length_median=17.886778346685574,length_range={3.805991386570213,44.026375114280484},posterior=1.0,rate=0.0011220563404772412,rate_95%_HPD={4.1248456300882377E-4,0.002390078065554776},rate_median=9.640877170479893E-4,rate_range={3.674763426997805E-4,0.004195897048209067}]:18.599392785753423)[&height=55.80928414506747,height_95%_HPD={41.242975419232806,67.104404876037},height_median=56.09979587972757,height_range={35.44584618347646,79.28479172984359},length=8.247125670708076,length_95%_HPD={0.9904988636772316,17.624991500718494},length_median=7.232912702641677,length_range={0.6615661399329724,32.52803469372867},posterior=1.0,rate=0.0011342528711887942,rate_95%_HPD={2.29936563191249E-4,0.00272392682441394},rate_median=8.892385262873664E-4,rate_range={1.8423231591461407E-4,0.007936149474709274}]:8.247125670708009,48[&height=2.236746484861763E-14,height_95%_HPD={0.0,2.8421709430404007E-14},height_median=2.8421709430404007E-14,height_range={0.0,5.6843418860808015E-14},length=64.05640981577545,length_95%_HPD={52.101672639990596,76.94420541631519},length_median=64.26241100352289,length_range={41.03650350905956,83.68997747238843},rate=0.001231518729826416,rate_95%_HPD={9.874761902000852E-4,0.0014880456397730068},rate_median=0.0012155275834443975,rate_range={8.790975069871251E-4,0.0019206289940431073}]:64.05640981577545)[&height=64.05640981577548,height_95%_HPD={52.101672639990625,76.94420541631521},height_median=64.26241100352291,height_range={41.036503509059585,83.68997747238846},length=6.136137234696094,length_95%_HPD={1.1640650224546079,13.641145337371213},length_median=5.176301898210596,length_range={0.8337980844780049,24.711423300364913},posterior=1.0,rate=0.0010696806528412916,rate_95%_HPD={2.1890588995875337E-4,0.002363306993925122},rate_median=9.038871049916828E-4,rate_range={1.8279377226303677E-4,0.0057162656121455034}]:6.136137234696264)[&height=70.19254705047175,height_95%_HPD={59.16351314138487,81.61970912929701},height_median=69.97634107301992,height_range={51.01516517026582,89.26008627865237},length=25.588693951978367,length_95%_HPD={13.387038760416303,39.66424334697771},length_median=25.19018537223758,length_range={8.620861290887277,46.367420802359774},posterior=1.0,rate=0.00207741030540895,rate_95%_HPD={0.001120336717993787,0.003295432688578203},rate_median=0.0019570989532279584,rate_range={0.0010506895571451126,0.0057838423427279215}]:25.0444974708996,((38[&height=2.241008036655606E-14,height_95%_HPD={0.0,2.8421709430404007E-14},height_median=2.8421709430404007E-14,height_range={0.0,5.6843418860808015E-14},length=66.15612150590313,length_95%_HPD={46.292041738422796,83.19213859718184},length_median=66.19576873415306,length_range={38.45629989870221,93.48285582967122},rate=0.0017155399311947167,rate_95%_HPD={0.0012754143993627964,0.002286482236171704},rate_median=0.0016695849166813784,rate_range={0.0011469284306850147,0.0029205174573236565}]:66.15612150590313,90[&height=2.241008036655606E-14,height_95%_HPD={0.0,2.8421709430404007E-14},height_median=2.8421709430404007E-14,height_range={0.0,5.6843418860808015E-14},length=66.15612150590313,length_95%_HPD={46.292041738422796,83.19213859718184},length_median=66.19576873415306,length_range={38.45629989870221,93.48285582967122},rate=0.0022003773668786944,rate_95%_HPD={0.0015977367025917914,0.002905293051469447},rate_median=0.002142863720623443,rate_range={0.0015041578705296361,0.003610754900022523}]:66.15612150590313)[&height=66.15612150590316,height_95%_HPD={46.292041738422824,83.19213859718187},height_median=66.19576873415309,height_range={38.45629989870224,93.48285582967125},length=14.488771578206661,length_95%_HPD={3.2988093503289235,28.813067191503592},length_median=13.107749931179661,length_range={1.897885875091859,40.298960548027225},posterior=1.0,rate=0.0013519908949986162,rate_95%_HPD={3.830576485825156E-4,0.0027572251664913635},rate_median=0.001187551292657451,rate_range={3.087929492593997E-4,0.007259743288414859}]:14.488771578206581,(91[&height=2.2390193124851456E-14,height_95%_HPD={0.0,2.8421709430404007E-14},height_median=2.8421709430404007E-14,height_range={0.0,5.6843418860808015E-14},length=15.235406679720862,length_95%_HPD={5.351385718923638,26.40452280442078},length_median=14.454788480369949,length_range={3.2101023990998385,41.57631944041053},rate=0.0012178686418319424,rate_95%_HPD={4.526487999273516E-4,0.0021579195419091403},rate_median=0.001108593014006975,rate_range={4.073901437635642E-4,0.005301899812448121}]:15.235406679720858,92[&height=2.2390193124851456E-14,height_95%_HPD={0.0,2.8421709430404007E-14},height_median=2.8421709430404007E-14,height_range={0.0,5.6843418860808015E-14},length=15.235406679720862,length_95%_HPD={5.351385718923638,26.40452280442078},length_median=14.454788480369949,length_range={3.2101023990998385,41.57631944041053},rate=0.0015947399656043451,rate_95%_HPD={5.824551411074774E-4,0.002789239665387317},rate_median=0.001454091453214477,rate_range={5.189330935924E-4,0.006454255130076271}]:15.235406679720858)[&height=15.235406679720882,height_95%_HPD={5.351385718923694,26.404522804420807},height_median=14.454788480369963,height_range={3.210102399099867,41.57631944041056},length=65.40948640438887,length_95%_HPD={41.75624497714506,82.08704284263163},length_median=66.37008952423881,length_range={35.49509608757489,91.49184497155561},posterior=1.0,rate=9.122717821465224E-4,rate_95%_HPD={6.545165807724004E-4,0.0012852693100927244},rate_median=8.736019387328366E-4,rate_range={6.296104939090438E-4,0.0015852098199707623}]:65.40948640438886)[&height=80.64489308410974,height_95%_HPD={63.048033484982085,97.17200311616939},height_median=81.79850344490106,height_range={47.79014916776646,101.62007541879295},length=14.587987027571852,length_95%_HPD={3.3654317080305134,31.29728297495184},length_median=12.461130075393001,length_range={1.9132811934695013,48.82594642123614},posterior=1.0,rate=0.0011556966498926973,rate_95%_HPD={2.708433326450693E-4,0.0024261061428101096},rate_median=0.0010277575267687664,rate_range={2.1732350618853513E-4,0.005586778564324959}]:14.592151437261606)[&height=95.23704452137135,height_95%_HPD={82.67307303435763,105.66069195122034},height_median=95.2454565418436,height_range={78.57828502942627,113.6409342523096},length=2.9963156060582388,length_95%_HPD={0.3984467033746597,7.071563464880754},length_median=2.4746660397583824,length_range={0.274868323540872,15.676982169020945},posterior=0.8848460615753698,rate=0.0011754205642828285,rate_95%_HPD={1.8496677499822188E-4,0.0026766717579198187},rate_median=9.685430107263019E-4,rate_range={1.7084429245912874E-4,0.007804446824787939}]:3.196737682485562,((((33[&height=2.2412921401085287E-14,height_95%_HPD={0.0,2.8421709430404007E-14},height_median=2.8421709430404007E-14,height_range={0.0,5.6843418860808015E-14},length=36.614521417874435,length_95%_HPD={18.731073420928624,53.4553054197726},length_median=36.19427043726351,length_range={13.942261016136555,73.8703494700301},rate=0.002853694483229438,rate_95%_HPD={0.0016007166729452701,0.0044738964665444305},rate_median=0.0027172101168515357,rate_range={0.0012441792007927654,0.0068059504418099895}]:36.61452141787444,82[&height=2.2412921401085287E-14,height_95%_HPD={0.0,2.8421709430404007E-14},height_median=2.8421709430404007E-14,height_range={0.0,5.6843418860808015E-14},length=36.614521417874435,length_95%_HPD={18.731073420928624,53.4553054197726},length_median=36.19427043726351,length_range={13.942261016136555,73.8703494700301},rate=0.002518408272364053,rate_95%_HPD={0.0014029299528987458,0.0039157768260469315},rate_median=0.0023899024593869666,rate_range={0.0011374547534453272,0.0060044741981018735}]:36.61452141787444)[&height=36.614521417874464,height_95%_HPD={18.731073420928652,53.45530541977263},height_median=36.194270437263555,height_range={13.942261016136584,73.8703494700301},length=30.45918659690007,length_95%_HPD={15.479469759190323,46.43324745826881},length_median=30.1010794861225,length_range={11.794868618384768,54.17558767629839},posterior=1.0,rate=0.0036667185566489104,rate_95%_HPD={0.0019442857661332252,0.005931249678791528},rate_median=0.0034217618291483274,rate_range={0.0018034300070386279,0.008055508131447805}]:30.45918659690009,72[&height=2.238735209032223E-14,height_95%_HPD={0.0,2.8421709430404007E-14},height_median=2.8421709430404007E-14,height_range={0.0,5.6843418860808015E-14},length=67.07370801477454,length_95%_HPD={49.12337872178249,83.52950104815537},length_median=66.91470266413168,length_range={42.75683736659556,94.02314605055567},rate=6.950304049922688E-4,rate_95%_HPD={5.140471261634678E-4,9.019255270825292E-4},rate_median=6.827262805214008E-4,rate_range={4.630380716739419E-4,0.001105223069788399}]:67.07370801477452)[&height=67.07370801477455,height_95%_HPD={49.123378721782515,83.5295010481554},height_median=66.91470266413171,height_range={42.75683736659559,94.02314605055567},length=8.373404404854675,length_95%_HPD={1.733243187578509,16.979946302218806},length_median=7.607336001275776,length_range={1.0231323504425518,29.543145351763158},posterior=1.0,rate=0.0013061604673460937,rate_95%_HPD={3.3790763788643343E-4,0.0028200319471822777},rate_median=0.0010923084696680397,rate_range={2.8733072078791994E-4,0.008306438156767487}]:8.373404404854497,66[&height=2.238735209032223E-14,height_95%_HPD={0.0,2.8421709430404007E-14},height_median=2.8421709430404007E-14,height_range={0.0,5.6843418860808015E-14},length=75.44711241962905,length_95%_HPD={57.860725561228634,91.66564695388476},length_median=75.72402261339668,length_range={49.03711756958742,101.20619689182085},rate=8.375096102273833E-4,rate_95%_HPD={6.563376488736173E-4,0.0010537386398240187},rate_median=8.235580713789627E-4,rate_range={5.838696447149133E-4,0.0012881042365590078}]:75.44711241962902)[&height=75.44711241962905,height_95%_HPD={57.86072556122866,91.66564695388476},height_median=75.72402261339671,height_range={49.03711756958745,101.20619689182088},length=19.533126303845027,length_95%_HPD={6.698571422231382,35.605326371572914},length_median=18.873221655832175,length_range={2.415471366162336,45.698643724309875},posterior=1.0,rate=0.0014292362860821547,rate_95%_HPD={5.341438829270653E-4,0.0026218950590979765},rate_median=0.001227948369635089,rate_range={5.062250262180925E-4,0.00871093356772409}]:19.393234152320602,((44[&height=2.231916726162074E-14,height_95%_HPD={0.0,2.8421709430404007E-14},height_median=2.8421709430404007E-14,height_range={0.0,5.6843418860808015E-14},length=17.717914186878524,length_95%_HPD={5.886010265703277,30.12817758407988},length_median=16.741035106776955,length_range={5.720961913927539,44.6781884693016},rate=0.001316616266309543,rate_95%_HPD={4.969909271526553E-4,0.002264601296871181},rate_median=0.0012053973854879731,rate_range={4.3442064982216296E-4,0.0036984417887838696}]:17.717914186878527,99[&height=2.231916726162074E-14,height_95%_HPD={0.0,2.8421709430404007E-14},height_median=2.8421709430404007E-14,height_range={0.0,5.6843418860808015E-14},length=17.717914186878524,length_95%_HPD={5.886010265703277,30.12817758407988},length_median=16.741035106776955,length_range={5.720961913927539,44.6781884693016},rate=0.0011269387764081076,rate_95%_HPD={4.1262320695818733E-4,0.0019170630381236393},rate_median=0.0010382464211938656,rate_range={3.616878821475795E-4,0.0030873742776204934}]:17.717914186878527)[&height=17.71791418687855,height_95%_HPD={5.886010265703305,30.128177584079907},height_median=16.741035106776977,height_range={5.720961913927567,44.6781884693016},length=36.43498328323707,length_95%_HPD={15.750578628734615,58.84998516387019},length_median=34.87619456440777,length_range={9.0642981739378,78.61378188317312},posterior=1.0,rate=7.577676721940242E-4,rate_95%_HPD={3.418382378692694E-4,0.0012766976646469497},rate_median=6.954303067466582E-4,rate_range={2.999313630355364E-4,0.002527889985803615}]:36.43498328323709,45[&height=2.2393034159380687E-14,height_95%_HPD={0.0,2.8421709430404007E-14},height_median=2.8421709430404007E-14,height_range={0.0,5.6843418860808015E-14},length=54.15289747011561,length_95%_HPD={30.93935240879344,76.80878897219205},length_median=53.76592137178884,length_range={27.25414660048844,96.27016635598395},rate=0.0015782058278521521,rate_95%_HPD={9.88250441176242E-4,0.0024497924069916965},rate_median=0.0015011787881955188,rate_range={8.141493482378943E-4,0.0030297228809780183}]:54.15289747011561)[&height=54.15289747011563,height_95%_HPD={30.939352408793468,76.80878897219208},height_median=53.76592137178887,height_range={27.25414660048847,96.27016635598395},length=40.65780071043008,length_95%_HPD={15.153977962161463,62.798117733632054},length_median=41.0978583179127,length_range={6.807424087082794,74.92791413202231},posterior=1.0,rate=8.02863130702515E-4,rate_95%_HPD={3.873531534720499E-4,0.0015728203042059236},rate_median=6.862523541150121E-4,rate_range={3.704673735039235E-4,0.004023997696543351}]:40.68744910183402)[&height=94.84034657194965,height_95%_HPD={82.82111081135781,106.85303576628844},height_median=94.75471550142854,height_range={74.77473610017059,114.73140353369129},length=3.2283302075943707,length_95%_HPD={0.49823610485829306,7.8684164470525815},length_median=2.619927934265057,length_range={0.3049045995720263,16.55847836662005},posterior=0.9524190323870452,rate=0.0010415559193490004,rate_95%_HPD={1.8496677499822188E-4,0.0023504746311251595},rate_median=8.780625291424264E-4,rate_range={1.6594440088969306E-4,0.007191286666249116}]:3.593435631907255)[&height=98.43378220385691,height_95%_HPD={87.2996957194187,110.06573089719274},height_median=98.50160350847483,height_range={79.86372031382987,115.78616403233586},length=10.282879661453464,length_95%_HPD={2.563774719816678,19.546380463962095},length_median=9.599289684733222,length_range={1.0888197625682494,28.437346818375204},posterior=1.0,rate=0.0012037796955042509,rate_95%_HPD={3.951825039880123E-4,0.002502426570869773},rate_median=0.0010307703864886832,rate_range={2.4721637064023256E-4,0.007677717349230556}]:10.282879661453336)[&height=108.71666186531024,height_95%_HPD={100.12991899865467,117.13232039880492},height_median=108.88067802587648,height_range={93.6906749917519,121.9320939154767},length=19.80174633945941,length_95%_HPD={12.4388572208168,28.07967278112561},length_median=19.230947731036977,length_range={6.826496906311448,35.93944917252078},posterior=1.0,rate=0.006401765290578239,rate_95%_HPD={0.00418976473773697,0.008663116882750779},rate_median=0.006459524594374208,rate_range={0.003065085215467413,0.010644221159774144}]:19.80174633945947,(9[&height=2.244985484996526E-14,height_95%_HPD={0.0,2.8421709430404007E-14},height_median=2.8421709430404007E-14,height_range={0.0,5.6843418860808015E-14},length=38.048502389040536,length_95%_HPD={0.007156157411174036,105.90971443512562},length_median=28.2180248864882,length_range={0.007156157411174036,133.00157752836955},rate=0.0015318506458492352,rate_95%_HPD={2.4734346349031266E-4,0.003470085737088985},rate_median=0.0012847982333038287,rate_range={1.8600630861785047E-4,0.00872641238378252}]:38.04850238904055,21[&height=2.244985484996526E-14,height_95%_HPD={0.0,2.8421709430404007E-14},height_median=2.8421709430404007E-14,height_range={0.0,5.6843418860808015E-14},length=38.048502389040536,length_95%_HPD={0.007156157411174036,105.90971443512562},length_median=28.2180248864882,length_range={0.007156157411174036,133.00157752836955},rate=0.002236892121095078,rate_95%_HPD={2.4333343941392488E-4,0.004961081081164858},rate_median=0.0018784456662187645,rate_range={1.8397158237754723E-4,0.009033803460152048}]:38.04850238904055)[&height=38.04850238904057,height_95%_HPD={0.007156157411202457,105.90971443512565},height_median=28.21802488648823,height_range={0.007156157411202457,133.00157752836955},length=90.4699058157291,length_95%_HPD={21.98561842400001,134.762008960598},length_median=99.62855043692069,length_range={0.07536792460894048,137.04017797029977},posterior=1.0,rate=0.0030824642842646305,rate_95%_HPD={5.459007129423535E-4,0.004750358216392788},rate_median=0.003167730447014847,rate_range={2.3189390487564162E-4,0.008351028813463592}]:90.46990581572913)[&height=128.5184082047697,height_95%_HPD={119.29404619801174,136.64801247105856},height_median=129.06182897400856,height_range={109.6313224768481,138.03641381615935},length=4.733903606004432,length_95%_HPD={0.003769477469475646,12.133883111305806},length_median=3.889130862092763,length_range={0.003769477469475646,23.679116799117338},posterior=1.0,rate=0.0019388580524078009,rate_95%_HPD={2.1745548236112812E-4,0.004453474555525313},rate_median=0.0016234054211161971,rate_range={2.0145322357675307E-4,0.009148096367109965}]:4.733903606004617,(((4[&height=2.2381670021263772E-14,height_95%_HPD={0.0,2.8421709430404007E-14},height_median=2.8421709430404007E-14,height_range={0.0,5.6843418860808015E-14},length=92.28894736959214,length_95%_HPD={69.38860639842115,114.19440065540122},length_median=93.04817644314812,length_range={37.613498909086715,126.4511283475838},rate=0.002476195824988776,rate_95%_HPD={0.0018949811728988623,0.003119733729874423},rate_median=0.002414908820812684,rate_range={0.0017282599075686348,0.005638820864431991}]:92.90090677988631,(25[&height=2.2378828986734544E-14,height_95%_HPD={0.0,2.8421709430404007E-14},height_median=2.8421709430404007E-14,height_range={0.0,5.6843418860808015E-14},length=59.78323027555127,length_95%_HPD={35.20428020788306,88.17387786202498},length_median=58.80045821703713,length_range={22.80758225221109,103.23196262780675},rate=0.002737283003967294,rate_95%_HPD={0.001562476659572243,0.004050565720875973},rate_median=0.0026383805851759364,rate_range={0.0014364478698953328,0.006884924814586705}]:59.78323027555127,26[&height=2.2378828986734544E-14,height_95%_HPD={0.0,2.8421709430404007E-14},height_median=2.8421709430404007E-14,height_range={0.0,5.6843418860808015E-14},length=59.78323027555127,length_95%_HPD={35.20428020788306,88.17387786202498},length_median=58.80045821703713,length_range={22.80758225221109,103.23196262780675},rate=0.0027341763901484665,rate_95%_HPD={0.001627285576659945,0.004076892142809825},rate_median=0.0026327827429992318,rate_range={0.0014129310134766348,0.006884924814586705}]:59.78323027555127)[&height=59.78323027555129,height_95%_HPD={35.20428020788309,88.17387786202498},height_median=58.80045821703716,height_range={22.80758225221112,103.23196262780678},length=33.71557738037738,length_95%_HPD={12.086581681630705,55.77777991247331},length_median=33.02016442110797,length_range={6.856358492090848,73.85610992218898},posterior=1.0,rate=0.0018994876060816875,rate_95%_HPD={8.026496521568172E-4,0.0034828938895575118},rate_median=0.001695666068560902,rate_range={6.334321031952813E-4,0.007417987033650814}]:33.11767650433505)[&height=92.90090677988634,height_95%_HPD={72.63116494766942,113.46498359089622},height_median=93.32105053829916,height_range={48.01990900211712,126.4511283475838},length=13.23851679907343,length_95%_HPD={0.023927967368891245,29.604756285358917},length_median=11.437702127375658,length_range={0.023927967368891245,58.14875622962731},posterior=0.6951219512195121,rate=0.001317637997754028,rate_95%_HPD={2.3115555543547022E-4,0.0028240607161367608},rate_median=0.0011192966919239939,rate_range={1.7933800224042483E-4,0.008485679466220732}]:10.852343038652151,5[&height=2.2381670021263772E-14,height_95%_HPD={0.0,2.8421709430404007E-14},height_median=2.8421709430404007E-14,height_range={0.0,5.6843418860808015E-14},length=100.43927246423101,length_95%_HPD={72.63028449020277,125.25641010751501},length_median=102.42200133235937,length_range={37.613498909086715,130.48189021017902},rate=0.0035028325156397323,rate_95%_HPD={0.002471616520686712,0.004771336734519659},rate_median=0.0033684257950542017,rate_range={0.0021481525029571056,0.008812852589505748}]:103.75324981853846)[&height=103.75324981853849,height_95%_HPD={81.41768869344371,124.1052693148393},height_median=104.77986571711274,height_range={54.4802158548803,130.48189021017905},length=15.201903513536084,length_95%_HPD={0.025286347594956737,34.3110831184937},length_median=13.30579039458052,length_range={0.025286347594956737,71.17669892986527},posterior=1.0,rate=0.0013525394857492963,rate_95%_HPD={2.4562159476099887E-4,0.0029159671995137057},rate_median=0.0011550761877406443,rate_range={1.640587247269195E-4,0.008838731702506309}]:15.201903513535783,(42[&height=2.2378828986734544E-14,height_95%_HPD={0.0,2.8421709430404007E-14},height_median=2.8421709430404007E-14,height_range={0.0,5.6843418860808015E-14},length=63.513933389485274,length_95%_HPD={39.03835948450531,88.80476830739688},length_median=63.98026995040556,length_range={28.491950493855626,99.62142746996376},rate=0.0027254680270923544,rate_95%_HPD={0.0017490632271171293,0.00404544199142932},rate_median=0.0025895945483854848,rate_range={0.0015166921137535583,0.005706090905130837}]:63.513933389485274,104[&height=2.2378828986734544E-14,height_95%_HPD={0.0,2.8421709430404007E-14},height_median=2.8421709430404007E-14,height_range={0.0,5.6843418860808015E-14},length=63.513933389485274,length_95%_HPD={39.03835948450531,88.80476830739688},length_median=63.98026995040556,length_range={28.491950493855626,99.62142746996376},rate=0.002626392206848774,rate_95%_HPD={0.0016707569586051163,0.003911657836852218},rate_median=0.002501237825202789,rate_range={0.0015547091719573173,0.005706090905130837}]:63.513933389485274)[&height=63.513933389485295,height_95%_HPD={39.038359484505335,88.80476830739691},height_median=63.98026995040557,height_range={28.491950493855654,99.62142746996379},length=55.44121994258887,length_95%_HPD={29.358168577265147,79.74038297856467},length_median=55.12835723740084,length_range={22.46158581418875,100.98957278076867},posterior=1.0,rate=0.004021071248907873,rate_95%_HPD={0.0022419293877729953,0.00613590509262263},rate_median=0.0038118575335294963,rate_range={0.001995447705554368,0.009165488555813582}]:55.44121994258898)[&height=118.95515333207427,height_95%_HPD={105.6511373612146,132.6906050706449},height_median=119.6529226495093,height_range={89.72243350087301,135.01343316420022},length=14.297158478699854,length_95%_HPD={2.795766954925938,27.377080622279806},length_median=13.28346031349821,length_range={1.7294227697562121,46.04034423956041},posterior=1.0,rate=0.0017681814243208874,rate_95%_HPD={4.3786063404552196E-4,0.0036647960871949146},rate_median=0.001523167088038455,rate_range={3.838503333885875E-4,0.008783932480154043}]:14.297158478700055)[&height=133.25231181077433,height_95%_HPD={126.85666787045919,138.17965909921335},height_median=133.85865334431458,height_range={119.12364886284945,138.27235746918967},length=2.5390975930497452,length_95%_HPD={1.3531751369555423E-4,7.528884155256293},length_median=1.840811417372862,length_range={1.3531751369555423E-4,17.454456964531403},posterior=1.0,rate=0.001578256741684096,rate_95%_HPD={2.1113992184676385E-4,0.0035972366750977574},rate_median=0.0012991467739894942,rate_range={2.1113992184676385E-4,0.00939835484256311}]:2.5390975930494903,67[&height=2.2381670021263772E-14,height_95%_HPD={0.0,2.8421709430404007E-14},height_median=2.8421709430404007E-14,height_range={0.0,5.6843418860808015E-14},length=135.79140940382376,length_95%_HPD={131.16023428311922,138.32964427982085},length_median=136.4467487660263,length_range={120.3300965273882,138.32964427982085},rate=0.0025895951614853785,rate_95%_HPD={0.0024067529821217627,0.0027907824037297323},rate_median=0.0025879328897538212,rate_range={0.0018208097906525199,0.0030871431441563783}]:135.7914094038238)[&height=135.79140940382382,height_95%_HPD={131.16023428311925,138.32964427982088},height_median=136.44674876602636,height_range={120.33009652738822,138.32964427982088},length=0.0,posterior=1.0,rate=1.0]:0.0;

tree TREE2 = ((((((((((((((((1[&height=2.3094769688100177E-14,height_95%_HPD={0.0,5.6843418860808015E-14},height_median=2.8421709430404007E-14,height_range={0.0,8.526512829121202E-14},length=10.57198577970274,length_95%_HPD={4.662228091327876,17.511388809089055},length_median=10.01966841620927,length_range={3.0994696682141125,25.804794350582057},rate=0.0017416477034201089,rate_95%_HPD={7.278313237310818E-4,0.0030200614225392632},rate_median=0.0016344729379565912,rate_range={6.109196239497854E-4,0.004798752709792675}]:10.571985779702747,23[&height=2.3094769688100177E-14,height_95%_HPD={0.0,5.6843418860808015E-14},height_median=2.8421709430404007E-14,height_range={0.0,8.526512829121202E-14},length=10.57198577970274,length_95%_HPD={4.662228091327876,17.511388809089055},length_median=10.01966841620927,length_range={3.0994696682141125,25.804794350582057},rate=0.0023110449406375092,rate_95%_HPD={0.0010066839835002575,0.004071429600069134},rate_median=0.002167836829820639,rate_range={7.34537781981229E-4,0.006611419433234089}]:10.571985779702747)[&height=10.57198577970277,height_95%_HPD={4.662228091327933,17.511388809089084},height_median=10.019668416209285,height_range={3.099469668214141,25.804794350582085},length=3.2827696462753475,length_95%_HPD={0.502217022205798,7.563481504878027},length_median=2.779161547641891,length_range={0.2569528326818613,15.853503096510224},posterior=1.0,rate=0.0016671932239364221,rate_95%_HPD={2.7508632301215393E-4,0.003714488987340306},rate_median=0.0013893170687804307,rate_range={2.565278139718228E-4,0.013145232862792304}]:3.282769646275316,41[&height=2.3063518308278662E-14,height_95%_HPD={0.0,5.6843418860808015E-14},height_median=2.8421709430404007E-14,height_range={0.0,8.526512829121202E-14},length=13.854755425978068,length_95%_HPD={6.873652067303425,21.26186582431629},length_median=13.415657434040703,length_range={5.5242793534482075,31.262811170997438},rate=0.0019095819636863322,rate_95%_HPD={9.981647333256684E-4,0.0031260497293817183},rate_median=0.0018070818891057104,rate_range={7.557319479700495E-4,0.004372291650811136}]:13.854755425978063)[&height=13.854755425978086,height_95%_HPD={6.873652067303453,21.261865824316345},height_median=13.415657434040732,height_range={5.524279353448236,31.262811170997438},length=12.77805121089117,length_95%_HPD={3.9440853090206645,23.133887078180635},length_median=11.79393768617608,length_range={3.102989391811775,38.180076876426284},posterior=1.0,rate=0.0026831838141361127,rate_95%_HPD={9.063101820369494E-4,0.0050694484113465174},rate_median=0.002422203181310696,rate_range={7.417386471633417E-4,0.010015926148989959}]:12.74834550594431,27[&height=2.3168636585860123E-14,height_95%_HPD={0.0,5.6843418860808015E-14},height_median=2.8421709430404007E-14,height_range={0.0,8.526512829121202E-14},length=26.57489061103848,length_95%_HPD={13.985727605557742,39.65200393353584},length_median=26.289756681397833,length_range={10.564629471269981,49.04969183787881},rate=0.001869230876969596,rate_95%_HPD={9.950876172243747E-4,0.0029162134346112536},rate_median=0.0017785856950607677,rate_range={9.15237184602701E-4,0.004487016004225484}]:26.60310093192237)[&height=26.603100931922395,height_95%_HPD={13.98572760555777,39.83547156892527},height_median=26.332319617598067,height_range={10.56462947127001,49.04969183787884},length=2.4219027578603742,length_95%_HPD={0.23329169142672868,5.839266038547507},length_median=1.9703799531100472,length_range={0.13341860941412165,15.3199222475438},posterior=0.9656137544982007,rate=0.001576530305972572,rate_95%_HPD={2.4057698820512542E-4,0.0036238540227139454},rate_median=0.0012980257983840147,rate_range={2.241770686417136E-4,0.012695700714839529}]:2.3717421256216795,77[&height=2.302658485939869E-14,height_95%_HPD={0.0,2.8421709430404007E-14},height_median=2.8421709430404007E-14,height_range={0.0,8.526512829121202E-14},length=28.9100994204578,length_95%_HPD={15.547814975649985,42.31024360487268},length_median=28.6807430170322,length_range={11.880589254550998,52.79968542874519},rate=0.0020577242158380795,rate_95%_HPD={0.0011281871783003735,0.003049330458113896},rate_median=0.001958782422440475,rate_range={0.0010413890677521142,0.004633603449155341}]:28.974843057544053)[&height=28.974843057544074,height_95%_HPD={15.547814975650013,42.183401246587025},height_median=28.720653185132576,height_range={11.880589254551026,52.79968542874522},length=4.370518107620774,length_95%_HPD={0.7741358355167876,9.672315949353589},length_median=3.694370048330242,length_range={0.3957091555582082,19.398308858084846},posterior=1.0,rate=0.00166468516514397,rate_95%_HPD={2.979251087575505E-4,0.0036104890970937433},rate_median=0.0014244354939340097,rate_range={2.472973749461723E-4,0.013828186386630406}]:4.370518107620857,(63[&height=2.3341939692143074E-14,height_95%_HPD={0.0,2.8421709430404007E-14},height_median=2.8421709430404007E-14,height_range={0.0,8.526512829121202E-14},length=14.775778568427794,length_95%_HPD={6.746973318337865,26.081875497073298},length_median=13.838304427857402,length_range={4.81147153594344,35.671914775211604},rate=0.0012293112549968037,rate_95%_HPD={5.173128764659682E-4,0.0020427892379139517},rate_median=0.0011664324539623922,rate_range={4.244865946935343E-4,0.0036558581691482784}]:14.775778568427793,100[&height=2.3341939692143074E-14,height_95%_HPD={0.0,2.8421709430404007E-14},height_median=2.8421709430404007E-14,height_range={0.0,8.526512829121202E-14},length=14.775778568427794,length_95%_HPD={6.746973318337865,26.081875497073298},length_median=13.838304427857402,length_range={4.81147153594344,35.671914775211604},rate=0.0015901463857011394,rate_95%_HPD={6.988758939090184E-4,0.0026212431004865658},rate_median=0.0015208231422778528,rate_range={5.457152786804329E-4,0.004354696330740592}]:14.775778568427793)[&height=14.775778568427816,height_95%_HPD={6.746973318337922,26.081875497073327},height_median=13.83830442785743,height_range={4.811471535943468,35.67191477521163},length=18.56958259673701,length_95%_HPD={5.78782705157289,32.86072969211506},length_median=18.046858478172986,length_range={1.9897404248936397,45.49853174051424},posterior=1.0,rate=0.0015151225707127854,rate_95%_HPD={5.801302992760534E-4,0.0031224841983165407},rate_median=0.001265294372207956,rate_range={4.6233881500467065E-4,0.01111201644686514}]:18.569582596737114)[&height=33.34536116516493,height_95%_HPD={18.4063689207765,46.617782285020354},height_median=33.34863944448685,height_range={17.056243151438707,57.17172248087084},length=10.982976126916398,length_95%_HPD={1.5580522986795557,23.560163067087743},length_median=9.583591501482616,length_range={1.3148897833856381,36.146315803349225},posterior=1.0,rate=0.0015353362729404638,rate_95%_HPD={3.0955223450646594E-4,0.003622228583326083},rate_median=0.0012454846648311557,rate_range={2.990054534471053E-4,0.008402146675394483}]:10.982976126916192,81[&height=2.3231139345503156E-14,height_95%_HPD={0.0,2.8421709430404007E-14},height_median=2.8421709430404007E-14,height_range={0.0,5.6843418860808015E-14},length=44.328337292081095,length_95%_HPD={28.623947766177224,60.42205928938269},length_median=44.07437105882679,length_range={22.9902986420785,72.32705143142758},rate=0.0016192525796487128,rate_95%_HPD={0.001085339920282226,0.002316689152997714},rate_median=0.0015713698792188934,rate_range={9.353330370437944E-4,0.0030036150656740203}]:44.3283372920811)[&height=44.32833729208112,height_95%_HPD={28.623947766177253,60.422059289382744},height_median=44.07437105882682,height_range={22.99029864207853,72.3270514314276},length=25.08944868660036,length_95%_HPD={8.911777747640855,39.11652877619905},length_median=24.87805929560352,length_range={6.812577189059709,48.57048503042476},posterior=1.0,rate=8.867875216030769E-4,rate_95%_HPD={3.976113694693507E-4,0.0017013847052569741},rate_median=7.876604812272839E-4,rate_range={3.5405106820130704E-4,0.0027612664527865943}]:25.089448686600555,(78[&height=2.3276595897970813E-14,height_95%_HPD={0.0,2.8421709430404007E-14},height_median=2.8421709430404007E-14,height_range={0.0,5.6843418860808015E-14},length=35.85056820684154,length_95%_HPD={9.184532083926143,59.167505545732666},length_median=34.730681413207954,length_range={7.48129143454841,69.05864797468897},rate=0.0012838267037888636,rate_95%_HPD={5.362683308228313E-4,0.0030691088965480264},rate_median=0.0010654129744524584,rate_range={4.7015042308580984E-4,0.005021246348815808}]:35.85056820684154,86[&height=2.3276595897970813E-14,height_95%_HPD={0.0,2.8421709430404007E-14},height_median=2.8421709430404007E-14,height_range={0.0,5.6843418860808015E-14},length=35.85056820684154,length_95%_HPD={9.184532083926143,59.167505545732666},length_median=34.730681413207954,length_range={7.48129143454841,69.05864797468897},rate=9.282867752507826E-4,rate_95%_HPD={3.852633206471256E-4,0.0022083648416676652},rate_median=7.666742151871783E-4,rate_range={3.6135819509911323E-4,0.0035623149416025013}]:35.85056820684154)[&height=35.85056820684156,height_95%_HPD={9.184532083926172,59.167505545732695},height_median=34.73068141320798,height_range={7.481291434548439,69.05864797468897},length=33.567217771839964,length_95%_HPD={7.53684723230127,57.60370125072123},length_median=34.167370144288405,length_range={5.587038666328567,74.05519636501184},posterior=1.0,rate=9.435391778410968E-4,rate_95%_HPD={3.1906029615251036E-4,0.0022383934829217976},rate_median=7.258369864666965E-4,rate_range={3.117789831285612E-4,0.004342979314694308}]:33.56721777184012)[&height=69.41778597868168,height_95%_HPD={66.00152711477935,76.43443696593273},height_median=68.44301504200686,height_range={66.00152711477935,86.92158493905214},length=10.154143088293166,length_95%_HPD={3.537040555982287,18.004526765121255},length_median=9.502135665922154,length_range={2.9576720886060315,26.908849370393384},posterior=1.0,rate=0.0022184439794677904,rate_95%_HPD={8.045175012908644E-4,0.004084978391416545},rate_median=0.0020407570888774816,rate_range={6.604447930026441E-4,0.006923382449342851}]:10.154143088292756,((((((((((2[&height=2.3645930386770547E-14,height_95%_HPD={0.0,5.6843418860808015E-14},height_median=2.8421709430404007E-14,height_range={0.0,8.526512829121202E-14},length=15.8509725991337,length_95%_HPD={8.12246304663617,24.670811515273925},length_median=15.525209566401998,length_range={5.177031273332176,32.04031259234989},rate=0.0018224523065053005,rate_95%_HPD={9.337215213679271E-4,0.0029587149241543454},rate_median=0.0017119771392029117,rate_range={7.840608029516156E-4,0.005546378345941142}]:15.850972599133694,40[&height=2.3645930386770547E-14,height_95%_HPD={0.0,5.6843418860808015E-14},height_median=2.8421709430404007E-14,height_range={0.0,8.526512829121202E-14},length=15.8509725991337,length_95%_HPD={8.12246304663617,24.670811515273925},length_median=15.525209566401998,length_range={5.177031273332176,32.04031259234989},rate=0.0022910549076169094,rate_95%_HPD={0.0012136965696289098,0.003730937757223051},rate_median=0.00215262193286982,rate_range={9.767505294414295E-4,0.006591327552536995}]:15.850972599133694)[&height=15.850972599133717,height_95%_HPD={8.122463046636199,24.670811515273982},height_median=15.525209566402026,height_range={5.177031273332204,32.04031259234989},length=10.181085611783306,length_95%_HPD={2.5367426935459747,18.338297929972867},length_median=9.86191115718512,length_range={1.583706488491515,31.213209494955862},posterior=1.0,rate=0.002170296227736399,rate_95%_HPD={6.851981571723995E-4,0.004417229306178764},rate_median=0.0018238536753246624,rate_range={5.091952813346206E-4,0.010905921837050974}]:10.181085611783368,((32[&height=2.3236821414561614E-14,height_95%_HPD={0.0,5.6843418860808015E-14},height_median=2.8421709430404007E-14,height_range={0.0,8.526512829121202E-14},length=10.717793997138914,length_95%_HPD={4.797346477770986,18.005178258098226},length_median=10.169855118420735,length_range={2.602637246691131,25.40688641668831},rate=0.0037349460256015953,rate_95%_HPD={0.0016418115450314232,0.006180676224114626},rate_median=0.003520930239661835,rate_range={0.00130344098832445,0.013017288473728246}]:10.71779399713891,58[&height=2.3236821414561614E-14,height_95%_HPD={0.0,5.6843418860808015E-14},height_median=2.8421709430404007E-14,height_range={0.0,8.526512829121202E-14},length=10.717793997138914,length_95%_HPD={4.797346477770986,18.005178258098226},length_median=10.169855118420735,length_range={2.602637246691131,25.40688641668831},rate=0.0012500665621323435,rate_95%_HPD={4.951926202645201E-4,0.0020765391256357584},rate_median=0.0011742078932539543,rate_range={4.3069321247518177E-4,0.0045163458706176255}]:10.71779399713891)[&height=10.717793997138934,height_95%_HPD={4.797346477770986,18.005178258098226},height_median=10.169855118420735,height_range={2.6026372466911596,25.40688641668831},length=8.253232595672936,length_95%_HPD={2.644625761800455,14.697719572416048},length_median=7.753773364141658,length_range={1.4547032148336854,26.418094749051022},posterior=1.0,rate=0.002341305059042498,rate_95%_HPD={7.520869121267972E-4,0.004623641631959329},rate_median=0.0020724469791418725,rate_range={5.418418118280896E-4,0.010817118531096672}]:8.253232595672902,55[&height=2.3194205896623183E-14,height_95%_HPD={0.0,5.6843418860808015E-14},height_median=2.8421709430404007E-14,height_range={0.0,8.526512829121202E-14},length=18.971026592811818,length_95%_HPD={10.83248616075332,27.332403175157708},length_median=18.756629944963557,length_range={8.56902073763402,36.21414260185796},rate=0.0019479496973833132,rate_95%_HPD={0.0011798933868106536,0.0030000116985257497},rate_median=0.0018690478853093002,rate_range={8.994203516742637E-4,0.004035168647797294}]:18.97102659281181)[&height=18.971026592811835,height_95%_HPD={10.83248616075332,27.332403175157708},height_median=18.756629944963585,height_range={8.569020737634048,36.214142601857986},length=7.0610316181052,length_95%_HPD={1.7820176803786723,13.31026356711699},length_median=6.6022985649759605,length_range={0.8693489592148183,22.30936722489477},posterior=1.0,rate=0.0022386719750457622,rate_95%_HPD={6.163704142608342E-4,0.004471445284125461},rate_median=0.0019620599508188338,rate_range={5.587514292902034E-4,0.014399363720117688}]:7.06103161810525)[&height=26.032058210917086,height_95%_HPD={17.46681928712985,35.199316616742124},height_median=25.822764629809548,height_range={14.267630430502152,43.2297990166893},length=4.205251069395685,length_95%_HPD={0.7993501192654691,8.781404194058425},length_median=3.799600129469546,length_range={0.3866651636437126,14.881706802157368},posterior=1.0,rate=0.002213134997069018,rate_95%_HPD={5.118960309218368E-4,0.004922695195367776},rate_median=0.0018134537870159416,rate_range={4.4201228812124034E-4,0.014258600563659313}]:4.205251069395647,(39[&height=2.3302165208733874E-14,height_95%_HPD={0.0,5.6843418860808015E-14},height_median=2.8421709430404007E-14,height_range={0.0,8.526512829121202E-14},length=23.371128688883466,length_95%_HPD={13.065459262056805,34.804185498313814},length_median=23.023454055108772,length_range={9.489484524167295,43.02771849212357},rate=0.0022742638402548456,rate_95%_HPD={0.0012737285155773738,0.0033268062059150613},rate_median=0.0021904895149214235,rate_range={0.0011596925823313259,0.00564255525225896}]:23.37112868888347,101[&height=2.3302165208733874E-14,height_95%_HPD={0.0,5.6843418860808015E-14},height_median=2.8421709430404007E-14,height_range={0.0,8.526512829121202E-14},length=23.371128688883466,length_95%_HPD={13.065459262056805,34.804185498313814},length_median=23.023454055108772,length_range={9.489484524167295,43.02771849212357},rate=0.0013548577285434232,rate_95%_HPD={7.763953306958122E-4,0.002020486284409978},rate_median=0.001302636686454033,rate_range={6.492687908862362E-4,0.003204655014068163}]:23.37112868888347)[&height=23.371128688883495,height_95%_HPD={13.065459262056805,34.80418549831387},height_median=23.0234540551088,height_range={9.489484524167324,43.02771849212357},length=6.866180591429244,length_95%_HPD={1.2117809741971826,13.715416647220948},length_median=6.296003565780268,length_range={0.794485743212789,21.41034890319426},posterior=1.0,rate=0.0015022882993035714,rate_95%_HPD={4.042777995845282E-4,0.0032691284132197692},rate_median=0.0012357600062378638,rate_range={3.0105814768179473E-4,0.00916215194797494}]:6.866180591429238)[&height=30.237309280312733,height_95%_HPD={20.865202349933327,39.680332309941804},height_median=29.992612939495572,height_range={18.69333469082906,46.30534643758966},length=3.6166218913470485,length_95%_HPD={0.41755290819166646,7.882425659888668},length_median=3.1987572297571987,length_range={0.24796209940006975,12.909660450165703},posterior=1.0,rate=0.0019365476771367768,rate_95%_HPD={3.663919022623126E-4,0.0046618543223522395},rate_median=0.0015500269373285261,rate_range={3.663919022623126E-4,0.01519282629789696}]:3.6166218913470125,37[&height=2.3447057969724538E-14,height_95%_HPD={0.0,5.6843418860808015E-14},height_median=2.8421709430404007E-14,height_range={0.0,8.526512829121202E-14},length=33.85393117165972,length_95%_HPD={24.93745730148487,43.17031855326715},length_median=33.6682250147133,length_range={20.948461042992136,49.41599292768032},rate=0.001513518762177172,rate_95%_HPD={0.0011003985358467863,0.0019366561466234548},rate_median=0.0014951304706407992,rate_range={9.800633180713423E-4,0.002366316620514125}]:33.853931171659724)[&height=33.853931171659745,height_95%_HPD={24.937457301484898,43.17031855326718},height_median=33.66822501471333,height_range={20.948461042992164,49.41599292768035},length=14.655259624800202,length_95%_HPD={5.53397122171458,23.7200559845612},length_median=14.271588316036343,length_range={4.8304317522948566,31.862016308094155},posterior=1.0,rate=0.004041918475240831,rate_95%_HPD={0.001789743327069468,0.007272201448049482},rate_median=0.0036764101335315796,rate_range={0.0016562337066281776,0.010599106362744802}]:14.655259624800223,(((((((3[&height=2.3094769688100177E-14,height_95%_HPD={0.0,5.6843418860808015E-14},height_median=2.8421709430404007E-14,height_range={0.0,8.526512829121202E-14},length=7.4017149862541824,length_95%_HPD={3.0869316520955863,12.098950956259273},length_median=7.215243190618494,length_range={1.3362567717132947,16.398762469161866},rate=0.001812361637555648,rate_95%_HPD={7.936190846926398E-4,0.0032287522290823253},rate_median=0.0016314702267939333,rate_range={5.756018091714018E-4,0.009094276088052116}]:7.4017149862541824,36[&height=2.3094769688100177E-14,height_95%_HPD={0.0,5.6843418860808015E-14},height_median=2.8421709430404007E-14,height_range={0.0,8.526512829121202E-14},length=7.4017149862541824,length_95%_HPD={3.0869316520955863,12.098950956259273},length_median=7.215243190618494,length_range={1.3362567717132947,16.398762469161866},rate=0.0032758798896419116,rate_95%_HPD={0.0014382691829629547,0.005793832520781406},rate_median=0.002954907642619337,rate_range={0.0012204329806694606,0.015116517817996071}]:7.4017149862541824)[&height=7.4017149862542055,height_95%_HPD={3.0869316520955863,12.098950956259273},height_median=7.215243190618523,height_range={1.3362567717132947,16.398762469161895},length=10.453483934725432,length_95%_HPD={3.7812463032305175,17.292875069929494},length_median=10.146774883433466,length_range={3.576156556454478,24.236160588960857},posterior=1.0,rate=0.005566229966682823,rate_95%_HPD={0.0022935812160274145,0.01034218772417173},rate_median=0.004984763737347587,rate_range={0.002033584323033674,0.014436982623571664}]:10.42700308625329,6[&height=2.2964082099755656E-14,height_95%_HPD={0.0,5.6843418860808015E-14},height_median=2.8421709430404007E-14,height_range={0.0,8.526512829121202E-14},length=17.813108196800236,length_95%_HPD={11.320038781192295,25.305469402011994},length_median=17.537796485595663,length_range={7.633170096738638,30.48075166889099},rate=0.0020446949217368744,rate_95%_HPD={0.0012704563460421902,0.0029105821098077394},rate_median=0.001983070369154209,rate_range={0.0010697784285570403,0.004299404965244018}]:17.828718072507474)[&height=17.828718072507495,height_95%_HPD={11.293679056122443,25.305469402012022},height_median=17.55000578218263,height_range={7.633170096738667,30.480751668891017},length=3.586917583912205,length_95%_HPD={0.4633412893238926,7.567578532366554},length_median=3.140098015881989,length_range={0.37772200862163174,15.235885127475413},posterior=0.9852059176329468,rate=0.0020920912981057882,rate_95%_HPD={4.272999342467729E-4,0.004572134893755641},rate_median=0.0017559558663950115,rate_range={3.5862872882193883E-4,0.014041898615568538}]:3.5603332782040766,14[&height=2.3106133826217092E-14,height_95%_HPD={0.0,5.6843418860808015E-14},height_median=2.8421709430404007E-14,height_range={0.0,8.526512829121202E-14},length=21.346960626532123,length_95%_HPD={14.65189679725988,28.70860730128919},length_median=21.13654084020633,length_range={12.192888983436944,33.271365667780486},rate=0.0016830717606597498,rate_95%_HPD={0.0011303253571662909,0.0023058856016826773},rate_median=0.0016475124267183738,rate_range={0.0010230817205708904,0.002927639374067626}]:21.389051350711547)[&height=21.389051350711572,height_95%_HPD={14.771923787632772,28.716089663814415},height_median=21.184823776243412,height_range={12.834777358869346,33.271365667780515},length=6.766222849069853,length_95%_HPD={1.711124314257873,12.434922737461505},length_median=6.282328548894128,length_range={1.5155149225197135,22.622911583469318},posterior=1.0,rate=0.002722639527763863,rate_95%_HPD={7.504565645481524E-4,0.005340418633889557},rate_median=0.0023930553350047217,rate_range={6.265339393738995E-4,0.011024673336114916}]:6.673564443870255,(30[&height=2.3222616241915468E-14,height_95%_HPD={0.0,5.6843418860808015E-14},height_median=2.8421709430404007E-14,height_range={0.0,8.526512829121202E-14},length=5.916740646785178,length_95%_HPD={1.623358179677325,11.530665956353516},length_median=5.39776651326882,length_range={0.8914653240710209,21.6908897530964},rate=0.0017191709153417255,rate_95%_HPD={4.695635186076731E-4,0.003445198581379136},rate_median=0.001509623608752511,rate_range={3.73291825933448E-4,0.009110099200590287}]:5.916740646785181,31[&height=2.3222616241915468E-14,height_95%_HPD={0.0,5.6843418860808015E-14},height_median=2.8421709430404007E-14,height_range={0.0,8.526512829121202E-14},length=5.916740646785178,length_95%_HPD={1.623358179677325,11.530665956353516},length_median=5.39776651326882,length_range={0.8914653240710209,21.6908897530964},rate=0.0014832828709392687,rate_95%_HPD={3.783444966355838E-4,0.0029666011642891163},rate_median=0.0013045205224919772,rate_range={3.230453817312905E-4,0.00802026953286345}]:5.916740646785181)[&height=5.916740646785204,height_95%_HPD={1.6233581796773535,11.530665956353516},height_median=5.397766513268849,height_range={0.8914653240710493,21.6908897530964},length=22.6675161881121,length_95%_HPD={13.45367261550652,32.66876505081379},length_median=22.329667562517542,length_range={7.769895044975314,40.113004385181085},posterior=1.0,rate=0.0013441634691791185,rate_95%_HPD={7.891665918245697E-4,0.0019240875012815802},rate_median=0.0013018050082428008,rate_range={6.718549584042307E-4,0.0034766131614228694}]:22.145875147796623)[&height=28.062615794581827,height_95%_HPD={20.812748288570617,36.81404930181532},height_median=27.829006618310274,height_range={17.428466774006097,41.651281378398224},length=2.3288285009147005,length_95%_HPD={0.283935830151961,5.389920034912933},length_median=1.947278339404754,length_range={0.09178484995454994,11.973782995089238},posterior=0.7309076369452219,rate=0.0017145774895975798,rate_95%_HPD={3.3923008689148044E-4,0.00391416649343159},rate_median=0.0014126449123211767,rate_range={2.534533310082357E-4,0.013815225182930846}]:2.1438311509560215,(10[&height=2.3191364862093953E-14,height_95%_HPD={0.0,5.6843418860808015E-14},height_median=2.8421709430404007E-14,height_range={0.0,8.526512829121202E-14},length=16.08788514823818,length_95%_HPD={8.424502331913416,25.05202993839943},length_median=15.775913753400566,length_range={5.631428315751322,33.23862473192162},rate=0.002109087787805599,rate_95%_HPD={0.001051924381000486,0.0034823340837862047},rate_median=0.0020041901318713327,rate_range={6.463511595363117E-4,0.006778019283195246}]:16.08788514823818,12[&height=2.3191364862093953E-14,height_95%_HPD={0.0,5.6843418860808015E-14},height_median=2.8421709430404007E-14,height_range={0.0,8.526512829121202E-14},length=16.08788514823818,length_95%_HPD={8.424502331913416,25.05202993839943},length_median=15.775913753400566,length_range={5.631428315751322,33.23862473192162},rate=0.004352672646919096,rate_95%_HPD={0.002282415836186752,0.007005107377190093},rate_median=0.0041475519798737795,rate_range={0.001979090034910006,0.010599106362744802}]:16.08788514823818)[&height=16.087885148238204,height_95%_HPD={8.424502331913445,25.052029938399457},height_median=15.77591375340058,height_range={5.63142831575135,33.23862473192165},length=13.526902966817772,length_95%_HPD={5.6703539426807055,22.406022524371508},length_median=13.248730564990481,length_range={3.6341584589225704,31.08979649520998},posterior=1.0,rate=0.0037452392465790684,rate_95%_HPD={0.0016215310489340316,0.006663801539549616},rate_median=0.003412654520598312,rate_range={0.0013279428067357974,0.011822715009352166}]:14.118561797299645)[&height=30.20644694553785,height_95%_HPD={22.7311171614148,38.59802383695619},height_median=30.059101786110915,height_range={19.349138998089686,43.18436947300748},length=2.1780595589739766,length_95%_HPD={0.20468897993444557,4.934834507820156},length_median=1.8538497544676886,length_range={0.056081567885613026,15.43995357769731},posterior=0.9074370251899241,rate=0.001973555694129609,rate_95%_HPD={3.3367984337954245E-4,0.004465400709072737},rate_median=0.0016087849593782245,rate_range={2.651965618113262E-4,0.013186664097163115}]:2.1078301159355703,34[&height=2.3259549690795444E-14,height_95%_HPD={0.0,5.6843418860808015E-14},height_median=2.8421709430404007E-14,height_range={0.0,8.526512829121202E-14},length=32.125601534528364,length_95%_HPD={23.76048010159147,40.45832538748466},length_median=32.012719807492516,length_range={18.971462404780638,45.79108301946246},rate=9.709114235724337E-4,rate_95%_HPD={7.062477072875156E-4,0.0012535338991698573},rate_median=9.555663176212441E-4,rate_range={6.105723316387991E-4,0.0015856105976763744}]:32.3142770614734)[&height=32.31427706147342,height_95%_HPD={24.560427206713058,41.04211543513759},height_median=32.13980809012709,height_range={20.316387778177173,45.79108301946252},length=3.6237003585961722,length_95%_HPD={0.5329038829477497,7.771168458624757},length_median=3.1681503619563927,length_range={0.26422348053961286,13.982545123190917},posterior=0.9988004798080767,rate=0.0020917764035368797,rate_95%_HPD={4.4502036263381975E-4,0.004545914148996782},rate_median=0.0017378827396186407,rate_range={3.2403268075774323E-4,0.013944314811324382}]:3.6265379559246966,35[&height=2.328796003608773E-14,height_95%_HPD={0.0,5.6843418860808015E-14},height_median=2.8421709430404007E-14,height_range={0.0,5.6843418860808015E-14},length=35.93626648573089,length_95%_HPD={27.778423594858538,45.1020528909811},length_median=35.629889031311365,length_range={23.633355914982914,50.273544500269},rate=0.0014246510711104867,rate_95%_HPD={0.0010566879210403468,0.0017891898369038432},rate_median=0.0014109532440932798,rate_range={9.416192056160157E-4,0.0021191240492442233}]:35.940815017398094)[&height=35.940815017398116,height_95%_HPD={27.778423594858538,45.10205289098113},height_median=35.64712013204134,height_range={23.633355914982943,50.273544500269026},length=12.56837577906193,length_95%_HPD={3.9521973552354552,20.751067158163096},length_median=11.977733084115677,length_range={3.5278836671260336,31.469104163157297},posterior=1.0,rate=0.004412252444338052,rate_95%_HPD={0.0017782339018985092,0.008156800473189424},rate_median=0.004005183065245626,rate_range={0.001511407173947903,0.01280576842606508}]:12.568375779061853)[&height=48.50919079645997,height_95%_HPD={39.65134761333658,57.92935700077922},height_median=48.5599972873775,height_range={34.614875289561695,62.07972657476597},length=7.621933683051709,length_95%_HPD={2.50789906941651,13.429640056979082},length_median=7.195745548082471,length_range={1.6947617395810965,23.03394473222994},posterior=1.0,rate=0.004400642524339658,rate_95%_HPD={0.0015895002311098283,0.00826706725006605},rate_median=0.003955766446110823,rate_range={0.0011078697430418564,0.014603296592966488}]:7.6219336830515445,(8[&height=2.328227796702927E-14,height_95%_HPD={0.0,5.6843418860808015E-14},height_median=2.8421709430404007E-14,height_range={0.0,5.6843418860808015E-14},length=39.26710343439159,length_95%_HPD={28.896526595953247,52.4598045600809},length_median=38.638013375171745,length_range={21.341789887543285,56.955254633881694},rate=0.0022906059359250456,rate_95%_HPD={0.001571901629208128,0.0030101242989720154},rate_median=0.0022667731175463695,rate_range={0.0013152422133828744,0.004731539152551729}]:39.26710343439159,(((16[&height=2.3532289005601398E-14,height_95%_HPD={0.0,5.6843418860808015E-14},height_median=2.8421709430404007E-14,height_range={0.0,5.6843418860808015E-14},length=17.759340354845015,length_95%_HPD={8.770495915316602,28.263805686557916},length_median=17.07827972166816,length_range={4.7034558369945785,36.25437359902038},rate=0.0035000423468362187,rate_95%_HPD={0.001676146756665272,0.005554373358714843},rate_median=0.0033124417723589105,rate_range={0.0015757337049119495,0.01183079772820435}]:17.75934035484501,29[&height=2.3532289005601398E-14,height_95%_HPD={0.0,5.6843418860808015E-14},height_median=2.8421709430404007E-14,height_range={0.0,5.6843418860808015E-14},length=17.759340354845015,length_95%_HPD={8.770495915316602,28.263805686557916},length_median=17.07827972166816,length_range={4.7034558369945785,36.25437359902038},rate=0.0034823391641887877,rate_95%_HPD={0.0017600161674457043,0.005613831644696033},rate_median=0.0032817738481855,rate_range={0.001611278986134602,0.011817867206422155}]:17.75934035484501)[&height=17.759340354845033,height_95%_HPD={8.77049591531663,28.263805686557916},height_median=17.078279721668174,height_range={4.703455836994607,36.25437359902041},length=5.155443817125273,length_95%_HPD={0.8638347405207867,10.934999325379565},length_median=4.518740127203976,length_range={0.5380903802956425,17.933515761805324},posterior=1.0,rate=0.0021499404935350916,rate_95%_HPD={4.568664245424722E-4,0.004925983506307195},rate_median=0.001792985276613519,rate_range={3.263613145794777E-4,0.011202071228924084}]:5.155443817125285,76[&height=2.3387396244610735E-14,height_95%_HPD={0.0,5.6843418860808015E-14},height_median=2.8421709430404007E-14,height_range={0.0,5.6843418860808015E-14},length=22.914784171970304,length_95%_HPD={12.755301465136426,33.55458788279873},length_median=22.38962199621048,length_range={8.152317438532549,43.54292042096171},rate=0.0016214430166555573,rate_95%_HPD={9.044541641854852E-4,0.002427564984361322},rate_median=0.0015607421879673417,rate_range={7.576617331499907E-4,0.004264444680331071}]:22.914784171970293)[&height=22.914784171970318,height_95%_HPD={12.755301465136426,33.55458788279876},height_median=22.389621996210494,height_range={8.152317438532549,43.54292042096171},length=6.057126572039447,length_95%_HPD={1.1916792366613578,12.079647459604843},length_median=5.489548043593793,length_range={0.6062463276339827,26.418718228873885},posterior=1.0,rate=0.0022774044114922034,rate_95%_HPD={5.279368755062655E-4,0.005082744810987847},rate_median=0.0018961948480614114,rate_range={4.0170279471545147E-4,0.013866066140449605}]:6.057126572039458,24[&height=2.329080107061696E-14,height_95%_HPD={0.0,5.6843418860808015E-14},height_median=2.8421709430404007E-14,height_range={0.0,5.6843418860808015E-14},length=28.971910744009747,length_95%_HPD={17.84122969263126,39.883495712984484},length_median=28.51989410276736,length_range={14.813095335325343,46.682929718569966},rate=0.001616783700051013,rate_95%_HPD={0.0010324455513712949,0.0023069381248200264},rate_median=0.0015732451897920298,rate_range={8.840619484731063E-4,0.003089670624927269}]:28.97191074400975)[&height=28.971910744009776,height_95%_HPD={17.841229692631288,39.88349571298451},height_median=28.51989410276736,height_range={14.813095335325372,46.682929718569994},length=10.295192690381691,length_95%_HPD={2.483219256781169,18.470489390951613},length_median=9.505438174928571,length_range={1.4880695947209546,39.35164086244234},posterior=1.0,rate=0.0025863151080382563,rate_95%_HPD={5.790846029135124E-4,0.005142400527534422},rate_median=0.0022641597207485728,rate_range={5.197025913822732E-4,0.012357060020284867}]:10.295192690381839)[&height=39.267103434391615,height_95%_HPD={28.896526595953276,52.459804560080926},height_median=38.638013375171774,height_range={21.341789887543314,56.95525463388172},length=16.86402104512019,length_95%_HPD={6.456534237775898,28.465921988096042},length_median=16.518899370618612,length_range={3.4428294314765964,35.15436630447353},posterior=1.0,rate=0.003334673710675969,rate_95%_HPD={0.0014176542968515349,0.006188835211116403},rate_median=0.002971962889812878,rate_range={0.00139993881640009,0.01290101962178606}]:16.864021045119898)[&height=56.13112447951151,height_95%_HPD={47.19085523804925,66.20365614062925},height_median=55.93581145563923,height_range={41.40218795867342,72.46803911480318},length=3.250946695230356,length_95%_HPD={0.7028449257058611,6.61843887901496},length_median=2.887594198276247,length_range={0.4475865600210085,16.265286098367042},posterior=1.0,rate=0.002696193524219123,rate_95%_HPD={6.585874540909751E-4,0.005634433496066992},rate_median=0.002338701008891257,rate_range={3.6582726063235987E-4,0.014630772561188657}]:3.250946695230546,(20[&height=2.298396934146026E-14,height_95%_HPD={0.0,2.8421709430404007E-14},height_median=2.8421709430404007E-14,height_range={0.0,5.6843418860808015E-14},length=20.46335653763699,length_95%_HPD={7.790487219873938,32.92774704105952},length_median=20.063262309653922,length_range={5.052832307284646,49.560945682096275},rate=0.002988255559352375,rate_95%_HPD={0.0012955995756857906,0.005434197688567803},rate_median=0.002664493627744827,rate_range={0.0010209474910638785,0.010049185232213445}]:20.463356537636987,53[&height=2.298396934146026E-14,height_95%_HPD={0.0,2.8421709430404007E-14},height_median=2.8421709430404007E-14,height_range={0.0,5.6843418860808015E-14},length=20.46335653763699,length_95%_HPD={7.790487219873938,32.92774704105952},length_median=20.063262309653922,length_range={5.052832307284646,49.560945682096275},rate=0.0012791887727074756,rate_95%_HPD={5.147733720787635E-4,0.0022863284108716178},rate_median=0.0011385328254238797,rate_range={4.3801954202228447E-4,0.004793176836242908}]:20.463356537636987)[&height=20.463356537637008,height_95%_HPD={7.790487219873967,32.92774704105955},height_median=20.06326230965395,height_range={5.052832307284675,49.5609456820963},length=38.91871463710503,length_95%_HPD={22.539571156963547,54.55682526414631},length_median=39.808126299596545,length_range={9.787411498956146,61.89466317882672},posterior=1.0,rate=0.002424519951037782,rate_95%_HPD={0.0015130435822981556,0.003691430718354703},rate_median=0.0022315067129104514,rate_range={0.0013908459507330285,0.008845883824452264}]:38.918714637105055)[&height=59.38207117474206,height_95%_HPD={50.1709561409524,69.56236038320938},height_median=59.13654147425921,height_range={44.65941596311512,74.36224024221687},length=3.160759964647203,length_95%_HPD={0.6335746964841746,6.226506777475791},length_median=2.8893256917753902,length_range={0.41976635106836113,13.623069097347823},posterior=1.0,rate=0.002566079627315367,rate_95%_HPD={6.241591397715998E-4,0.005406795921832162},rate_median=0.0022199670373026902,rate_range={4.749334207786687E-4,0.012457330284592685}]:3.160759964647198,(7[&height=2.333341658855539E-14,height_95%_HPD={0.0,2.8421709430404007E-14},height_median=2.8421709430404007E-14,height_range={0.0,5.6843418860808015E-14},length=45.79121015457568,length_95%_HPD={29.519895629079002,61.291287234331094},length_median=46.25965525971163,length_range={17.681552040474088,69.28000369298978},rate=0.0018954050959856987,rate_95%_HPD={0.001312835876056063,0.0027054165612952047},rate_median=0.0018017117350382882,rate_range={0.0012020884483299155,0.004848397800381566}]:45.79121015457569,70[&height=2.333341658855539E-14,height_95%_HPD={0.0,2.8421709430404007E-14},height_median=2.8421709430404007E-14,height_range={0.0,5.6843418860808015E-14},length=45.79121015457568,length_95%_HPD={29.519895629079002,61.291287234331094},length_median=46.25965525971163,length_range={17.681552040474088,69.28000369298978},rate=0.0012574087642823672,rate_95%_HPD={8.233744598903008E-4,0.0017806887792316778},rate_median=0.0011994169936176643,rate_range={7.274874819753457E-4,0.003100264821257683}]:45.79121015457569)[&height=45.79121015457571,height_95%_HPD={29.51989562907903,61.29128723433112},height_median=46.25965525971166,height_range={17.681552040474116,69.28000369298981},length=16.75162098481356,length_95%_HPD={2.7703157196424115,33.15163285282138},length_median=15.728056790800977,length_range={0.9528120607405697,48.11456467298629},posterior=1.0,rate=0.001220018655471687,rate_95%_HPD={3.2069562545882337E-4,0.0029232433005684007},rate_median=9.261566549380069E-4,rate_range={2.7876484584249167E-4,0.01396094765496798}]:16.75162098481355)[&height=62.54283113938926,height_95%_HPD={54.02214736198212,73.29939876352056},height_median=62.31173096023076,height_range={49.86057037155807,77.49575523513975},length=7.562511832176849,length_95%_HPD={3.2067736964650777,12.581668747024295},length_median=7.208383319122426,length_range={2.4319378624400088,17.374494014648064},posterior=1.0,rate=0.00543153307248331,rate_95%_HPD={0.0024596628552101185,0.00931944291726565},rate_median=0.005108744975566754,rate_range={0.0019919004912983115,0.014393471710578584}]:7.56251183217686,59[&height=2.324250348362007E-14,height_95%_HPD={0.0,2.8421709430404007E-14},height_median=2.8421709430404007E-14,height_range={0.0,5.6843418860808015E-14},length=70.10534297156607,length_95%_HPD={61.32416139985018,80.45768438709773},length_median=69.5745834730638,length_range={56.566285410171645,90.62865067503469},rate=7.896623443342611E-4,rate_95%_HPD={6.646117008272726E-4,9.105041601732729E-4},rate_median=7.899054180341327E-4,rate_range={5.809162937471757E-4,0.0010808320290968056}]:70.10534297156609)[&height=70.10534297156612,height_95%_HPD={61.32416139985018,80.45768438709776},height_median=69.5745834730638,height_range={56.56628541017167,90.62865067503472},length=5.0389494017942615,length_95%_HPD={1.2415181474958814,9.488384236337865},length_median=4.646296469558507,length_range={1.1118837307739113,16.022919627910497},posterior=1.0,rate=0.0032831175101494586,rate_95%_HPD={0.0010911334270087341,0.006645008045099845},rate_median=0.002861602892413838,rate_range={7.608677881074987E-4,0.012256579178784992}]:5.0389494017942695,(19[&height=2.326807279438313E-14,height_95%_HPD={0.0,2.8421709430404007E-14},height_median=2.8421709430404007E-14,height_range={0.0,5.6843418860808015E-14},length=39.51453857113145,length_95%_HPD={7.027387820938742,65.76523282170807},length_median=39.62662151471945,length_range={5.3735199635343065,77.57452739967218},rate=0.0017488200231238061,rate_95%_HPD={6.687614754036785E-4,0.0044215969240149235},rate_median=0.0013363918141917996,rate_range={6.687614754036785E-4,0.009475373809521839}]:39.514538571131446,69[&height=2.326807279438313E-14,height_95%_HPD={0.0,2.8421709430404007E-14},height_median=2.8421709430404007E-14,height_range={0.0,5.6843418860808015E-14},length=39.51453857113145,length_95%_HPD={7.027387820938742,65.76523282170807},length_median=39.62662151471945,length_range={5.3735199635343065,77.57452739967218},rate=0.001125109836602416,rate_95%_HPD={4.2812547859166463E-4,0.00291951980494742},rate_median=8.544600983099498E-4,rate_range={4.0871606760649527E-4,0.0062018777733905805}]:39.514538571131446)[&height=39.51453857113147,height_95%_HPD={7.02738782093877,65.7652328217081},height_median=39.62662151471947,height_range={5.373519963534321,77.5745273996722},length=35.62975380222905,length_95%_HPD={9.548489421083872,64.25780654057469},length_median=35.669565143578026,length_range={3.4655751557839807,75.17148042425106},posterior=1.0,rate=9.509701239502466E-4,rate_95%_HPD={3.393338892694298E-4,0.0021223617686915524},rate_median=7.345796227737149E-4,rate_range={3.0453812196417056E-4,0.007591480904405883}]:35.62975380222892)[&height=75.14429237336039,height_95%_HPD={66.95571110433437,85.97414432542715},height_median=74.66563199498802,height_range={62.552534821571726,96.81743451874239},length=4.427636693614319,length_95%_HPD={0.6547072279564503,8.839682231229702},length_median=4.0437524531123685,length_range={0.6082729761173624,15.20375589728637},posterior=1.0,rate=0.0026577284328633713,rate_95%_HPD={5.895643297924552E-4,0.005932717949635454},rate_median=0.0021745203457091177,rate_range={5.389848664056969E-4,0.013888389061871075}]:4.427636693614048)[&height=79.57192906697443,height_95%_HPD={71.76188757215476,88.73383374712186},height_median=78.78545931145175,height_range={69.79856801613172,101.18430803106408},length=2.9026355087152056,length_95%_HPD={0.5335194524238887,6.328107360876473},length_median=2.518446619847346,length_range={0.3453372003757238,11.304810564431577},posterior=1.0,rate=0.0022711275969911657,rate_95%_HPD={4.887465944829012E-4,0.004892040131568985},rate_median=0.0019405510408188492,rate_range={3.552258314581359E-4,0.011683800476608046}]:2.902635508715022,28[&height=2.3245344518149298E-14,height_95%_HPD={0.0,2.8421709430404007E-14},height_median=2.8421709430404007E-14,height_range={0.0,5.6843418860808015E-14},length=82.47456457568944,length_95%_HPD={73.92193420745025,91.88745018536494},length_median=81.83143878695222,length_range={71.36696649188337,101.79729886202671},rate=5.430513480585127E-4,rate_95%_HPD={4.6283272625023605E-4,6.235180644896168E-4},rate_median=5.436467708766187E-4,rate_range={4.165112928896448E-4,7.042338106363295E-4}]:82.47456457568943)[&height=82.47456457568946,height_95%_HPD={73.92193420745028,91.88745018536497},height_median=81.83143878695225,height_range={71.36696649188337,101.79729886202671},length=5.5686943348739995,length_95%_HPD={1.0316177560132616,11.057199099052426},length_median=5.037224829970533,length_range={0.8077504562423741,17.997094196368025},posterior=1.0,rate=0.0029321155944590904,rate_95%_HPD={8.688490838397564E-4,0.005960085604974301},rate_median=0.0025231308575514857,rate_range={7.007681341172038E-4,0.014321292120422213}]:5.568694334874294,18[&height=2.3245344518149298E-14,height_95%_HPD={0.0,2.8421709430404007E-14},height_median=2.8421709430404007E-14,height_range={0.0,5.6843418860808015E-14},length=88.04325891056372,length_95%_HPD={78.24382389465681,98.06392052142989},length_median=87.82571093931406,length_range={73.74371713596791,105.86490093930193},rate=5.176220433246615E-4,rate_95%_HPD={4.3507408070678915E-4,5.97149225958824E-4},rate_median=5.167230307819186E-4,rate_range={3.827254922959703E-4,6.699855581756236E-4}]:88.04325891056372)[&height=88.04325891056375,height_95%_HPD={78.24382389465684,98.06392052142992},height_median=87.82571093931409,height_range={73.74371713596794,105.86490093930193},length=9.003737019411936,length_95%_HPD={2.7974560773399304,14.714681644882972},length_median=8.863434434832193,length_range={2.3764365939186263,20.702671112740504},posterior=1.0,rate=0.0040612014277178,rate_95%_HPD={0.0016050755046156647,0.00811525456245058},rate_median=0.0035139639071248246,rate_range={0.0015377744937056853,0.012040568805445292}]:9.439814078176838,(((((13[&height=2.326239072532467E-14,height_95%_HPD={0.0,2.8421709430404007E-14},height_median=2.8421709430404007E-14,height_range={0.0,5.6843418860808015E-14},length=26.30496294587161,length_95%_HPD={8.239995675194166,48.68446745119488},length_median=24.2431212442358,length_range={6.714390762310643,59.480012598689854},rate=0.0012184151069674076,rate_95%_HPD={4.464911357213844E-4,0.002393281397610302},rate_median=0.0010870973140203121,rate_range={3.883906882369368E-4,0.004407809961101103}]:26.304962945871605,98[&height=2.326239072532467E-14,height_95%_HPD={0.0,2.8421709430404007E-14},height_median=2.8421709430404007E-14,height_range={0.0,5.6843418860808015E-14},length=26.30496294587161,length_95%_HPD={8.239995675194166,48.68446745119488},length_median=24.2431212442358,length_range={6.714390762310643,59.480012598689854},rate=0.0019226072358481416,rate_95%_HPD={7.428521815925299E-4,0.003726447165872271},rate_median=0.0017202918997588981,rate_range={6.806450219059004E-4,0.006504388454734151}]:26.304962945871605)[&height=26.30496294587163,height_95%_HPD={8.239995675194194,48.68446745119488},height_median=24.24312124423583,height_range={6.714390762310671,59.480012598689854},length=36.96098656247811,length_95%_HPD={14.058732845369661,56.41103576616193},length_median=38.257774904469606,length_range={7.882471443711495,63.391143432330324},posterior=1.0,rate=0.0010951887273342108,rate_95%_HPD={5.580822316840986E-4,0.002240754575420651},rate_median=9.129628168382117E-4,rate_range={5.064951753456392E-4,0.004293299861223495}]:36.955664230706276,65[&height=2.3245344518149298E-14,height_95%_HPD={0.0,2.8421709430404007E-14},height_median=2.8421709430404007E-14,height_range={0.0,5.6843418860808015E-14},length=63.26849344071224,length_95%_HPD={52.57884586127096,71.98765294814294},length_median=63.878061761315266,length_range={42.627200265239594,78.68181723819767},rate=0.0011211844023513856,rate_95%_HPD={9.52999719452333E-4,0.0013200024480348866},rate_median=0.0011065541684556921,rate_range={8.380693280931635E-4,0.0016457264736559067}]:63.260627176577884)[&height=63.260627176577906,height_95%_HPD={52.57884586127096,71.95178180045228},height_median=63.874016537060584,height_range={42.62720026523962,78.68181723819767},length=5.550053358032174,length_95%_HPD={0.5589638012097282,13.731926577702183},length_median=4.3636690629250765,length_range={0.43700388390628575,25.953156132990856},posterior=0.9988004798080767,rate=0.0011789933991302596,rate_95%_HPD={1.8257462149565197E-4,0.0029491984588594714},rate_median=9.054359067114876E-4,rate_range={1.7948249847282526E-4,0.009493036660501447}]:5.551262221097495,((15[&height=2.3171477620389353E-14,height_95%_HPD={0.0,2.8421709430404007E-14},height_median=2.8421709430404007E-14,height_range={0.0,5.6843418860808015E-14},length=21.233429386650798,length_95%_HPD={8.489319257324112,36.952585547836236},length_median=19.83508415525992,length_range={5.852315046126108,59.34935524492613},rate=0.0013394457800740103,rate_95%_HPD={5.268353892901922E-4,0.0023484695372437236},rate_median=0.0012411485079964202,rate_range={3.699710528745952E-4,0.0039444917029097335}]:21.233429386650798,85[&height=2.3171477620389353E-14,height_95%_HPD={0.0,2.8421709430404007E-14},height_median=2.8421709430404007E-14,height_range={0.0,5.6843418860808015E-14},length=21.233429386650798,length_95%_HPD={8.489319257324112,36.952585547836236},length_median=19.83508415525992,length_range={5.852315046126108,59.34935524492613},rate=0.0012507313106876637,rate_95%_HPD={4.79864549270055E-4,0.0022066219274024528},rate_median=0.0011538238930372691,rate_range={3.673887138809542E-4,0.0040665961046321315}]:21.233429386650798)[&height=21.233429386650823,height_95%_HPD={8.48931925732414,36.952585547836264},height_median=19.83508415525995,height_range={5.852315046126108,59.34935524492616},length=44.17034448067191,length_95%_HPD={26.43897099344771,59.03218297314653},length_median=45.678176881205474,length_range={12.493872973257751,65.19197793995619},posterior=1.0,rate=7.109652608631911E-4,rate_95%_HPD={4.541578721945107E-4,0.0010640623137302243},rate_median=6.533047249986049E-4,rate_range={4.2302895367919935E-4,0.002308048692913843}]:44.17034448067167,(60[&height=2.321977520738624E-14,height_95%_HPD={0.0,2.8421709430404007E-14},height_median=2.8421709430404007E-14,height_range={0.0,8.526512829121202E-14},length=60.97829419577989,length_95%_HPD={50.86098822896007,70.64189135328671},length_median=61.65024098633591,length_range={35.22759336581328,76.61077410021586},rate=0.0010956841413116212,rate_95%_HPD={9.169542861358564E-4,0.0012940008080418962},rate_median=0.0010776753585350534,rate_range={8.457406331845324E-4,0.0018228211398706256}]:60.97829419577987,62[&height=2.321977520738624E-14,height_95%_HPD={0.0,2.8421709430404007E-14},height_median=2.8421709430404007E-14,height_range={0.0,8.526512829121202E-14},length=60.97829419577989,length_95%_HPD={50.86098822896007,70.64189135328671},length_median=61.65024098633591,length_range={35.22759336581328,76.61077410021586},rate=7.855219897964743E-4,rate_95%_HPD={6.577130349742947E-4,9.440293398333999E-4},rate_median=7.741646835623948E-4,rate_range={5.721835579515337E-4,0.001407352312809716}]:60.97829419577987)[&height=60.97829419577989,height_95%_HPD={50.860988228960096,70.64189135328674},height_median=61.65024098633594,height_range={35.227593365813306,76.61077410021589},length=4.425479671542591,length_95%_HPD={0.379757895757848,11.65439875380693},length_median=3.4587111559863217,length_range={0.23256830209814439,24.894126561075296},posterior=1.0,rate=0.0013654373594951706,rate_95%_HPD={2.271958281440661E-4,0.003352546254924978},rate_median=0.0010729155306575078,rate_range={2.063138388705289E-4,0.011173283645009049}]:4.425479671542611)[&height=65.4037738673225,height_95%_HPD={58.781383504785595,73.35889810450925},height_median=65.29836361400369,height_range={53.28277493996242,80.21803782357327},length=3.405571597990052,length_95%_HPD={0.33560929346558055,8.45510173849111},length_median=2.715280013729867,length_range={0.2692317948680625,15.654419968154002},posterior=1.0,rate=0.0011951861732430033,rate_95%_HPD={2.271958281440661E-4,0.0028737857292088836},rate_median=9.476633398334188E-4,rate_range={2.1429316478011152E-4,0.009656470641504633}]:3.4081155303529016)[&height=68.8118893976754,height_95%_HPD={66.0005367216688,74.17855369087982},height_median=68.06676002212232,height_range={66.0005367216688,83.00746871076663},length=15.514707242231378,length_95%_HPD={7.407636279413495,26.0776797506432},length_median=14.853520675999597,length_range={5.083787789239537,30.46100414848499},posterior=1.0,rate=0.0033460158075078656,rate_95%_HPD={0.0015770895390143584,0.0055255969542928945},rate_median=0.003132775330906629,rate_range={0.0014909509384077688,0.008620995521535363}]:15.514707242231196,(((((((17[&height=2.3358985899318447E-14,height_95%_HPD={0.0,5.6843418860808015E-14},height_median=2.8421709430404007E-14,height_range={0.0,8.526512829121202E-14},length=11.463326140143812,length_95%_HPD={5.996631267660433,18.04207573912595},length_median=11.200828498143707,length_range={3.463728441317471,23.816812471344633},rate=0.00209937782588443,rate_95%_HPD={0.0010772823381752967,0.0034051736201656728},rate_median=0.0019918220484478107,rate_range={8.708144090750522E-4,0.006371182852331742}]:11.46332614014382,46[&height=2.3358985899318447E-14,height_95%_HPD={0.0,5.6843418860808015E-14},height_median=2.8421709430404007E-14,height_range={0.0,8.526512829121202E-14},length=11.463326140143812,length_95%_HPD={5.996631267660433,18.04207573912595},length_median=11.200828498143707,length_range={3.463728441317471,23.816812471344633},rate=0.001455333737089875,rate_95%_HPD={7.456687555985502E-4,0.0023395320969512765},rate_median=0.0013733611052862242,rate_range={6.44558678206499E-4,0.00380602915652755}]:11.46332614014382)[&height=11.463326140143844,height_95%_HPD={5.996631267660462,18.042075739125977},height_median=11.20082849814375,height_range={3.4637284413174996,23.81681247134466},length=3.6217336437440535,length_95%_HPD={0.5286816038396722,7.9813392968938786},length_median=3.120297174918676,length_range={0.25318153971700497,14.218370800420757},posterior=1.0,rate=0.0015118986178407595,rate_95%_HPD={2.7420996379804016E-4,0.0033423258890655488},rate_median=0.0012562713546209366,rate_range={2.1460076318303558E-4,0.009204012592111602}]:3.621733643744042,((80[&height=2.3259549690795444E-14,height_95%_HPD={0.0,5.6843418860808015E-14},height_median=2.8421709430404007E-14,height_range={0.0,8.526512829121202E-14},length=10.600492744987827,length_95%_HPD={5.670411581459746,16.54291007030311},length_median=10.293770784964046,length_range={3.9996189094400165,22.58028544401641},rate=0.001777638461881097,rate_95%_HPD={9.155867342344753E-4,0.0027698949738137533},rate_median=0.0017022847341483195,rate_range={7.510202484909235E-4,0.0042181565823131584}]:10.4975117316117,93[&height=2.3273754863441586E-14,height_95%_HPD={0.0,5.6843418860808015E-14},height_median=2.8421709430404007E-14,height_range={0.0,8.526512829121202E-14},length=10.523871937482404,length_95%_HPD={5.670411581459746,16.48354479561563},length_median=10.197864525850328,length_range={3.9996189094400165,22.58028544401641},rate=0.001273584448569379,rate_95%_HPD={6.621461405920491E-4,0.001964416025250756},rate_median=0.0012230621363579107,rate_range={5.379319927782334E-4,0.003356268489771031}]:10.4975117316117)[&height=10.497511731611723,height_95%_HPD={5.482634106267028,16.255875518127652},height_median=10.171200401034838,height_range={3.999618909440045,22.58028544401641},length=1.2243715039218452,length_95%_HPD={0.05614570161310439,3.0807386314517515},length_median=0.9700167590710862,length_range={0.05614570161310439,7.113174018458622},posterior=0.9150339864054379,rate=0.0014612003061283065,rate_95%_HPD={2.57499578982809E-4,0.003490844283267453},rate_median=0.0011728145085410004,rate_range={1.6385875118950832E-4,0.01367179220039888}]:1.2242765685182082,95[&height=2.3458422107841453E-14,height_95%_HPD={0.0,5.6843418860808015E-14},height_median=2.8421709430404007E-14,height_range={0.0,8.526512829121202E-14},length=11.643259458490078,length_95%_HPD={6.330851385559583,17.094578727620032},length_median=11.335619510138287,length_range={4.9691313337918075,25.479991061049432},rate=0.0015379250806556823,rate_95%_HPD={8.38257145538704E-4,0.0023077123192243453},rate_median=0.0014868676641621556,rate_range={6.138995455733114E-4,0.003192488488421339}]:11.721788300129909)[&height=11.721788300129932,height_95%_HPD={6.334333971900406,17.163171945194378},height_median=11.42510581405211,height_range={5.457817081310992,25.47999106104946},length=3.3632714837579476,length_95%_HPD={0.4113855819501424,7.287850342681537},length_median=2.9770849070051995,length_range={0.1990197952249133,12.033616355553335},posterior=1.0,rate=0.001406369696387475,rate_95%_HPD={2.777251578569565E-4,0.003028279056101133},rate_median=0.0011260258077947908,rate_range={2.241770686417136E-4,0.016011438142743542}]:3.363271483757954)[&height=15.085059783887885,height_95%_HPD={9.974074220003956,21.145922495352934},height_median=14.690656698561654,height_range={7.763502726857567,27.872746771764625},length=3.11631855510692,length_95%_HPD={0.376636100095439,6.745434318638587},length_median=2.6803180542582155,length_range={0.1531806474059323,13.34161266550987},posterior=1.0,rate=0.0013486059691803199,rate_95%_HPD={2.6812061223866894E-4,0.0030682945394717126},rate_median=0.0010946768593734914,rate_range={2.6812061223866894E-4,0.01242871667773186}]:3.116318555106904,((47[&height=2.315443141321398E-14,height_95%_HPD={0.0,5.6843418860808015E-14},height_median=2.8421709430404007E-14,height_range={0.0,8.526512829121202E-14},length=5.051160003753657,length_95%_HPD={1.975023777998075,8.721827697834584},length_median=4.8359450589884645,length_range={1.2097648283879892,14.645910682821949},rate=0.0021008684831794523,rate_95%_HPD={8.518064973992791E-4,0.003833992703980199},rate_median=0.0019215097433903205,rate_range={6.406077391952247E-4,0.007597571396348662}]:5.051160003753662,79[&height=2.315443141321398E-14,height_95%_HPD={0.0,5.6843418860808015E-14},height_median=2.8421709430404007E-14,height_range={0.0,8.526512829121202E-14},length=5.051160003753657,length_95%_HPD={1.975023777998075,8.721827697834584},length_median=4.8359450589884645,length_range={1.2097648283879892,14.645910682821949},rate=0.002100933413658706,rate_95%_HPD={8.227605682868408E-4,0.003810218792610418},rate_median=0.001915389221017822,rate_range={6.562548912113605E-4,0.009204012592111602}]:5.051160003753662)[&height=5.051160003753685,height_95%_HPD={1.975023777998132,8.721827697834613},height_median=4.835945058988493,height_range={1.2097648283880176,14.645910682821977},length=7.674041985921814,length_95%_HPD={2.7700498597814516,12.935008115944768},length_median=7.418358663009194,length_range={1.4268525721064123,18.46006137606156},posterior=1.0,rate=0.0029112797877472616,rate_95%_HPD={0.0011600778604298847,0.0054644835090276464},rate_median=0.002630169036763613,rate_range={9.804998721440286E-4,0.012843016925468171}]:7.674041985921827,94[&height=2.3657294524887462E-14,height_95%_HPD={0.0,5.6843418860808015E-14},height_median=2.8421709430404007E-14,height_range={0.0,8.526512829121202E-14},length=12.725201989675488,length_95%_HPD={7.791708905469079,17.918999537369885},length_median=12.436120090334235,length_range={6.8250933482108,22.14941035010054},rate=0.00314326920486052,rate_95%_HPD={0.0019775596907491647,0.00455494907946444},rate_median=0.0030766035324242495,rate_range={0.0016618710807549666,0.005580593039446125}]:12.725201989675488)[&height=12.725201989675512,height_95%_HPD={7.7917089054691075,17.918999537369913},height_median=12.436120090334263,height_range={6.825093348210828,22.149410350100567},length=5.476176349319307,length_95%_HPD={1.6288150624859838,10.473485419115704},length_median=5.012442074542655,length_range={1.0140777209415717,20.487211579791776},posterior=1.0,rate=0.0025934393247226454,rate_95%_HPD={7.467304028588856E-4,0.005024861702981848},rate_median=0.002323162076528409,rate_range={5.240269966749717E-4,0.01012782209705676}]:5.476176349319278)[&height=18.20137833899479,height_95%_HPD={12.66104021128885,24.487467285521333},height_median=17.73666694858136,height_range={10.990474200447423,29.979474423288792},length=20.769509279059246,length_95%_HPD={9.483695671597104,30.452329421642787},length_median=21.115710412339865,length_range={6.967363758394413,38.775184728042504},posterior=1.0,rate=0.0032931265476410787,rate_95%_HPD={0.001779353468327049,0.005644293142590883},rate_median=0.002970507057243907,rate_range={0.0015672189915059604,0.008568649993237042}]:20.769509279059253,73[&height=2.3407283486315335E-14,height_95%_HPD={0.0,2.8421709430404007E-14},height_median=2.8421709430404007E-14,height_range={0.0,8.526512829121202E-14},length=38.97088761805402,length_95%_HPD={27.17179373924303,50.73595989665348},length_median=38.863071363901774,length_range={22.2667479453348,57.55417338191219},rate=0.0018154309844734908,rate_95%_HPD={0.0012641361429891371,0.002420553071641133},rate_median=0.0017725001857392636,rate_range={0.0011384286810952574,0.003194511425264617}]:38.97088761805402)[&height=38.97088761805404,height_95%_HPD={27.171793739243057,50.73595989665351},height_median=38.86307136390179,height_range={22.266747945334856,57.55417338191222},length=19.957235479418966,length_95%_HPD={8.674115611628551,32.971597457561856},length_median=19.420378762745862,length_range={4.3040513781111684,41.003490316422514},posterior=1.0,rate=0.003142740099819102,rate_95%_HPD={0.0014872468586053856,0.005430380361065448},rate_median=0.002867350536720036,rate_range={0.0013551136911587422,0.012040568805445292}]:19.957235479418813,(83[&height=2.3341939692143074E-14,height_95%_HPD={0.0,2.8421709430404007E-14},height_median=2.8421709430404007E-14,height_range={0.0,8.526512829121202E-14},length=44.75267857704112,length_95%_HPD={28.206303239723184,61.22497971469967},length_median=45.35862918868162,length_range={13.89488420461268,67.0996630463044},rate=0.002153159607780481,rate_95%_HPD={0.0014502051528863347,0.003172468429673556},rate_median=0.0020290981892132475,rate_range={0.0013541346246763951,0.006475815824236866}]:44.75267857704112,89[&height=2.3341939692143074E-14,height_95%_HPD={0.0,2.8421709430404007E-14},height_median=2.8421709430404007E-14,height_range={0.0,8.526512829121202E-14},length=44.75267857704112,length_95%_HPD={28.206303239723184,61.22497971469967},length_median=45.35862918868162,length_range={13.89488420461268,67.0996630463044},rate=0.002355725960006302,rate_95%_HPD={0.001557710680187765,0.0035018616401864206},rate_median=0.0022258969633776053,rate_range={0.0014382218824377185,0.007291723745748583}]:44.75267857704112)[&height=44.75267857704114,height_95%_HPD={28.206303239723212,61.2249797146997},height_median=45.35862918868163,height_range={13.89488420461268,67.09966304630443},length=14.175444520431846,length_95%_HPD={3.3078228652208423,27.664849580317835},length_median=13.15355355259539,length_range={1.7050581927721566,40.86391243782384},posterior=1.0,rate=0.0015836473446551324,rate_95%_HPD={4.5985677978200565E-4,0.0034393243125722564},rate_median=0.0013227349600422472,rate_range={3.998891220793772E-4,0.009993726770035563}]:14.175444520431718)[&height=58.928123097472856,height_95%_HPD={47.866201121882895,68.55663234691495},height_median=58.988105483160645,height_range={47.81302890739828,78.10817404698876},length=17.666847610844744,length_95%_HPD={7.544805394340614,28.942330717291455},length_median=17.37745843220914,length_range={4.196314814315741,38.122457633165396},posterior=1.0,rate=0.0037459519242658227,rate_95%_HPD={0.0017630293880420674,0.006660983278773788},rate_median=0.003347325094062446,rate_range={0.0016192874694232486,0.013174066970343378}]:17.666847610844975,(43[&height=2.326239072532467E-14,height_95%_HPD={0.0,2.8421709430404007E-14},height_median=2.8421709430404007E-14,height_range={0.0,5.6843418860808015E-14},length=59.75253296134131,length_95%_HPD={56.0014480108637,65.83651179572145},length_median=58.929803541412944,length_range={56.00013031088713,73.38654777351427},rate=0.0010950345436485504,rate_95%_HPD={9.585136275382911E-4,0.0012143894723519817},rate_median=0.001100706233121432,rate_range={8.566110818500907E-4,0.0013124541936729013}]:59.75253296134132,((68[&height=2.315443141321398E-14,height_95%_HPD={0.0,2.8421709430404007E-14},height_median=2.8421709430404007E-14,height_range={0.0,5.6843418860808015E-14},length=15.339517349081957,length_95%_HPD={5.204808998918651,25.930185947896348},length_median=14.453249722871092,length_range={4.088369430488882,42.549175796531514},rate=0.002524293348757764,rate_95%_HPD={0.0010215847248931287,0.004327982473851934},rate_median=0.002362661972414146,rate_range={7.909732974729857E-4,0.008336678097024168}]:15.339517349081957,88[&height=2.315443141321398E-14,height_95%_HPD={0.0,2.8421709430404007E-14},height_median=2.8421709430404007E-14,height_range={0.0,5.6843418860808015E-14},length=15.339517349081957,length_95%_HPD={5.204808998918651,25.930185947896348},length_median=14.453249722871092,length_range={4.088369430488882,42.549175796531514},rate=0.0015868437344403652,rate_95%_HPD={6.093490658181553E-4,0.002712285538055011},rate_median=0.0014878421030562945,rate_range={5.016949741334483E-4,0.005186874680549348}]:15.339517349081957)[&height=15.33951734908198,height_95%_HPD={5.204808998918679,25.930185947896376},height_median=14.45324972287112,height_range={4.08836943048891,42.549175796531514},length=7.452810354238007,length_95%_HPD={1.1339251365136533,16.737125016888598},length_median=6.28600501618957,length_range={0.446555436845685,33.07158426171148},posterior=1.0,rate=0.0012931404103092858,rate_95%_HPD={2.4253216453535954E-4,0.0030572977880326895},rate_median=0.0010392444908635338,rate_range={2.2413441633827708E-4,0.010248463803386223}]:7.4528103542381,87[&height=2.3171477620389353E-14,height_95%_HPD={0.0,2.8421709430404007E-14},height_median=2.8421709430404007E-14,height_range={0.0,5.6843418860808015E-14},length=22.792327703320062,length_95%_HPD={11.078459151379121,36.235189215495154},length_median=21.950973017313885,length_range={7.37508383102238,49.87067121849063},rate=0.0023980072200216022,rate_95%_HPD={0.001108715086284272,0.0037906877693792756},rate_median=0.0022739104077112945,rate_range={0.0010103384359067716,0.006631589268969561}]:22.792327703320055)[&height=22.79232770332008,height_95%_HPD={11.07845915137915,36.235189215495154},height_median=21.950973017313913,height_range={7.375083831022408,49.87067121849063},length=36.960205258021155,length_95%_HPD={21.75961550241702,50.30407443369873},length_median=37.250400125303464,length_range={10.573991141573131,59.602500840706696},posterior=1.0,rate=0.0014860270861926225,rate_95%_HPD={9.472419109242201E-4,0.0021949174488957893},rate_median=0.001404812184222658,rate_range={8.131433699347389E-4,0.004957354087285908}]:36.96020525802126)[&height=59.752532961341345,height_95%_HPD={56.0014480108637,65.83651179572145},height_median=58.92980354141297,height_range={56.00013031088716,73.3865477735143},length=16.84243774697642,length_95%_HPD={8.589808260570635,25.958669483854933},length_median=16.414740879264805,length_range={5.034543320962726,31.23161970106277},posterior=1.0,rate=0.0036458819550547507,rate_95%_HPD={0.0018759581688707202,0.005797377974974328},rate_median=0.0034420078311229096,rate_range={0.0017970323865095705,0.010446894443234137}]:16.842437746976486)[&height=76.59497070831783,height_95%_HPD={67.45884314943713,86.6672332246928},height_median=76.3530949828976,height_range={62.00792480579722,92.67850423590886},length=3.785023564505134,length_95%_HPD={0.5829473819852211,7.789085337108872},length_median=3.3520393847606655,length_range={0.39218487815507785,13.877056820453419},posterior=1.0,rate=0.002061526302878341,rate_95%_HPD={4.7739191175696894E-4,0.0045410337854039345},rate_median=0.0017246034184704227,rate_range={3.7748468087172313E-4,0.014058190193017463}]:3.78502356450511,(50[&height=2.324250348362007E-14,height_95%_HPD={0.0,2.8421709430404007E-14},height_median=2.8421709430404007E-14,height_range={0.0,5.6843418860808015E-14},length=66.06721196155836,length_95%_HPD={48.20862336675316,79.04409314785896},length_median=67.10884557116523,length_range={47.80072055250062,89.3595068923524},rate=8.38302247412801E-4,rate_95%_HPD={6.468399306111702E-4,0.001096585508378174},rate_median=8.117501642458877E-4,rate_range={5.671270399099219E-4,0.0012207511544682248}]:66.06721196155836,102[&height=2.324250348362007E-14,height_95%_HPD={0.0,2.8421709430404007E-14},height_median=2.8421709430404007E-14,height_range={0.0,5.6843418860808015E-14},length=66.06721196155836,length_95%_HPD={48.20862336675316,79.04409314785896},length_median=67.10884557116523,length_range={47.80072055250062,89.3595068923524},rate=3.409986840624437E-4,rate_95%_HPD={2.564700490710274E-4,4.49429205755931E-4},rate_median=3.3090201880638754E-4,rate_range={2.1948891952743973E-4,5.373843019281896E-4}]:66.06721196155836)[&height=66.06721196155839,height_95%_HPD={48.20862336675319,79.04409314785902},height_median=67.10884557116526,height_range={47.80072055250065,89.3595068923524},length=14.312782311264307,length_95%_HPD={2.136231162569217,32.53461727541078},length_median=11.967794725752114,length_range={1.6135094856342675,44.65741174167769},posterior=1.0,rate=0.0012464651331677756,rate_95%_HPD={2.7330180467906784E-4,0.003182402611093407},rate_median=9.831062692860961E-4,rate_range={2.6661067269563696E-4,0.007451259205888543}]:14.312782311264556)[&height=80.37999427282294,height_95%_HPD={71.01710030965502,90.23075762150046},height_median=79.97141820494207,height_range={66.82860830298313,96.59055486076807},length=3.946602367083966,length_95%_HPD={0.5218663348381938,8.637902795866637},length_median=3.3778857049368867,length_range={0.3966118645049903,16.87385060444373},posterior=1.0,rate=0.0015930528438220489,rate_95%_HPD={3.376897567886099E-4,0.003528161589160978},rate_median=0.0013662287183535153,rate_range={2.660124209445685E-4,0.009822743231326547}]:3.9466023670836563)[&height=84.3265966399066,height_95%_HPD={75.99805780325629,94.64806180084393},height_median=83.79446227856909,height_range={72.34137454211904,99.66463860840675},length=5.596522783849041,length_95%_HPD={0.6693555287162383,11.622094282218981},length_median=4.980978581935879,length_range={0.5424615140245237,21.788539433623384},posterior=1.0,rate=0.002257768079492601,rate_95%_HPD={4.605384983701193E-4,0.005591531115384372},rate_median=0.0017796458263060525,rate_range={3.9812206531367466E-4,0.014603296592966488}]:5.5965227838491955,61[&height=2.3245344518149298E-14,height_95%_HPD={0.0,2.8421709430404007E-14},height_median=2.8421709430404007E-14,height_range={0.0,5.6843418860808015E-14},length=89.92311942375576,length_95%_HPD={81.56618892393853,99.27649397724117},length_median=89.58050142706705,length_range={76.54739597857987,105.51384995902929},rate=0.0010505661705849324,rate_95%_HPD={9.24790071323987E-4,0.0011623118294907771},rate_median=0.001051552738132649,rate_range={8.400103041261851E-4,0.0012799734841539086}]:89.92311942375576)[&height=89.9231194237558,height_95%_HPD={81.56618892393855,99.2764939772412},height_median=89.58050142706708,height_range={76.54739597857987,105.51384995902929},length=7.585545407180765,length_95%_HPD={1.385713664475773,15.669544050182722},length_median=6.8025723791696535,length_range={0.832525937664073,20.542910860452537},posterior=1.0,rate=0.0020151276331541014,rate_95%_HPD={5.563796589152587E-4,0.004407418563631962},rate_median=0.001679797842162654,rate_range={4.609610814251284E-4,0.013579476101426477}]:7.559953564984795)[&height=97.48307298874059,height_95%_HPD={88.47186288249515,106.40448987464033},height_median=97.41304682224728,height_range={84.39739807369685,110.80474131953969},length=2.7296302718980994,length_95%_HPD={0.4718747925759743,5.835662473310052},length_median=2.3870376840926752,length_range={0.27308760520494957,11.01026003068776},posterior=0.8442622950819673,rate=0.0021431606864415875,rate_95%_HPD={4.1375291417856036E-4,0.004749606925671139},rate_median=0.0018173969466561914,rate_range={3.26299434057457E-4,0.013991567195902032}]:2.3301157602739266,(56[&height=2.323966244909084E-14,height_95%_HPD={0.0,2.8421709430404007E-14},height_median=2.8421709430404007E-14,height_range={0.0,5.6843418860808015E-14},length=71.96004907852418,length_95%_HPD={44.40741776850493,100.66632266975235},length_median=73.57283481856959,length_range={29.240269776486997,105.90309744363901},rate=0.0012224020457738322,rate_95%_HPD={7.96224041396392E-4,0.0018383021891132155},rate_median=0.0011298962452332777,rate_range={7.577564210443634E-4,0.002898799462436743}]:71.96004907852416,(57[&height=2.3233980380032383E-14,height_95%_HPD={0.0,2.8421709430404007E-14},height_median=2.8421709430404007E-14,height_range={0.0,5.6843418860808015E-14},length=38.623797624107105,length_95%_HPD={19.68081449101156,67.12806036271057},length_median=37.26480319031536,length_range={11.955167525256357,81.74786345437133},rate=0.002374169943575137,rate_95%_HPD={0.001089690143980425,0.0038640886568614835},rate_median=0.002224845479119249,rate_range={9.87892463714273E-4,0.0070246496772368595}]:38.62379762410711,75[&height=2.3233980380032383E-14,height_95%_HPD={0.0,2.8421709430404007E-14},height_median=2.8421709430404007E-14,height_range={0.0,5.6843418860808015E-14},length=38.623797624107105,length_95%_HPD={19.68081449101156,67.12806036271057},length_median=37.26480319031536,length_range={11.955167525256357,81.74786345437133},rate=0.0021880673292146366,rate_95%_HPD={0.0010016319104785751,0.0035645808714491753},rate_median=0.0020557745334803656,rate_range={8.797315016405939E-4,0.006558967272910106}]:38.62379762410711)[&height=38.623797624107134,height_95%_HPD={19.68081449101159,67.1280603627106},height_median=37.26480319031538,height_range={11.955167525256385,81.74786345437136},length=33.336251454417194,length_95%_HPD={3.93859300116587,55.619930010857985},length_median=34.18951964554313,length_range={3.7842091042822688,68.66263640929375},posterior=1.0,rate=0.0027876827875030945,rate_95%_HPD={8.776561952512725E-4,0.008721715306152452},rate_median=0.0018350654420968798,rate_range={8.776561952512725E-4,0.016520700632305162}]:33.33625145441706)[&height=71.9600490785242,height_95%_HPD={44.407417768504956,100.66632266975238},height_median=73.57283481856962,height_range={29.240269776487025,105.90309744363901},length=27.391470769529548,length_95%_HPD={4.634902630536288,52.801128204864284},length_median=25.1696069708752,length_range={2.6474992988770367,78.0224907471947},posterior=1.0,rate=0.001171148553969273,rate_95%_HPD={2.746446465246435E-4,0.0028233660797449744},rate_median=8.940757143356152E-4,rate_range={2.746446465246435E-4,0.007740841221075021}]:27.85313967049032)[&height=99.81318874901451,height_95%_HPD={89.98050882178993,108.99923665560472},height_median=99.81624381526538,height_range={85.16111252971193,114.06086397505695},length=8.381980295650008,length_95%_HPD={2.451468528230251,14.546857283378458},length_median=7.970562477177651,length_range={1.8326448268830262,21.3724780127652},posterior=1.0,rate=0.004154163713321315,rate_95%_HPD={0.0014820623402842631,0.007916993075066181},rate_median=0.0036778203799522034,rate_range={0.0013076620552593618,0.014983486638620732}]:8.38198029565001,((((((11[&height=2.2878851063878796E-14,height_95%_HPD={0.0,5.6843418860808015E-14},height_median=2.8421709430404007E-14,height_range={0.0,8.526512829121202E-14},length=29.856527457154847,length_95%_HPD={18.528725607324134,39.353309824329074},length_median=30.17740823136966,length_range={15.189816010615104,44.560670137545685},rate=0.004807564670677046,rate_95%_HPD={0.003280837886372451,0.006865014484149027},rate_median=0.004603891118742966,rate_range={0.002961481170418204,0.00887849899882012}]:29.856527457154854,49[&height=2.2878851063878796E-14,height_95%_HPD={0.0,5.6843418860808015E-14},height_median=2.8421709430404007E-14,height_range={0.0,8.526512829121202E-14},length=29.856527457154847,length_95%_HPD={18.528725607324134,39.353309824329074},length_median=30.17740823136966,length_range={15.189816010615104,44.560670137545685},rate=0.002176111934249075,rate_95%_HPD={0.0014776392201072742,0.003109030667059196},rate_median=0.0020793552929598428,rate_range={0.001399550432902976,0.004201879812621906}]:29.856527457154854)[&height=29.856527457154876,height_95%_HPD={18.528725607324162,39.353309824329074},height_median=30.17740823136969,height_range={15.189816010615104,44.56067013754571},length=7.169530807320746,length_95%_HPD={1.0445047126851534,15.765425507829633},length_median=6.244910844943334,length_range={0.6491917033848864,29.35545133329464},posterior=1.0,rate=0.0019950920381942086,rate_95%_HPD={3.933566128054092E-4,0.004344812661967722},rate_median=0.0016574376026585946,rate_range={3.3294405061725215E-4,0.012967444994096603}]:7.169530807320747,((52[&height=2.2893056236524938E-14,height_95%_HPD={0.0,5.6843418860808015E-14},height_median=2.8421709430404007E-14,height_range={0.0,8.526512829121202E-14},length=26.949908364858036,length_95%_HPD={16.829231327406433,36.46731719509667},length_median=26.62140860535171,length_range={14.560365594438593,43.09685388451524},rate=0.0022889343946222774,rate_95%_HPD={0.0014628777351190397,0.003200081990640159},rate_median=0.0022213612095288602,rate_range={0.001336637778876454,0.003954811997532036}]:26.94130476694867,71[&height=2.288737416746648E-14,height_95%_HPD={0.0,5.6843418860808015E-14},height_median=2.8421709430404007E-14,height_range={0.0,8.526512829121202E-14},length=26.954443913065038,length_95%_HPD={16.829231327406433,36.48630873866628},length_median=26.625097352831226,length_range={14.560365594438593,43.09685388451524},rate=0.0031186683985375027,rate_95%_HPD={0.002082709841686757,0.004398555186906239},rate_median=0.0030412247232484797,rate_range={0.001843657248957533,0.0054471235345765}]:26.94130476694867)[&height=26.94130476694869,height_95%_HPD={16.82923132740646,36.457484559222536},height_median=26.614461418462973,height_range={14.560365594438593,43.09685388451527},length=3.5083844119369565,length_95%_HPD={0.5292924736592965,7.877950934319912},length_median=2.9928590537548416,length_range={0.4188965423796134,17.210903849231784},posterior=0.9972011195521792,rate=0.0016135959119819492,rate_95%_HPD={3.051419196574694E-4,0.0036154764255817845},rate_median=0.0013537999429438642,rate_range={2.4495426316345546E-4,0.009919055955273263}]:3.5124259136474087,64[&height=2.3100451757158635E-14,height_95%_HPD={0.0,5.6843418860808015E-14},height_median=2.8421709430404007E-14,height_range={0.0,8.526512829121202E-14},length=30.447751324132746,length_95%_HPD={20.313329992151665,39.87273873898059},length_median=30.6528396441144,length_range={17.506551087134994,46.273952519342714},rate=0.0028928121777958135,rate_95%_HPD={0.002069213246643326,0.004035642642399929},rate_median=0.0027941058918649697,rate_range={0.0018539698665751602,0.005036220142542071}]:30.453730680596074)[&height=30.4537306805961,height_95%_HPD={20.313329992151694,39.910359841271685},height_median=30.65686264122057,height_range={17.506551087134994,46.27395251934274},length=6.572327583879375,length_95%_HPD={1.222323242529086,15.20468329890737},length_median=5.435727604927642,length_range={0.6379892808024294,33.97249782491184},posterior=1.0,rate=0.0016375078481513886,rate_95%_HPD={2.807771859573702E-4,0.0035901256302957496},rate_median=0.0014107704450747852,rate_range={2.732638276960106E-4,0.008689170963663546}]:6.572327583879524)[&height=37.02605826447562,height_95%_HPD={26.768067593392658,47.274751054285176},height_median=37.13457073860144,height_range={21.408559890500143,59.144741173975945},length=7.864760238249692,length_95%_HPD={1.729267363347759,15.374826514145951},length_median=7.242368886051871,length_range={0.7849266566439042,23.143738323803277},posterior=1.0,rate=0.0019472263747888176,rate_95%_HPD={5.790399095553793E-4,0.004404127358688655},rate_median=0.001598419815271777,rate_range={5.034201393752423E-4,0.01497591052038767}]:7.864760238249829,((84[&height=2.3077723480924805E-14,height_95%_HPD={0.0,5.6843418860808015E-14},height_median=2.8421709430404007E-14,height_range={0.0,8.526512829121202E-14},length=22.70012628954336,length_95%_HPD={9.198436952781407,34.584340910213314},length_median=22.99110776070134,length_range={7.428877624234758,41.0972465493131},rate=0.002674793313972272,rate_95%_HPD={0.0013556322341277854,0.0048728654910585026},rate_median=0.002350355920962399,rate_range={0.0012225746841395806,0.006913464602572083}]:22.700126289543356,96[&height=2.3077723480924805E-14,height_95%_HPD={0.0,5.6843418860808015E-14},height_median=2.8421709430404007E-14,height_range={0.0,8.526512829121202E-14},length=22.70012628954336,length_95%_HPD={9.198436952781407,34.584340910213314},length_median=22.99110776070134,length_range={7.428877624234758,41.0972465493131},rate=0.0026162662028496528,rate_95%_HPD={0.0013124664497788199,0.004728312834614926},rate_median=0.0023066891415652567,rate_range={0.0012370826664013593,0.007058025152653637}]:22.700126289543356)[&height=22.700126289543377,height_95%_HPD={9.198436952781435,34.58434091021334},height_median=22.991107760701354,height_range={7.4288776242347865,41.0972465493131},length=15.428329370437108,length_95%_HPD={4.523747616043238,28.611726716336435},length_median=14.488783097201122,length_range={2.8199384682704647,38.283715573522656},posterior=1.0,rate=0.0017913857162567383,rate_95%_HPD={6.081354599347968E-4,0.0036683263993103417},rate_median=0.001563171995815637,rate_range={5.898174100418806E-4,0.007391782764968996}]:15.428329370437119,105[&height=2.3037948997515605E-14,height_95%_HPD={0.0,2.8421709430404007E-14},height_median=2.8421709430404007E-14,height_range={0.0,8.526512829121202E-14},length=38.12845565998048,length_95%_HPD={22.202239513270655,51.848517903937164},length_median=38.793827334235914,length_range={14.539716395660605,61.33448605362673},rate=0.0022705549275153967,rate_95%_HPD={0.0014418030404088472,0.0034644722038897346},rate_median=0.002121532600158064,rate_range={0.001354877883350423,0.0057173572305219715}]:38.128455659980474)[&height=38.128455659980496,height_95%_HPD={22.202239513270683,51.84851790393719},height_median=38.79382733423594,height_range={14.539716395660633,61.33448605362676},length=6.762362842744824,length_95%_HPD={0.8689080218163809,17.194075047650927},length_median=5.350048080910696,length_range={0.5148343987958839,29.77562260726239},posterior=1.0,rate=0.0015340336453410989,rate_95%_HPD={2.524692577815654E-4,0.0036566064241157297},rate_median=0.001258327965445304,rate_range={2.1253389816965756E-4,0.011506916360569743}]:6.762362842744956)[&height=44.89081850272545,height_95%_HPD={33.99732639832186,55.63466360196733},height_median=44.69457785405407,height_range={29.523603862700796,68.52454716826226},length=17.295780559674668,length_95%_HPD={5.992920248471307,32.02395901075697},length_median=15.85417032612606,length_range={3.3083538391896354,41.19428295455866},posterior=1.0,rate=0.002199079336264916,rate_95%_HPD={7.698891922663063E-4,0.004197967935947933},rate_median=0.0020005444675648214,rate_range={7.37665804478605E-4,0.009969996064200102}]:17.295780559674547,((((22[&height=2.2853281753115738E-14,height_95%_HPD={0.0,5.6843418860808015E-14},height_median=2.8421709430404007E-14,height_range={0.0,8.526512829121202E-14},length=4.3176905561923435,length_95%_HPD={1.245006497346992,7.955838524446705},length_median=4.012367924635754,length_range={0.8552280775598717,15.160947230730969},rate=0.0032940658662615513,rate_95%_HPD={9.77282990627995E-4,0.0066142573369524175},rate_median=0.0028916630846593464,rate_range={7.641598619875549E-4,0.014928059229377568}]:4.317690556192346,103[&height=2.2853281753115738E-14,height_95%_HPD={0.0,5.6843418860808015E-14},height_median=2.8421709430404007E-14,height_range={0.0,8.526512829121202E-14},length=4.3176905561923435,length_95%_HPD={1.245006497346992,7.955838524446705},length_median=4.012367924635754,length_range={0.8552280775598717,15.160947230730969},rate=0.001070740709994912,rate_95%_HPD={3.4904958668710887E-4,0.0021610958221011498},rate_median=9.426669109693612E-4,rate_range={2.635031153626766E-4,0.004928584935841415}]:4.317690556192346)[&height=4.317690556192369,height_95%_HPD={1.2450064973470205,7.955838524446733},height_median=4.012367924635768,height_range={0.8552280775598717,15.160947230730969},length=22.79071128090285,length_95%_HPD={10.136585944576424,36.108480684798224},length_median=22.680732205528948,length_range={6.426248394650827,44.09578748078911},posterior=1.0,rate=0.003897690003759647,rate_95%_HPD={0.002000251905806469,0.006855077530055762},rate_median=0.003504483038777253,rate_range={0.0016508609153998764,0.012343295793175738}]:22.790711280902862,97[&height=2.3126021067921692E-14,height_95%_HPD={0.0,5.6843418860808015E-14},height_median=2.8421709430404007E-14,height_range={0.0,8.526512829121202E-14},length=27.10840183709519,length_95%_HPD={13.854087660152473,40.53392974942096},length_median=26.99638404032379,length_range={9.973748370631967,45.348342820371585},rate=0.00195830687630906,rate_95%_HPD={0.001076213501534766,0.0030975651065010368},rate_median=0.0018219950889315788,rate_range={0.0010340284458995902,0.004935798106415725}]:27.108401837095208)[&height=27.108401837095233,height_95%_HPD={13.854087660152473,40.53392974942099},height_median=26.996384040323804,height_range={9.973748370631995,45.348342820371585},length=22.25381807558585,length_95%_HPD={6.5302679361848845,37.63166260910462},length_median=21.27674181364611,length_range={6.043300956562845,49.39227850651018},posterior=1.0,rate=0.0031280315356589897,rate_95%_HPD={0.0012350329745296365,0.00615561337012991},rate_median=0.0028143663754102537,rate_range={0.0012350329745296365,0.009455193034933083}]:22.253818075585873,((51[&height=2.3114656929804777E-14,height_95%_HPD={0.0,5.6843418860808015E-14},height_median=2.8421709430404007E-14,height_range={0.0,8.526512829121202E-14},length=12.668070600489306,length_95%_HPD={4.863136141538632,21.95856358150769},length_median=12.209538239194245,length_range={3.296121446976656,28.661017072720497},rate=0.0033532685669661905,rate_95%_HPD={0.0014031937248816788,0.0058505837103618435},rate_median=0.003065209643972876,rate_range={0.0012800290577932034,0.011490908343361722}]:12.668070600489314,54[&height=2.3114656929804777E-14,height_95%_HPD={0.0,5.6843418860808015E-14},height_median=2.8421709430404007E-14,height_range={0.0,8.526512829121202E-14},length=12.668070600489306,length_95%_HPD={4.863136141538632,21.95856358150769},length_median=12.209538239194245,length_range={3.296121446976656,28.661017072720497},rate=0.0014697402646483178,rate_95%_HPD={6.440395451131856E-4,0.002589697440415837},rate_median=0.0013501817925174113,rate_range={5.816007476611947E-4,0.0051819221592584695}]:12.668070600489314)[&height=12.668070600489337,height_95%_HPD={4.863136141538632,21.95856358150772},height_median=12.209538239194274,height_range={3.2961214469766844,28.661017072720526},length=17.080792821894068,length_95%_HPD={5.588326416483682,31.613291878840926},length_median=15.78874419350128,length_range={3.64695510531196,41.864043349622705},posterior=1.0,rate=0.0020491160140660046,rate_95%_HPD={7.071241580978297E-4,0.004088943596729465},rate_median=0.0018218259654269806,rate_range={6.234964911710285E-4,0.008039472011571992}]:17.08079282189397,74[&height=2.319704693115241E-14,height_95%_HPD={0.0,5.6843418860808015E-14},height_median=2.8421709430404007E-14,height_range={0.0,8.526512829121202E-14},length=29.748863422383277,length_95%_HPD={18.00447113387864,44.95024357764228},length_median=28.529279165926503,length_range={14.728449464746348,52.618733761281504},rate=0.0017621701203556258,rate_95%_HPD={0.0010022632317957423,0.002573564822103798},rate_median=0.0017299381007783777,rate_range={9.293898423021891E-4,0.0034480011734211325}]:29.74886342238328)[&height=29.748863422383305,height_95%_HPD={18.00447113387864,44.95024357764228},height_median=28.529279165926532,height_range={14.728449464746376,52.61873376128153},length=19.613356490297726,length_95%_HPD={3.9791074701176683,35.56450475227375},length_median=19.12765524942619,length_range={2.644154250109267,49.071051782208926},posterior=1.0,rate=0.0019626591711437457,rate_95%_HPD={6.000416378405013E-4,0.004816298581313274},rate_median=0.001588336825561539,rate_range={5.833128269182397E-4,0.011902410117195823}]:19.6133564902978)[&height=49.362219912681105,height_95%_HPD={33.9042667097639,63.96812014841038},height_median=49.06983864321977,height_range={27.655147395697895,77.59207913117224},length=7.175700875437388,length_95%_HPD={0.9287983195532377,15.027855952823089},length_median=6.488416107684692,length_range={0.47842534074953846,27.96790885630753},posterior=1.0,rate=0.0015984143811417636,rate_95%_HPD={3.823946193258128E-4,0.003627377060257192},rate_median=0.0012873319228084094,rate_range={2.6781898989273686E-4,0.013012430283580017}]:7.1757008754374,48[&height=2.317715968944781E-14,height_95%_HPD={0.0,2.8421709430404007E-14},height_median=2.8421709430404007E-14,height_range={0.0,5.6843418860808015E-14},length=56.53792078811849,length_95%_HPD={40.59637214557375,69.78748689189798},length_median=56.776406915320315,length_range={36.91065191938338,81.46210259463525},rate=0.001769020467375383,rate_95%_HPD={0.001364735073113611,0.002360462697554447},rate_median=0.001723578774126558,rate_range={0.0011923684313540981,0.002719009116489182}]:56.537920788118484)[&height=56.537920788118505,height_95%_HPD={40.59637214557378,69.78748689189801},height_median=56.77640691532035,height_range={36.91065191938341,81.46210259463528},length=5.648678274281616,length_95%_HPD={0.6053203505432236,12.568302532027332},length_median=4.837441741303174,length_range={0.4736058920632473,24.977479758654184},posterior=1.0,rate=0.0016049634943691498,rate_95%_HPD={2.896012924865495E-4,0.0038259338838944855},rate_median=0.0012842521309674088,rate_range={2.730591925972857E-4,0.012227952391430952}]:5.648678274281494)[&height=62.1865990624,height_95%_HPD={46.08070533200298,76.82196487522143},height_median=62.20286897435709,height_range={40.98578412554704,91.17636558125106},length=27.223118997222883,length_95%_HPD={14.204756396027264,41.041043722504924},length_median=26.920920069644026,length_range={9.397097441425572,50.53540882165426},posterior=1.0,rate=0.003138085142547369,rate_95%_HPD={0.0017004254095978604,0.005050373780258621},rate_median=0.0029261918252517487,rate_range={0.0015353118545428878,0.008174323352000013}]:27.223118997222663,(((33[&height=2.3211252103798556E-14,height_95%_HPD={0.0,2.8421709430404007E-14},height_median=2.8421709430404007E-14,height_range={0.0,5.6843418860808015E-14},length=32.74456666687977,length_95%_HPD={16.12376690327237,52.65816753123224},length_median=31.923912154189523,length_range={12.2671429153162,61.33615396485632},rate=0.004681216321148495,rate_95%_HPD={0.0023685203090535872,0.007705581837905607},rate_median=0.004354003737160218,rate_range={0.00218375733684746,0.011431622597990798}]:32.74456666687978,82[&height=2.3211252103798556E-14,height_95%_HPD={0.0,2.8421709430404007E-14},height_median=2.8421709430404007E-14,height_range={0.0,5.6843418860808015E-14},length=32.74456666687977,length_95%_HPD={16.12376690327237,52.65816753123224},length_median=31.923912154189523,length_range={12.2671429153162,61.33615396485632},rate=0.004519664205801983,rate_95%_HPD={0.002254414531890906,0.007395046761969615},rate_median=0.004193898348843725,rate_range={0.0021022004156783652,0.011431622597990798}]:32.74456666687978)[&height=32.7445666668798,height_95%_HPD={16.123766903272397,52.658167531232266},height_median=31.92391215418955,height_range={12.267142915316228,61.33615396485635},length=31.003580030372316,length_95%_HPD={15.667237734465402,48.573240173222075},length_median=29.928650161243034,length_range={11.50591931785327,61.27019400204051},posterior=1.0,rate=0.005628376950520546,rate_95%_HPD={0.0027593654955249116,0.008638623316707493},rate_median=0.0053534718430427695,rate_range={0.00252424736521345,0.01301612921067168}]:31.00358003037242,72[&height=2.323966244909084E-14,height_95%_HPD={0.0,2.8421709430404007E-14},height_median=2.8421709430404007E-14,height_range={0.0,5.6843418860808015E-14},length=63.74814669725219,length_95%_HPD={40.773581564202516,83.65996924871584},length_median=64.20639917158866,length_range={26.72979218236793,94.75413498500363},rate=0.0010394774212735068,rate_95%_HPD={6.967660962284085E-4,0.001451606931660028},rate_median=9.971397910742475E-4,rate_range={6.604672162190781E-4,0.002341181062953399}]:63.748146697252196)[&height=63.74814669725222,height_95%_HPD={40.773581564202544,83.65996924871587},height_median=64.20639917158866,height_range={26.72979218236796,94.75413498500366},length=7.048893382201335,length_95%_HPD={1.3442206391348464,15.208458995917297},length_median=6.127015940409358,length_range={0.523729907928967,29.965671363766674},posterior=1.0,rate=0.001783161333480022,rate_95%_HPD={3.7729745374612364E-4,0.003760028836946341},rate_median=0.0015230644949877047,rate_range={2.9648710064398133E-4,0.017161722178268016}]:7.0488933822010225,66[&height=2.324250348362007E-14,height_95%_HPD={0.0,2.8421709430404007E-14},height_median=2.8421709430404007E-14,height_range={0.0,5.6843418860808015E-14},length=70.79704007945321,length_95%_HPD={46.27920607154388,89.40807904813647},length_median=71.44163082314262,length_range={33.82487123436317,100.61443467990058},rate=0.0012074715937717812,rate_95%_HPD={8.858734210091093E-4,0.0016852327041275358},rate_median=0.0011593157461244293,rate_range={7.829204395710341E-4,0.0024658769752636234}]:70.79704007945321)[&height=70.79704007945324,height_95%_HPD={46.27920607154391,89.4080790481365},height_median=71.44163082314265,height_range={33.8248712343632,100.61443467990061},length=18.612677980169277,length_95%_HPD={5.86492267335241,34.82649346016271},length_median=17.70707035156289,length_range={2.094109549009545,49.46342449779439},posterior=1.0,rate=0.002067159922305509,rate_95%_HPD={7.008106716196263E-4,0.004149719243737904},rate_median=0.0017275597371975621,rate_range={6.14879065662551E-4,0.014310159645467606}]:18.612677980169423)[&height=89.40971805962266,height_95%_HPD={70.1860693461912,103.56082291155312},height_median=90.09477926438305,height_range={62.706034084530785,110.2333456015374},length=5.784286898064876,length_95%_HPD={0.8690898007706096,13.330273610199256},length_median=4.810911998915692,length_range={0.5172212260031728,26.14768204920999},posterior=1.0,rate=0.0016280675824920105,rate_95%_HPD={2.8602178385705513E-4,0.0036769623534242805},rate_median=0.0013707840867769176,rate_range={2.444697231955508E-4,0.009369970253298311}]:5.784286898064963,(((38[&height=2.3256708656266213E-14,height_95%_HPD={0.0,2.8421709430404007E-14},height_median=2.8421709430404007E-14,height_range={0.0,5.6843418860808015E-14},length=56.45754730633218,length_95%_HPD={27.68373362348038,79.11388432163031},length_median=58.93524285237562,length_range={21.19433229190679,88.98833410790765},rate=0.0035699086117946996,rate_95%_HPD={0.0022032549752254897,0.006100892579067555},rate_median=0.0031690644940592042,rate_range={0.0020558543904336795,0.008641887500594192}]:56.457547306332195,90[&height=2.3256708656266213E-14,height_95%_HPD={0.0,2.8421709430404007E-14},height_median=2.8421709430404007E-14,height_range={0.0,5.6843418860808015E-14},length=56.45754730633218,length_95%_HPD={27.68373362348038,79.11388432163031},length_median=58.93524285237562,length_range={21.19433229190679,88.98833410790765},rate=0.003556645096197989,rate_95%_HPD={0.0021426583387461986,0.005992531118258001},rate_median=0.003168885858048345,rate_range={0.002051496791631865,0.008641887500594192}]:56.457547306332195)[&height=56.457547306332216,height_95%_HPD={27.68373362348038,79.11388432163034},height_median=58.93524285237565,height_range={21.194332291906818,88.98833410790768},length=18.646746496700747,length_95%_HPD={3.5379652859242157,39.405115270738264},length_median=16.366641039365405,length_range={2.11694908486362,51.32075109355135},posterior=1.0,rate=0.0016014339839018753,rate_95%_HPD={3.718995284671561E-4,0.0035346680679781807},rate_median=0.001328843088156036,rate_range={3.456228804695875E-4,0.00920516825351425}]:18.646746496700587,(91[&height=2.315727244774321E-14,height_95%_HPD={0.0,2.8421709430404007E-14},height_median=2.8421709430404007E-14,height_range={0.0,8.526512829121202E-14},length=14.604647653194679,length_95%_HPD={4.461120031030049,26.744956963684615},length_median=13.917613536330329,length_range={3.4289492607368004,46.808344139364465},rate=0.0014815943956068276,rate_95%_HPD={4.2772941009470224E-4,0.003010338622617551},rate_median=0.0012947943624813615,rate_range={4.0742032026666434E-4,0.005218225366391905}]:14.604647653194679,92[&height=2.315727244774321E-14,height_95%_HPD={0.0,2.8421709430404007E-14},height_median=2.8421709430404007E-14,height_range={0.0,8.526512829121202E-14},length=14.604647653194679,length_95%_HPD={4.461120031030049,26.744956963684615},length_median=13.917613536330329,length_range={3.4289492607368004,46.808344139364465},rate=0.001963356975003885,rate_95%_HPD={5.507996199755748E-4,0.003950081090672968},rate_median=0.0017152047233746641,rate_range={5.109654069060318E-4,0.007096719106302353}]:14.604647653194679)[&height=14.604647653194702,height_95%_HPD={4.461120031030077,26.74495696368463},height_median=13.917613536330357,height_range={3.4289492607368004,46.808344139364465},length=60.499646149838114,length_95%_HPD={34.222187495749495,82.72775468157515},length_median=61.11022026373254,length_range={31.526754678610516,93.15120517111873},posterior=1.0,rate=0.0014690909558480636,rate_95%_HPD={9.535720879869893E-4,0.0022591132603741937},rate_median=0.0013935939431134127,rate_range={8.747185104682057E-4,0.002662524058257534}]:60.4996461498381)[&height=75.1042938030328,height_95%_HPD={54.330673070476564,94.69181531511532},height_median=75.34370184138555,height_range={43.093774081313214,103.33039544556993},length=14.016886021175798,length_95%_HPD={2.2604114964874498,31.924181379260403},length_median=11.807825357226392,length_range={1.204390401280122,52.23118999393343},posterior=1.0,rate=0.0016989196467917687,rate_95%_HPD={3.52474763980752E-4,0.0038620090238311944},rate_median=0.0014530573533987805,rate_range={3.268081601040803E-4,0.012932348571334696}]:14.016886021176035,((44[&height=2.3341939692143074E-14,height_95%_HPD={0.0,2.8421709430404007E-14},height_median=2.8421709430404007E-14,height_range={0.0,8.526512829121202E-14},length=20.073767177976638,length_95%_HPD={5.7507201769942355,40.90757560357561},length_median=18.15403080176293,length_range={5.5295847567559235,57.760348167095344},rate=0.0018216335895958195,rate_95%_HPD={5.364998141754858E-4,0.0035256533889318755},rate_median=0.0016575821240276253,rate_range={5.020364108405117E-4,0.005816439195271551}]:20.07376717797663,99[&height=2.3341939692143074E-14,height_95%_HPD={0.0,2.8421709430404007E-14},height_median=2.8421709430404007E-14,height_range={0.0,8.526512829121202E-14},length=20.073767177976638,length_95%_HPD={5.7507201769942355,40.90757560357561},length_median=18.15403080176293,length_range={5.5295847567559235,57.760348167095344},rate=0.001205186292394899,rate_95%_HPD={3.663848372675653E-4,0.0023458365906460586},rate_median=0.0010985030947345225,rate_range={3.1837566616193803E-4,0.003546645897359615}]:20.07376717797663)[&height=20.073767177976656,height_95%_HPD={5.750720176994264,40.90757560357564},height_median=18.154030801762957,height_range={5.529584756755952,57.760348167095344},length=29.558815082276023,length_95%_HPD={5.1500090614125895,59.09986503733445},length_median=27.46259580892717,length_range={4.425817736294121,75.77142803178563},posterior=1.0,rate=0.0014535386198068214,rate_95%_HPD={4.2509185959617907E-4,0.0032651100369810674},rate_median=0.0012081544910554475,rate_range={4.2509185959617907E-4,0.007881478079411097}]:29.558815082275842,45[&height=2.3256708656266213E-14,height_95%_HPD={0.0,2.8421709430404007E-14},height_median=2.8421709430404007E-14,height_range={0.0,5.6843418860808015E-14},length=49.63258226025247,length_95%_HPD={29.97301289018081,75.0818402834908},length_median=47.77174009974787,length_range={22.331098301875173,90.56053683012247},rate=0.0021560211104816153,rate_95%_HPD={0.0012458486706688166,0.0031028826704119577},rate_median=0.0021297744301150903,rate_range={0.0010969312465306832,0.004563718460837882}]:49.63258226025248)[&height=49.6325822602525,height_95%_HPD={29.97301289018084,75.08184028349083},height_median=47.77174009974789,height_range={22.3310983018752,90.5605368301225},length=39.48859756395615,length_95%_HPD={10.303314451712964,61.90777506092002},length_median=40.610083274569554,length_range={8.56355809616187,77.28420628027675},posterior=1.0,rate=0.0012159876917071741,rate_95%_HPD={5.367279793502245E-4,0.0024084759888044166},rate_median=0.0010147217046324397,rate_range={5.115080854994543E-4,0.004928429078131599}]:39.48859756395634)[&height=89.12117982420884,height_95%_HPD={71.44901729401391,104.49508153840327},height_median=89.83995470502724,height_range={62.26541118319474,109.46305736671263},length=6.072825133479084,length_95%_HPD={0.5918218177345267,14.236138498808472},length_median=5.045346097040387,length_range={0.554915802348944,25.55745952575441},posterior=1.0,rate=0.0013917433985851293,rate_95%_HPD={2.7236932649792956E-4,0.0032018658875939527},rate_median=0.0011284961212786235,rate_range={2.2830054465887342E-4,0.009089162826903247}]:6.072825133478787)[&height=95.19400495768762,height_95%_HPD={75.62392810476375,108.72341421186425},height_median=96.29322364108756,height_range={68.54619960816149,114.62155878036852},length=13.00116408697676,length_95%_HPD={2.5455196241245517,26.22969387069932},length_median=11.926541838441153,length_range={2.054747447177135,36.10643015191634},posterior=1.0,rate=0.0016310351338376492,rate_95%_HPD={4.377833919775159E-4,0.0037225715464056916},rate_median=0.0013214273493665613,rate_range={3.6602449076431623E-4,0.00793790611137256}]:13.0011640869769)[&height=108.19516904466452,height_95%_HPD={98.14487149906806,117.76131080263964},height_median=108.5900367477314,height_range={91.25436199632658,121.19202355663067},length=14.88467715647236,length_95%_HPD={7.942925673197379,23.16940832690841},length_median=14.16617632620293,length_range={6.1218200691006786,36.22777411327647},posterior=1.0,rate=0.008739545927248895,rate_95%_HPD={0.004264658288154547,0.013018442822823545},rate_median=0.008693578723209888,rate_range={0.0037505646474282656,0.017431180589356506}]:14.884677156471966,(9[&height=2.3236821414561614E-14,height_95%_HPD={0.0,2.8421709430404007E-14},height_median=2.8421709430404007E-14,height_range={0.0,5.6843418860808015E-14},length=40.66731825129602,length_95%_HPD={13.9813784092065,71.29850321401364},length_median=38.80582846303896,length_range={8.831578352210826,99.45073442687955},rate=0.0030611716084676436,rate_95%_HPD={0.001026766379644116,0.005857131260241061},rate_median=0.0027194253517947736,rate_range={8.0352590296176E-4,0.013805989401351899}]:40.66731825129602,21[&height=2.3236821414561614E-14,height_95%_HPD={0.0,2.8421709430404007E-14},height_median=2.8421709430404007E-14,height_range={0.0,5.6843418860808015E-14},length=40.66731825129602,length_95%_HPD={13.9813784092065,71.29850321401364},length_median=38.80582846303896,length_range={8.831578352210826,99.45073442687955},rate=0.0020016867817481835,rate_95%_HPD={5.532186957712757E-4,0.003727070615740635},rate_median=0.0018024718717535213,rate_range={4.905714137845735E-4,0.008144917445840463}]:40.66731825129602)[&height=40.66731825129604,height_95%_HPD={13.981378409206556,71.29850321401366},height_median=38.805828463038985,height_range={8.831578352210855,99.45073442687958},length=82.41252794984096,length_95%_HPD={51.03267990371491,110.10191216348315},length_median=84.1790630172114,length_range={23.37365300571483,120.47941836436104},posterior=1.0,rate=0.0039846489946429395,rate_95%_HPD={0.0025592775043407337,0.005991896039310642},rate_median=0.003757386841096861,rate_range={0.0023543480314499425,0.012057283261883716}]:82.41252794984045)[&height=123.07984620113649,height_95%_HPD={113.8695187694814,131.99773687394574},height_median=123.42297541423125,height_range={106.17346006800103,135.41366158477823},length=9.770233396843311,length_95%_HPD={3.164002178522722,17.238263869735334},length_median=9.299757940871139,length_range={1.0256797650291674,27.767766660094424},posterior=1.0,rate=0.005406863395855192,rate_95%_HPD={0.0017519331121877294,0.010254068346816067},rate_median=0.004895360852287847,rate_range={9.358008503148028E-4,0.015031416727008966}]:9.770233396844162,((4[&height=2.324250348362007E-14,height_95%_HPD={0.0,2.8421709430404007E-14},height_median=2.8421709430404007E-14,height_range={0.0,5.6843418860808015E-14},length=94.31820060251616,length_95%_HPD={63.27463850750317,118.88170387969039},length_median=96.41173746505423,length_range={44.185891977424276,128.04460218501697},rate=0.003075082920209033,rate_95%_HPD={0.002319792989139772,0.004114432809200738},rate_median=0.0029657658054547797,rate_range={0.0021738495737780293,0.0055420587723422005}]:100.61150625114003,(5[&height=2.323966244909084E-14,height_95%_HPD={0.0,2.8421709430404007E-14},height_median=2.8421709430404007E-14,height_range={0.0,5.6843418860808015E-14},length=85.94223634008198,length_95%_HPD={58.126660354571186,111.63176085254393},length_median=86.60944083681497,length_range={40.712639561167705,125.29009128212888},rate=0.004511916526589553,rate_95%_HPD={0.002987761799218193,0.0062633944284333455},rate_median=0.004357881485878441,rate_range={0.002630797970223485,0.010643812243935036}]:85.49731958180126,(25[&height=2.324250348362007E-14,height_95%_HPD={0.0,2.8421709430404007E-14},height_median=2.8421709430404007E-14,height_range={0.0,5.6843418860808015E-14},length=67.29347941266316,length_95%_HPD={40.3085689961043,93.26021762875705},length_median=67.5829598717728,length_range={28.181371919008996,110.83353654207032},rate=0.003750534383888516,rate_95%_HPD={0.0023545772046451325,0.005568826259414119},rate_median=0.0035620394483700914,rate_range={0.0021507192986485544,0.008093502852111729}]:67.29347941266316,26[&height=2.324250348362007E-14,height_95%_HPD={0.0,2.8421709430404007E-14},height_median=2.8421709430404007E-14,height_range={0.0,5.6843418860808015E-14},length=67.29347941266316,length_95%_HPD={40.3085689961043,93.26021762875705},length_median=67.5829598717728,length_range={28.181371919008996,110.83353654207032},rate=0.0036044290340858237,rate_95%_HPD={0.002283966274806608,0.005336823898939521},rate_median=0.003432332240980772,rate_range={0.0020566283192998956,0.008093502852111729}]:67.29347941266316)[&height=67.29347941266319,height_95%_HPD={40.30856899610433,93.26021762875708},height_median=67.5829598717728,height_range={28.181371919009024,110.83353654207035},length=24.10237213086587,length_95%_HPD={2.3732962565297555,48.33746128264926},length_median=22.814338333575805,length_range={0.24757817261041737,73.05032262275239},posterior=1.0,rate=0.0026795748083186634,rate_95%_HPD={6.083608027162917E-4,0.005784203022335794},rate_median=0.0023022507397285614,rate_range={2.5185383945512625E-4,0.012659235125417542}]:18.203840169138104)[&height=85.49731958180129,height_95%_HPD={59.27916554689514,109.75185868260877},height_median=86.27209849960607,height_range={40.712639561167734,120.82678185664949},length=15.480872288558203,length_95%_HPD={0.003551547694840451,33.778540173178214},length_median=13.930100323392445,length_range={0.003551547694840451,53.687925211983924},posterior=0.568172730907637,rate=0.0023592978874043286,rate_95%_HPD={4.064220692043639E-4,0.00498527427923123},rate_median=0.002050567383732695,rate_range={2.461616587592601E-4,0.01676167150230416}]:15.114186669338764)[&height=100.61150625114006,height_95%_HPD={77.419976136343,120.54493222320625},height_median=101.56974710629203,height_range={61.51298046715418,128.044602185017},length=18.908393658818927,length_95%_HPD={3.6104810898332858,37.86585987534852},length_median=17.20994801188813,length_range={0.18502127627326104,67.31605009447534},posterior=1.0,rate=0.0021403807063593134,rate_95%_HPD={5.329259821561309E-4,0.004725325340582343},rate_median=0.0018061936183392435,rate_range={3.223550246836499E-4,0.012186885730309245}]:18.908393658818795,(42[&height=2.3253867621736986E-14,height_95%_HPD={0.0,2.8421709430404007E-14},height_median=2.8421709430404007E-14,height_range={0.0,5.6843418860808015E-14},length=64.13550338289025,length_95%_HPD={39.24136383172005,95.50359533020891},length_median=61.86663883278155,length_range={23.71389545850259,105.5056528883423},rate=0.004081915441204922,rate_95%_HPD={0.0024019919481022794,0.005949194441757205},rate_median=0.003987729253503269,rate_range={0.002129737923691279,0.01034947876960038}]:64.13550338289024,104[&height=2.3253867621736986E-14,height_95%_HPD={0.0,2.8421709430404007E-14},height_median=2.8421709430404007E-14,height_range={0.0,5.6843418860808015E-14},length=64.13550338289025,length_95%_HPD={39.24136383172005,95.50359533020891},length_median=61.86663883278155,length_range={23.71389545850259,105.5056528883423},rate=0.0041394853855501075,rate_95%_HPD={0.0024300845277579245,0.0059744591490551845},rate_median=0.004049223420129524,rate_range={0.0022759792444562105,0.010544347997233063}]:64.13550338289024)[&height=64.13550338289026,height_95%_HPD={39.24136383172008,95.50359533020891},height_median=61.86663883278156,height_range={23.713895458502606,105.50565288834233},length=55.384396527068894,length_95%_HPD={25.9241549989478,79.85659817953463},length_median=56.87353169720518,length_range={20.17645435752489,96.16537044695849},posterior=1.0,rate=0.004748462121692087,rate_95%_HPD={0.002614365978652967,0.007969253270088555},rate_median=0.00427988831139758,rate_range={0.0024496622599522095,0.01183079772820435}]:55.38439652706859)[&height=119.51989990995885,height_95%_HPD={105.10258809935664,131.99660966905137},height_median=120.21807791918502,height_range={90.54542804897983,135.69802190158816},length=13.33017968802115,length_95%_HPD={2.815240968402108,26.666429628482774},length_median=12.377842434095761,length_range={0.207159784593955,39.10743804017119},posterior=1.0,rate=0.0024135094402524663,rate_95%_HPD={5.839222504349746E-4,0.005289875875771499},rate_median=0.002026480431531811,rate_range={3.3132170925159743E-4,0.013719460942682435}]:13.3301796880218)[&height=132.85007959798065,height_95%_HPD={125.72971989547449,138.03680275653048},height_median=133.60001376126593,height_range={113.25849730503178,138.2562482526058},length=2.766275170325173,length_95%_HPD={0.0016157935907585852,8.167562391938702},length_median=1.9450554372825621,length_range={0.0010767053518776493,22.877481873575704},posterior=1.0,rate=0.0023887864140252225,rate_95%_HPD={2.651420467692059E-4,0.005804005813402476},rate_median=0.0018842584403369893,rate_range={2.253614548998457E-4,0.016832953113110546}]:2.766275170324832,67[&height=2.3245344518149298E-14,height_95%_HPD={0.0,2.8421709430404007E-14},height_median=2.8421709430404007E-14,height_range={0.0,5.6843418860808015E-14},length=135.61635476830548,length_95%_HPD={130.5410915960901,138.32961277131028},length_median=136.38085994937558,length_range={117.97081423414485,138.32961277131028},rate=0.003697332054697921,rate_95%_HPD={0.0034079188139278277,0.004004616438500579},rate_median=0.003701957515521926,rate_range={0.00228054641510741,0.004372286860697514}]:135.61635476830546)[&height=135.61635476830548,height_95%_HPD={130.5410915960901,138.3296127713103},height_median=136.38085994937563,height_range={117.97081423414488,138.3296127713103},length=0.0,posterior=1.0,rate=1.0]:0.0;

tree TREE3 = ((((((((((((((((1[&height=2.328796003608773E-14,height_95%_HPD={0.0,5.6843418860808015E-14},height_median=2.8421709430404007E-14,height_range={0.0,8.526512829121202E-14},length=8.210133612733214,length_95%_HPD={2.7096337770537104,14.687767348835763},length_median=7.848481542606606,length_range={1.5033946769681563,22.70475301385666},rate=0.0021335798290890457,rate_95%_HPD={7.350896487464015E-4,0.003983213545038943},rate_median=0.0019014284113514333,rate_range={6.404512207010181E-4,0.010621689619815654}]:8.208680186153426,23[&height=2.328796003608773E-14,height_95%_HPD={0.0,5.6843418860808015E-14},height_median=2.8421709430404007E-14,height_range={0.0,8.526512829121202E-14},length=8.21035275895805,length_95%_HPD={2.7096337770537104,14.687767348835763},length_median=7.848481542606606,length_range={1.5033946769681563,22.70475301385666},rate=0.0033847088356976033,rate_95%_HPD={0.001171144324966961,0.006246048429939123},rate_median=0.0030019769682660576,rate_range={9.685039639859633E-4,0.014257905213487146}]:8.208680186153426)[&height=8.20868018615345,height_95%_HPD={2.797276929605516,14.752685616219978},height_median=7.846151904941735,height_range={1.5033946769681847,22.70475301385669},length=1.9857965299103573,length_95%_HPD={0.15451402371061818,4.763671570085663},length_median=1.6395767731304574,length_range={0.07714310793056711,10.056952343018423},posterior=0.9998000799680128,rate=0.001979744948410191,rate_95%_HPD={2.9890246030750384E-4,0.0047440766779389895},rate_median=0.0015583198127985625,rate_range={2.695572094284133E-4,0.02040133689533834}]:1.9870721022091793,41[&height=2.3214093138327783E-14,height_95%_HPD={0.0,5.6843418860808015E-14},height_median=2.8421709430404007E-14,height_range={0.0,8.526512829121202E-14},length=10.195533142137771,length_95%_HPD={4.436454411097685,16.810454096544106},length_median=9.782584788320086,length_range={2.1333282808541583,23.656209255971604},rate=0.0028476378173335577,rate_95%_HPD={0.0012845586953695682,0.004840644910948394},rate_median=0.002654200582917166,rate_range={0.0010345253963897222,0.01249557804403938}]:10.195752288362605)[&height=10.195752288362629,height_95%_HPD={4.436454411097685,16.810454096544163},height_median=9.782584788320115,height_range={2.1333282808541867,23.656209255971604},length=11.049833741469394,length_95%_HPD={4.573134547476059,18.77796414678265},length_median=10.527606000965228,length_range={3.1728165342488523,25.576511381678458},posterior=1.0,rate=0.0027311195254425893,rate_95%_HPD={0.0012141896590737165,0.004859936439387514},rate_median=0.0025165467097984793,rate_range={0.0010014911925554939,0.0079192334583258}]:11.049833741469461,27[&height=2.319704693115241E-14,height_95%_HPD={0.0,5.6843418860808015E-14},height_median=2.8421709430404007E-14,height_range={0.0,8.526512829121202E-14},length=21.245586029832065,length_95%_HPD={11.716991998712714,31.353002803314595},length_median=21.216724737546592,length_range={8.87215073448968,40.27532860471878},rate=0.0022994567989221967,rate_95%_HPD={0.001363580905287219,0.003715846745391895},rate_median=0.002158454011100469,rate_range={0.0010753504159664415,0.005457083195363268}]:21.245586029832065)[&height=21.24558602983209,height_95%_HPD={11.716991998712771,31.353002803314624},height_median=21.21672473754662,height_range={8.87215073448968,40.27532860471881},length=4.311299105146175,length_95%_HPD={0.5585631001629281,9.543580190226407},length_median=3.702367568494644,length_range={0.3775782828736993,17.84974327295498},posterior=1.0,rate=0.0019253567803367397,rate_95%_HPD={4.220112077788752E-4,0.004451908420020269},rate_median=0.0015781986864849772,rate_range={2.8159059861965734E-4,0.01406130617504298}]:4.311299105146144,77[&height=2.319988796568164E-14,height_95%_HPD={0.0,2.8421709430404007E-14},height_median=2.8421709430404007E-14,height_range={0.0,8.526512829121202E-14},length=25.55688513497821,length_95%_HPD={14.874681379151895,35.02164946028962},length_median=25.461938989271914,length_range={13.163054238786216,42.85612204027484},rate=0.0026396751165466597,rate_95%_HPD={0.0016428983539898195,0.003960351905640718},rate_median=0.0025176782432318814,rate_range={0.0014477937654015743,0.005005338278172255}]:25.55688513497821)[&height=25.556885134978234,height_95%_HPD={14.874681379151895,35.02164946028962},height_median=25.461938989271943,height_range={13.163054238786245,42.85612204027487},length=8.474390984175221,length_95%_HPD={1.3254679194619428,17.985265827551117},length_median=7.321850840350216,length_range={0.9760541568847145,30.255948776061928},posterior=1.0,rate=0.0017852022040300314,rate_95%_HPD={3.5825967172850066E-4,0.004278555885436667},rate_median=0.001461945604188179,rate_range={2.7590404894698226E-4,0.010827532253075967}]:8.474390984175205,(63[&height=2.3214093138327783E-14,height_95%_HPD={0.0,2.8421709430404007E-14},height_median=2.8421709430404007E-14,height_range={0.0,5.6843418860808015E-14},length=13.867075000690654,length_95%_HPD={4.119765909254113,24.896936965501226},length_median=13.126941288900113,length_range={3.1811758567096717,36.848013895505346},rate=0.0021758886461858617,rate_95%_HPD={7.722522056823693E-4,0.004253610110493977},rate_median=0.0019221438246022889,rate_range={6.33201637761629E-4,0.007930885654535364}]:13.867075000690662,100[&height=2.3214093138327783E-14,height_95%_HPD={0.0,2.8421709430404007E-14},height_median=2.8421709430404007E-14,height_range={0.0,5.6843418860808015E-14},length=13.867075000690654,length_95%_HPD={4.119765909254113,24.896936965501226},length_median=13.126941288900113,length_range={3.1811758567096717,36.848013895505346},rate=0.0021337197298361486,rate_95%_HPD={7.279118217392062E-4,0.004142367391770197},rate_median=0.0018872198550050634,rate_range={6.222033024623903E-4,0.00794100144895383}]:13.867075000690662)[&height=13.867075000690685,height_95%_HPD={4.119765909254113,24.896936965501254},height_median=13.126941288900142,height_range={3.1811758567097,36.8480138955054},length=20.164201118462742,length_95%_HPD={7.512819705616906,32.81240665824268},length_median=19.49726571284973,length_range={5.657086445352661,44.198278134953256},posterior=1.0,rate=0.0016416926597202955,rate_95%_HPD={7.462141243064826E-4,0.002951057826432454},rate_median=0.0015090308010571487,rate_range={6.26934623099986E-4,0.005218811377780764}]:20.164201118462756)[&height=34.03127611915344,height_95%_HPD={22.27838148690904,45.956749828929446},height_median=33.756262815155644,height_range={17.201864776892606,61.30068055299736},length=7.288520272483745,length_95%_HPD={0.8065014371962036,19.843936243576238},length_median=5.709979124600665,length_range={0.5557060641534832,36.31783532298833},posterior=1.0,rate=0.0017821828147091365,rate_95%_HPD={2.511927741119402E-4,0.004159115845052468},rate_median=0.0014393891643202518,rate_range={2.511927741119402E-4,0.014001782617207503}]:7.288520272483808,81[&height=2.3151590378684753E-14,height_95%_HPD={0.0,2.8421709430404007E-14},height_median=2.8421709430404007E-14,height_range={0.0,5.6843418860808015E-14},length=41.319796391637226,length_95%_HPD={28.672989569374906,55.80758613831756},length_median=40.794180525715994,length_range={21.613073576831184,68.27445768691621},rate=0.0018358267476247117,rate_95%_HPD={0.0012301179579866015,0.002429625708095165},rate_median=0.0018098135693618358,rate_range={0.001036541155676689,0.0033822897355274746}]:41.319796391637226)[&height=41.31979639163725,height_95%_HPD={28.672989569374934,55.80758613831756},height_median=40.79418052571602,height_range={21.613073576831212,68.27445768691624},length=28.44599499424133,length_95%_HPD={13.441867080041206,41.20822761529065},length_median=29.111964696673212,length_range={5.776045065801469,49.1372419047875},posterior=1.0,rate=0.0011821241304509325,rate_95%_HPD={6.721666262761451E-4,0.002006362359914188},rate_median=0.0010654645688780145,rate_range={5.898687052019918E-4,0.005030457644323064}]:28.44599499424131,(78[&height=2.3202729000210868E-14,height_95%_HPD={0.0,2.8421709430404007E-14},height_median=2.8421709430404007E-14,height_range={0.0,5.6843418860808015E-14},length=24.1282702239408,length_95%_HPD={6.01539670911518,48.900374627568866},length_median=20.58313258502831,length_range={5.00040259413565,65.60256281867443},rate=0.0017655302128776662,rate_95%_HPD={4.998922853513087E-4,0.003711693231808737},rate_median=0.0015958194721176857,rate_range={4.782104898804233E-4,0.006633445693971653}]:24.12827022394078,86[&height=2.3202729000210868E-14,height_95%_HPD={0.0,2.8421709430404007E-14},height_median=2.8421709430404007E-14,height_range={0.0,5.6843418860808015E-14},length=24.1282702239408,length_95%_HPD={6.01539670911518,48.900374627568866},length_median=20.58313258502831,length_range={5.00040259413565,65.60256281867443},rate=0.0014971773768567705,rate_95%_HPD={4.446010767694981E-4,0.003146802732218784},rate_median=0.0013533509329517674,rate_range={3.858227371456169E-4,0.005365186196293554}]:24.12827022394078)[&height=24.128270223940806,height_95%_HPD={6.015396709115237,48.900374627568894},height_median=20.583132585028338,height_range={5.00040259413565,65.60256281867446},length=45.63752116193769,length_95%_HPD={18.98837561722246,64.31664434392326},length_median=48.783500785711816,length_range={9.453329566726765,76.16780213098492},posterior=1.0,rate=8.165117841827853E-4,rate_95%_HPD={4.4956012441450257E-4,0.0015367012808456322},rate_median=6.877736940094298E-4,rate_range={4.2791644344891434E-4,0.003349432617026382}]:45.637521161937755)[&height=69.76579138587856,height_95%_HPD={66.00216176214984,75.76097506659073},height_median=69.01631060014958,height_range={66.00216176214984,85.33401499397637},length=11.276826114948427,length_95%_HPD={4.2407386649887115,19.0071491325712},length_median=10.607431606841722,length_range={2.6349466248419446,27.829551377784824},posterior=1.0,rate=0.0026787204585176885,rate_95%_HPD={0.001089868651098279,0.004673881181854142},rate_median=0.0025119389982392886,rate_range={8.558309514789233E-4,0.010027612474736929}]:11.276826114948477,((((((((((2[&height=2.302658485939869E-14,height_95%_HPD={0.0,5.6843418860808015E-14},height_median=2.8421709430404007E-14,height_range={0.0,5.6843418860808015E-14},length=14.47000390643334,length_95%_HPD={8.463718958264693,20.888509802033212},length_median=14.093759838001937,length_range={6.043012762909342,28.631327717754942},rate=0.00343512763624684,rate_95%_HPD={0.001996684112152233,0.005086215859147365},rate_median=0.003342199109744976,rate_range={0.0015813431491470712,0.007467858709746824}]:14.47000390643333,40[&height=2.302658485939869E-14,height_95%_HPD={0.0,5.6843418860808015E-14},height_median=2.8421709430404007E-14,height_range={0.0,5.6843418860808015E-14},length=14.47000390643334,length_95%_HPD={8.463718958264693,20.888509802033212},length_median=14.093759838001937,length_range={6.043012762909342,28.631327717754942},rate=0.002790181805127845,rate_95%_HPD={0.0016062851009281734,0.004081619187441633},rate_median=0.002709957138742676,rate_range={0.0012847345942019939,0.006314262448440272}]:14.47000390643333)[&height=14.470003906433353,height_95%_HPD={8.463718958264721,20.88850980203327},height_median=14.09375983800195,height_range={6.04301276290937,28.63132771775497},length=8.894655632350897,length_95%_HPD={2.7917419460580106,16.144778383534018},length_median=8.377889448168027,length_range={1.4646849309016972,21.8305700406421},posterior=1.0,rate=0.0027426429008966624,rate_95%_HPD={9.81924540122463E-4,0.005313874167063236},rate_median=0.002415627281180508,rate_range={8.320413528519674E-4,0.014344343847834114}]:8.894655632350842,((32[&height=2.3074882446395577E-14,height_95%_HPD={0.0,5.6843418860808015E-14},height_median=2.8421709430404007E-14,height_range={0.0,8.526512829121202E-14},length=9.085175686187162,length_95%_HPD={3.5190981300258386,14.807039558735298},length_median=8.717487779652544,length_range={2.8457672418922755,22.735481247223916},rate=0.0068514090346173135,rate_95%_HPD={0.002983730034408482,0.012189950203818635},rate_median=0.006370218044401086,rate_range={0.0024746558310784032,0.01884125997978434}]:9.085175686187156,58[&height=2.3074882446395577E-14,height_95%_HPD={0.0,5.6843418860808015E-14},height_median=2.8421709430404007E-14,height_range={0.0,8.526512829121202E-14},length=9.085175686187162,length_95%_HPD={3.5190981300258386,14.807039558735298},length_median=8.717487779652544,length_range={2.8457672418922755,22.735481247223916},rate=0.0014260337009166567,rate_95%_HPD={5.856500605885354E-4,0.0025258229741452506},rate_median=0.0013204009690306554,rate_range={4.2728557408406415E-4,0.004342529166492742}]:9.085175686187156)[&height=9.08517568618718,height_95%_HPD={3.5190981300258386,14.807039558735326},height_median=8.717487779652558,height_range={2.8457672418922755,22.735481247223916},length=6.864341241754918,length_95%_HPD={1.577091662291508,13.188870119664728},length_median=6.355947006059175,length_range={0.8903503865880964,19.01534485103521},posterior=1.0,rate=0.0029389433813088346,rate_95%_HPD={8.916326804137759E-4,0.006486059636681836},rate_median=0.0024995351382572062,rate_range={6.71122147386391E-4,0.015582413831381215}]:6.864341241754877,55[&height=2.309192865357095E-14,height_95%_HPD={0.0,5.6843418860808015E-14},height_median=2.8421709430404007E-14,height_range={0.0,5.6843418860808015E-14},length=15.949516927942035,length_95%_HPD={9.051296771964132,24.28127623429691},length_median=15.716186142525792,length_range={5.037113116210406,30.256981308545704},rate=0.002712124142264501,rate_95%_HPD={0.0015074764134194961,0.004078571764294046},rate_median=0.0025814285052048226,rate_range={0.0012704917015064553,0.008445923027705532}]:15.949516927942033)[&height=15.949516927942057,height_95%_HPD={9.051296771964132,24.28127623429691},height_median=15.716186142525835,height_range={5.037113116210435,30.25698130854576},length=7.415142610842121,length_95%_HPD={1.7124417557012777,14.210698565000953},length_median=6.942375301585031,length_range={0.9836190503456805,24.903338332612435},posterior=1.0,rate=0.0025988395719606637,rate_95%_HPD={6.592524203457207E-4,0.005714819076867336},rate_median=0.002154296314517824,rate_range={5.172917300520887E-4,0.013859578389348322}]:7.415142610842139)[&height=23.364659538784196,height_95%_HPD={16.63141002537205,30.250390925693665},height_median=23.113298997777576,height_range={13.598816139648434,34.99150573958093},length=3.4204458843485948,length_95%_HPD={0.46900690208107676,7.515459108840659},length_median=2.907826928010401,length_range={0.27156956952988764,16.054385878549766},posterior=1.0,rate=0.0024276967470258934,rate_95%_HPD={3.852748188594508E-4,0.005328537615127421},rate_median=0.0019990400453446366,rate_range={3.5130138558650044E-4,0.017540270705704256}]:3.4204458843484886,(39[&height=2.306920037733712E-14,height_95%_HPD={0.0,5.6843418860808015E-14},height_median=2.8421709430404007E-14,height_range={0.0,8.526512829121202E-14},length=21.70224866367145,length_95%_HPD={13.288776775529058,31.106379078014456},length_median=21.379034134631958,length_range={8.517580391930196,36.1134270097789},rate=0.0030148164862322307,rate_95%_HPD={0.0018547873293686928,0.004455794512419792},rate_median=0.002908970952714642,rate_range={0.001646300590067048,0.007622121137836465}]:21.702248663671448,101[&height=2.306920037733712E-14,height_95%_HPD={0.0,5.6843418860808015E-14},height_median=2.8421709430404007E-14,height_range={0.0,8.526512829121202E-14},length=21.70224866367145,length_95%_HPD={13.288776775529058,31.106379078014456},length_median=21.379034134631958,length_range={8.517580391930196,36.1134270097789},rate=0.001296103650678524,rate_95%_HPD={7.886344753052507E-4,0.0018962573775203263},rate_median=0.0012474826144824793,rate_range={6.847192820957142E-4,0.003203411720450228}]:21.702248663671448)[&height=21.70224866367147,height_95%_HPD={13.288776775529087,31.106379078014456},height_median=21.379034134632,height_range={8.517580391930224,36.11342700977893},length=5.0828567594613245,length_95%_HPD={0.700711232773358,11.361790086021983},length_median=4.375260948641646,length_range={0.45146570434108924,24.830371863464393},posterior=1.0,rate=0.0016229473103460254,rate_95%_HPD={2.8699854237867865E-4,0.0037844070920810633},rate_median=0.0013138519489144956,rate_range={2.563380172597583E-4,0.014785548038639176}]:5.082856759461215)[&height=26.785105423132684,height_95%_HPD={19.398966556134894,34.39455836817389},height_median=26.658359259377917,height_range={15.533612908385635,39.00757043384843},length=3.8680661907901404,length_95%_HPD={0.7652810327157624,8.742249394665677},length_median=3.362377096286032,length_range={0.24127513096647135,14.246147728751353},posterior=1.0,rate=0.0022751579650789637,rate_95%_HPD={4.577027452557418E-4,0.005063278563540115},rate_median=0.001907076089054513,rate_range={3.8032694544350653E-4,0.015211696537268235}]:3.868066190790323,37[&height=2.3117497964334008E-14,height_95%_HPD={0.0,5.6843418860808015E-14},height_median=2.8421709430404007E-14,height_range={0.0,5.6843418860808015E-14},length=30.653171613922993,length_95%_HPD={23.21960572898368,38.195497060324854},length_median=30.865657727916407,length_range={18.81867059630582,41.04794148662192},rate=0.0021346670035139806,rate_95%_HPD={0.0016119408376697774,0.0027409518781049383},rate_median=0.0020853343691132706,rate_range={0.001467208564513374,0.0035567819277888646}]:30.653171613922982)[&height=30.653171613923007,height_95%_HPD={23.21960572898371,38.195497060324854},height_median=30.86565772791645,height_range={18.81867059630585,41.04794148662195},length=12.853710123751904,length_95%_HPD={3.9465558186160905,23.672859416042385},length_median=12.447555209579157,length_range={3.9439958547536946,32.17664449084714},posterior=1.0,rate=0.00858187620561791,rate_95%_HPD={0.0027571664294243356,0.017786755587119625},rate_median=0.006989291294846104,rate_range={0.0026046488403335165,0.022029100674438837}]:12.853710123751874,(((((3[&height=2.3410124520844565E-14,height_95%_HPD={0.0,5.6843418860808015E-14},height_median=2.8421709430404007E-14,height_range={0.0,8.526512829121202E-14},length=9.95477720711877,length_95%_HPD={4.57932553832228,15.17140403621903},length_median=9.745323713981733,length_range={3.290127144663529,19.120793023571224},rate=0.00344578140316293,rate_95%_HPD={0.001813542652687632,0.005644022526013073},rate_median=0.003239464037299157,rate_range={0.001481664980224353,0.009700722618430031}]:9.95477720711877,36[&height=2.3410124520844565E-14,height_95%_HPD={0.0,5.6843418860808015E-14},height_median=2.8421709430404007E-14,height_range={0.0,8.526512829121202E-14},length=9.95477720711877,length_95%_HPD={4.57932553832228,15.17140403621903},length_median=9.745323713981733,length_range={3.290127144663529,19.120793023571224},rate=0.004244907588481571,rate_95%_HPD={0.002126586524354691,0.006825523904370943},rate_median=0.004004292060157958,rate_range={0.0017835253635685375,0.01250888406298963}]:9.95477720711877)[&height=9.954777207118793,height_95%_HPD={4.57932553832228,15.171404036219059},height_median=9.745323713981762,height_range={3.2901271446635576,19.120793023571252},length=12.626149706602751,length_95%_HPD={5.9530214541527755,19.471907642216678},length_median=12.225947124338937,length_range={4.381227894456515,25.77711500192092},posterior=1.0,rate=0.00739376083501907,rate_95%_HPD={0.0037490028889422423,0.011590553413518966},rate_median=0.00705665686640879,rate_range={0.0034501746364158496,0.01928386385882155}]:12.626149706602709,(6[&height=2.3341939692143074E-14,height_95%_HPD={0.0,5.6843418860808015E-14},height_median=2.8421709430404007E-14,height_range={0.0,8.526512829121202E-14},length=18.169273748272126,length_95%_HPD={11.506113061666227,24.886219484022632},length_median=17.958620280993458,length_range={8.16505216470523,29.401615675852398},rate=0.0028795847747662036,rate_95%_HPD={0.0018819714689810518,0.004051076826638238},rate_median=0.0028091512333566094,rate_range={0.0016273086372235468,0.006017376555157241}]:18.16927374827211,14[&height=2.3341939692143074E-14,height_95%_HPD={0.0,5.6843418860808015E-14},height_median=2.8421709430404007E-14,height_range={0.0,8.526512829121202E-14},length=18.169273748272126,length_95%_HPD={11.506113061666227,24.886219484022632},length_median=17.958620280993458,length_range={8.16505216470523,29.401615675852398},rate=0.0026245623842914866,rate_95%_HPD={0.0016613696078933136,0.0036720433660294423},rate_median=0.0025681442749062935,rate_range={0.0014244909943469373,0.005458206485957463}]:18.16927374827211)[&height=18.169273748272136,height_95%_HPD={11.506113061666255,24.88621948402266},height_median=17.958620280993472,height_range={8.16505216470523,29.401615675852426},length=4.411653165449448,length_95%_HPD={0.6049502913545695,9.570840406906882},length_median=3.8675831880246534,length_range={0.3016787431288037,16.92746410663925},posterior=1.0,rate=0.002023682369145545,rate_95%_HPD={4.1605389676606575E-4,0.004484154623474705},rate_median=0.0016611874118056197,rate_range={3.819288741315782E-4,0.0159143247374065}]:4.411653165449366)[&height=22.580926913721502,height_95%_HPD={16.523613390335726,29.00339463335858},height_median=22.336104040339627,height_range={13.859971631921496,32.8985508209486},length=5.269030117884101,length_95%_HPD={1.1419255057256095,10.233012291765803},length_median=4.891575579602872,length_range={0.5316770600907432,18.0637826049214},posterior=1.0,rate=0.003661285541923078,rate_95%_HPD={0.0010202592733966414,0.007871531957374766},rate_median=0.0031049989589267447,rate_range={7.386261196384546E-4,0.02293893995167043}]:5.269030117884256,(10[&height=2.3543653143718313E-14,height_95%_HPD={0.0,5.6843418860808015E-14},height_median=2.8421709430404007E-14,height_range={0.0,8.526512829121202E-14},length=17.971659350401463,length_95%_HPD={9.833844743970033,26.41014065144296},length_median=17.582976437168625,length_range={5.193894684038639,33.14454162164188},rate=0.004320679899613473,rate_95%_HPD={0.00233655490985993,0.006574019690454212},rate_median=0.0041541815067400786,rate_range={0.0020202807546154875,0.013202816243540224}]:17.971659350401456,12[&height=2.3543653143718313E-14,height_95%_HPD={0.0,5.6843418860808015E-14},height_median=2.8421709430404007E-14,height_range={0.0,8.526512829121202E-14},length=17.971659350401463,length_95%_HPD={9.833844743970033,26.41014065144296},length_median=17.582976437168625,length_range={5.193894684038639,33.14454162164188},rate=0.004839206609584354,rate_95%_HPD={0.0026738813703923,0.007261491015587045},rate_median=0.0046536272339859856,rate_range={0.0022219972589718018,0.01519734986329567}]:17.971659350401456)[&height=17.97165935040148,height_95%_HPD={9.833844743970062,26.41014065144296},height_median=17.582976437168654,height_range={5.193894684038668,33.144541621641906},length=9.878297681204138,length_95%_HPD={3.289490306543854,16.523836571690637},length_median=9.662327651399522,length_range={1.8036563467387765,24.75293947855198},posterior=1.0,rate=0.004692242567196362,rate_95%_HPD={0.0019736165838598633,0.00857640986295028},rate_median=0.004164937823151131,rate_range={0.00149117657995767,0.020923028617084154}]:9.878297681204277)[&height=27.849957031605758,height_95%_HPD={21.218280242670943,34.97394952473714},height_median=27.645567270239354,height_range={18.534093942436556,39.748751274199066},length=3.2237158216875774,length_95%_HPD={0.649887314996505,6.3734237705101435},length_median=2.9181539578540807,length_range={0.3660340570584566,12.960733234862872},posterior=1.0,rate=0.0035188800116921944,rate_95%_HPD={8.763349623724723E-4,0.007488082209290321},rate_median=0.0030270708021558645,rate_range={7.107632792065102E-4,0.017825842144223017}]:3.2271954842700588,(34[&height=2.3367509002906135E-14,height_95%_HPD={0.0,5.6843418860808015E-14},height_median=2.8421709430404007E-14,height_range={0.0,5.6843418860808015E-14},length=24.293563906083314,length_95%_HPD={14.573975008516655,33.66608567118848},length_median=24.406512965156722,length_range={9.652293712341674,38.62935910331274},rate=0.0014423622790865995,rate_95%_HPD={8.852597519117851E-4,0.002154756250003572},rate_median=0.001373597416355227,rate_range={8.020466902426269E-4,0.003448291101728954}]:24.29356390608331,35[&height=2.3367509002906135E-14,height_95%_HPD={0.0,5.6843418860808015E-14},height_median=2.8421709430404007E-14,height_range={0.0,5.6843418860808015E-14},length=24.293563906083314,length_95%_HPD={14.573975008516655,33.66608567118848},length_median=24.406512965156722,length_range={9.652293712341674,38.62935910331274},rate=0.0024589168148212584,rate_95%_HPD={0.0015831610091535879,0.003714518511334435},rate_median=0.002329502888443236,rate_range={0.0013771782654910158,0.005501552647836024}]:24.29356390608331)[&height=24.293563906083335,height_95%_HPD={14.573975008516683,33.66608567118851},height_median=24.40651296515675,height_range={9.652293712341702,38.62935910331274},length=6.773786689662857,length_95%_HPD={0.8933070538506627,14.276458977390476},length_median=5.987655427055607,length_range={0.5668323170249465,20.33888343704632},posterior=1.0,rate=0.0017442710392029696,rate_95%_HPD={3.865269344454793E-4,0.004180117099732387},rate_median=0.001369932558024642,rate_range={3.349596178509626E-4,0.012097730926179011}]:6.783588609792481)[&height=31.077152515875817,height_95%_HPD={23.591513717746608,38.04219197202961},height_median=30.878317864669356,height_range={21.05073685663369,43.52843516127206},length=2.7941117619965294,length_95%_HPD={0.4799320489105696,5.836337381443968},length_median=2.467326240038986,length_range={0.29694597465281447,14.2160663534072},posterior=0.9978008796481408,rate=0.0028791110675569186,rate_95%_HPD={5.211598077623717E-4,0.006417597211836988},rate_median=0.0024172900883350644,rate_range={3.824196485208538E-4,0.015987419408386716}]:2.7844875113726104,(30[&height=2.319704693115241E-14,height_95%_HPD={0.0,5.6843418860808015E-14},height_median=2.8421709430404007E-14,height_range={0.0,8.526512829121202E-14},length=7.881452978628312,length_95%_HPD={1.7665881545169952,16.058676429992246},length_median=7.00671756677302,length_range={0.9972519119944252,27.091068616468192},rate=0.0026232039332258436,rate_95%_HPD={6.443416211943267E-4,0.005308863168513324},rate_median=0.002296094504693029,rate_range={5.647257835525041E-4,0.013995072319087438}]:7.881452978628309,31[&height=2.319704693115241E-14,height_95%_HPD={0.0,5.6843418860808015E-14},height_median=2.8421709430404007E-14,height_range={0.0,8.526512829121202E-14},length=7.881452978628312,length_95%_HPD={1.7665881545169952,16.058676429992246},length_median=7.00671756677302,length_range={0.9972519119944252,27.091068616468192},rate=0.0016093072407160596,rate_95%_HPD={4.208064816769719E-4,0.0032387413992293686},rate_median=0.0014207781437787054,rate_range={3.242973248440742E-4,0.011022480610440817}]:7.881452978628309)[&height=7.8814529786283325,height_95%_HPD={1.7665881545169952,16.058676429992246},height_median=7.006717566773034,height_range={0.9972519119944252,27.09106861646822},length=25.973864791073204,length_95%_HPD={15.09188118459926,37.18806251004219},length_median=26.01236422173693,length_range={7.2361080360206245,42.684830959850956},posterior=1.0,rate=0.0013982931968437341,rate_95%_HPD={8.337519806348544E-4,0.0021150495225834656},rate_median=0.00132177435594828,rate_range={7.471617263455447E-4,0.004491076611220207}]:25.980187048620095)[&height=33.86164002724843,height_95%_HPD={26.51490006197824,41.19531113226333},height_median=33.70874415944989,height_range={24.282999348854887,46.59136615468799},length=9.645241710426331,length_95%_HPD={3.3852016717257243,16.800154373717263},length_median=9.132320455437728,length_range={2.8565844512896064,24.955060043698865},posterior=1.0,rate=0.006171030493364354,rate_95%_HPD={0.002533599853428616,0.012764210153797617},rate_median=0.005549229770152784,rate_range={0.0019528686640639791,0.017500923003582077}]:9.645241710426454)[&height=43.50688173767488,height_95%_HPD={34.38485911573903,52.45459095378095},height_median=43.25554753542173,height_range={31.01810708849922,61.72509163029497},length=9.741703562007416,length_95%_HPD={3.8273169930562716,15.754392511224793},length_median=9.304631131104763,length_range={3.5446640183058093,22.99881920816898},posterior=1.0,rate=0.006847436833435241,rate_95%_HPD={0.002843033042876618,0.011684692514608627},rate_median=0.006343285406698889,rate_range={0.0025378130534308685,0.015898256837118808}]:9.741703562007388,(20[&height=2.3160113482272438E-14,height_95%_HPD={0.0,2.8421709430404007E-14},height_median=2.8421709430404007E-14,height_range={0.0,5.6843418860808015E-14},length=16.1788060687978,length_95%_HPD={6.629407214843582,26.71979854079123},length_median=15.702481033287171,length_range={4.721127316610534,33.15271962531949},rate=0.0030451170831153787,rate_95%_HPD={0.0013193107576142966,0.00530464815359686},rate_median=0.002767096693575273,rate_range={0.0012848616770533118,0.010400553984283719}]:16.178806068797805,53[&height=2.3160113482272438E-14,height_95%_HPD={0.0,2.8421709430404007E-14},height_median=2.8421709430404007E-14,height_range={0.0,5.6843418860808015E-14},length=16.1788060687978,length_95%_HPD={6.629407214843582,26.71979854079123},length_median=15.702481033287171,length_range={4.721127316610534,33.15271962531949},rate=0.0021951007946875494,rate_95%_HPD={9.626979556361639E-4,0.003908214201815197},rate_median=0.002002089859177691,rate_range={8.167036775891096E-4,0.006779577808902302}]:16.178806068797805)[&height=16.17880606879783,height_95%_HPD={6.629407214843582,26.71979854079123},height_median=15.7024810332872,height_range={4.721127316610563,33.15271962531952},length=37.06977923088448,length_95%_HPD={22.668224291089913,49.52067533976572},length_median=37.021256992239685,length_range={18.907025275253886,58.090718244244655},posterior=1.0,rate=0.0035077251486235585,rate_95%_HPD={0.0023065330420935963,0.004947680224455719},rate_median=0.0033912945911706537,rate_range={0.0020396515097492614,0.006396124188010808}]:37.069779230884436)[&height=53.24858529968227,height_95%_HPD={43.88348008487935,64.39198260393711},height_median=52.57871852017313,height_range={39.294983644541745,72.8128655468799},length=4.923729985705427,length_95%_HPD={1.5033016022710797,9.892594992826446},length_median=4.484843313427504,length_range={0.6280380184955447,14.544142910082314},posterior=1.0,rate=0.003468653219686733,rate_95%_HPD={0.0010319052733928824,0.006832598738280077},rate_median=0.0030718863268559583,rate_range={8.161597652364945E-4,0.01804333462789431}]:4.923729985705286,(8[&height=2.337603210649382E-14,height_95%_HPD={0.0,2.8421709430404007E-14},height_median=2.8421709430404007E-14,height_range={0.0,5.6843418860808015E-14},length=42.53795861940084,length_95%_HPD={29.858321224559162,55.12799309315966},length_median=43.076499130433334,length_range={23.089929133441828,61.85991152022868},rate=0.003251378861519247,rate_95%_HPD={0.002278036240319678,0.004451173052663547},rate_median=0.0031334226174536874,rate_range={0.0020470455446267495,0.006256955990031115}]:42.53795861940085,((16[&height=2.3202729000210868E-14,height_95%_HPD={0.0,5.6843418860808015E-14},height_median=2.8421709430404007E-14,height_range={0.0,5.6843418860808015E-14},length=22.089977254982042,length_95%_HPD={12.159759409710773,33.77011640197159},length_median=21.4425703500087,length_range={9.05456855256918,45.869861770158735},rate=0.003876086444980627,rate_95%_HPD={0.0021741734541255242,0.006073853071908937},rate_median=0.0037183658413481495,rate_range={0.0017265098669809558,0.008708168442210881}]:22.089977254982042,(29[&height=2.315727244774321E-14,height_95%_HPD={0.0,5.6843418860808015E-14},height_median=2.8421709430404007E-14,height_range={0.0,5.6843418860808015E-14},length=18.051121885395712,length_95%_HPD={8.116357133195777,29.097156879050203},length_median=17.480958831496032,length_range={4.92244367129058,38.15803828339871},rate=0.0036070914215394424,rate_95%_HPD={0.0017050165034272367,0.006013928930204853},rate_median=0.0033692177647306875,rate_range={0.0014952131041209092,0.01156109224774705}]:18.051121885395716,76[&height=2.315727244774321E-14,height_95%_HPD={0.0,5.6843418860808015E-14},height_median=2.8421709430404007E-14,height_range={0.0,5.6843418860808015E-14},length=18.051121885395712,length_95%_HPD={8.116357133195777,29.097156879050203},length_median=17.480958831496032,length_range={4.92244367129058,38.15803828339871},rate=0.0031874548690283334,rate_95%_HPD={0.0015407193783276948,0.00533642731361337},rate_median=0.0029924447475070705,rate_range={0.0012979848966620678,0.01017682174785511}]:18.051121885395716)[&height=18.05112188539574,height_95%_HPD={8.116357133195805,29.09715687905023},height_median=17.480958831496032,height_range={4.922443671290608,38.15803828339874},length=4.0388553695863605,length_95%_HPD={0.5481785138994155,9.431957459058893},length_median=3.3934918315186096,length_range={0.19340074868337354,21.169196760202894},posterior=1.0,rate=0.0020210584486409114,rate_95%_HPD={3.1588279498791504E-4,0.004684630602942706},rate_median=0.0016346425052366658,rate_range={2.8238135250665495E-4,0.01556476085294901}]:4.038855369586326)[&height=22.089977254982067,height_95%_HPD={12.159759409710801,33.77011640197162},height_median=21.44257035000873,height_range={9.054568552569208,45.869861770158764},length=7.018457944710735,length_95%_HPD={1.3141437356981527,15.176671174842312},length_median=5.991810704122642,length_range={0.7939051853500132,32.3893771189106},posterior=1.0,rate=0.002222097864595186,rate_95%_HPD={4.4431223041371945E-4,0.004809131679085799},rate_median=0.0018989768950669382,rate_range={3.655744106768501E-4,0.012892159200596548}]:7.018457944710686,24[&height=2.3316370381380016E-14,height_95%_HPD={0.0,5.6843418860808015E-14},height_median=2.8421709430404007E-14,height_range={0.0,5.6843418860808015E-14},length=29.10843519969273,length_95%_HPD={17.516558018491835,42.41717268517364},length_median=28.433400564764987,length_range={14.1038935882585,51.60940506921315},rate=0.0023968559615609227,rate_95%_HPD={0.0014508575220062527,0.0035142302372602203},rate_median=0.0023265242319058486,rate_range={0.0012074043748730456,0.0050622376272714325}]:29.108435199692728)[&height=29.108435199692753,height_95%_HPD={17.516558018491835,42.41717268517367},height_median=28.433400564764987,height_range={14.10389358825853,51.60940506921318},length=13.429523419708108,length_95%_HPD={4.602892349656429,23.363401045806},length_median=12.883819455241031,length_range={2.866305347873052,32.18538127614386},posterior=1.0,rate=0.0049069157855811405,rate_95%_HPD={0.00202962451635554,0.009247197356462995},rate_median=0.00438251825730147,rate_range={0.0016189023331161477,0.018145339398468782}]:13.429523419708115)[&height=42.53795861940087,height_95%_HPD={29.85832122455919,55.12799309315969},height_median=43.07649913043339,height_range={23.089929133441856,61.85991152022868},length=15.634356665986829,length_95%_HPD={5.0323064973416365,26.70567397195633},length_median=14.92900737356549,length_range={3.4234520238920965,34.50399934532503},posterior=1.0,rate=0.004689221687078884,rate_95%_HPD={0.0019334746441435505,0.008969785096671751},rate_median=0.004242533826349252,rate_range={0.0015987235932464845,0.0162823294474578}]:15.634356665986687)[&height=58.172315285387555,height_95%_HPD={48.17639659027529,68.45499171536065},height_median=57.54483230886317,height_range={44.549783248398455,79.75886598036853},length=5.156417294564088,length_95%_HPD={1.2181552557556472,9.869670070726059},length_median=4.747594033476794,length_range={0.7818795164261019,18.362073654972846},posterior=1.0,rate=0.004597333861430534,rate_95%_HPD={0.0012231538404054545,0.009945735508431225},rate_median=0.0039369811878309496,rate_range={9.800469542770402E-4,0.019165102526555317}]:5.1564172945643065,(7[&height=2.3202729000210868E-14,height_95%_HPD={0.0,2.8421709430404007E-14},height_median=2.8421709430404007E-14,height_range={0.0,5.6843418860808015E-14},length=54.038849291345876,length_95%_HPD={40.18954343456775,67.82777934070957},length_median=54.3630837627251,length_range={26.79903895979217,82.23502598569401},rate=0.001925427948100668,rate_95%_HPD={0.0014674706844907133,0.0025045497116969323},rate_median=0.0018829756947309178,rate_range={0.0012323315841550784,0.0038530886952725164}]:54.03884929134588,70[&height=2.3202729000210868E-14,height_95%_HPD={0.0,2.8421709430404007E-14},height_median=2.8421709430404007E-14,height_range={0.0,5.6843418860808015E-14},length=54.038849291345876,length_95%_HPD={40.18954343456775,67.82777934070957},length_median=54.3630837627251,length_range={26.79903895979217,82.23502598569401},rate=0.0016926537928266332,rate_95%_HPD={0.0013043635910846515,0.0022261643457666402},rate_median=0.0016502157274034712,rate_range={0.0010834013057366882,0.003463905942096403}]:54.03884929134588)[&height=54.038849291345905,height_95%_HPD={40.18954343456775,67.8277793407096},height_median=54.363083762725125,height_range={26.7990389597922,82.23502598569404},length=9.289883288605795,length_95%_HPD={1.1435329692336609,21.69539635869151},length_median=7.98690193867747,length_range={0.8287926040868854,34.534810345156174},posterior=1.0,rate=0.001638973882229382,rate_95%_HPD={2.6767982881816226E-4,0.004080581021294291},rate_median=0.001258174238691187,rate_range={2.503467256408184E-4,0.009789040183151106}]:9.289883288605957)[&height=63.32873257995186,height_95%_HPD={53.637516721621154,74.09231138378352},height_median=62.815464494419075,height_range={50.155267263886,85.8346180919804},length=8.234878891445025,length_95%_HPD={3.84831632457103,13.066913095053621},length_median=7.862206406421393,length_range={3.5603022463728564,20.537448560563348},posterior=1.0,rate=0.007774615197786925,rate_95%_HPD={0.0037254923480433792,0.012838201774361225},rate_median=0.007405017062024428,rate_range={0.002924175883687018,0.015088379557308773}]:8.234878891444893,59[&height=2.315727244774321E-14,height_95%_HPD={0.0,2.8421709430404007E-14},height_median=2.8421709430404007E-14,height_range={0.0,5.6843418860808015E-14},length=71.56361147139674,length_95%_HPD={60.83362085394626,82.85447812180078},length_median=70.97175154012791,length_range={56.81643852167227,91.73320003567922},rate=9.751645815283819E-4,rate_95%_HPD={8.111822836624354E-4,0.0011317723290805934},rate_median=9.755846911176055E-4,rate_range={7.338698509384113E-4,0.0012817059636430247}]:71.56361147139673)[&height=71.56361147139675,height_95%_HPD={60.833620853946286,82.85447812180081},height_median=70.97175154012794,height_range={56.816438521672296,91.73320003567925},length=5.882155000663729,length_95%_HPD={1.76469648603927,11.509596482612153},length_median=5.321260437359136,length_range={1.2235541206391076,18.2453431838957},posterior=1.0,rate=0.004205380046622248,rate_95%_HPD={0.0012915090781719409,0.008089838947792789},rate_median=0.0037748403404036426,rate_range={0.001077827319400549,0.015925025608995964}]:5.882155000663886,(19[&height=2.3143067275097065E-14,height_95%_HPD={0.0,2.8421709430404007E-14},height_median=2.8421709430404007E-14,height_range={0.0,5.6843418860808015E-14},length=32.641342939288315,length_95%_HPD={13.900271290719473,57.581620735646},length_median=30.558375772342096,length_range={10.263771110892762,65.90845820483267},rate=0.0015975699909743669,rate_95%_HPD={6.909302243135868E-4,0.002835009447183244},rate_median=0.001486132828592262,rate_range={6.492147360034489E-4,0.004441119633167979}]:32.641342939288315,69[&height=2.3143067275097065E-14,height_95%_HPD={0.0,2.8421709430404007E-14},height_median=2.8421709430404007E-14,height_range={0.0,5.6843418860808015E-14},length=32.641342939288315,length_95%_HPD={13.900271290719473,57.581620735646},length_median=30.558375772342096,length_range={10.263771110892762,65.90845820483267},rate=0.0013965676730136867,rate_95%_HPD={6.019218513747837E-4,0.0024593383611604585},rate_median=0.0012899555602880173,rate_range={5.563761781627184E-4,0.0039029313975962737}]:32.641342939288315)[&height=32.641342939288336,height_95%_HPD={13.900271290719502,57.58162073564603},height_median=30.558375772342124,height_range={10.263771110892762,65.9084582048327},length=44.8044235327724,length_95%_HPD={16.949225822724472,68.78955721675867},length_median=46.38354917362629,length_range={5.176647486967099,73.36540436453343},posterior=1.0,rate=6.430191849501648E-4,rate_95%_HPD={3.0508469507539894E-4,0.0013026702067420457},rate_median=5.248959510690998E-4,rate_range={2.934024620863934E-4,0.004540384290489571}]:44.804423532772304)[&height=77.44576647206064,height_95%_HPD={68.22003564423667,88.58776044285682},height_median=77.0473969599019,height_range={62.59807633771691,96.05714877747232},length=3.596851028766262,length_95%_HPD={0.6494158081317636,7.582905338189704},length_median=3.2103000839768754,length_range={0.3742025363211354,16.132908870002595},posterior=1.0,rate=0.002841779710679355,rate_95%_HPD={5.227307743885108E-4,0.006381685259264761},rate_median=0.0023306582682127773,rate_range={4.26958594156775E-4,0.017957929491893954}]:3.5968510287663946)[&height=81.04261750082703,height_95%_HPD={73.10003855863862,91.04871817556004},height_median=80.53322063719958,height_range={68.85489291353088,99.92149674689638},length=3.8274423567487053,length_95%_HPD={0.7944778590273955,7.729292790058565},length_median=3.4301645813899455,length_range={0.5101862682575415,13.760785946173328},posterior=1.0,rate=0.0030106438929068053,rate_95%_HPD={7.88896573847748E-4,0.006416997137768365},rate_median=0.0025709361874910793,rate_range={6.623571274261729E-4,0.016172314451388073}]:3.827442356748719,28[&height=2.3162954516801665E-14,height_95%_HPD={0.0,2.8421709430404007E-14},height_median=2.8421709430404007E-14,height_range={0.0,5.6843418860808015E-14},length=84.87005985757574,length_95%_HPD={75.97723162505143,94.8732033680419},length_median=84.4380822401371,length_range={70.46279981030074,103.3322415535162},rate=5.612105190604259E-4,rate_95%_HPD={4.7669240258892354E-4,6.467318997753401E-4},rate_median=5.602996293761415E-4,rate_range={4.2583159777662464E-4,7.323414988667636E-4}]:84.87005985757573)[&height=84.87005985757575,height_95%_HPD={75.97723162505146,94.87320336804193},height_median=84.43808224013712,height_range={70.46279981030077,103.3322415535162},length=5.585692152031935,length_95%_HPD={1.3299491437924758,10.73273671574681},length_median=5.126123089018137,length_range={0.8885881023676205,17.82921224185371},posterior=1.0,rate=0.0037646260931520847,rate_95%_HPD={9.609401425562352E-4,0.007745939942251672},rate_median=0.003274675774648915,rate_range={7.813412191605407E-4,0.015676409732341703}]:5.585692152032024,18[&height=2.3162954516801665E-14,height_95%_HPD={0.0,2.8421709430404007E-14},height_median=2.8421709430404007E-14,height_range={0.0,5.6843418860808015E-14},length=90.45575200960778,length_95%_HPD={80.83158923235797,100.00236406157734},length_median=90.27815923682343,length_range={76.05872864880958,106.72467322063663},rate=9.521019376123968E-4,rate_95%_HPD={8.306529703101637E-4,0.0010835747867756456},rate_median=9.503938216837776E-4,rate_range={7.583202396198418E-4,0.0012065489729846013}]:90.45575200960775)[&height=90.45575200960778,height_95%_HPD={80.831589232358,100.00236406157737},height_median=90.27815923682346,height_range={76.0587286488096,106.72467322063666},length=10.314681369658853,length_95%_HPD={3.1209919910889994,18.684949630001128},length_median=9.82946939732659,length_range={2.671463674960151,27.041985219329774},posterior=1.0,rate=0.00571075195179874,rate_95%_HPD={0.0020387541024244867,0.01112667902641264},rate_median=0.004965312245358646,rate_range={0.0018817794699480031,0.016107633528157875}]:10.3146813696588,((((((13[&height=2.3123180033392465E-14,height_95%_HPD={0.0,2.8421709430404007E-14},height_median=2.8421709430404007E-14,height_range={0.0,5.6843418860808015E-14},length=20.44724549300637,length_95%_HPD={5.831888176051336,37.77447350705697},length_median=19.18649302312697,length_range={3.068293357247086,51.947248942346576},rate=0.0015775760445767564,rate_95%_HPD={5.334940750953911E-4,0.0032516438552355207},rate_median=0.0013729376046159316,rate_range={4.298202152764846E-4,0.008510886843846727}]:20.447245493006378,98[&height=2.3123180033392465E-14,height_95%_HPD={0.0,2.8421709430404007E-14},height_median=2.8421709430404007E-14,height_range={0.0,5.6843418860808015E-14},length=20.44724549300637,length_95%_HPD={5.831888176051336,37.77447350705697},length_median=19.18649302312697,length_range={3.068293357247086,51.947248942346576},rate=0.0027222786108698285,rate_95%_HPD={9.232300884577003E-4,0.005633145600150737},rate_median=0.002359139786290434,rate_range={8.509274352744336E-4,0.014776036195589913}]:20.447245493006378)[&height=20.447245493006402,height_95%_HPD={5.831888176051336,37.774473507056996},height_median=19.186493023127,height_range={3.068293357247086,51.947248942346604},length=38.09923212718358,length_95%_HPD={16.12834416800203,55.5578640937283},length_median=39.29796556316694,length_range={8.797328402911205,66.28486709199315},posterior=1.0,rate=0.0010862985419223786,rate_95%_HPD={5.69127509159369E-4,0.001988719137674199},rate_median=9.507639881727666E-4,rate_range={4.7968404583049947E-4,0.003946032547912834}]:38.099232127183214,65[&height=2.3188523827564726E-14,height_95%_HPD={0.0,2.8421709430404007E-14},height_median=2.8421709430404007E-14,height_range={0.0,5.6843418860808015E-14},length=58.54647762018959,length_95%_HPD={45.34665294283977,68.49964069764614},length_median=59.46208273214003,length_range={35.02044226123641,77.09312991777445},rate=0.0013615092263040338,rate_95%_HPD={0.0011114390927271119,0.001696041807234437},rate_median=0.00132873597523266,rate_range={9.884424947074896E-4,0.0023051728026997845}]:58.5464776201896)[&height=58.54647762018962,height_95%_HPD={45.34665294283977,68.49964069764617},height_median=59.46208273214006,height_range={35.02044226123644,77.09312991777448},length=10.039841276827207,length_95%_HPD={1.6527900508351792,21.662013627090275},length_median=8.797111740511248,length_range={1.0078435677209683,33.62071461794582},posterior=1.0,rate=0.0012765261939801686,rate_95%_HPD={2.624261291021734E-4,0.0030291531255606115},rate_median=0.0010223395491620868,rate_range={2.468003509245285E-4,0.007014853022196824}]:10.039841276827403,(((15[&height=2.323966244909084E-14,height_95%_HPD={0.0,2.8421709430404007E-14},height_median=2.8421709430404007E-14,height_range={0.0,8.526512829121202E-14},length=20.321646906047295,length_95%_HPD={7.4213716322907,36.277393432093035},length_median=18.936374593570854,length_range={5.320861970044518,48.93656897810388},rate=0.0018066254369175476,rate_95%_HPD={7.05577933183234E-4,0.003263841741262927},rate_median=0.0016542861083569142,rate_range={5.718194398158003E-4,0.005684380127112617}]:20.321646906047302,85[&height=2.323966244909084E-14,height_95%_HPD={0.0,2.8421709430404007E-14},height_median=2.8421709430404007E-14,height_range={0.0,8.526512829121202E-14},length=20.321646906047295,length_95%_HPD={7.4213716322907,36.277393432093035},length_median=18.936374593570854,length_range={5.320861970044518,48.93656897810388},rate=0.0014785934815252416,rate_95%_HPD={5.727785076262067E-4,0.002660801736272386},rate_median=0.0013549485177779168,rate_range={5.192592804078022E-4,0.004968454483109933}]:20.321646906047302)[&height=20.321646906047327,height_95%_HPD={7.421371632290715,36.277393432093035},height_median=18.936374593570882,height_range={5.320861970044547,48.93656897810388},length=32.38645890968055,length_95%_HPD={13.442050156943367,50.692549804120034},length_median=32.76857167468605,length_range={7.815177142243101,63.25114918648815},posterior=1.0,rate=0.0012160427309956354,rate_95%_HPD={6.150668604135894E-4,0.0022318398949981947},rate_median=0.0010741829790506664,rate_range={5.57339888639071E-4,0.004923823465689544}]:32.38645890968044,60[&height=2.3143067275097065E-14,height_95%_HPD={0.0,2.8421709430404007E-14},height_median=2.8421709430404007E-14,height_range={0.0,5.6843418860808015E-14},length=52.70810581572776,length_95%_HPD={37.859571991918244,66.39563747544209},length_median=53.86527968177565,length_range={21.31760764296159,75.5780466484626},rate=0.001245929826752685,rate_95%_HPD={9.368990534768863E-4,0.0016910799925824547},rate_median=0.0011885673431461519,rate_range={8.581943381993024E-4,0.002922395970818129}]:52.70810581572775)[&height=52.70810581572777,height_95%_HPD={37.85957199191827,66.39563747544209},height_median=53.865279681775675,height_range={21.31760764296162,75.57804664846263},length=4.930575575184079,length_95%_HPD={0.44489829603280384,13.485335373070669},length_median=3.714121710377185,length_range={0.32531685961956214,24.336498373377395},posterior=1.0,rate=0.0016276075049930366,rate_95%_HPD={2.335943695083831E-4,0.003938976474826403},rate_median=0.0012807878099176885,rate_range={2.335943695083831E-4,0.011287075758655991}]:4.930575575184022,62[&height=2.3168636585860123E-14,height_95%_HPD={0.0,2.8421709430404007E-14},height_median=2.8421709430404007E-14,height_range={0.0,5.6843418860808015E-14},length=57.63868139091178,length_95%_HPD={44.48239475519733,68.42161187435734},length_median=58.73588871729184,length_range={36.36297383200646,77.00153328630262},rate=9.341274088274796E-4,rate_95%_HPD={7.562350811489887E-4,0.0011966726700976145},rate_median=9.089939470193669E-4,rate_range={6.558581240793542E-4,0.0014906090445927004}]:57.63868139091177)[&height=57.63868139091179,height_95%_HPD={44.48239475519736,68.42161187435737},height_median=58.73588871729187,height_range={36.36297383200646,77.00153328630262},length=10.94763750610507,length_95%_HPD={1.7416861235810472,23.107361808767337},length_median=9.691618466404456,length_range={1.0078059677080233,29.949007428239213},posterior=1.0,rate=0.0010970435439357305,rate_95%_HPD={2.831952261519567E-4,0.0028021374047916534},rate_median=8.268499762097102E-4,rate_range={2.688808481397721E-4,0.00791612021017906}]:10.947637506105231)[&height=68.58631889701702,height_95%_HPD={66.00142806550862,73.15521212701643},height_median=67.93798776456391,height_range={66.00142806550862,84.23603515053229},length=18.40440701091459,length_95%_HPD={7.99438609522069,27.95066552910336},length_median=18.39644144074709,length_range={4.065681853758292,34.014388147317945},posterior=1.0,rate=0.002784226120988174,rate_95%_HPD={0.0014192563465381552,0.0048021208773799135},rate_median=0.0025296663395677167,rate_range={0.0013346073810215503,0.011105664724748287}]:18.40440701091468,((((((17[&height=2.3316370381380016E-14,height_95%_HPD={0.0,5.6843418860808015E-14},height_median=2.8421709430404007E-14,height_range={0.0,8.526512829121202E-14},length=13.597611187393738,length_95%_HPD={7.0466043460459815,20.151456960233176},length_median=13.293420933878153,length_range={4.603820133067742,26.619429775555304},rate=0.001982833584724913,rate_95%_HPD={0.001060363584849839,0.003087278559463673},rate_median=0.0018922031506050548,rate_range={9.09444357207204E-4,0.004944487367740016}]:13.597611187393735,46[&height=2.3316370381380016E-14,height_95%_HPD={0.0,5.6843418860808015E-14},height_median=2.8421709430404007E-14,height_range={0.0,8.526512829121202E-14},length=13.597611187393738,length_95%_HPD={7.0466043460459815,20.151456960233176},length_median=13.293420933878153,length_range={4.603820133067742,26.619429775555304},rate=0.0015976341736490326,rate_95%_HPD={8.272494855678485E-4,0.0024357264293482284},rate_median=0.0015339106495275711,rate_range={6.757367415913445E-4,0.00423935035143333}]:13.597611187393735)[&height=13.597611187393758,height_95%_HPD={7.0466043460459815,20.151456960233205},height_median=13.293420933878181,height_range={4.603820133067771,26.619429775555332},length=3.3728608188096167,length_95%_HPD={0.3503202430964194,7.961876233377282},length_median=2.842233680963524,length_range={0.17599057898743808,12.739049849528726},posterior=1.0,rate=0.0016606895522625284,rate_95%_HPD={2.579243766732364E-4,0.004027514270863353},rate_median=0.001302077341676682,rate_range={2.3776029708375918E-4,0.011210690631826604}]:3.0847240412872505,(((47[&height=2.3162954516801665E-14,height_95%_HPD={0.0,5.6843418860808015E-14},height_median=2.8421709430404007E-14,height_range={0.0,8.526512829121202E-14},length=4.267285204764838,length_95%_HPD={1.3605982193409432,7.479592951883504},length_median=3.9803354386456675,length_range={1.0863195869128504,14.571178764749632},rate=0.003822361778663529,rate_95%_HPD={0.0014069080808889417,0.007091326652120267},rate_median=0.0035116881951382213,rate_range={9.79488276500032E-4,0.013189229093641881}]:4.26728520476484,79[&height=2.3162954516801665E-14,height_95%_HPD={0.0,5.6843418860808015E-14},height_median=2.8421709430404007E-14,height_range={0.0,8.526512829121202E-14},length=4.267285204764838,length_95%_HPD={1.3605982193409432,7.479592951883504},length_median=3.9803354386456675,length_range={1.0863195869128504,14.571178764749632},rate=0.0020432130098356573,rate_95%_HPD={7.28653182815387E-4,0.0038548407968425655},rate_median=0.0018727438389609865,rate_range={4.802933557105505E-4,0.006916610814743519}]:4.26728520476484)[&height=4.267285204764863,height_95%_HPD={1.3605982193409432,7.479592951883532},height_median=3.98033543864571,height_range={1.0863195869128504,14.57117876474966},length=7.384267028506389,length_95%_HPD={3.265797567913367,12.765454708550578},length_median=6.910724702837889,length_range={2.255916871129841,18.343230755708163},posterior=1.0,rate=0.0046020394484547235,rate_95%_HPD={0.0019220030150260176,0.007724052844482937},rate_median=0.004390734702177846,rate_range={0.001685447839925772,0.01413656464409106}]:7.384267028506396,94[&height=2.2893056236524938E-14,height_95%_HPD={0.0,5.6843418860808015E-14},height_median=2.8421709430404007E-14,height_range={0.0,8.526512829121202E-14},length=11.651552233271234,length_95%_HPD={6.680741772162861,17.933716338469424},length_median=11.143954791483054,length_range={5.6330305185678355,22.59919725778579},rate=0.003922162422037023,rate_95%_HPD={0.0020930603874748446,0.005894390669790024},rate_median=0.003847573151813201,rate_range={0.0018822246717279272,0.00781202861210807}]:11.651552233271236)[&height=11.651552233271259,height_95%_HPD={6.6807417721628894,17.933716338469424},height_median=11.143954791483083,height_range={5.6330305185678355,22.599197257785818},length=3.7889436731309027,length_95%_HPD={0.7589723866390159,7.319595569499214},length_median=3.5394480829884785,length_range={0.4272537625213033,11.515026648162333},posterior=1.0,rate=0.003294990308683571,rate_95%_HPD={8.495138246672906E-4,0.006566699574976676},rate_median=0.0028086527811825015,rate_range={8.02806505723037E-4,0.021424165449664415}]:3.6439228677004483,(93[&height=2.3128862102450923E-14,height_95%_HPD={0.0,5.6843418860808015E-14},height_median=2.8421709430404007E-14,height_range={0.0,8.526512829121202E-14},length=12.41690857076999,length_95%_HPD={6.254874111395452,18.704063651345464},length_median=12.268025615130071,length_range={3.681909690431553,25.232754806902776},rate=0.0014441268849972894,rate_95%_HPD={7.749651415978962E-4,0.0023419692245345946},rate_median=0.001361151049686737,rate_range={6.345019245880013E-4,0.004176811728754317}]:12.410857514492962,95[&height=2.3151590378684753E-14,height_95%_HPD={0.0,5.6843418860808015E-14},height_median=2.8421709430404007E-14,height_range={0.0,8.526512829121202E-14},length=12.406863200404452,length_95%_HPD={6.114326156390462,18.544519193667654},length_median=12.255591689325577,length_range={3.681909690431553,25.232754806902776},rate=0.0020376542203183997,rate_95%_HPD={0.0011108125330802213,0.0033217777467571203},rate_median=0.00192571543309531,rate_range={8.799502801018982E-4,0.006874613745356513}]:12.410857514492962)[&height=12.410857514492985,height_95%_HPD={6.254874111395452,18.704063651345493},height_median=12.261750476177362,height_range={3.6819096904315813,25.232754806902804},length=2.3626540734111083,length_95%_HPD={0.17661034680583043,5.9235418543611615},length_median=1.8892684025402176,length_range={0.11102529289274798,11.212999399009874},posterior=0.9924030387844862,rate=0.001686741727969407,rate_95%_HPD={2.952957892899171E-4,0.004074639776408328},rate_median=0.0013057285574439527,rate_range={2.7731090658118275E-4,0.01578967331265971}]:2.884617586478722)[&height=15.295475100971707,height_95%_HPD={10.313803690423384,20.864756536340096},height_median=14.874571609575376,height_range={9.549414164717916,26.57597579762995},length=1.4718639465258054,length_95%_HPD={0.191919079582064,3.512568256478417},length_median=1.253674120657216,length_range={0.0768744109384869,7.087429217861597},posterior=0.507796881247501,rate=0.0021968680314188764,rate_95%_HPD={3.6617094558392656E-4,0.005138801212263689},rate_median=0.001749399218928989,rate_range={3.20582896306651E-4,0.019833932358584532}]:1.3868601277093013)[&height=16.68233522868101,height_95%_HPD={11.446352020045865,22.65539107055777},height_median=16.252042748568584,height_range={10.341125898365817,29.62302184474767},length=3.1218549548668157,length_95%_HPD={0.5332855939869319,6.67680932477586},length_median=2.687480692545627,length_range={0.23740045903102214,13.709388496319647},posterior=0.6033586565373851,rate=0.002197287137032226,rate_95%_HPD={4.063423194315426E-4,0.00472954753463972},rate_median=0.0018616119593233814,rate_range={3.2612435834120606E-4,0.012737934531566478}]:2.304831906792689,80[&height=2.3040790032044832E-14,height_95%_HPD={0.0,2.8421709430404007E-14},height_median=2.8421709430404007E-14,height_range={0.0,5.6843418860808015E-14},length=17.38257441559368,length_95%_HPD={8.75950978936612,25.019407384258827},length_median=17.629450492045606,length_range={6.210767475525358,33.534425279176475},rate=0.0022092826698698414,rate_95%_HPD={0.001160436474938005,0.003745031604222589},rate_median=0.001980565956041372,rate_range={9.599248408561018E-4,0.006169995728141325}]:18.987167135473676)[&height=18.987167135473697,height_95%_HPD={12.30371949347277,25.119943685522614},height_median=18.830489909173743,height_range={10.247712420719893,33.5344252791765},length=21.472474368263274,length_95%_HPD={9.269277136092498,35.03444775506914},length_median=21.173457940340143,length_range={5.738226440698099,46.15815048503926},posterior=1.0,rate=0.004037048698170914,rate_95%_HPD={0.0018905071450400863,0.0068950905537737295},rate_median=0.003686234980762733,rate_range={0.0017344474951425022,0.012985820691566827}]:21.472474368263207,73[&height=2.321977520738624E-14,height_95%_HPD={0.0,2.8421709430404007E-14},height_median=2.8421709430404007E-14,height_range={0.0,5.6843418860808015E-14},length=40.459641503736876,length_95%_HPD={28.32660785854503,52.86968964445141},length_median=40.17000200572467,length_range={19.777629047546355,58.591712216387464},rate=0.0020739717142817947,rate_95%_HPD={0.0014797994596412478,0.0027665803485248802},rate_median=0.0020296997407579813,rate_range={0.0013453559017503655,0.0043275417431166896}]:40.45964150373688)[&height=40.459641503736904,height_95%_HPD={28.32660785854506,52.86968964445144},height_median=40.17000200572468,height_range={19.777629047546355,58.59171221638749},length=22.37595293470887,length_95%_HPD={7.526984296277078,39.493523004547455},length_median=21.8100388617462,length_range={4.581808885373206,53.16136465998099},posterior=1.0,rate=0.0041374318807904445,rate_95%_HPD={0.001652643746941638,0.007753301581016148},rate_median=0.003547986726981162,rate_range={0.001424701148841686,0.016559416971640213}]:22.37595293470875,(83[&height=2.308908761904172E-14,height_95%_HPD={0.0,2.8421709430404007E-14},height_median=2.8421709430404007E-14,height_range={0.0,5.6843418860808015E-14},length=45.681144694916846,length_95%_HPD={24.00668712296229,67.05146700237643},length_median=45.50072644536198,length_range={19.219340825596348,75.8087984962152},rate=0.002857489051137989,rate_95%_HPD={0.0016372876003594458,0.004648423363165846},rate_median=0.0026551750531274496,rate_range={0.0015315791842747716,0.0065770826474146026}]:45.68114469491683,89[&height=2.308908761904172E-14,height_95%_HPD={0.0,2.8421709430404007E-14},height_median=2.8421709430404007E-14,height_range={0.0,5.6843418860808015E-14},length=45.681144694916846,length_95%_HPD={24.00668712296229,67.05146700237643},length_median=45.50072644536198,length_range={19.219340825596348,75.8087984962152},rate=0.002997223189050722,rate_95%_HPD={0.0017620637888394817,0.004942010057793069},rate_median=0.0027847577038661187,rate_range={0.0016134239128732324,0.006659928531670037}]:45.68114469491683)[&height=45.68114469491685,height_95%_HPD={24.00668712296232,67.05146700237646},height_median=45.50072644536199,height_range={19.219340825596376,75.80879849621523},length=17.154449743528936,length_95%_HPD={3.3431695716001997,32.746248697116414},length_median=16.324907347911847,length_range={2.0164666216404328,45.22539377842429},posterior=1.0,rate=0.0020779225975838247,rate_95%_HPD={6.103195212477041E-4,0.004666511704405376},rate_median=0.0016504404158794975,rate_range={4.986678652988834E-4,0.013852023164850239}]:17.1544497435288)[&height=62.83559443844565,height_95%_HPD={47.80060985677976,77.82494989799447},height_median=62.25651375604745,height_range={47.80060985677976,89.88548786079099},length=22.32933904788819,length_95%_HPD={8.279909823212336,37.12684352841927},length_median=22.175808662970873,length_range={5.614914914889397,44.82817246930817},posterior=1.0,rate=0.0035821034032025903,rate_95%_HPD={0.0016232803529510874,0.007011703473016187},rate_median=0.003064240139502992,rate_range={0.001503027899967807,0.011474694216026617}]:22.32933904788807,(43[&height=2.317431865491858E-14,height_95%_HPD={0.0,2.8421709430404007E-14},height_median=2.8421709430404007E-14,height_range={0.0,5.6843418860808015E-14},length=64.30073712183585,length_95%_HPD={56.00456879411203,78.5548938469085},length_median=62.693622515759394,length_range={56.000315786235916,86.81258701627387},rate=0.001340643936617661,rate_95%_HPD={0.0010401037362804716,0.0015510364282194794},rate_median=0.0013570389637598932,rate_range={9.383105477454839E-4,0.0016805727133132888}]:64.30073712183587,((68[&height=2.3077723480924805E-14,height_95%_HPD={0.0,2.8421709430404007E-14},height_median=2.8421709430404007E-14,height_range={0.0,5.6843418860808015E-14},length=16.569852350776582,length_95%_HPD={6.6538321190327,26.28445032120365},length_median=16.20489162642391,length_range={6.459523603316285,35.2596758939178},rate=0.001939043049033612,rate_95%_HPD={8.843607032755956E-4,0.003361952848077944},rate_median=0.0017860162615331919,rate_range={7.89249716010277E-4,0.004618339137289552}]:16.569852350776586,88[&height=2.3077723480924805E-14,height_95%_HPD={0.0,2.8421709430404007E-14},height_median=2.8421709430404007E-14,height_range={0.0,5.6843418860808015E-14},length=16.569852350776582,length_95%_HPD={6.6538321190327,26.28445032120365},length_median=16.20489162642391,length_range={6.459523603316285,35.2596758939178},rate=0.001708000400694045,rate_95%_HPD={7.6505377278337E-4,0.0029330504035052526},rate_median=0.0015785389764370037,rate_range={6.662069094389869E-4,0.004026060725850844}]:16.569852350776586)[&height=16.569852350776607,height_95%_HPD={6.653832119032728,26.28445032120368},height_median=16.204891626423922,height_range={6.459523603316285,35.25967589391783},length=6.9535017609174705,length_95%_HPD={0.7271279275224742,17.753303121503038},length_median=5.517491920780387,length_range={0.4804450149043049,33.125453937619255},posterior=1.0,rate=0.001745324758481606,rate_95%_HPD={2.556877469857251E-4,0.00436656790540362},rate_median=0.0013506963743146068,rate_range={2.4699704509044965E-4,0.015521507260767653}]:6.953501760917398,87[&height=2.3097610722629408E-14,height_95%_HPD={0.0,2.8421709430404007E-14},height_median=2.8421709430404007E-14,height_range={0.0,5.6843418860808015E-14},length=23.523354111693983,length_95%_HPD={12.839387081100682,37.40612948186741},length_median=22.6445293085499,length_range={10.652137799521029,50.58419798574121},rate=0.002298508907266006,rate_95%_HPD={0.0011831858394658783,0.0035286885138578095},rate_median=0.002226357105477649,rate_range={9.683217441215201E-4,0.00502809750529685}]:23.52335411169398)[&height=23.523354111694005,height_95%_HPD={12.83938708110071,37.40612948186741},height_median=22.644529308549913,height_range={10.652137799521057,50.58419798574124},length=40.77738301014197,length_95%_HPD={23.418829506315262,56.22383048047011},length_median=40.519060346397936,length_range={18.839434271883107,67.09835696599484},posterior=1.0,rate=0.0017626692227187787,rate_95%_HPD={0.0011204013753394557,0.0026643924848728366},rate_median=0.0016984367088897726,rate_range={0.0010088652218155129,0.003781173303256374}]:40.77738301014189)[&height=64.3007371218359,height_95%_HPD={56.00456879411206,78.5548938469085},height_median=62.69362251575942,height_range={56.000315786235944,86.8125870162739},length=20.864196364497907,length_95%_HPD={6.519197444248292,33.59542426026904},length_median=21.060480012487005,length_range={4.526350631417799,44.18326068755229},posterior=1.0,rate=0.0038205293502516467,rate_95%_HPD={0.001697077357709047,0.007857408583779065},rate_median=0.003187652084465917,rate_range={0.0014678752763734415,0.014435410860073996}]:20.86419636449783)[&height=85.16493348633372,height_95%_HPD={75.48029946022235,95.2390549874786},height_median=85.0272037886996,height_range={69.89854714199258,104.89645400683466},length=1.8257924215975059,length_95%_HPD={0.12630822455103896,4.497537790053315},length_median=1.477221477077265,length_range={0.004985693176678296,10.02771025400034},posterior=1.0,rate=0.0020121603881214103,rate_95%_HPD={2.860900626626417E-4,0.004740729113707548},rate_median=0.0016144491965738614,rate_range={2.793943061947014E-4,0.015620019876822485}]:1.8257924215979813)[&height=86.9907259079317,height_95%_HPD={77.43249285804372,96.93388764733206},height_median=86.85378033533887,height_range={72.77087853224683,106.50960961432438},length=1.7724598516855752,length_95%_HPD={0.19089756480198616,4.280021219819929},length_median=1.422863871433016,length_range={0.1457257524887723,10.504173262756467},posterior=1.0,rate=0.001982443469378413,rate_95%_HPD={2.803648935086023E-4,0.004584778861617997},rate_median=0.0016383719892429423,rate_range={2.7731090658118275E-4,0.013964003585843519}]:1.7724598516853405,(50[&height=2.315727244774321E-14,height_95%_HPD={0.0,2.8421709430404007E-14},height_median=2.8421709430404007E-14,height_range={0.0,5.6843418860808015E-14},length=64.32141000002856,length_95%_HPD={47.809253204256834,83.55185054298201},length_median=62.83034269395644,length_range={47.809253204256834,95.67731930329553},rate=9.570050426450891E-4,rate_95%_HPD={6.960723179618802E-4,0.0012530398153131703},rate_median=9.46355997863534E-4,rate_range={5.770363442659388E-4,0.0013635057066692143}]:64.32141000002856,102[&height=2.315727244774321E-14,height_95%_HPD={0.0,2.8421709430404007E-14},height_median=2.8421709430404007E-14,height_range={0.0,5.6843418860808015E-14},length=64.32141000002856,length_95%_HPD={47.809253204256834,83.55185054298201},length_median=62.83034269395644,length_range={47.809253204256834,95.67731930329553},rate=5.010413169202806E-4,rate_95%_HPD={3.456270032249575E-4,6.602731493863841E-4},rate_median=4.958636729411937E-4,rate_range={2.9494485806382E-4,7.574551225331434E-4}]:64.32141000002856)[&height=64.32141000002859,height_95%_HPD={47.80925320425686,83.55185054298204},height_median=62.83034269395644,height_range={47.80925320425686,95.67731930329556},length=24.44177575958854,length_95%_HPD={4.806400044558586,42.9829388397978},length_median=24.739792185320802,length_range={2.6369471710800525,52.41479016839101},posterior=1.0,rate=9.441084182742728E-4,rate_95%_HPD={3.3039347680795565E-4,0.002473503564508495},rate_median=6.661443937921097E-4,rate_range={2.860900626626417E-4,0.006380921289743431}]:24.441775759588452)[&height=88.76318575961704,height_95%_HPD={78.91153135440275,98.48072734163685},height_median=88.50621329829815,height_range={74.06233927246467,107.0012414652225},length=5.737815569563025,length_95%_HPD={0.893758965311946,12.179228696203282},length_median=4.930129948351805,length_range={0.6327895059958735,23.276122060096498},posterior=1.0,rate=0.0020834678887519534,rate_95%_HPD={4.471200603739856E-4,0.004637481620049017},rate_median=0.001702992327263805,rate_range={3.5338588135512983E-4,0.01277276574580176}]:5.7378155695632245,61[&height=2.3162954516801665E-14,height_95%_HPD={0.0,2.8421709430404007E-14},height_median=2.8421709430404007E-14,height_range={0.0,5.6843418860808015E-14},length=94.50100132918021,length_95%_HPD={83.75119198674747,104.32867506204283},length_median=94.45052790879586,length_range={77.96789354421499,113.49169827745455},rate=9.700126675498405E-4,rate_95%_HPD={8.422496128220531E-4,0.0010919142966626036},rate_median=9.64884765352087E-4,rate_range={7.672903632624166E-4,0.0011946671891589742}]:94.50100132918024)[&height=94.50100132918027,height_95%_HPD={83.7511919867475,104.32867506204286},height_median=94.45052790879586,height_range={77.96789354421502,113.49169827745457},length=6.269432050086309,length_95%_HPD={1.1673464767265358,12.731464492540354},length_median=5.667597503228471,length_range={0.48262493973324183,20.580979226348717},posterior=1.0,rate=0.002152264463390816,rate_95%_HPD={5.439895139441842E-4,0.004829716360744545},rate_median=0.0017533800530871492,rate_range={4.436834275854202E-4,0.01766385614613931}]:6.2694320500863085)[&height=100.77043337926658,height_95%_HPD={90.44210303626078,110.13564183300885},height_median=101.00515059892658,height_range={82.76861688610538,116.81879391932956},length=2.906035272618137,length_95%_HPD={0.5705232897537087,6.280975479188058},length_median=2.5489658721387016,length_range={0.27563438096831305,11.572084570383481},posterior=1.0,rate=0.0027549885344456066,rate_95%_HPD={6.479462091231405E-4,0.006048953450877785},rate_median=0.0023322911339618045,rate_range={4.980821084322504E-4,0.015456298570799675}]:2.9060352726179843,(56[&height=2.3165795551330895E-14,height_95%_HPD={0.0,2.8421709430404007E-14},height_median=2.8421709430404007E-14,height_range={0.0,5.6843418860808015E-14},length=74.75666468015258,length_95%_HPD={50.39799268291375,100.30595545376306},length_median=75.3279553672439,length_range={35.018853664629475,104.8652114595957},rate=0.0011151795971412767,rate_95%_HPD={7.729758637265786E-4,0.001587000137235335},rate_median=0.001065932215083257,rate_range={7.312267491624858E-4,0.002240750765610526}]:74.75666468015258,(57[&height=2.317715968944781E-14,height_95%_HPD={0.0,2.8421709430404007E-14},height_median=2.8421709430404007E-14,height_range={0.0,5.6843418860808015E-14},length=32.40547901609883,length_95%_HPD={10.940536815368745,52.96576657438473},length_median=32.14172484890203,length_range={9.498011417582589,70.99839119036352},rate=0.0033708036030238604,rate_95%_HPD={0.0013830335914756127,0.006627073908169997},rate_median=0.0029476963333315703,rate_range={0.001288127211856863,0.0101929696074619}]:32.40547901609883,75[&height=2.317715968944781E-14,height_95%_HPD={0.0,2.8421709430404007E-14},height_median=2.8421709430404007E-14,height_range={0.0,5.6843418860808015E-14},length=32.40547901609883,length_95%_HPD={10.940536815368745,52.96576657438473},length_median=32.14172484890203,length_range={9.498011417582589,70.99839119036352},rate=0.0030016629848116324,rate_95%_HPD={0.0012780126679472893,0.005917780806169163},rate_median=0.0026136305558234632,rate_range={0.001185924159155122,0.00926097297552683}]:32.40547901609883)[&height=32.40547901609885,height_95%_HPD={10.940536815368773,52.96576657438473},height_median=32.14172484890206,height_range={9.498011417582589,70.99839119036355},length=42.35118566405354,length_95%_HPD={18.15162270659272,68.24774206705743},length_median=41.19418982661945,length_range={13.066742720344322,83.5090721254234},posterior=1.0,rate=0.002202609816333648,rate_95%_HPD={0.0010416293145888785,0.003808579993076481},rate_median=0.002025691522750874,rate_range={9.655264831950018E-4,0.00652538132281578}]:42.35118566405376)[&height=74.75666468015261,height_95%_HPD={50.39799268291378,100.30595545376306},height_median=75.32795536724392,height_range={35.0188536646295,104.86521145959573},length=28.919803971731824,length_95%_HPD={7.879266730842772,51.391311380417804},length_median=27.78708816946928,length_range={6.406015741171203,69.36608301117565},posterior=1.0,rate=0.0014116653820521884,rate_95%_HPD={4.914750471030497E-4,0.0028236968973364834},rate_median=0.0012270162571470628,rate_range={4.179907686420508E-4,0.005372969268660436}]:28.91980397173195)[&height=103.67646865188456,height_95%_HPD={93.30460208220109,113.04558024613505},height_median=104.00278846735566,height_range={84.56954949337556,118.82999068045085},length=8.566441454421973,length_95%_HPD={3.0964637329260114,14.457359495960617},length_median=8.18566699176344,length_range={2.505140790511547,23.53159287066798},posterior=1.0,rate=0.005326414078833693,rate_95%_HPD={0.0019357597066442331,0.009785445326352983},rate_median=0.004815594259086373,rate_range={0.0015637232929413155,0.014492460052161895}]:8.566441454422105,((((((11[&height=2.3080564515454035E-14,height_95%_HPD={0.0,5.6843418860808015E-14},height_median=2.8421709430404007E-14,height_range={0.0,5.6843418860808015E-14},length=30.681813051947714,length_95%_HPD={16.42919146111305,42.61457187188111},length_median=30.723598741915623,length_range={14.320173324070282,52.90815085819651},rate=0.006262541786138727,rate_95%_HPD={0.0037911284408957287,0.00973370855224266},rate_median=0.005927839783029675,rate_range={0.00350683513607872,0.013175824592043212}]:30.68181305194771,49[&height=2.3080564515454035E-14,height_95%_HPD={0.0,5.6843418860808015E-14},height_median=2.8421709430404007E-14,height_range={0.0,5.6843418860808015E-14},length=30.681813051947714,length_95%_HPD={16.42919146111305,42.61457187188111},length_median=30.723598741915623,length_range={14.320173324070282,52.90815085819651},rate=0.002391883691654659,rate_95%_HPD={0.0014743138500870234,0.003744279443336681},rate_median=0.0022626718292446976,rate_range={0.0012718813202957843,0.004874789488026466}]:30.68181305194771)[&height=30.68181305194773,height_95%_HPD={16.429191461113078,42.61457187188111},height_median=30.72359874191565,height_range={14.32017332407031,52.908150858196535},length=5.929500975738255,length_95%_HPD={1.4114099155113848,11.867395782779539},length_median=5.355696261757608,length_range={0.6198211138760712,19.327097297090063},posterior=1.0,rate=0.002367670851161505,rate_95%_HPD={6.279262435465766E-4,0.005154623115634158},rate_median=0.002010817983635646,rate_range={4.7799346514351167E-4,0.013319901509015127}]:5.929500975738339,((52[&height=2.3020902790340232E-14,height_95%_HPD={0.0,5.6843418860808015E-14},height_median=2.8421709430404007E-14,height_range={0.0,8.526512829121202E-14},length=28.370346306068235,length_95%_HPD={10.550529407978217,40.045295983415784},length_median=29.023821521932646,length_range={7.231296409883555,48.03854697298274},rate=0.0029187164613536233,rate_95%_HPD={0.0015915210267681394,0.0057669438167074795},rate_median=0.002605313345091527,rate_range={0.001563435923108454,0.010407721008579724}]:28.37034630606823,71[&height=2.3020902790340232E-14,height_95%_HPD={0.0,5.6843418860808015E-14},height_median=2.8421709430404007E-14,height_range={0.0,8.526512829121202E-14},length=28.370346306068235,length_95%_HPD={10.550529407978217,40.045295983415784},length_median=29.023821521932646,length_range={7.231296409883555,48.03854697298274},rate=0.0031302930967588567,rate_95%_HPD={0.0016601280972209573,0.00613453907079162},rate_median=0.0028042789130901507,rate_range={0.0016601280972209573,0.011231803405135663}]:28.37034630606823)[&height=28.370346306068253,height_95%_HPD={10.550529407978246,40.04529598341581},height_median=29.02382152193266,height_range={7.2312964098835835,48.03854697298277},length=4.4165683550583426,length_95%_HPD={0.4050289833459857,10.71923985439392},length_median=3.5913640322609695,length_range={0.3439782112806711,22.943516426178107},posterior=1.0,rate=0.002086509492557168,rate_95%_HPD={2.8308400902288424E-4,0.004922421460008907},rate_median=0.0016934006916772628,rate_range={2.7674994450593545E-4,0.01826065084477976}]:4.416568355058335,64[&height=2.3214093138327783E-14,height_95%_HPD={0.0,5.6843418860808015E-14},height_median=2.8421709430404007E-14,height_range={0.0,5.6843418860808015E-14},length=32.78691466112657,length_95%_HPD={15.644525651869841,47.341803379342764},length_median=33.1595278695699,length_range={9.996705956010473,53.76442424385792},rate=0.002592487726867081,rate_95%_HPD={0.0015006328904213493,0.004335874763488277},rate_median=0.0023851495011795185,rate_range={0.0014137387458347012,0.007853908705664483}]:32.786914661126566)[&height=32.78691466112659,height_95%_HPD={15.644525651869841,47.34180337934279},height_median=33.159527869569914,height_range={9.996705956010501,53.764424243857945},length=3.8243993665594176,length_95%_HPD={0.4707486016999667,9.288058250095446},length_median=3.059436385291292,length_range={0.33794579985310236,19.165081258627595},posterior=1.0,rate=0.001974050614392392,rate_95%_HPD={3.107008696858493E-4,0.004839208491686662},rate_median=0.0015902386429310577,rate_range={2.8395645113916664E-4,0.010577271681618186}]:3.824399366559483)[&height=36.61131402768607,height_95%_HPD={21.923156558993625,49.227651761651316},height_median=36.763252458732076,height_range={18.187475664762175,57.59753149552256},length=9.03943327890945,length_95%_HPD={2.6383355921571336,17.33678989217246},length_median=8.58247335708058,length_range={0.9318089502533695,25.467942670373972},posterior=1.0,rate=0.002391695755686999,rate_95%_HPD={7.53249448384264E-4,0.004986381982137647},rate_median=0.0019831381772858884,rate_range={6.476144338105E-4,0.016782433850617683}]:9.039433278909364,((84[&height=2.2884533132937253E-14,height_95%_HPD={0.0,2.8421709430404007E-14},height_median=2.8421709430404007E-14,height_range={0.0,8.526512829121202E-14},length=23.243255806451195,length_95%_HPD={11.072439839880246,38.13896325757099},length_median=22.402405010720308,length_range={9.269470210643505,47.16094348106081},rate=0.0024838439708255483,rate_95%_HPD={0.0012035336787179717,0.004189650319822605},rate_median=0.002344042358852872,rate_range={0.0010495905975477261,0.005761058904948177}]:23.2432558064512,96[&height=2.2884533132937253E-14,height_95%_HPD={0.0,2.8421709430404007E-14},height_median=2.8421709430404007E-14,height_range={0.0,8.526512829121202E-14},length=23.243255806451195,length_95%_HPD={11.072439839880246,38.13896325757099},length_median=22.402405010720308,length_range={9.269470210643505,47.16094348106081},rate=0.003037107653322158,rate_95%_HPD={0.0014526107673000384,0.005082124302531348},rate_median=0.002858203650001992,rate_range={0.0013457211135609502,0.007092170262171292}]:23.2432558064512)[&height=23.24325580645122,height_95%_HPD={11.072439839880275,38.13896325757102},height_median=22.402405010720322,height_range={9.269470210643533,47.16094348106081},length=14.669215994209061,length_95%_HPD={3.0499218532553414,27.709262382408},length_median=13.946416273797709,length_range={2.4024453268449832,35.81311172889757},posterior=1.0,rate=0.0022059177016047534,rate_95%_HPD={7.122271665468924E-4,0.004898529841233619},rate_median=0.0017940788198272865,rate_range={5.978061062168712E-4,0.009702525957178}]:14.669215994209164,105[&height=2.3035107962986374E-14,height_95%_HPD={0.0,2.8421709430404007E-14},height_median=2.8421709430404007E-14,height_range={0.0,8.526512829121202E-14},length=37.91247180066036,length_95%_HPD={21.97578022115617,53.17518190421835},length_median=37.78768891431562,length_range={16.39369687172703,60.05232584393431},rate=0.0024177269951897442,rate_95%_HPD={0.0015598193983211881,0.003714097154696778},rate_median=0.00231804663706514,rate_range={0.0013972843948176748,0.005515821541318519}]:37.91247180066036)[&height=37.912471800660384,height_95%_HPD={21.9757802211562,53.17518190421838},height_median=37.78768891431564,height_range={16.39369687172703,60.05232584393434},length=7.738275505935174,length_95%_HPD={0.5597874657624686,18.905719236520937},length_median=6.15565678763646,length_range={0.5597874657624686,35.756405027167474},posterior=1.0,rate=0.0018114306833918751,rate_95%_HPD={2.707553703436406E-4,0.004368383230275324},rate_median=0.0014546053326201323,rate_range={2.707553703436406E-4,0.014868517699851074}]:7.73827550593505)[&height=45.650747306595434,height_95%_HPD={30.728893267559158,59.58939644200575},height_median=46.08691100560014,height_range={24.499024335698493,66.40746814642445},length=20.558558924518916,length_95%_HPD={5.4378843715913945,36.49295214455964},length_median=19.785441529393935,length_range={3.5780133144856165,44.314374158417564},posterior=1.0,rate=0.0024717107022862667,rate_95%_HPD={8.90913934942178E-4,0.005607595149632099},rate_median=0.0020072599198335613,rate_range={8.37081480447531E-4,0.010828129722578019}]:20.558558924519005,((((22[&height=2.29584000306972E-14,height_95%_HPD={0.0,5.6843418860808015E-14},height_median=2.8421709430404007E-14,height_range={0.0,8.526512829121202E-14},length=4.387602287259854,length_95%_HPD={1.373981821938628,8.136376411507456},length_median=4.019351127943992,length_range={1.0242164857011176,13.968815972993113},rate=0.0030635219576819893,rate_95%_HPD={9.850435878591421E-4,0.005666481018286319},rate_median=0.002829874220195632,rate_range={6.34880533931828E-4,0.01291815575995922}]:4.387602287259852,103[&height=2.29584000306972E-14,height_95%_HPD={0.0,5.6843418860808015E-14},height_median=2.8421709430404007E-14,height_range={0.0,8.526512829121202E-14},length=4.387602287259854,length_95%_HPD={1.373981821938628,8.136376411507456},length_median=4.019351127943992,length_range={1.0242164857011176,13.968815972993113},rate=0.001582325634388746,rate_95%_HPD={4.683562221085485E-4,0.0029325543691845458},rate_median=0.0014566888641466451,rate_range={3.555146201505856E-4,0.006589942631094703}]:4.387602287259852)[&height=4.387602287259875,height_95%_HPD={1.3739818219386564,8.136376411507456},height_median=4.019351127943992,height_range={1.024216485701146,13.968815972993141},length=25.040852978813902,length_95%_HPD={12.584257934328235,38.506104859920725},length_median=24.5153564040534,length_range={9.877154068259387,49.18873616922346},posterior=1.0,rate=0.005490707850894407,rate_95%_HPD={0.0029754381797413776,0.00893378696099241},rate_median=0.00516583005046967,rate_range={0.0026926581868639762,0.012631588588233288}]:25.04085297881387,97[&height=2.3222616241915468E-14,height_95%_HPD={0.0,4.263256414560601E-14},height_median=2.8421709430404007E-14,height_range={0.0,8.526512829121202E-14},length=29.428455266073723,length_95%_HPD={16.241627483320954,43.0286877378428},length_median=28.77704698073984,length_range={14.063087767669685,52.07622115905815},rate=0.0026513009496721284,rate_95%_HPD={0.0015208903267108509,0.004005258435028353},rate_median=0.002540067269761915,rate_range={0.0013888791584242785,0.005203461926615395}]:29.42845526607372)[&height=29.428455266073744,height_95%_HPD={16.241627483320954,43.02868773784283},height_median=28.77704698073987,height_range={14.063087767669714,52.07622115905815},length=19.643718390545732,length_95%_HPD={8.056650167429751,31.061173559523837},length_median=19.574861341176906,length_range={4.289337860152443,38.94496427991497},posterior=1.0,rate=0.003933202637418261,rate_95%_HPD={0.001846892350550543,0.006837546005885407},rate_median=0.0035117627207505294,rate_range={0.0017286366286145577,0.015350349371749249}]:19.643718390545853,((51[&height=2.3273754863441586E-14,height_95%_HPD={0.0,5.6843418860808015E-14},height_median=2.8421709430404007E-14,height_range={0.0,8.526512829121202E-14},length=16.92543473459167,length_95%_HPD={7.183524392543461,29.81217726199756},length_median=15.936357845974996,length_range={4.6225092112185,40.67883530509292},rate=0.0026663878000927834,rate_95%_HPD={0.0010574739974721355,0.004606140022854001},rate_median=0.0025041312646403397,rate_range={9.524520816958181E-4,0.00846417411455538}]:16.92543473459167,54[&height=2.3273754863441586E-14,height_95%_HPD={0.0,5.6843418860808015E-14},height_median=2.8421709430404007E-14,height_range={0.0,8.526512829121202E-14},length=16.92543473459167,length_95%_HPD={7.183524392543461,29.81217726199756},length_median=15.936357845974996,length_range={4.6225092112185,40.67883530509292},rate=0.0018520503316436226,rate_95%_HPD={7.301339138117777E-4,0.0031949242012840103},rate_median=0.001742027472963746,rate_range={6.580984479676828E-4,0.00588360050049851}]:16.92543473459167)[&height=16.925434734591693,height_95%_HPD={7.1835243925434895,29.81217726199759},height_median=15.936357845975017,height_range={4.622509211218528,40.678835305092946},length=14.730902291117625,length_95%_HPD={2.7281476396433106,27.052618837425896},length_median=14.351532091830236,length_range={2.7281476396433106,40.14595784885452},posterior=1.0,rate=0.0035623213710290333,rate_95%_HPD={0.0010747786261456117,0.00899186304900361},rate_median=0.0026608550876084077,rate_range={0.001001177327609127,0.014379305005132943}]:14.73090229111769,74[&height=2.3324893484967704E-14,height_95%_HPD={0.0,4.263256414560601E-14},height_median=2.8421709430404007E-14,height_range={0.0,5.6843418860808015E-14},length=31.656337025709362,length_95%_HPD={16.370774266476488,47.16256114852263},length_median=31.08489166204535,length_range={14.005804604706526,57.09295174313269},rate=0.0019297039847761086,rate_95%_HPD={0.0010997777829558507,0.0031102817691701784},rate_median=0.0018110740875318591,rate_range={9.293918772135792E-4,0.00404818099431445}]:31.65633702570936)[&height=31.656337025709384,height_95%_HPD={16.370774266476516,47.162561148522656},height_median=31.084891662045365,height_range={14.005804604706555,57.09295174313269},length=17.41583663091019,length_95%_HPD={4.957580417898583,30.615047156183905},length_median=16.59370982670243,length_range={2.9904263721444266,41.174257616531094},posterior=1.0,rate=0.002086271236633157,rate_95%_HPD={8.082477693285893E-4,0.004202819737388092},rate_median=0.0018165648769351132,rate_range={7.24172817240371E-4,0.008934882489535759}]:17.415836630910214)[&height=49.0721736566196,height_95%_HPD={30.097720572187697,63.7355630072212},height_median=49.917417631460125,height_range={24.524764965573723,70.91069136442054},length=7.785033330713342,length_95%_HPD={1.2427699213451575,16.955273577348436},length_median=6.769025363784351,length_range={0.8647124091939133,30.65340657094086},posterior=1.0,rate=0.0019168107183431741,rate_95%_HPD={3.619512943997308E-4,0.004337991817256368},rate_median=0.0015786243236695517,rate_range={3.619512943997308E-4,0.01048871429845526}]:7.7850333307132615,48[&height=2.3180000723977038E-14,height_95%_HPD={0.0,2.8421709430404007E-14},height_median=2.8421709430404007E-14,height_range={0.0,5.6843418860808015E-14},length=56.857206987332845,length_95%_HPD={35.00338911258902,72.59501812352151},length_median=57.50936310874226,length_range={29.125555739056253,89.51307614425895},rate=0.0019722818332575344,rate_95%_HPD={0.0013751100709464242,0.002883124211469381},rate_median=0.0018948377691324005,rate_range={0.0011784130968488546,0.003729524700858504}]:56.85720698733284)[&height=56.85720698733286,height_95%_HPD={35.00338911258905,72.59501812352154},height_median=57.509363108742285,height_range={29.12555573905628,89.51307614425895},length=9.352099243781534,length_95%_HPD={1.7599654082836764,21.074355545036312},length_median=7.822893926552837,length_range={1.2748838492541523,35.59616061584926},posterior=1.0,rate=0.0018297403684467712,rate_95%_HPD={3.725655994505597E-4,0.004299382120853515},rate_median=0.001508064621779098,rate_range={2.7692612393591295E-4,0.009044899914362655}]:9.35209924378158)[&height=66.20930623111444,height_95%_HPD={50.758664492185915,81.34538176608588},height_median=65.8137371333447,height_range={47.08513200974859,96.58521886384737},length=27.719746670615347,length_95%_HPD={9.735633779183374,40.664648248042255},length_median=28.056958545784788,length_range={9.566018646142055,57.21924683420673},posterior=1.0,rate=0.004010456131803827,rate_95%_HPD={0.002043425151987042,0.007507421480189117},rate_median=0.0036129764208922737,rate_range={0.0017707776411894235,0.011182128164439155}]:27.329956792785595,(((38[&height=2.3083405549983262E-14,height_95%_HPD={0.0,2.8421709430404007E-14},height_median=2.8421709430404007E-14,height_range={0.0,5.6843418860808015E-14},length=55.1883687958378,length_95%_HPD={33.28189061232965,74.73360085684178},length_median=56.35851876819582,length_range={21.601521011968444,86.84732250920601},rate=0.004079055926692709,rate_95%_HPD={0.00263750095736998,0.005982912683953028},rate_median=0.003804473931781925,rate_range={0.0022963817961664506,0.010088607529774954}]:55.188368795837796,90[&height=2.3083405549983262E-14,height_95%_HPD={0.0,2.8421709430404007E-14},height_median=2.8421709430404007E-14,height_range={0.0,5.6843418860808015E-14},length=55.1883687958378,length_95%_HPD={33.28189061232965,74.73360085684178},length_median=56.35851876819582,length_range={21.601521011968444,86.84732250920601},rate=0.004429867799775341,rate_95%_HPD={0.003006735670798415,0.006642733548579945},rate_median=0.004132837102454894,rate_range={0.002640128715187791,0.01087505924422156}]:55.188368795837796)[&height=55.18836879583782,height_95%_HPD={33.281890612329676,74.7336008568418},height_median=56.35851876819585,height_range={21.601521011968472,86.84732250920604},length=13.631998877831627,length_95%_HPD={2.3944707472293345,28.09894085803016},length_median=12.17522755162787,length_range={1.0810893693334123,43.56912301494695},posterior=1.0,rate=0.0022624964647758893,rate_95%_HPD={5.367987057633167E-4,0.005015327322911667},rate_median=0.0018619374323460879,rate_range={3.288369347733696E-4,0.014935943719933042}]:13.631998877831435,(91[&height=2.313454417150938E-14,height_95%_HPD={0.0,2.8421709430404007E-14},height_median=2.8421709430404007E-14,height_range={0.0,5.6843418860808015E-14},length=13.130803131293163,length_95%_HPD={3.451644012129293,24.24600572527308},length_median=12.187287712481059,length_range={3.3192576043145436,40.911585064167355},rate=0.0022038286838201825,rate_95%_HPD={6.169610883056718E-4,0.004152801989056053},rate_median=0.001975424482994795,rate_range={4.7297461068653365E-4,0.007739095624545316}]:13.130803131293165,92[&height=2.313454417150938E-14,height_95%_HPD={0.0,2.8421709430404007E-14},height_median=2.8421709430404007E-14,height_range={0.0,5.6843418860808015E-14},length=13.130803131293163,length_95%_HPD={3.451644012129293,24.24600572527308},length_median=12.187287712481059,length_range={3.3192576043145436,40.911585064167355},rate=0.0023000827198507785,rate_95%_HPD={6.340445235288808E-4,0.004322945624515299},rate_median=0.0020595993144211055,rate_range={5.620466719428094E-4,0.007587286908159375}]:13.130803131293165)[&height=13.130803131293188,height_95%_HPD={3.4516440121293215,24.246005725273108},height_median=12.187287712481087,height_range={3.319257604314572,40.91158506416738},length=55.68956454237615,length_95%_HPD={37.12900957273877,73.02650585185651},length_median=56.37703995692931,length_range={22.983634821196148,83.73072308044306},posterior=1.0,rate=0.0019463165727982529,rate_95%_HPD={0.001379483649553509,0.002729751042951192},rate_median=0.001857957297169702,rate_range={0.001151240153071607,0.004442808107326609}]:55.68956454237606)[&height=68.82036767366925,height_95%_HPD={48.64548784646348,87.15352673087992},height_median=69.73204428740397,height_range={36.50611850715342,92.49114129906712},length=16.210568981587365,length_95%_HPD={3.788557056540128,32.9339490593285},length_median=14.762618515205943,length_range={2.090363547770565,47.20262740553781},posterior=1.0,rate=0.0019151423842498206,rate_95%_HPD={5.399864886558241E-4,0.003964231157068218},rate_median=0.0016392613910209163,rate_range={5.171332009913093E-4,0.011464219985926219}]:16.21056898158774,((44[&height=2.3148749344155523E-14,height_95%_HPD={0.0,2.8421709430404007E-14},height_median=2.8421709430404007E-14,height_range={0.0,5.6843418860808015E-14},length=14.451275384980864,length_95%_HPD={4.918488977123559,25.88876708595275},length_median=13.630368438091416,length_range={4.586445655843448,43.196121721495075},rate=0.0024026930380754955,rate_95%_HPD={9.119087435458151E-4,0.004397669635994365},rate_median=0.002194033448647355,rate_range={7.20151244976349E-4,0.00678466057503397}]:14.451275384980859,99[&height=2.3148749344155523E-14,height_95%_HPD={0.0,2.8421709430404007E-14},height_median=2.8421709430404007E-14,height_range={0.0,5.6843418860808015E-14},length=14.451275384980864,length_95%_HPD={4.918488977123559,25.88876708595275},length_median=13.630368438091416,length_range={4.586445655843448,43.196121721495075},rate=0.0016889277839879173,rate_95%_HPD={6.337391218173049E-4,0.00311286663467898},rate_median=0.001547018741125443,rate_range={4.7598137807487045E-4,0.004760707619102874}]:14.451275384980859)[&height=14.451275384980882,height_95%_HPD={4.918488977123587,25.88876708595278},height_median=13.630368438091445,height_range={4.586445655843477,43.1961217214951},length=26.237359542212598,length_95%_HPD={6.769401025312348,50.41093374776477},length_median=24.95008543691624,length_range={3.632754583635844,66.60607263895494},posterior=1.0,rate=0.0016665909067260548,rate_95%_HPD={5.342494772922368E-4,0.0035052105497318416},rate_median=0.0013557080913570562,rate_range={4.6402002500303794E-4,0.009381001560080572}]:26.237359542212488,45[&height=2.3162954516801665E-14,height_95%_HPD={0.0,2.8421709430404007E-14},height_median=2.8421709430404007E-14,height_range={0.0,5.6843418860808015E-14},length=40.688634927193355,length_95%_HPD={18.032088094736167,64.76362176163407},length_median=39.42040352885655,length_range={15.286999341511375,80.51441819131095},rate=0.0030905978323714344,rate_95%_HPD={0.0015313907871249916,0.005208315705243502},rate_median=0.002880616234980664,rate_range={0.0014210665190234248,0.007342235893524816}]:40.68863492719335)[&height=40.68863492719337,height_95%_HPD={18.032088094736196,64.7636217616341},height_median=39.42040352885655,height_range={15.286999341511404,80.51441819131098},length=44.342301728063305,length_95%_HPD={13.613317232752564,68.61549715874156},length_median=45.236037348047766,length_range={8.43053460466193,84.09100762099051},posterior=1.0,rate=0.0015872026683000644,rate_95%_HPD={7.284685565155217E-4,0.0034783312528017343},rate_median=0.0013197293943423,rate_range={6.803623247529297E-4,0.007005542665734231}]:44.342301728063624)[&height=85.030936655257,height_95%_HPD={68.62753955162367,100.12448108845123},height_median=85.10873630474504,height_range={59.12437870590621,106.82167029139151},length=9.41323768623655,length_95%_HPD={1.8196177074284634,19.709206533072532},length_median=8.395120348956183,length_range={0.6862189881154421,32.63783749813753},posterior=1.0,rate=0.0018710156323654681,rate_95%_HPD={4.3801535187039105E-4,0.003969553869988334},rate_median=0.0015617404396728125,rate_range={3.064926448931643E-4,0.02007193323667884}]:8.50832636864304)[&height=93.53926302390003,height_95%_HPD={78.14170080135142,105.99497994115939},height_median=93.89774531440023,height_range={70.4063650749625,112.31621058665235},length=4.975952887659495,length_95%_HPD={0.42891320147150225,11.52492316319092},length_median=4.1099731023057515,length_range={0.2528963222403888,26.697273011434845},posterior=0.8580567772890844,rate=0.0020336252414118357,rate_95%_HPD={3.5275491929475685E-4,0.004764338316703405},rate_median=0.0016656325792096327,rate_range={2.841583164878193E-4,0.0189056398998374}]:5.184517572421342,(((33[&height=2.324250348362007E-14,height_95%_HPD={0.0,2.8421709430404007E-14},height_median=2.8421709430404007E-14,height_range={0.0,5.6843418860808015E-14},length=36.76720739484669,length_95%_HPD={18.317846151421037,57.4684531379433},length_median=36.697234010515665,length_range={13.89606320286974,70.90531844293912},rate=0.005766201373878513,rate_95%_HPD={0.0030850000331889395,0.009641369007948996},rate_median=0.005240557625511615,rate_range={0.0027158534118093703,0.013634206013215595}]:36.767207394846714,82[&height=2.324250348362007E-14,height_95%_HPD={0.0,2.8421709430404007E-14},height_median=2.8421709430404007E-14,height_range={0.0,5.6843418860808015E-14},length=36.76720739484669,length_95%_HPD={18.317846151421037,57.4684531379433},length_median=36.697234010515665,length_range={13.89606320286974,70.90531844293912},rate=0.004783782785050399,rate_95%_HPD={0.0025289510803300293,0.00798127915489699},rate_median=0.004362555067974354,rate_range={0.00228380966950687,0.011972590467855954}]:36.767207394846714)[&height=36.767207394846736,height_95%_HPD={18.317846151421065,57.4684531379433},height_median=36.69723401051569,height_range={13.896063202869769,70.90531844293912},length=31.858567469177295,length_95%_HPD={15.768662868951424,50.39017787697493},length_median=30.84149842826288,length_range={12.33185795178548,64.3750687384943},posterior=1.0,rate=0.006106597615030137,rate_95%_HPD={0.0030957034980047963,0.010168972422914889},rate_median=0.005726061283733641,rate_range={0.002572890173565722,0.014329990201648058}]:31.85856746917719,72[&height=2.313170313698015E-14,height_95%_HPD={0.0,2.8421709430404007E-14},height_median=2.8421709430404007E-14,height_range={0.0,5.6843418860808015E-14},length=68.62577486402391,length_95%_HPD={47.504222565827746,86.73757014727478},length_median=68.62599293486326,length_range={41.12930661738375,100.21171277845207},rate=0.0010683363897614382,rate_95%_HPD={7.722802157265783E-4,0.0014433430547349437},rate_median=0.0010435959851252318,rate_range={7.053413874107871E-4,0.0018452765399438018}]:68.6257748640239)[&height=68.62577486402392,height_95%_HPD={47.504222565827774,86.73757014727481},height_median=68.62599293486329,height_range={41.129306617383776,100.2117127784521},length=9.237369226653806,length_95%_HPD={1.503636968819265,20.138368640239648},length_median=7.974728823990326,length_range={0.994851625844035,40.98088913388767},posterior=1.0,rate=0.0022030214414480464,rate_95%_HPD={3.84866325324099E-4,0.004978617432614145},rate_median=0.0018246673566041783,rate_range={3.416647057401859E-4,0.014241590250833863}]:9.237369226654053,66[&height=2.3162954516801665E-14,height_95%_HPD={0.0,2.8421709430404007E-14},height_median=2.8421709430404007E-14,height_range={0.0,5.6843418860808015E-14},length=77.86314409067796,length_95%_HPD={57.69496316067534,95.91849681840222},length_median=78.61345699146386,length_range={47.522220713050416,105.88325716666242},rate=0.0013070803320285747,rate_95%_HPD={9.84615995579566E-4,0.001672830078689641},rate_median=0.001275575245942448,rate_range={9.186416967615908E-4,0.0020511240072119486}]:77.86314409067795)[&height=77.86314409067798,height_95%_HPD={57.69496316067537,95.91849681840225},height_median=78.61345699146389,height_range={47.522220713050416,105.88325716666245},length=20.325602753678748,length_95%_HPD={5.54380785851707,34.69508465152326},length_median=19.500940911516473,length_range={4.02995145693734,45.613551841353015},posterior=1.0,rate=0.002883619732823437,rate_95%_HPD={0.001099766521132631,0.005997403584634216},rate_median=0.0024558856991919917,rate_range={0.0010069774887241575,0.010572686282923571}]:20.8606365056434)[&height=98.72378059632138,height_95%_HPD={86.33113681346258,111.15429572290708},height_median=98.89540499794873,height_range={73.20134745156447,118.2291436670074},length=13.519129509985092,length_95%_HPD={3.682393059181038,25.697128994381103},length_median=12.477580347361872,length_range={2.577914765673995,33.60525706418443},posterior=1.0,rate=0.0022109310945979655,rate_95%_HPD={6.975865785423047E-4,0.004672425943812697},rate_median=0.0019238663226102007,rate_range={6.130202612046708E-4,0.007735201923443042}]:13.51912950998529)[&height=112.24291010630667,height_95%_HPD={102.67024745610318,121.22102659447872},height_median=112.55418900676392,height_range={92.98948712985803,126.1848556727435},length=16.51637031829479,length_95%_HPD={9.928045013677092,24.35102587035462},length_median=15.835593359341807,length_range={7.944309518156103,32.10822389317114},posterior=1.0,rate=0.01159112663953606,rate_95%_HPD={0.006545791137308609,0.016223742858077813},rate_median=0.011486616396024638,rate_range={0.005593742706945959,0.022029100674438837}]:16.516370318294733,(9[&height=2.2972605203343344E-14,height_95%_HPD={0.0,2.8421709430404007E-14},height_median=2.8421709430404007E-14,height_range={0.0,5.6843418860808015E-14},length=23.47182127768995,length_95%_HPD={0.0013487577306534604,69.97458021088957},length_median=16.577309860887503,length_range={0.0013487577306534604,133.4528228399951},rate=0.003026832146863015,rate_95%_HPD={4.687914969397591E-4,0.00659218023970625},rate_median=0.00263423718677332,rate_range={2.841583164878193E-4,0.018032436506195567}]:23.47182127768996,21[&height=2.2972605203343344E-14,height_95%_HPD={0.0,2.8421709430404007E-14},height_median=2.8421709430404007E-14,height_range={0.0,5.6843418860808015E-14},length=23.47182127768995,length_95%_HPD={0.0013487577306534604,69.97458021088957},length_median=16.577309860887503,length_range={0.0013487577306534604,133.4528228399951},rate=0.00263395610404667,rate_95%_HPD={4.012909951233058E-4,0.0058316850486725535},rate_median=0.0022360493172620096,rate_range={2.4714510102739537E-4,0.020775507644950654}]:23.47182127768996)[&height=23.47182127768998,height_95%_HPD={0.001348757730681882,69.9745802108896},height_median=16.57730986088753,height_range={0.001348757730681882,133.45282283999512},length=105.28745914691136,length_95%_HPD={59.20346308882898,135.24076519177612},length_median=111.3759924319265,length_range={0.5085788238489783,136.39237072517227},posterior=1.0,rate=0.00429762971740946,rate_95%_HPD={0.0028964413099738114,0.0061103054276291134},rate_median=0.00424387598515567,rate_range={3.7866886326054386E-4,0.012682125159679376}]:105.28745914691142)[&height=128.7592804246014,height_95%_HPD={120.03737063301985,136.68767772309545},height_median=129.25740052476687,height_range={109.22709300787054,138.18099279006273},length=3.321241832257596,length_95%_HPD={0.002807135849622,8.754331594366747},length_median=2.6491518929397344,length_range={0.001577773288033768,20.404712342531468},posterior=1.0,rate=0.0027008607905063525,rate_95%_HPD={3.26072437609421E-4,0.006209186156266697},rate_median=0.002248521251627604,rate_range={2.898504477748648E-4,0.020408311413538733}]:3.3212418322574706,(((4[&height=2.3162954516801665E-14,height_95%_HPD={0.0,2.8421709430404007E-14},height_median=2.8421709430404007E-14,height_range={0.0,5.6843418860808015E-14},length=93.7746358123944,length_95%_HPD={68.9220661115143,117.70220870863898},length_median=94.8503237248209,length_range={44.42074654847448,129.52630138769368},rate=0.0034162891534816053,rate_95%_HPD={0.002581219273469739,0.004385247419768707},rate_median=0.0033219169300307315,rate_range={0.002377482447309507,0.005510263590559584}]:93.9343740987168,(25[&height=2.317715968944781E-14,height_95%_HPD={0.0,2.8421709430404007E-14},height_median=2.8421709430404007E-14,height_range={0.0,5.6843418860808015E-14},length=60.261392357338764,length_95%_HPD={31.942881592370355,93.07340384315306},length_median=60.278761481901654,length_range={21.795162821603213,109.23865974668179},rate=0.0053543464638079324,rate_95%_HPD={0.002768357984331316,0.008343127953643985},rate_median=0.0049725952256882144,rate_range={0.0026634382367537804,0.013307062532059289}]:60.261392357338785,26[&height=2.317715968944781E-14,height_95%_HPD={0.0,2.8421709430404007E-14},height_median=2.8421709430404007E-14,height_range={0.0,5.6843418860808015E-14},length=60.261392357338764,length_95%_HPD={31.942881592370355,93.07340384315306},length_median=60.278761481901654,length_range={21.795162821603213,109.23865974668179},rate=0.004727411973375184,rate_95%_HPD={0.002586883179946663,0.007544215627239406},rate_median=0.004384804609675393,rate_range={0.002316475758534971,0.01259267695154181}]:60.261392357338785)[&height=60.261392357338806,height_95%_HPD={31.942881592370384,93.07340384315309},height_median=60.27876148190168,height_range={21.795162821603242,109.23865974668182},length=34.52206381053082,length_95%_HPD={8.875319030043968,63.56354225378021},length_median=32.72214985791015,length_range={2.6232748796607694,93.79508598136383},posterior=1.0,rate=0.003381976566606903,rate_95%_HPD={0.0010272308909650492,0.006693264739314687},rate_median=0.0029423555573946944,rate_range={8.589429398162347E-4,0.015525365777658999}]:33.672981741378024)[&height=93.93437409871683,height_95%_HPD={71.7401213114047,116.35638000426846},height_median=94.44114870982206,height_range={58.0780979557793,124.33228633988325},length=12.119891037071483,length_95%_HPD={0.012966475802983268,31.395023750214534},length_median=9.962674743414368,length_range={0.012966475802983268,56.73418862420749},posterior=0.49160335865653737,rate=0.0020024805899189393,rate_95%_HPD={3.3097872144237947E-4,0.0046154652692395924},rate_median=0.0015992918639269205,rate_range={2.7586638789282175E-4,0.01849042537003703}]:9.068027184787653,5[&height=2.3162954516801665E-14,height_95%_HPD={0.0,2.8421709430404007E-14},height_median=2.8421709430404007E-14,height_range={0.0,5.6843418860808015E-14},length=97.47204897751132,length_95%_HPD={67.97587428793598,124.48501593605725},length_median=98.9125328070401,length_range={44.42074654847448,133.81699518992673},rate=0.005308033287097243,rate_95%_HPD={0.0035453741460305757,0.00733008622973192},rate_median=0.005119566619828679,rate_range={0.0031252328981008675,0.011805473810803413}]:103.00240128350445)[&height=103.00240128350448,height_95%_HPD={79.03208738478747,124.67856898259858},height_median=104.29084028507206,height_range={64.61162164637236,133.81699518992676},length=18.92219231812712,length_95%_HPD={2.017210624141967,41.10861507445969},length_median=16.95017968284575,length_range={0.05178692074287028,63.22664187006052},posterior=1.0,rate=0.0022306359156304117,rate_95%_HPD={4.721018850469274E-4,0.0048963254440565025},rate_median=0.0019039959824712184,rate_range={2.80374681463913E-4,0.014317482711678448}]:18.922192318126463,(42[&height=2.315727244774321E-14,height_95%_HPD={0.0,2.8421709430404007E-14},height_median=2.8421709430404007E-14,height_range={0.0,5.6843418860808015E-14},length=59.85460511095962,length_95%_HPD={31.039543191349992,89.95922047319031},length_median=58.72296224391538,length_range={22.664507305165614,100.83192766209923},rate=0.004716169425723456,rate_95%_HPD={0.0025582332562348693,0.007486758899582013},rate_median=0.004470657603618448,rate_range={0.002244000029539197,0.01152432673368505}]:59.854605110959625,104[&height=2.315727244774321E-14,height_95%_HPD={0.0,2.8421709430404007E-14},height_median=2.8421709430404007E-14,height_range={0.0,5.6843418860808015E-14},length=59.85460511095962,length_95%_HPD={31.039543191349992,89.95922047319031},length_median=58.72296224391538,length_range={22.664507305165614,100.83192766209923},rate=0.004317870821666165,rate_95%_HPD={0.0024085289047354142,0.006680102872314225},rate_median=0.00409460876455992,rate_range={0.0022627911411514366,0.009685507893079044}]:59.854605110959625)[&height=59.854605110959646,height_95%_HPD={31.03954319135002,89.95922047319034},height_median=58.72296224391541,height_range={22.664507305165642,100.83192766209926},length=62.06998849067146,length_95%_HPD={31.872649526989406,90.97357945185911},length_median=61.98783913655572,length_range={23.18078025724195,103.41163835981166},posterior=1.0,rate=0.005264057788724392,rate_95%_HPD={0.003041669027542098,0.008577210410615614},rate_median=0.004928922335935065,rate_range={0.0027163782944215464,0.01262182332708336}]:62.0699884906713)[&height=121.92459360163095,height_95%_HPD={107.29710793222887,134.23800825085607},height_median=122.9229198284661,height_range={90.51665537945843,136.50052994924337},length=10.155928655227592,length_95%_HPD={0.703495497195405,23.124536787577412},length_median=8.741473259564806,length_range={0.009287716503266097,42.82077506174136},posterior=1.0,rate=0.002110442481360665,rate_95%_HPD={3.4132783279715453E-4,0.004632424870584523},rate_median=0.0017740142369745086,rate_range={2.9399379138022176E-4,0.014465007312241665}]:10.155928655227925)[&height=132.08052225685887,height_95%_HPD={124.51484209725575,138.02364543663035},height_median=132.79768906299495,height_range={111.0896511676144,138.28195053412443},length=3.1291777207514784,length_95%_HPD={8.865035334792992E-5,8.694084367776952},length_median=2.321056255277,length_range={8.865035334792992E-5,23.897558218850477},posterior=1.0,rate=0.0028646849787840266,rate_95%_HPD={3.7970142944086587E-4,0.006978002943092313},rate_median=0.0022652697679147555,rate_range={2.499977457890866E-4,0.02046441597828572}]:3.129177720751329,67[&height=2.3162954516801665E-14,height_95%_HPD={0.0,2.8421709430404007E-14},height_median=2.8421709430404007E-14,height_range={0.0,5.6843418860808015E-14},length=135.20969997761017,length_95%_HPD={129.25491124607214,138.32565783170318},length_median=136.08867121313187,length_range={117.32708254282657,138.32565783170318},rate=0.004462758660237411,rate_95%_HPD={0.004089972234449424,0.004811381009779392},rate_median=0.004458277545967879,rate_range={0.003076221215728017,0.0054734299556239785}]:135.20969997761017)[&height=135.2096999776102,height_95%_HPD={129.25491124607217,138.3256578317032},height_median=136.0886712131319,height_range={117.32708254282659,138.3256578317032},length=0.0,posterior=1.0,rate=1.0]:0.0;
[truncated: 218,922 more chars]
