# Supplementary material for: Localized Phylogenetic Discordance Among Nuclear Loci Due to Incomplete Lineage Sorting and Introgression in the Family of Cotton and Cacao (Malvaceae)
Source: Front Plant Sci. 2022 Apr 13;13:850521. doi: 10.3389/fpls.2022.850521 (PMC9043901; doi:10.3389/fpls.2022.850521)

Hernández-Gutiérrez et al. Localized Phylogenetic Discordance among Nuclear Loci due to ILS and Introgression in The Family of Cotton and Cacao (Malvaceae)

Supplementary Figures

Figure S1. Maximum Likelihood concatenated phylogeny. Numbers associated to nodes represent Bootstrap support.

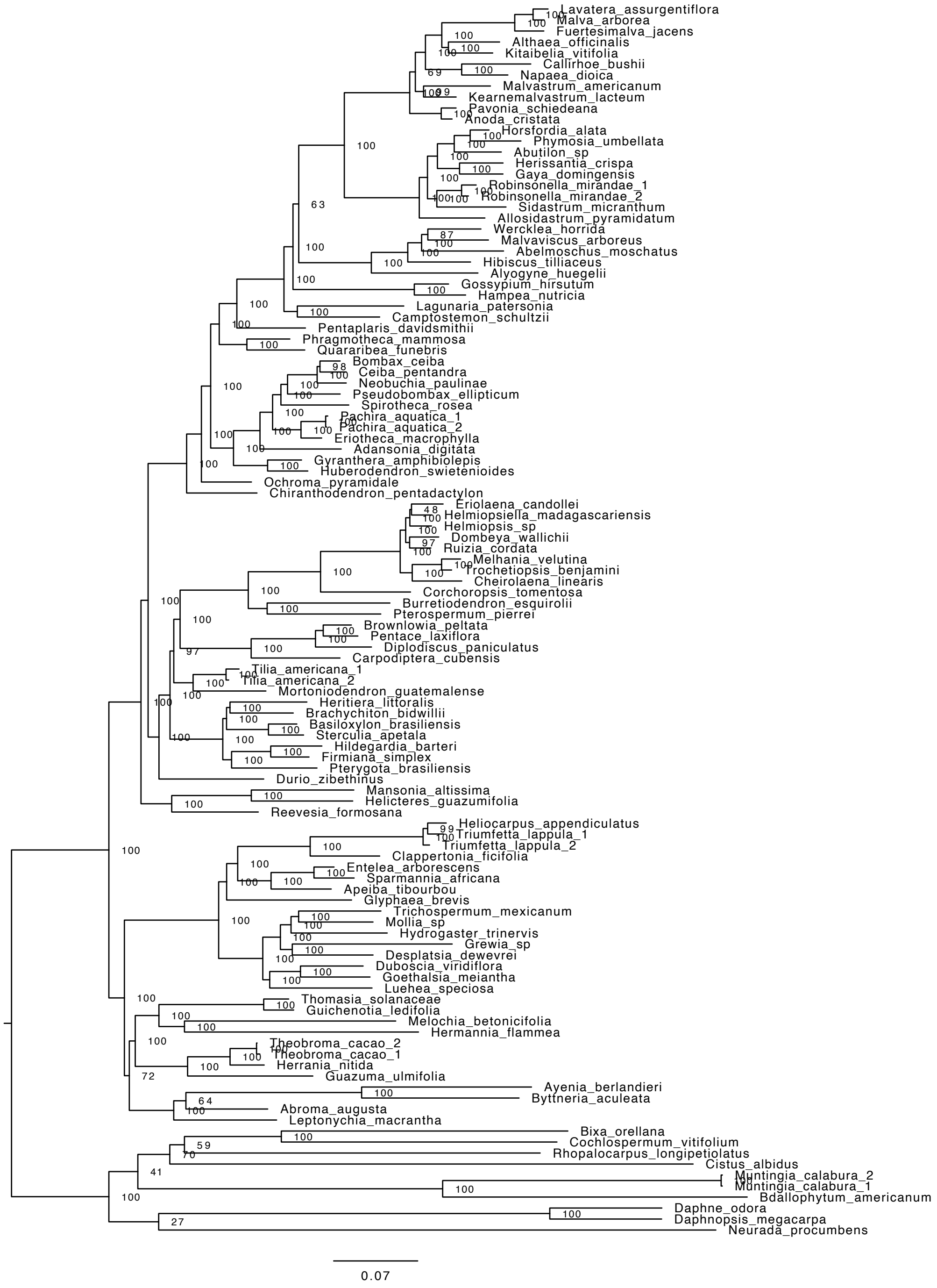

Figure S2. Species tree generated with SVDQuartets. Values associated to nodes correspond to Bootstrap suppot.

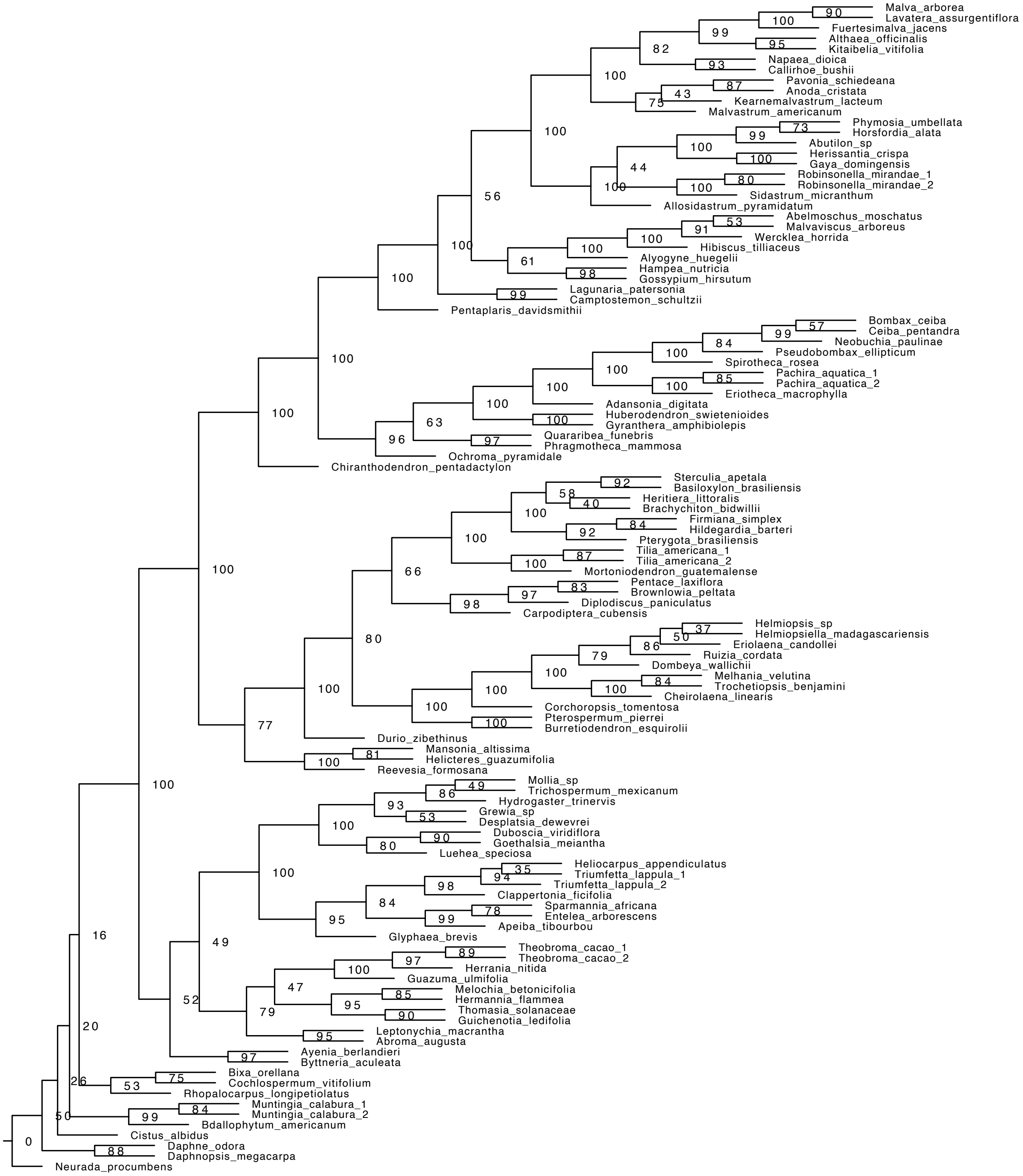

Figure S3. Species tree generated with ASTRAL-III. Values associated to nodes represent local posterior probability.

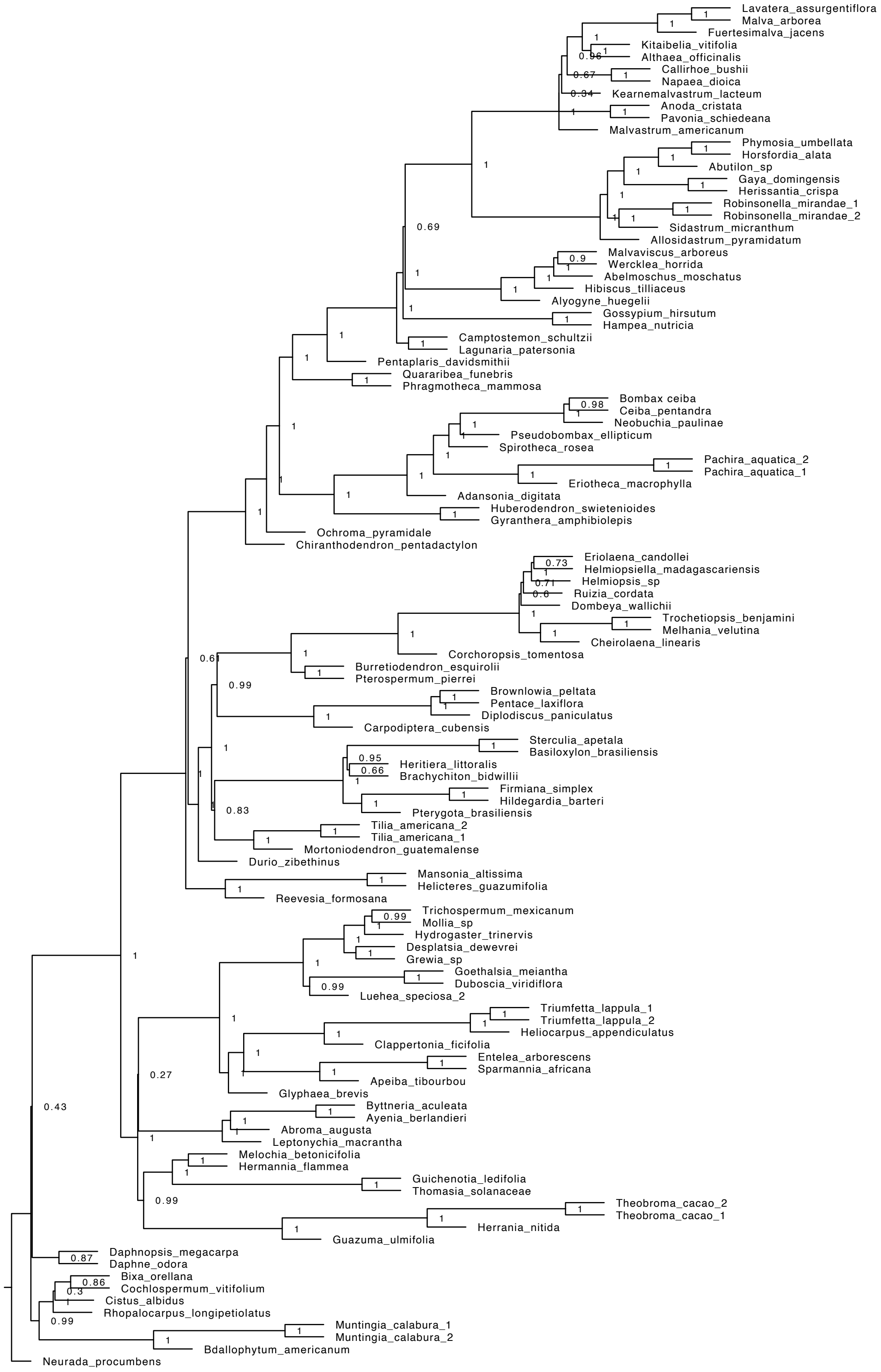

Figure S4. Species tree generated with ASTRAL-III. Values associated to nodes represent quartet score.

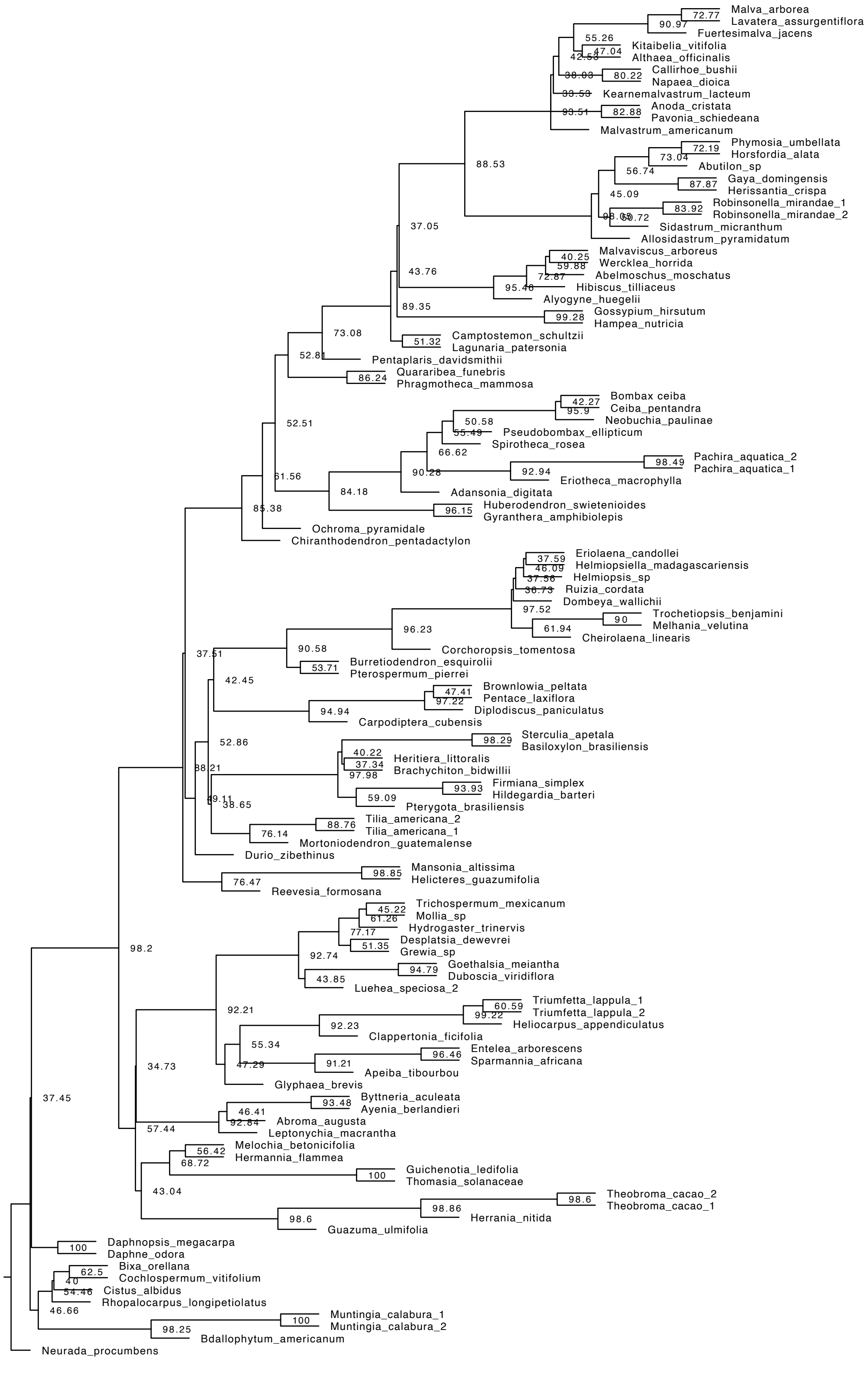

Figure S5. Comparison between prior information and divergence time estimates. The graphics show the groups where a calibration prior was applied, and for the five dating analyses (set1, set2, set3, concat 3 sets, and concat 5 loci) compared to the analysis using only the prior information (i.e., without molecular data).

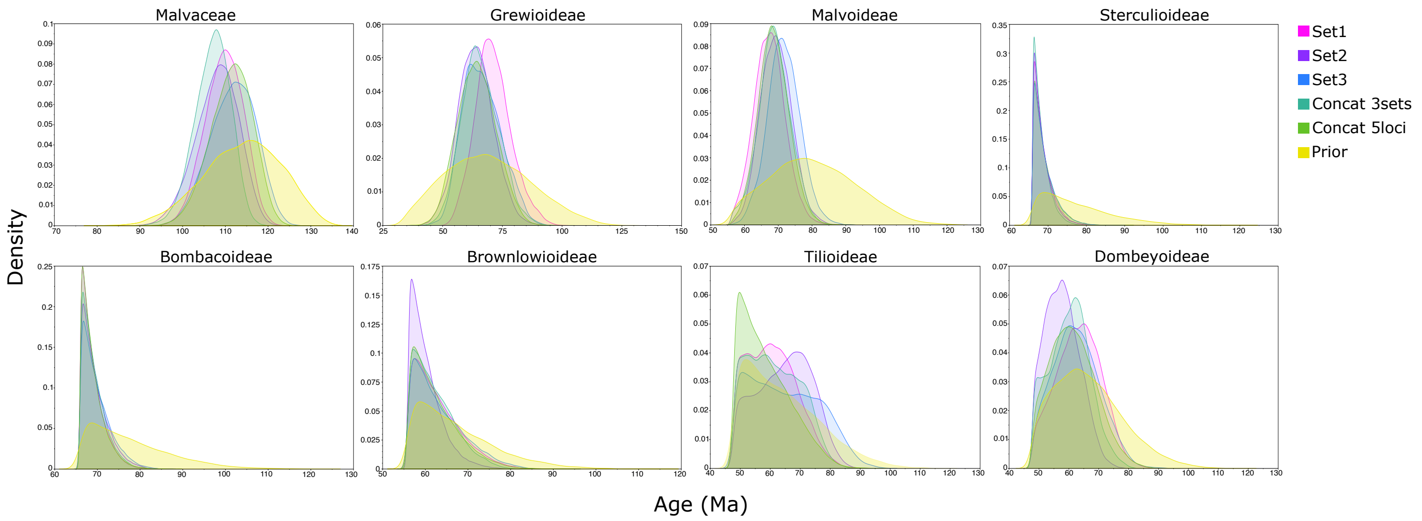

Supplement: Supplementary file 3 [file Data_Sheet_3.PDF]
